# Discovery of a Highly Potent and Selective Dual PROTAC Degrader of CDK12 and CDK13

**DOI:** 10.1021/acs.jmedchem.2c00384

**Published:** 2022-08-08

**Authors:** Jianzhang Yang, Yu Chang, Jean Ching-Yi Tien, Zhen Wang, Yang Zhou, Pujuan Zhang, Weixue Huang, Josh Vo, Ingrid J. Apel, Cynthia Wang, Victoria Zhixuan Zeng, Yunhui Cheng, Shuqin Li, George Xiaoju Wang, Arul M. Chinnaiyan, Ke Ding

**Affiliations:** † International Cooperative Laboratory of Traditional Chinese Medicine Modernization and Innovative Drug Discovery of Chinese Ministry of Education (MOE), Guangzhou City Key Laboratory of Precision Chemical Drug Development, College of Pharmacy, Jinan University, 855 Xingye Avenue East, Guangzhou 511400, People’s Republic of China; ‡ Michigan Center for Translational Pathology, University of Michigan, Ann Arbor, Michigan 48109, United States; § State Key Laboratory of Bioorganic and Natural Products Chemistry, Shanghai Institute of Organic Chemistry, Chinese Academy of Sciences, #345 Ling Ling Road, Shanghai 200032, People’s Republic of China; ∥ Department of Pathology, University of Michigan, Ann Arbor, Michigan 48109, United States; ⊥ Department of Computational Medicine and Bioinformatics, University of Michigan, Ann Arbor, Michigan 48109, United States; # Howard Hughes Medical Institute, University of Michigan, Ann Arbor, Michigan 48109, United States; ∇ Department of Urology, University of Michigan, Ann Arbor, Michigan 48109, United States; ○ Institute of Basic Medicine and Cancer (IBMC), Chinese Academy of Sciences, Hangzhou, Zhejiang 310022, People’s Republic of China; ◆ The First Affiliated Hospital (Huaqiao Hospital), Jinan University, 601 Huangpu Avenue West, Guangzhou 510632, China

## Abstract

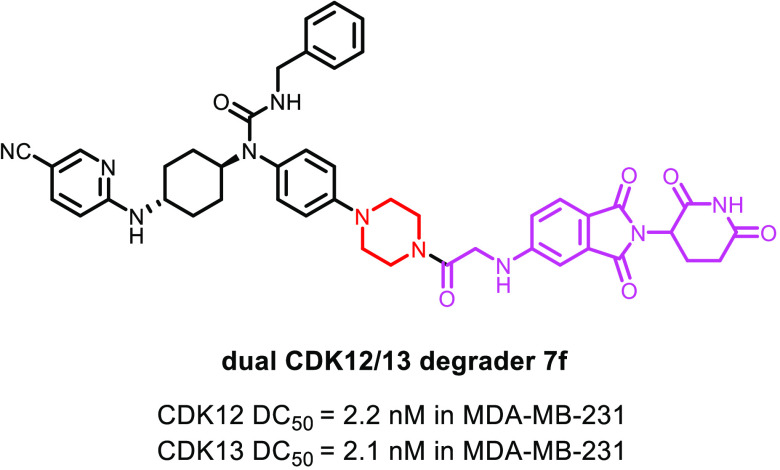

Selective degradation of the cyclin-dependent kinases 12 and 13 (CDK12/13) presents a novel therapeutic opportunity for triple-negative breast cancer (TNBC), but there is still a lack of dual CDK12/13 degraders. Here, we report the discovery of the first series of highly potent and selective dual CDK12/13 degraders by employing the proteolysis-targeting chimera (PROTAC) technology. The optimal compound **7f** effectively degraded CDK12 and CDK13 with DC_50_ values of 2.2 and 2.1 nM, respectively, in MDA-MB-231 breast cancer cells. Global proteomic profiling demonstrated the target selectivity of **7f**. *In vitro*, **7f** suppressed expression of core DNA damage response (DDR) genes in a time- and dose-dependent manner. Further, **7f** markedly inhibited proliferation of multiple TNBC cell lines including MFM223, with an IC_50_ value of 47 nM. Importantly, **7f** displayed a significantly improved antiproliferative activity compared to the structurally similar inhibitor **4**, suggesting the potential advantage of a CDK12/13 degrader for TNBC targeted therapy.

## Introduction

Triple-negative breast cancer (TNBC), characterized by the absence of estrogen receptor (ER), progesterone receptor (PR), and human epidermal growth factor receptor 2 (HER2), is a highly aggressive breast cancer lacking efficient targeted therapies.^1−6^ Although poly(ADP-ribose) polymerase (PARP) inhibitors (*e.g.*, Olaparib and Talazoparibtosylate)^7,8^ and the trophoblast cell surface antigen 2 (Trop2)-targeted antibody–drug conjugate (ADC) (Sacituzumab govitecan)^9^ have been approved for the treatment of TNBC, they are only effective in a limited number of patients.^10,11^ For example, PARP inhibitors were designated for ∼20% of TNBC patients with BRCA1/2 mutations,^7^ and Trop2 ADC was approved for the relapsed metastatic TNBC patients with failures of at least two prior therapies.^9^ To date, chemotherapy remains the standard treatment option for most TNBC patients,^11−13^ therefore, the development of novel targeted therapies for TNBC is highly desirable.

The cyclin-dependent kinases 12 and 13 (CDK12/13) are transcription-associated CDK that are complexed with cyclin K (CCNK) to regulate gene transcription (*e.g.*, DNA damage response (DDR) genes) by phosphorylating the C-terminal domain (CTD) of RNA polymerase II (RNAP II).^14−18^ Collective studies suggest that CDK12/13 are potential therapeutic targets for TNBC treatment.^16,19−22^ For example, downregulation of CDK12 or CDK13 by Clustered Regularly Interspaced Short Palindromic Repeats (CRISPR) technology was shown to reduce expression of some DDR genes and colony formation of TNBC MDA-MB-231 cells, and the suppressing effects were recapitulated when both CDK12/CDK13 were deleted.^21^ Moreover, CDK12 silencing induced DNA damage and apoptosis, while CDK13 knockdown triggered apoptosis without inducing a DNA damage signal, underlying the importance of dual inhibition of CDK12/CDK13 to induce TNBC cell death. Indeed, a selective dual inhibitor of CDK12/CDK13, SR-4835, exhibited highly promising antiproliferative activity both *in vitro* and *in vivo*.^21^

Several classes of small-molecule inhibitors of CDK12/13 kinases have been developed;^21,23−27^ however, most of these compounds eventually induced mutation-mediated resistance.^25,27,28^ Additionally, noncatalytic functions of CDK12/13 beyond CTD phosphorylation were also characterized in cancers, which would not be modulated by the CDK12/13 kinase inhibitors.^29−32^ Proteolysis targeting chimeras (PROTACs), which hijack the ubiquitin-proteasome system to degrade a target protein, have become a novel drug discovery paradigm.^33−37^ Different from the ATP competitive kinase inhibitors that function in an occupancy-driven manner, PROTAC-mediated kinase degradation is a catalytic and event-driven process, making them less likely to induce resistance mutations. Moreover, by depleting the protein scaffold, PROTACs can regulate both the catalytic and noncatalytic functions of the kinase.^38^ Therefore, selective degradation of CDK12/13 by using PROTAC technology may address the above-mentioned issue regarding CDK12/13 kinase inhibitors, representing a novel targeted therapeutic opportunity for TNBC.^38^

**Figure 1:**
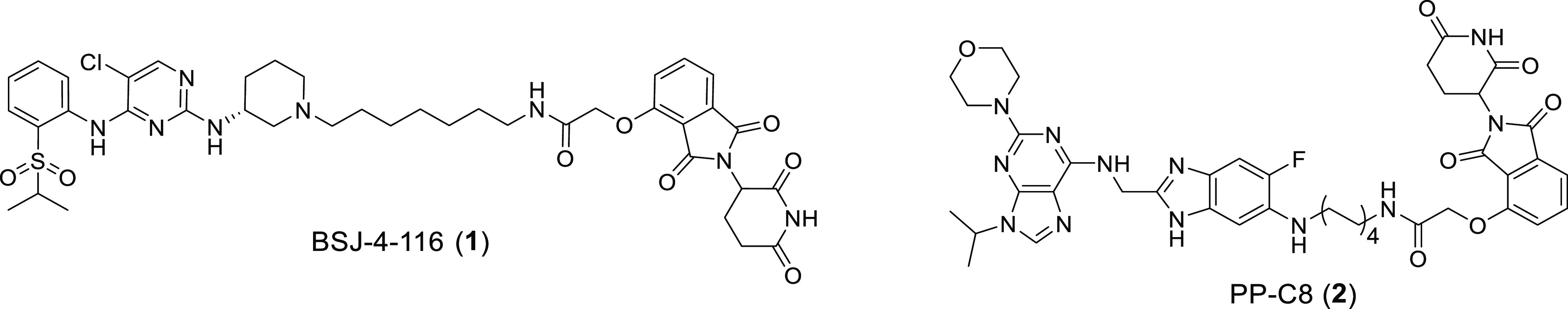
Chemical structures of the previously reported selective CDK12 PROTACs.

Recently, selective CDK12 PROTACs BSJ-4-116 (**1**)^28^ and PP-C8 (**2**)^39^ were reported (Figure 1). Compound **1** was the first isoform-selective CDK12 degrader derived from a dual CDK12/13 covalent inhibitor THZ531. Quantitative proteomics demonstrated the selectivity of BSJ-4-116 for CDK12 over other targets including CDK13. Compound **2** was also a highly selective CDK12 degrader, which was designed by connecting SR-4835, a noncovalent dual inhibitor of CDK12/13, with a ligand for the E3 ligase cereblon (CRBN). However, there is no selective CDK12/13 dual degrader reported to date. In this study, we designed and characterized the first series of dual CDK12/13 degraders, among which the optimal compound **7f** degraded both CDK12 and CDK13 with DC_50_ values of 2.2 and 2.1 nM, respectively. More importantly, compound **7f** exhibited a high selectivity to CDK12/13 as assessed by global proteomics, representing a potential lead molecule for further development of CDK12/13 degraders as new targeted therapy for TNBC patients.

## Results and Discussion

### Design of CDK12/13 PROTAC Degraders

**Figure 2:**
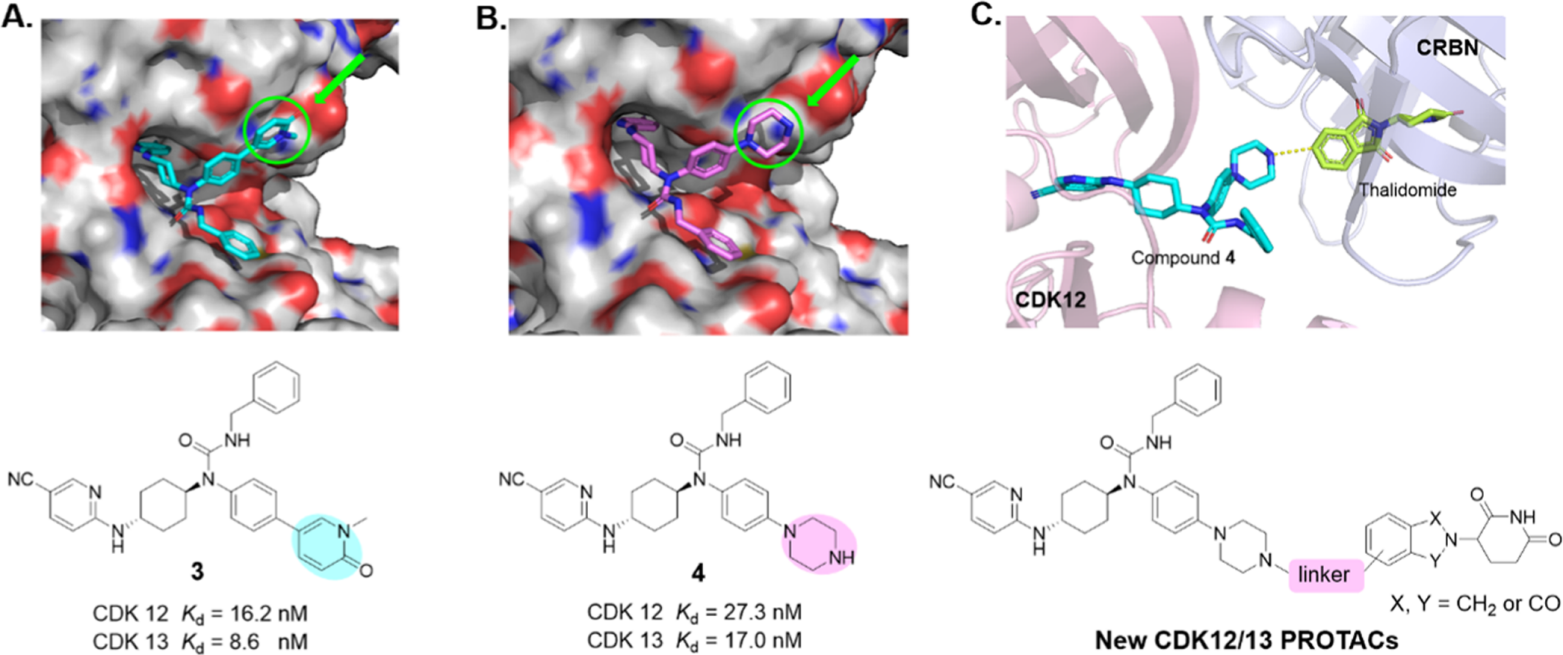
Design of CDK12/13 PROTACs based on cereblon ligands. Docking models of compounds **3** (A) and **4** (B) with CDK12 (PDB: 6CKX). (C) Design of new CDK12/13 PROTACs based on thalidomide or lenalidomide.

The heterobifunctional PROTAC molecules are known to consist of three elements: a ligand for the protein of interest (POI), a linker, and a ligand for recruiting an E3 ligase.^33,40,41^ Design of new PROTACs was started from a previously reported CDK12/13 dual inhibitor **3** (Figure 2A), which bound tightly to CDK12 and CDK13 with *K*_d_ values of 16.2 and 8.6 nM (Figure S1), respectively, and exhibited an excellent kinome-wide selectivity.^26^ The computational modeling study suggested that 1-methylpyridin-2(1*H*)-one group of compound **3** extended to the solvent-exposed area (Figure 2A), which may be replaced by the hydrophilic piperazinyl moiety to facilitate E3 ligase ligand tethering without compromising its binding affinity. The resulting compound **4** indeed displayed a similar binding affinity with CDK12/13 as compound **3** (Figures 2B and S1), indicating that it could be feasible to use the piperazinyl group in compound **4** as the E3 ligase ligand tethering site. Thalidomide and lenalidomide were selected as the E3 ligase ligand because both have been widely adopted in various PROTACs, especially in clinically investigated PROTAC degraders.^42^ Thus, the new CDK12/13 PROTACs were designed by connecting compound **4** with thalidomide/lenalidomide through various linkers (Figure 2C). The degradation efficiency of these new PROTACs was assessed by immunoblotting assays in MD-MBA-231 TNBC cells, which harbor high levels of CDK12 and CDK13^21^ after treatment for 15 h.

### Chemical Synthesis

**Scheme 1:**
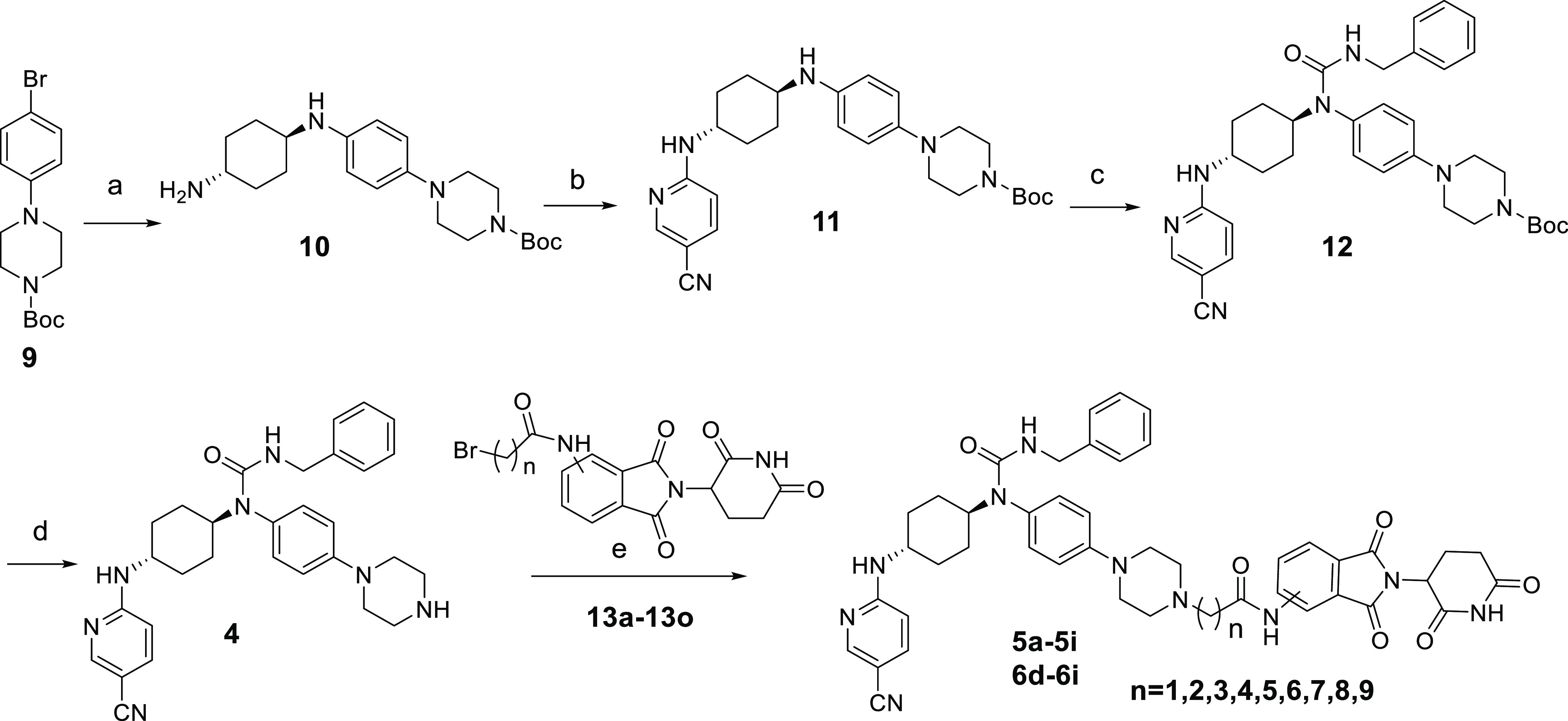
Synthesis of Compounds **5a**–**5i** and **6d**–**6i** Reagents and conditions: (a) *trans*-1,4-diaminocyclohexane, d-proline, CuI, K_3_PO_4_, dry dimethyl sulfoxide (DMSO), 100 °C, 10 h, 44%; (b) 5-cyano-2-fluoropyridine, Cs_2_CO_3_, *N,N*-dimethylformamide (DMF), rt, overnight, 92%; (c) benzyl isocyanate, *N,N*-diisopropylethylamine (DIPEA), DMF, 95 °C, 4 h, 52%; (d) trifluoroacetic acid (TFA), dichloromethane (DCM), 50 °C, overnight, 79%; (e) KHCO_3_, 80 °C, overnight, 28–62%.

The synthetic routes for compounds **5a**–**5i** and **6d**–**6i** are illustrated in Scheme 1. The Ullmann coupling reaction between *tert*-butyl 4-(4-bromophenyl)piperazine-1-carboxylate and *trans*-1,4-diaminocyclohexane produced compound **10**, which went through the nucleophilic substitution reaction with 5-cyano-2-fluoropyridine to yield compound **11**. Reaction of compound **11** with benzyl isocyanate produced compound **12**. The key intermediate **4** was obtained after deprotection of the Boc group on compound **12**. Finally, compound **4** was reacted with various thalidomide derivatives to generate the final compounds **5a**–**5i** and **6d**–**6i**.

**Scheme 2:**
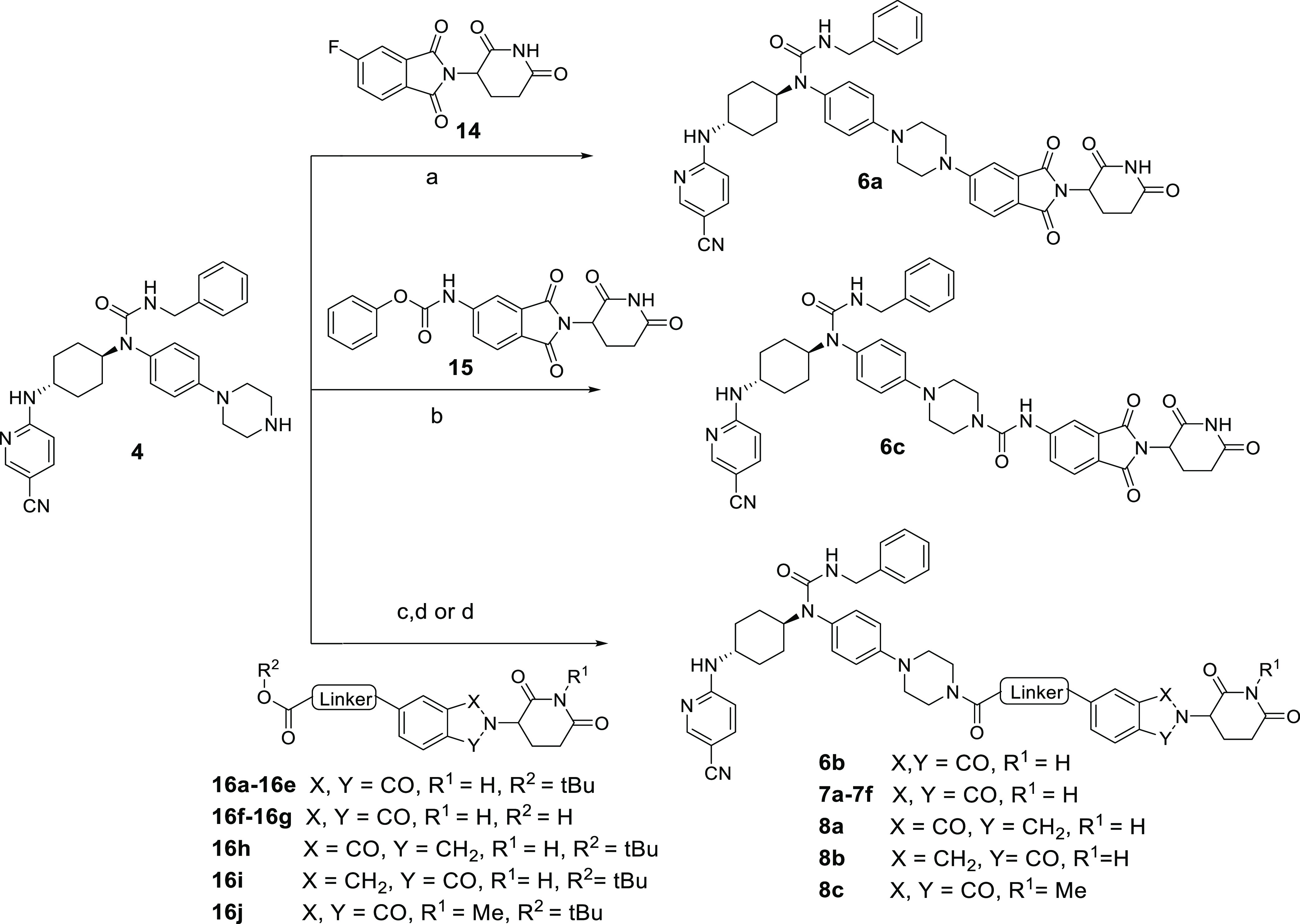
Synthesis of Compounds **6a**–**6c**, **7a**–**7f**, and **8a**–**8c** Reagents and conditions: (a) DMSO, DIPEA, 120 °C, 8 h, 63%; (b) CH_3_CN, DMF, DIPEA, 4-dimethylaminopyridine (DMAP), 60 °C, 4 h, 44%; (c) TFA, DCM, 2 h, rt, 60–90%; (d) 2-(7-azabenzotriazol-1-yl)-*N,N,N*′*,N*′-tetramethyluronium hexafluorophosphate (HATU), DIPEA, DMF, rt, 15 min, 65–86%.

The synthesis of compounds **6a**–**6c**, **7a**–**7f**, and **8a**–**8c** is outlined in Scheme 2. Compound **6a** was prepared through nucleophilic substitution reaction between the fluoro-substituted thalidomide **14** and compound **4**. Compound **6c** was obtained by reacting compound **15** with compound **4**. Amidation of the piperazine group in compound **4** produced the final compounds **6b**, **7a**–**7f**, and **8a**–**8c**.

### Optimization of CDK12/13 PROTAC Degraders

The first batch of compounds (**5a**–**5i**) was designed by attaching compound **4** to the position 4 of pomalidomide through the linear linkers with various lengths. The results in Table 1 and Figure S2 showed that compound **5f** with the amide-containing eight-atom linker was the most potent degrader with degradation efficiency of 75 and 56% for CDK12 and CDK13 at 1.0 *μ*M, respectively. Further increase of the linker length resulted in compounds **5h** and **5i** with loss of potency, especially for compound **5i** with an 11-atom linker, which was completely inactive.

**Table 1:**
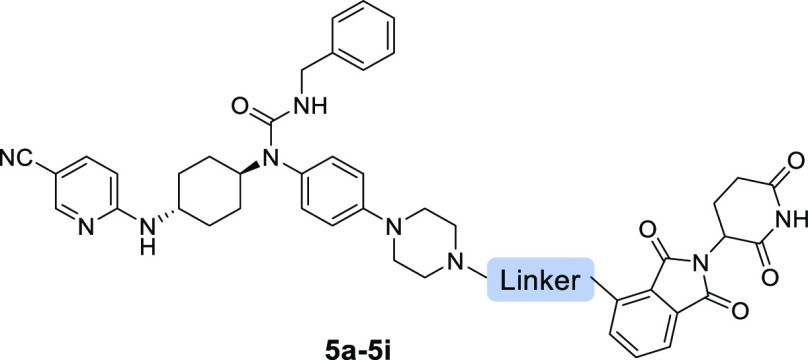

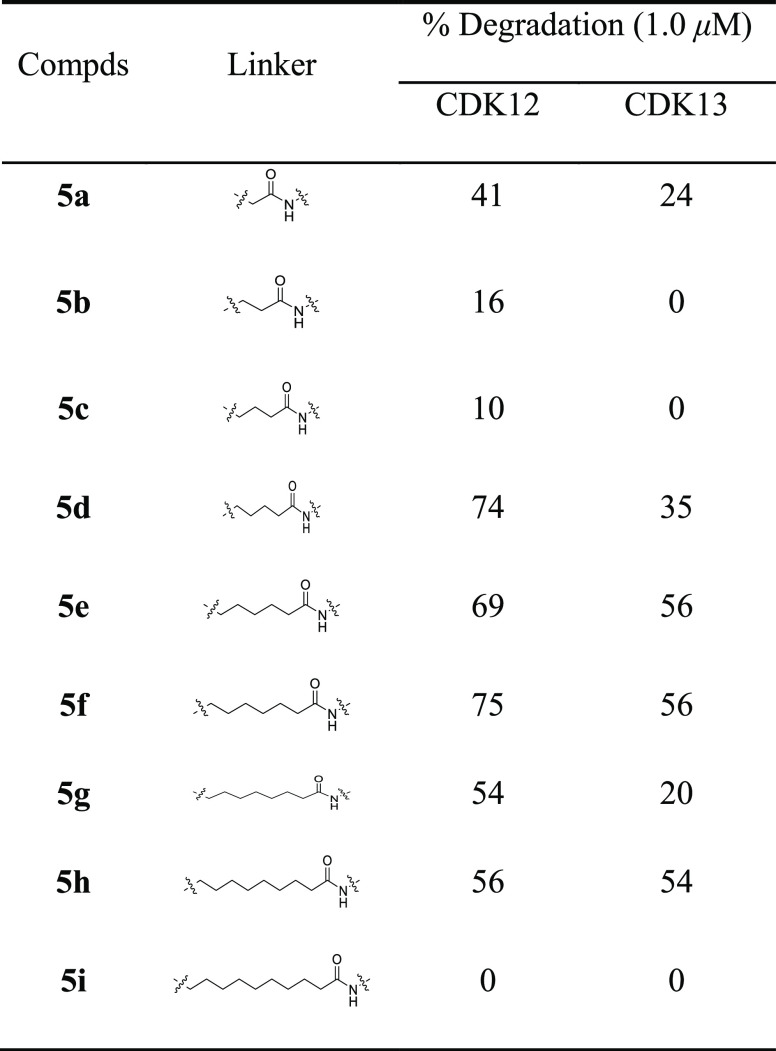
Degradation Efficiency of CDK12/13 Degraders **5a**–**5i**^a^

a MDA-MB-231 cells were treated with the indicated compounds at 1.0 μM for 15 h. CDK12/13 protein levels were determined by immunoblotting and normalized against α-tubulin.

The second batch of compounds (**6a**–**6i**) was prepared by switching the attachment point of compound **4** from position 4 to position 5 of pomalidomide, and the degradation efficiency for these compounds is summarized in Table 2 and Figure S2. Compared to the first batch, this series of compounds was generally more potent. The top three potent compounds (**6c**–**6e**) with short linkers bearing the amide were shown to degrade CDK12 and CDK13 by more than 95 and 60%, respectively. Further increasing (**6g**–**6i**) or decreasing (**6a**–**6b**) the linker length led to the reduction of degradation efficiency. These results suggested that a linker with a length between 2 and 4 atoms at the position 5 of thalidomide is optimal for CDK12/13 degradation.

**Table 2:**
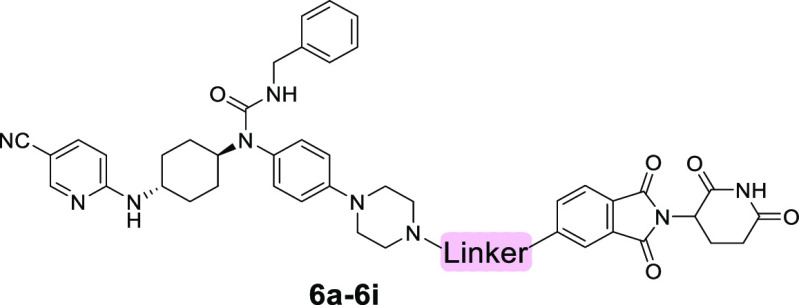

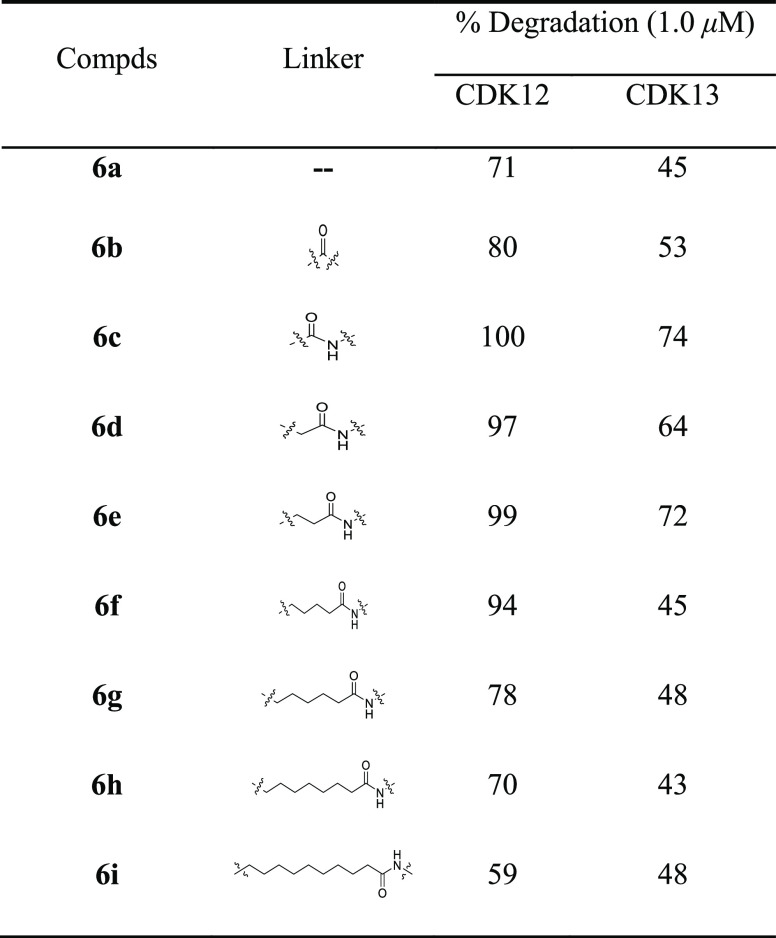
Degradation Efficiency of CDK12/13 Degraders **6a**–**6i**^a^

a MDA-MB-231 cells were treated with the indicated compounds at 1.0 μM for 15 h. CDK12/13 protein levels were determined by immunoblotting and normalized against α-tubulin.

Given that the linker composition has an important effect on the potency of degraders,^43−45^ the third batch of compounds was constructed by using various three-atom linkers between compound **4** and the position 5 of thalidomide. As shown in Table 3 and Figure S2, the degradation efficiencies of CDK12 and CDK13 of compounds **7a**, **7c**, and **7d** featuring the linkers of alkynyl, cyclopropyl, and methylene groups, respectively, were dramatically decreased, whereas the compounds **7b**, **7e**, and **7f** bearing other linkers maintained the potent degradation effects (Table 3 and Figure S2). Compound **7f**, containing a −CH_2_–NH– in the linker, degraded CDK12 and CDK13 with the efficiencies of 88 and 74%, respectively, at 1.0 μM. Based on these results, two additional compounds **8a** and **8b** were designed by replacing thalidomide in compound **7f** with lenalidomide. These two compounds were found to be slightly less effective in the degradation of CDK12 and CDK13 than compound **7f** (Table 4 and Figure S2).

**Table 3:**
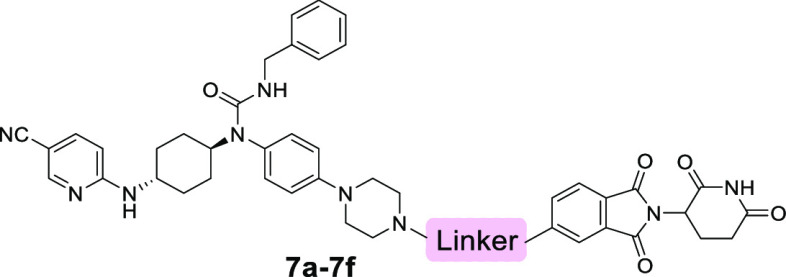

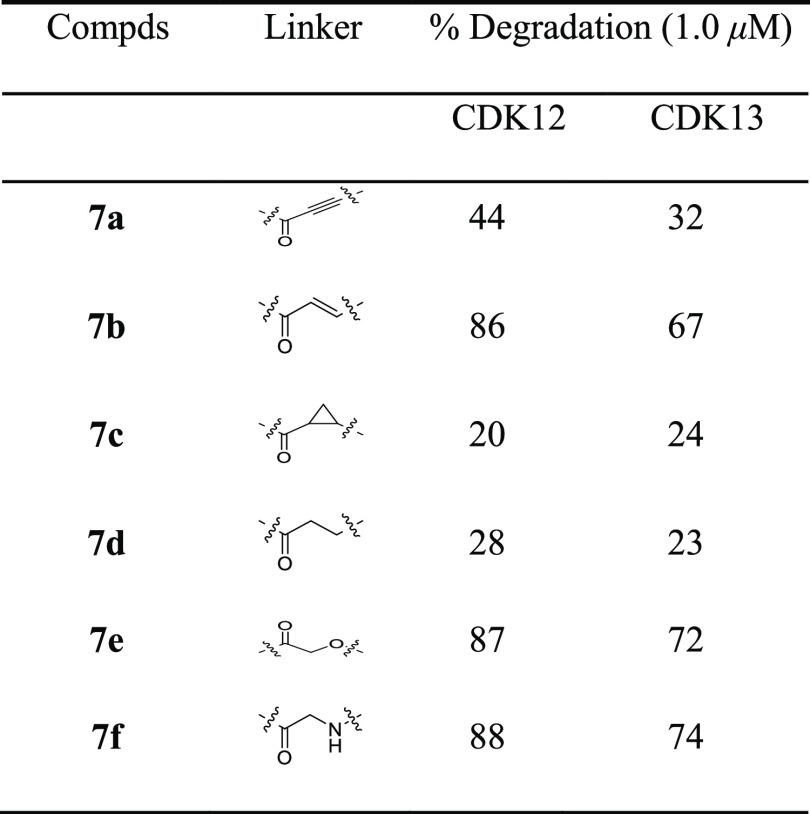
Degradation Efficiency of PROTAC CDK12/13 Degraders **7a**–**7f**^a^

a MDA-MB-231 cells were treated with the indicated compounds at 1.0 μM for 15 h. CDK12/13 protein levels were determined by immunoblotting and normalized against α-tubulin.

**Table 4:**
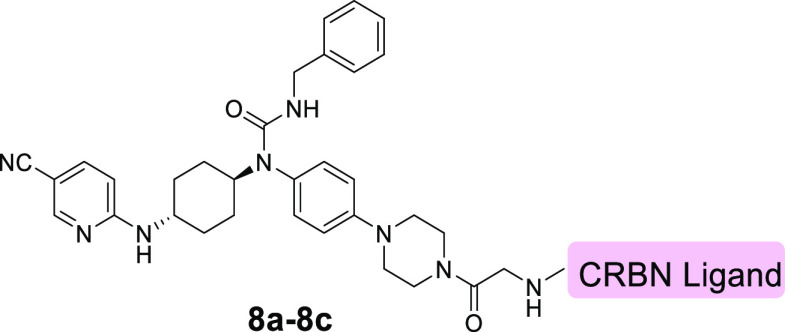

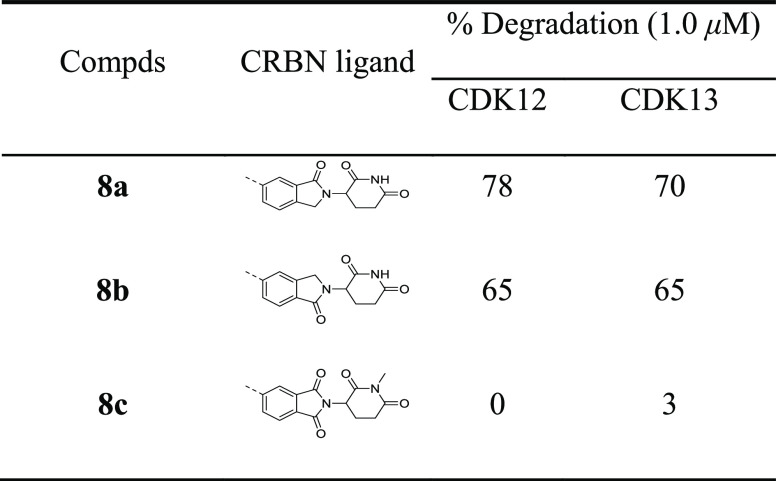
Degradation Efficiency of PROTAC CDK12/13 Degraders **8a**–**8c**^a^

a DA-MB-231 cells were treated with the indicated compounds at 1.0 μM for 15 h. CDK12/13 protein levels were determined by immunoblotting and normalized against α-tubulin.

To select the optimal compound for further evaluation, we determined the DC_50_ and DC_90_ values by immunoblotting the compounds with degradation efficiencies greater than 60% for both CDK12 and CDK13. The results are summarized in Table 5 and Figure S3. It was shown that compound **7f** degraded CDK12 and CDK13 in a dose-dependent manner with DC_50_ and DC_90_ of 2.2, 2.1 nm and 33.3, 21.6 nM, respectively, representing the most potent dual CDK12/13 degrader among the compounds evaluated. Compound **8c**, which is a methylated derivative of compound **7f**, is totally inactive for both CDK12 and CDK13 because the methylation abolishes the binding of pomalidomide to CRBN.^33^ Compound **8c** was also utilized as a negative control for further biological investigation.

**Table 5:** DC_50_ and DC_90_ Values of Selected Compounds in MD-MBA-231 Cells^a^

	CDK12		CDK13	
compds	DC_50_ (nM)	DC_90_ (nM)	DC_50_ (nM)	DC_90_ (nM)
**6c**	6.8	52.3	4.5	17.1
**6d**	35.5	166.9	7.1	7.7
**6e**	4.4	181.7	12.5	101.1
**7b**	33.1	386.6	20.7	198.5
**7e**	3.9	50.6	36.0	427.9
**7f**	2.2	33.3	2.1	21.6
**8a**	19.3	99.3	6.2	76.6
**8b**	5.5	721.6	8.5	184.6

a Degradation potencies (DC_50_ and DC_90_) were determined by immunoblotting after treatment with the degraders in MDA-MB-231 cells for 15 h.

### Global Proteomic Profiling of CDK12/13 Degrader **7f**

**Figure 3:**
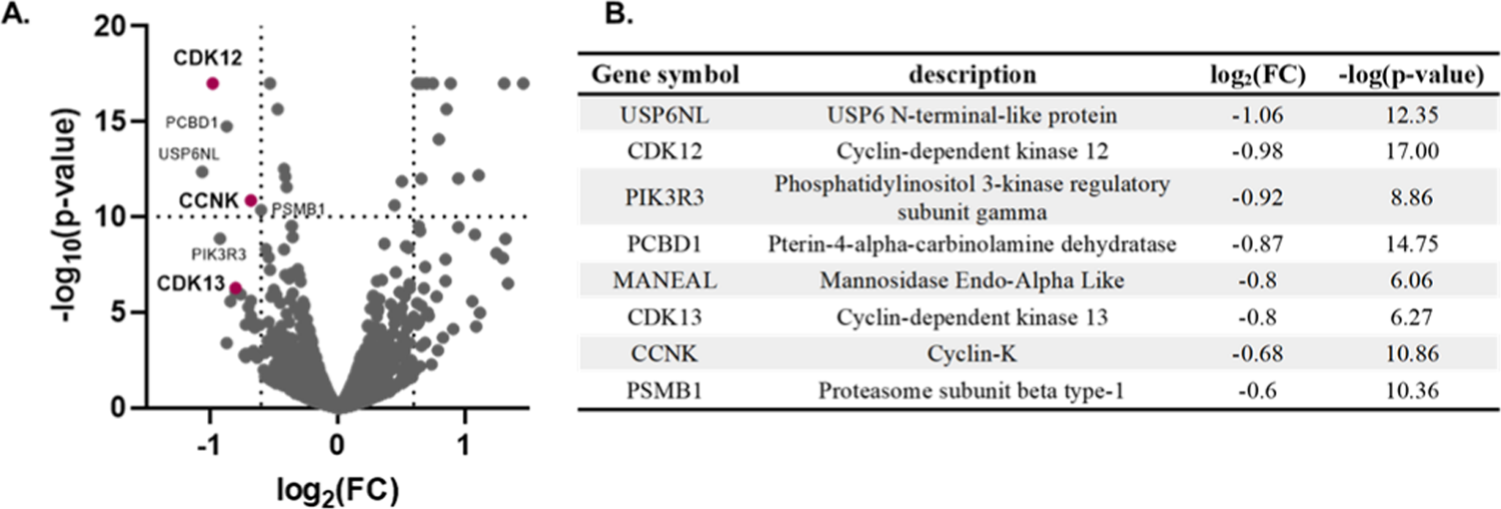
(A) Global proteomic analyses of MFM223 cells after treatment with **7f** for 5 h. (B) Top decreased proteins after treatment with **7f**.

To investigate the selectivity of the CDK12/13 degraders, we performed the global proteomic profiling study by the Tandem Mass Tag (TMT)-based quantitative proteomics in MFM223 cells, which is a TNBC cell line with high CDK12/13 expression, after treatment with compound **7f** at 500 nM for 5 h. The volcano plot clearly shows that CDK12, CDK13, and CCNK (a partner protein of CDK12 and CDK13) are among the top significantly degraded proteins (Figure 3A,B), indicating that **7f** was highly selective for CDK12 and CDK13.

The other top decreased proteins (Figure 3B, Table S2) included phosphoinositide-3-kinase regulatory subunit 3 (PIK3R3), USP6 N-terminal-like protein (USP6NL), proteasome subunit β type-1 (PSMB1), and pterin-4 α-carbinolamine dehydratase 1 (PCBD1). PIK3R3 is a regulatory subunit of PI3K.^46,47^ It is well known that PIK3R3 is overexpressed in different types of cancer.^48,49^ Knockdown of PIK3R3 induced G0/G1 cell cycle arrest.^49^ USP6NL is up-regulated and functions as an oncogene in human breast cancer and colorectal cancer. Overexpression of USP6NL enhanced cell proliferation and promoted cell cycle progression from the G0/G1 phase to the S phase.^50,51^ PSMB (proteasome β subunit family), one component of the ubiquitin–proteasome system, plays an important role in tumor cells and immune cells.^52^ Particularly, PSMB4 facilitates breast cancer progression by promoting cell proliferation and viability.^53^ PCBD (pterin-4a-carbinolamine dehydratase (PCD)) is a bifunctional protein. In the cytoplasm, it acts as a dehydratase, whereas in the nucleus, it increases the transcriptional activity of hepatocyte nuclear factor 1 (HNF1).^54,55^

Except for PCBD1, all of these “off-target” proteins were oncogenes involved in regulating cell proliferation and cell cycle progression, a phenotype observed in cells upon inhibition of CDK12/13 function. The altered proteins identified from proteomic profiling might also be the downstream responses upon CDK12/13 degradation by compound **7f**. We will further characterize and validate these proteins and investigate the mechanism of degradation of these “off-targets”.

### *In Vitro* Assessment of Compound **7f** in TNBC Cells

**Figure 4:**
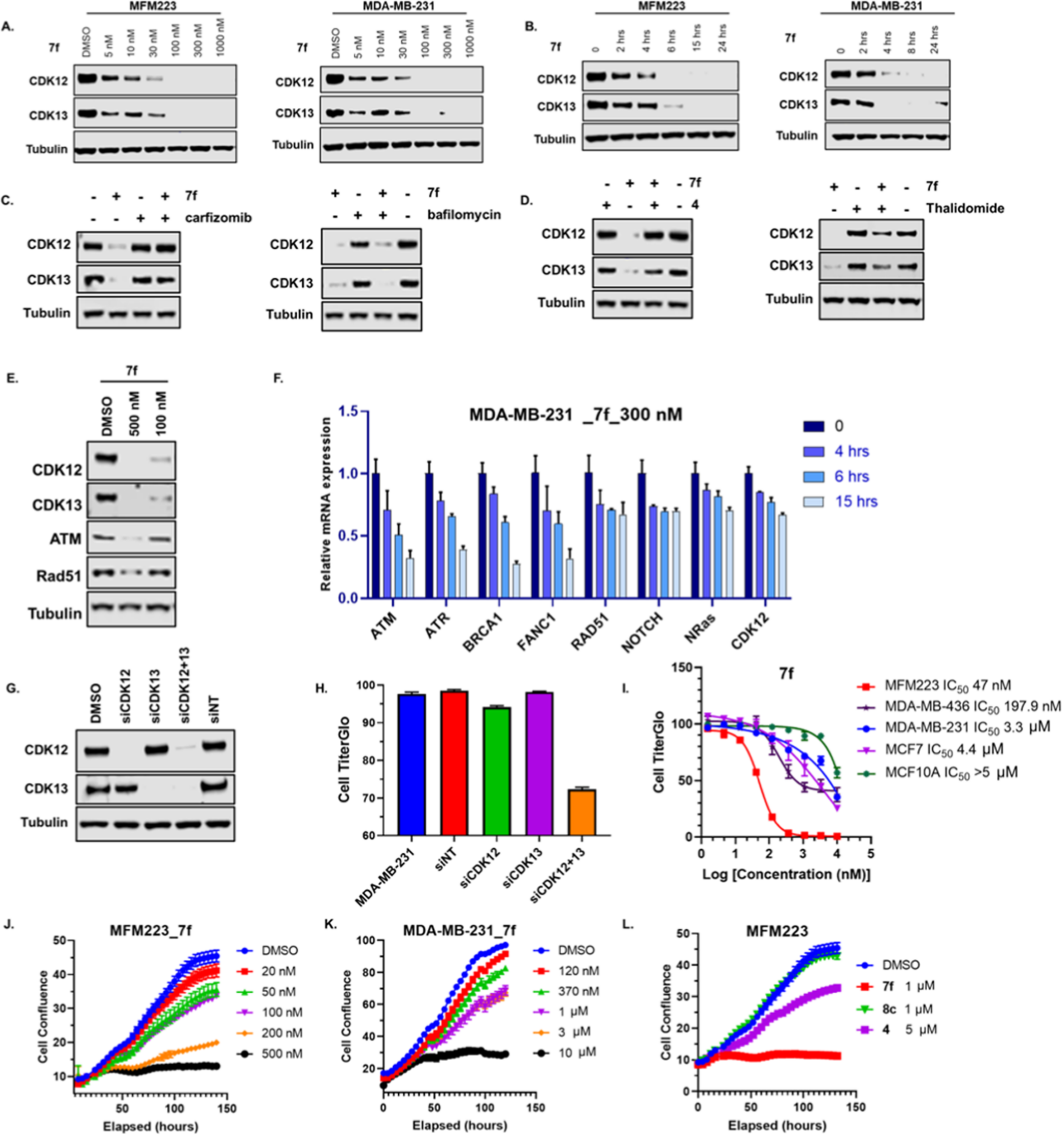
(A) Dose-dependent degradation by compound **7f** on CDK12 and CDK13 in MFM223 and MDA-MB-231 cells. (B) Time-dependent degradation by compound **7f** on CDK12 and CDK13 in MFM223 and MDA-MB-231 cells. (C) MD-MBA-231 cells were pretreated with DMSO, carfizomib (100 nM), or bafilomycin (50 nM) for 1 h, followed by treatment with **7f** (500 nM) for an additional 6 h. (D) MD-MBA-231 cells were treated with DMSO, **4** (5.0 μM), or thalidomide (5.0 μM) together with **7f** (500 nM) for 6 h. (E) Western blot analysis of ATM and Rad51 in MDA-MB-231 cells after treatment with compound **7f** at different doses. (F) Expression of DDR genes in MDA-MB-231 cells by quantitative polymerase chain reaction (qPCR) at different time points upon the treatment with vehicle or **7f** (500 nM). (Three replicates per treatment, normalized to β-actin and DMSO). (G) Knockdown of CDK12 or/and CDK13 by siRNA for 72 h in MDA-MB-231 cells. (H) Cell viability assay of MDA-MB-231 cells after siCDK12 or/and siCDK13 for 5 days. (I) Cell viability assays in multiple breast cells treated by compound **7f**. (J) Growth inhibitory curves of compound **7f** in MFM223 cells with the indicated concentrations (20–500 nM). (K) Growth inhibitory curves of compound **7f** in MDA-MB-231 cells with the indicated concentrations (120–10 000 nM). (L) Antiproliferative activities of compounds **4**, **7f**, and **8c** in MFM223 cells.

The degradative effect of compound **7f** was further confirmed in MFM223 and MDA-MB-231 cells. As shown in Figure 4A, compound **7f** significantly degraded both CDK12 and CDK13 in a dose-dependent manner in these cells. The kinetics of CDK12 and CDK13 degradation by compound **7f** were also evaluated. As shown in Figure 4B, treatment of compound **7f** at a concentration of 500 nM almost completely degraded CDK12 and CDK13 proteins after treatment for 4 h, indicating the fast kinetics of **7f** in degradation of CDK12 and CDK13.

We further investigated the mechanism of CDK12/13 degradation by compound **7f** in MDA-MB-231 cells. Western blot analysis showed that while compound **7f** at 500 nM effectively reduced the level of CDK12 proteins in MDA-MB-231 cells after treatment for 6 h, pretreatment with the proteasome inhibitor carfizomib^56^ completely blocked the degradation of CDK12 and CDK13 mediated by compound **7f** (Figure 4C). Addition of the lysosomal inhibitor bafilomycin^57^ did not affect the effect of compound **7f** on CDK12/13. It is worth noting that neither carfizomib nor bafilomycin had an effect on CDK12/13. These results demonstrated that compound **7f** induced CDK12 and CDK13 degradation indeed through the ubiquitin–proteasome system. In addition, both the warhead compound **4** and thalidomide competitively inhibited the degradation of CDK12 and CDK13 by compound **7f** (Figure 4D).

We next assessed the effect of our dual CDK12/13 degrader on the expression of downstream DDR genes. As shown in Figure 4E, along with the degradation of CDK12 and CDK13, ATM and Rad51 were also decreased upon the treatment with compound **7f**. Figure 4F further shows that at a concentration of 500 nM in MDA-MB-231 cells, compound **7f** significantly suppressed the expression of a cast of DDR genes in a time-dependent manner, including *ATM*, *ATR*, *BRCA1*, and *FANCI*, consistent with the results from the genetic knockdown of CDK12/13.^14,21,27^

It has been reported that a double knockout of CDK12 and CDK13 showed a superior inhibition of cell growth compared to a single knockout of either CDK12 or CDK13.^21,58^ To confirm the results in breast cancer, we knocked down CDK12/13 genes in MDA-MB-231 cells by siRNA. Western blot showed that siRNA specifically and efficiently knocked down CDK12 or CDK13 after incubation for 72 h (Figure 4G). While single knockdown of CDK12 or CDK13 had no effect on cell growth, double knockdown of both CDK12 and CDK13 significantly inhibited the growth of MDA-MB-231 cells (Figure 4H). The antiproliferative effects of **7f** were next investigated in a panel of breast cancer and benign breast cell lines. Figure 4I shows that compound **7f** dramatically inhibited the cell growth of MFM223 and MDA-MB-436, both of which are BRCA-deficient TNBC cell lines, with IC_50_ values of 47 and 197.9 nM, respectively. However, the compound was obviously less effective in BRCA-proficient MDA-MB-231 TNBC cells. The MCF12A benign breast cells and MCF7 non-TNBC cells were also obviously less sensitive to **7f** with IC_50_ values greater than 5.0 μM.

The cell growth inhibitory effects were further confirmed by IncuCyte. As shown in Figure 4J,K, **7f** significantly suppressed the proliferation of MFM223 and MDA-MB-231 cells in a dose-dependent manner, while the negative control compound **8c** was almost totally inactive (Figure 4L). More importantly, compound **7f** was more potent in inhibiting the cell growth of MFM223 compared to the structurally similar CDK12/13 inhibitor **4**, which showed only a modest inhibitory effect on proliferation in MFM223 cells (Figure 4L). The outperformance of degrader **7f** over inhibitor **4** in this proliferation assay could be due to the dual depletion of both catalytic and scaffold functions of the kinase CDK12/13 by **7f**, which warrants further investigation.

**Figure 5:**
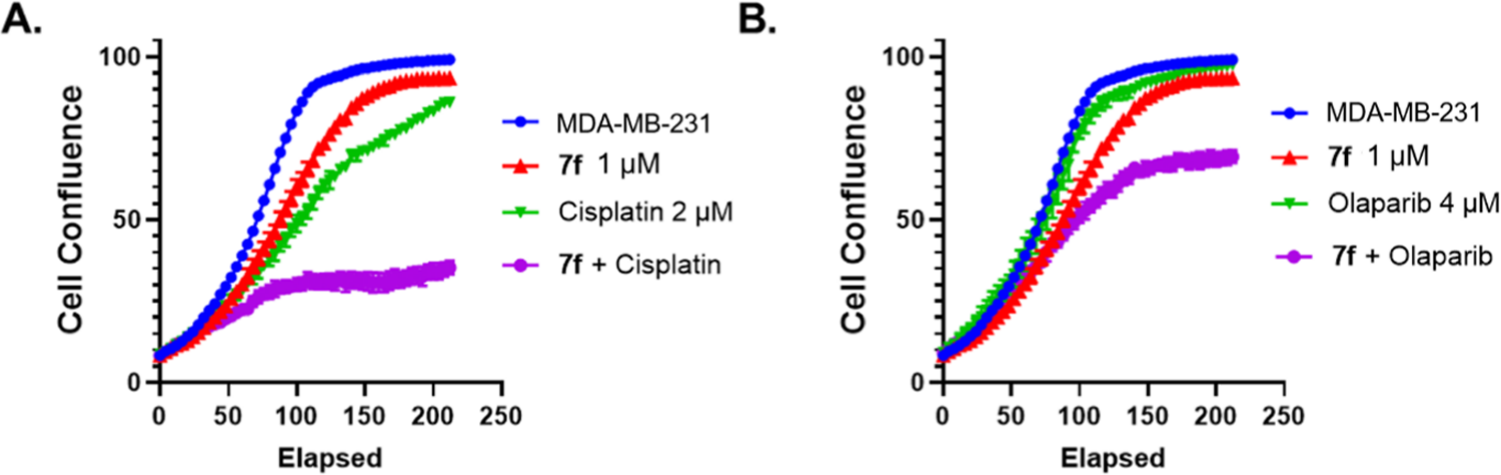
(A) Combination of **7f** with Cisplatin in MDA-MB-231 cells. (B) Combination of **7f** with Olaparib in MDA-MB-231 cells.

It was well documented that BRCA1/2 functions as DNA double-strand break (DSB) homologous recombination (HR) repair factors, and mutation or loss of BRCA1/2 (BRCAness) in breast cancers is deficient in the process of homologous recombination (HR-deficient).^59^ CDK12 inhibition causes a BRCAness phenotype by blocking homologous recombination in TNBC,^21^ and the CDK12 inhibitor THZ531 was previously reported to impair HR and prevent the damaged DNA from recruiting RAD51.^60^ Compound **7f** has less antiproliferative effect in BRCA-proficient MDA-MB-231 cells compared to BRCA-deficient MFM223 cells, indicating that BRCA1/2-mediated DNA damage repair might compromise the effectiveness of CDK12/13 degraders. We therefore hypothesized that a combination of compound **7f** with a DDR inhibitor could generate a synergistic effect. As shown in Figure 5A, the monotreatment of either **7f** or the DNA synthesis inhibitor (cisplatin) demonstrated a moderate growth inhibition on MDA-MB-231 cells. However, the combo-treatment of **7f** and cisplatin significantly suppressed cell proliferation. A similar synergistic effect was also observed for the combination of **7f** and a PARP inhibitor (Olaparib) (Figure 5B).

### Modeling of the Ternary Complex

**Figure 6:**
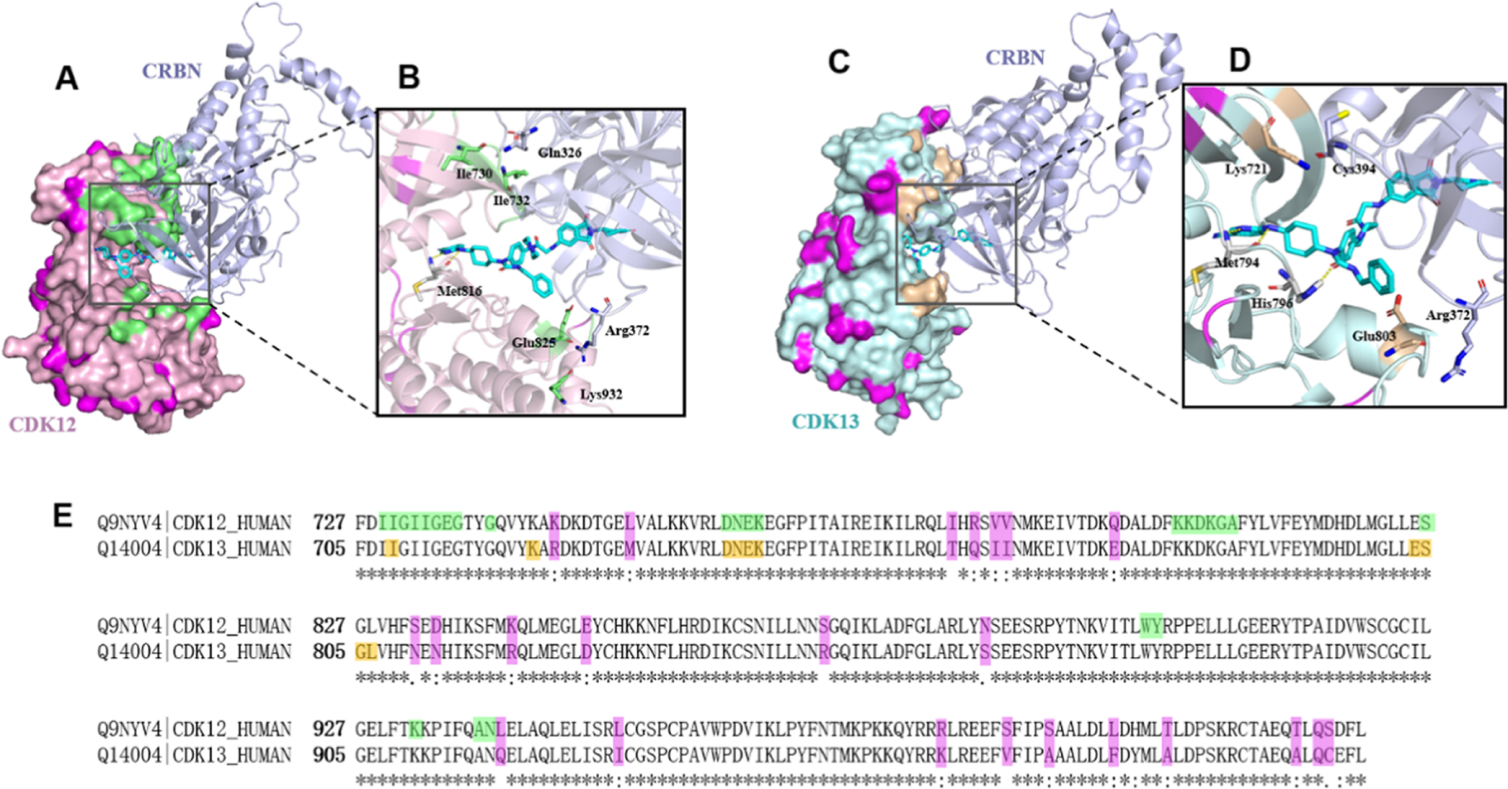
Docking models of the top-ranked ternary complexes. (A) Ternary complex of compound **7f** (cyan), CDK12 (lightpink, PDB: 6CKX), and CRBN (lightblue, PDB: 4TZ4). Nonconserved residues in the kinase domains of CDK12 and CDK13 are highlighted in purple. PPI interfaces between CDK12 and CRBN are highlighted as green. (B, D) Magnified view of **7f** (cyan) binding to the grooves between CDK12 (light pink) and CRBN (light blue), CDK13 (pale green), and CRBN (light blue), respectively. (C) Ternary complex of compound **7f** (cyan), CDK13 (pale green, PDB: 5EFQ), and CRBN (light blue, PDB: 4TZ4). (E) Amino acid sequence alignment in kinase domains of CDK12 and CDK13. Nonconserved residues are highlighted as purple. PPI interface residues between CDK12 and CRBN, CDK13 and CRBN are highlighted as green and yellow, respectively.

To rationalize the dual CDK12/13 degradation effects of compound **7f**, we carried out a computational study to model the ternary complexes of CDK12/13 with CRBN. Protein–protein docking and 500 ns molecular dynamics (MD) simulations were performed to predict the possible structures of the ternary complex. The representative structures from MD simulations are shown in Figure 6A,C. Both complexes (CDK12/**7f**/CRBN and CDK13/**7f**/CRBN) have an extensive interface with good shape complementarity (Figure 6A,C). Compound **7f** fits nicely into the grooves between CDK12/13 and CRBN proteins without major rearrangements (Figure 6B,D). Specifically, the thalidomide group in compound **7f** extends into CRBN through the P-loop of CDK12 and CDK13, where the residues involved in the protein–protein interaction (PPI) between CRBN and CDK12/CDK13 are highly conserved (Figures 6A,C,E and S4). The formations of the efficient ternary complex for both CRBN-**7f**-CDK12 and CRBN-**7f**-CDK13 suggest the dual degradation of CDK12/13 by **7f**. It is worth noting that the interacting interface for CDK12/13 and CRBN mediated by compound **7f** in our model is unique to that mediated by BSJ-4-116.^28^ The most different residues are Lys745 in CDK12 and Arg723 in CDK13 highlighted in the amino acid sequence alignment (Figure 6E). Indeed, Jiang et al. also discovered the residue differences in CDK12/13 that could render the selectivity of BSJ-4-116 on CDK12 over CDK13.^28^

### Preliminary *In Vivo* Pharmacodynamic Investigation of **7b**

**Figure 7:**
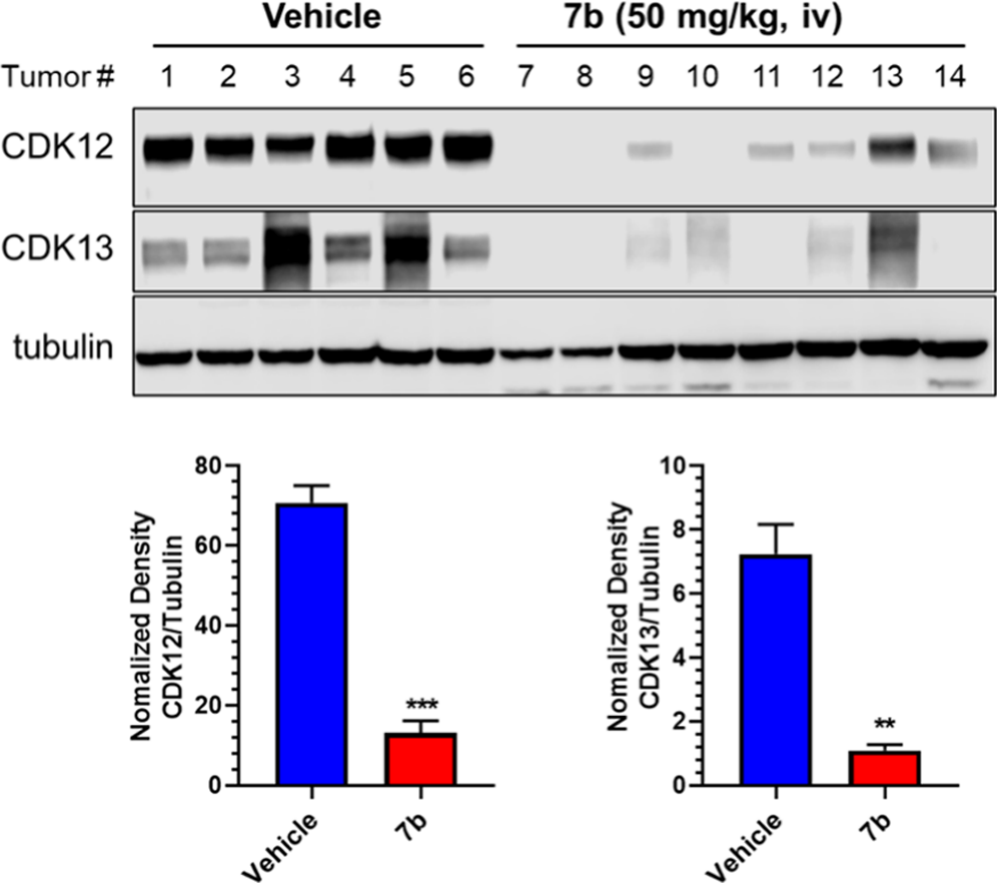
Preliminary Pharmacodynamic study of **7b** in an MDA-MB-436 xenografted mouse model. Data presented are mean ± standard deviation; *p* values from two-way analysis of variance (ANOVA) tests; **p value <0.01, ***p value <0.001.

Due to the undesirable pharmacokinetic properties (PK) and poor solubility of **7f** (Table S1), we therefore selected compound **7b**, an analogue of **7f** with similar CDK12/13 dual degrading potencies (Table 3), and improved solubility (data not shown), for the pharmacodynamic study in an MDA-MB-436 xenografted mouse model. The drug was administered by i.v. injection with a dose of 50 mg/kg. The tumor tissues were harvested after injection for 6 h and subjected to Western blot analysis. As shown in Figure 7, compound **7b** significantly degraded both CDK12 and CDK13 compared to the vehicle control group. An additional PD in the MDA-MB-231 xenografted mouse model also demonstrated that compound **7b** markedly degraded both CDK12 and CDK13 after treatment for 6 h (Figure S5), indicating its strong and fast degradation efficiency *in vivo*.

## Conclusions

In summary, we have designed the first series of dual CDK12/13 degraders, which were synthesized by conjugating the optimized CDK12/13 inhibitor **4** with CRBN ligands pomalidomide/lenalidomide. The most potent degrader **7f** bearing thalidomide effectively depleted CDK12 and CDK13 proteins with DC_50_ values of 2.2 and 2.1 nM, respectively, in MDA-MB-231 cells and displayed extraordinary selectivity among 6319 proteins identified in the global proteomic profiling study. Mechanistic studies demonstrated that the degradation of CDK12/13 by compound **7f** was indeed through the ubiquitin–proteasome system. Compound **7f**-mediated depletion of CDK12/13 also inhibited the expression of DDR genes. *In vitro*, degrader **7f** potently inhibited cell growth of HR-deficient MFM223 and MDA-MB-436 TNBC cells with IC_50_ values of 47 and 197.9 nM, respectively, which significantly outperformed the structurally similar kinase inhibitor **4**. More importantly, a combination of compound **7f** with a DDR inhibitor demonstrates a significant synergistic effect in suppressing the growth of HR-proficient MDA-MB-231 cells. The *in vivo* PD study in the MDA-MB-436 xenografted mouse model demonstrated an efficient degradation of CDK12/13. Taken together, compound **7f** disclosed in this study represents the first potent and highly selective CDK12/13 degrader, which could serve as a valuable chemical probe for further evaluation of its therapeutic potential to target CDK12/13 in TNBC.

## Experimental Section

### General Methods for Chemistry

All commercially available reagents and solvents were used without further purification. All chemical reactions were monitored by thin-layer chromatography (TLC) plates with visualization under UV light (254 or 365 nm). ^1^H NMR spectra were performed with a Bruker AV-400/600 spectrometer, ^13^C NMR spectra were recorded on a Bruker AV-600 spectrometer at 150 MHz, and internal reference was either TMS or a deuterated NMR solvent. Low-resolution mass spectra (MS) were recorded on an Agilent 1200 HPLC-MSD mass spectrometer. High-resolution mass spectral analysis was recorded on an Applied Biosystems Q-STAR Elite ESI-LC-MS/MS mass spectrometer. The purity of all final compounds was confirmed to be >95% by HPLC analysis with the Agilent 1260 system. The analytical columns were the YMC-Triart C18 reversed-phase column, 5 μm, 4.6 mm × 250 mm, and a flow rate of 1.0 mL/min.

#### *tert*-Butyl 4-(4-(((1r,4r)-4-Aminocyclohexyl)amino)phenyl)piperazine-1-carboxylate (**10**)

Potassium phosphate (31.0 g, 146 mmol) was added to a solution of *trans*-cyclohexane-1,4-diamine (29.3 g, 256.4 mmol), *tert*-butyl 4-(4-bromophenyl)piperazine-1-carboxylate **9** (25.0 g, 73.26 mmol), CuI (1.4 g, 7.3 mmol), and d-Proline (843 mg, 7.3 mmol) in anhydrous DMSO (500 mL). The resulted suspension was then evacuated and backfilled with argon (3 cycles). The reaction mixture was then heated at 100 °C for 10 h before being filtered through celite. The reaction solvent was evaporated under reduced pressure and purified by silica gel column chromatography to afford the title compound as a gray solid. (12.0 g, yield 44%): ^1^H NMR (400 MHz, DMSO-*d*_6_) δ 6.74 (d, *J* = 8.8 Hz, 2H), 6.49 (d, *J* = 8.9 Hz, 2H), 4.88 (d, *J* = 8.2 Hz, 1H), 3.42 (t, *J* = 5.1 Hz, 4H), 3.03 (s, 1H), 2.83 (t, *J* = 5.1 Hz, 4H), 2.76 (s, 1H), 1.95 (d, *J* = 12.8 Hz, 2H), 1.85 (d, *J* = 12.4 Hz, 2H), 1.41 (s, 9H), 1.26 (q, *J* = 10.9 Hz, 2H), 1.11 (q, *J* = 11.6 Hz, 2H). HRMS (ESI) for C_21_H_34_N_4_O_2_ [M + H]^+^, calcd: 375.2755, found: 375.2739.

#### *tert*-Butyl 4-(4-(((1r,4r)-4-((5-Cyanopyridin-2-yl)amino)cyclohexyl)amino)phenyl)piperazine-1-carboxylate (**11**)

To a solution of *tert*-butyl 4-(4-(((1r,4r)-4-aminocyclohexyl)amino)phenyl)piperazine-1-carboxylate **10** (9.0 g, 24 mmol) in DMF (40 mL) were added 5-cyano-2-fluoropyridine (2.94 g, 24 mmol) and Cs_2_CO_3_ (9.4 g, 28.9 mmol). The mixture was stirred at room temperature overnight. The reaction mixture was then filtered, and the solvent was removed under reduced pressure. The crude material was purified by column chromatography to afford **11** as a white solid (10.6 g, yield 92%). ^1^H NMR (400 MHz, DMSO-*d*_6_) δ 8.37 (d, *J* = 2.3 Hz, 1H), 7.63 (dd, *J* = 8.7, 2.2 Hz, 1H), 7.52 (d, *J* = 7.5 Hz, 1H), 6.75 (d, *J* = 8.6 Hz, 2H), 6.52 (dd, *J* = 9.0, 3.4 Hz, 3H), 4.92 (d, *J* = 8.3 Hz, 1H), 3.76 (s, 1H), 3.42 (t, *J* = 5.2 Hz, 4H), 3.12 (d, *J* = 8.3 Hz, 1H), 2.84 (t, *J* = 5.1 Hz, 4H), 1.98 (t, *J* = 11.7 Hz, 4H), 1.41 (s, 9H), 1.32 (t, *J* = 12.2 Hz, 2H), 1.22 (t, *J* = 13.3 Hz, 2H). HRMS (ESI) for C_27_H_36_N_6_O_2_ [M + H]^+^, calcd: 477.2973, found: 477.2949.

#### *tert*-Butyl 4-(4-(3-Benzyl-1-((1r,4r)-4-((5-cyanopyridin-2-yl)amino)cyclohexyl)ureido)phenyl)piperazine-1-carboxylate (**12**)

To a solution of *tert*-butyl 4-(4-(((1r,4r)-4-((5-cyanopyridin-2-yl)amino)cyclohexyl)amino)phenyl)piperazine-1-carboxylate **11** (10.6 g, 22.25 mmol) and DIPEA (8.6 g, 66.76 mmol) in DMF (20 mL) was added benzyl isocyanate (8.87 g, 66.67 mmol) at room temperature. The mixture was stirred at 95 °C for 4 h. The solvent was removed under reduced pressure and purified by column chromatography to give a white solid (7.1 g, yield 52%). ^1^H NMR (400 MHz, DMSO-*d*_6_) δ 8.29 (d, *J* = 2.3 Hz, 1H), 7.60 (dd, *J* = 8.9, 2.3 Hz, 1H), 7.47 (d, *J* = 7.6 Hz, 1H), 7.27 (t, *J* = 7.4 Hz, 2H), 7.17 (t, *J* = 8.4 Hz, 3H), 7.06–6.96 (m, 4H), 6.47 (d, *J* = 8.9 Hz, 1H), 5.58 (t, *J* = 6.1 Hz, 1H), 4.31–4.21 (m, 1H), 4.15 (d, *J* = 6.0 Hz, 2H), 3.46 (t, *J* = 5.2 Hz, 5H), 3.16 (t, *J* = 5.2 Hz, 4H), 1.90 (d, *J* = 11.7 Hz, 2H), 1.77 (d, *J* = 12.1 Hz, 2H), 1.42 (s, 9H), 1.31 (q, *J* = 12.1, 11.6 Hz, 2H), 1.09 (q, *J* = 12.2, 11.6 Hz, 2H). HRMS (ESI) for C_35_H_43_N_7_O_3_ [M + H]^+^, calcd: 610.3500, found: 610.3505.

#### 3-Benzyl-1-((1r,4r)-4-((5-cyanopyridin-2-yl)amino)cyclohexyl)-1-(4-(piperazin-1-yl)phenyl)urea (**4**)

TFA (10 mL) was added to a solution of *tert*-butyl 4-(4-(3-benzyl-1-((1r,4r)-4-((5-cyanopyridin-2-yl)amino)cyclohexyl)ureido)phenyl)piperazine-1-carboxylate **12** (7.0 g, 11.5 mmol) in DCM (20 mL), and the mixture was stirred at 50 °C for overnight. The reaction mixture was then concentrated to dryness under reduced pressure, and the resultant crude material was purified by column chromatography to afford the title compound as a white solid (4.6 g, yield 79%). ^1^H NMR (600 MHz, DMSO-*d*_6_) δ 8.30 (d, *J* = 2.3 Hz, 1H), 7.60 (d, *J* = 6.7 Hz, 1H), 7.53 (s, 1H), 7.27 (t, *J* = 7.5 Hz, 2H), 7.17 (dd, *J* = 12.7, 7.3 Hz, 3H), 7.03–6.93 (m, 4H), 6.49 (d, *J* = 8.9 Hz, 1H), 5.58 (t, *J* = 6.2 Hz, 1H), 4.26 (tt, *J* = 12.0, 3.0 Hz, 1H), 4.15 (d, *J* = 6.0 Hz, 2H), 3.51 (s, 1H), 3.10 (t, *J* = 5.0 Hz, 4H), 2.83 (t, *J* = 5.0 Hz, 4H), 1.91 (d, *J* = 12.2 Hz, 2H), 1.77 (d, *J* = 10.5 Hz, 2H), 1.31 (q, *J* = 13.8, 12.9 Hz, 2H), 1.24 (s, 1H), 1.10 (q, *J* = 11.4 Hz, 2H). ^13^C NMR (151 MHz, DMSO-*d*_6_) δ 159.71, 157.27, 153.55, 151.202, 141.80, 131.92, 128.47 (5 C), 127.14 (4 C), 126.68, 119.59, 115.74, 94.41, 53.36, 49.16 (2 C), 46.07 (2 C), 43.93, 40.52, 31.77 (2 C), 30.66 (2 C). HRMS (ESI) for C_30_H_35_N_7_O [M + H]^+^, calcd: 510.2976, found: 510.2956.

#### 2-(4-(4-(3-Benzyl-1-((1r,4r)-4-((5-cyanopyridin-2-yl)amino)cyclohexyl)ureido)phenyl)piperazin-1-yl)-*N*-(2-(2,6-dioxopiperidin-3-yl)-1,3-dioxoisoindolin-4-yl)acetamide (**5a**)

To a solution of **4** (62 mg, 0.12 mmol) in DMF (8 mL) were added **13a** (94 mg, 0.24 mmol) and KHCO_3_ (36 mg, 0.36 mmol). After stirring at 80 °C for 5 h, the reaction mixture was then filtered and the solvent was removed under reduced pressure. The crude material was purified by column chromatography to afford **5a** as a white solid (31 mg, yield 31%). ^1^H NMR (600 MHz, DMSO-*d*_6_) δ 11.12 (s, 1H), 11.03 (s, 1H), 8.82 (d, *J* = 8.5 Hz, 1H), 8.30 (d, *J* = 2.3 Hz, 1H), 7.87 (dd, *J* = 8.5, 7.3 Hz, 1H), 7.61 (d, *J* = 7.3 Hz, 2H), 7.48 (s, 1H), 7.27 (t, *J* = 7.6 Hz, 2H), 7.17 (t, *J* = 5.6 Hz, 3H), 7.02 (s, 4H), 6.47 (d, *J* = 8.9 Hz, 1H), 5.61 (t, *J* = 6.2 Hz, 1H), 5.12 (dd, *J* = 12.9, 5.4 Hz, 1H), 4.27 (t, *J* = 12, 3.6 Hz, 1H), 4.16 (d, *J* = 6.0 Hz, 2H), 3.50 (s, 1H), 3.38–3.35 (m, 4H), 3.32 (d, *J* = 20.7 Hz, 2H), 2.90–2.81 (m, 1H), 2.74 (h, *J* = 6.7 Hz, 4H), 2.60–2.52 (m, 2H), 2.06 (m, 1H), 1.92 (d, *J* = 11.9 Hz, 2H), 1.78 (d, *J* = 11.9 Hz, 2H), 1.32 (q, *J* = 12.1, 11.3 Hz, 2H), 1.11 (q, *J* = 12.5 Hz, 2H). ^13^C NMR (151 MHz, DMSO-*d*_6_) δ 173.19, 170.46, 170.30, 168.55, 167.28, 159.69, 157.32, 153.54, 150.34, 141.73, 136.92, 136.81, 132.04, 131.78, 128.68, 128.50 (4 C), 127.13 (4 C), 126.72, 124.71, 119.57, 118.46, 116.24, 115.90, 94.46, 61.76, 60.25, 53.41 (2 C), 49.35, 49.02, 47.96 (2 C), 43.92, 40.42, 31.76 (2 C), 31.30, 30.67, 22.33, 21.23. HRMS (ESI) for C_45_H_46_N_10_O_6_[M + H]^+^, calcd: 823.3675, found: 823.3652. HPLC analysis: MeOH-H_2_O (75:25), 11.77 min, 100% purity.

#### 3-(4-(4-(3-Benzyl-1-((1r,4r)-4-((5-cyanopyridin-2-yl)amino)cyclohexyl)ureido)phenyl)piperazin-1-yl)-*N*-(2-(2,6-dioxopiperidin-3-yl)-1,3-dioxoisoindolin-4-yl)propanamide (**5b**)

Compound **5b** was synthesized by following a similar procedure as that of **5a**. ^1^H NMR (400 MHz, DMSO-*d*_6_) δ 11.11 (s, 1H), 10.47 (s, 1H), 8.56 (d, *J* = 8.4 Hz, 1H), 8.29 (d, *J* = 2.3 Hz, 1H), 7.83 (t, *J* = 7.9 Hz, 1H), 7.65–7.57 (m, 2H), 7.53 (s, 1H), 7.27 (t, *J* = 7.5 Hz, 2H), 7.17 (t, *J* = 7.4 Hz, 3H), 7.08–6.90 (m, 4H), 6.49 (d, *J* = 8.9 Hz, 1H), 5.60 (t, *J* = 6.1 Hz, 1H), 5.11 (dd, *J* = 12.5, 5.5 Hz, 1H), 4.26 (tt, *J* = 17.4, 4.8 Hz, 2H), 4.15 (d, *J* = 6.3 Hz, 2H), 3.51 (s, 1H), 3.25 (q, *J* = 5.2 Hz, 4H), 3.17 (d, *J* = 5.1 Hz, 2H), 2.91–2.79 (m, 1H), 2.76–2.66 (m, 4H), 2.66–2.57 (m, 4H), 2.10–2.00 (m, 1H), 1.91 (d, *J* = 11.8 Hz, 2H), 1.77 (d, *J* = 11.6 Hz, 2H), 1.38–1.25 (m, 2H), 1.18–1.02 (m, 2H). ^13^C NMR (151 MHz, DMSO-*d*_6_) δ 173.19, 171.88, 170.26, 167.83, 167.12, 159.72, 157.26, 153.56, 150.67, 141.79, 136.92, 136.52, 132.03, 131.94, 128.63, 128.48 (4 C), 127.15 (4 C), 126.91, 126.68, 119.61, 118.72, 117.28, 115.87, 94.38, 53.77, 53.41, 53.00 (2 C), 49.32, 49.04, 47.68 (2 C), 43.93, 40.52, 34.36, 31.76 (2 C), 31.27, 30.65, 22.45. HRMS (ESI) for C_46_H_48_N_10_O_6_ [M + H]^+^, calcd: 837.3831, found: 837.3802. HPLC analysis: MeOH-H_2_O (80:20), 7.34 min, 97.7% purity.

#### 4-(4-(4-(3-Benzyl-1-((1r,4r)-4-((5-cyanopyridin-2-yl)amino)cyclohexyl)ureido)phenyl)piperazin-1-yl)-*N*-(2-(2,6-dioxopiperidin-3-yl)-1,3-dioxoisoindolin-4-yl)butanamide (**5c**)

Compound **5c** was synthesized by following a similar procedure as that of **5a**. ^1^H NMR (600 MHz, DMSO-*d*_6_) δ 11.17 (s, 1H), 9.71 (s, 1H), 8.50 (d, *J* = 8.4 Hz, 1H), 8.29 (d, *J* = 2.3 Hz, 1H), 7.84–7.80 (m, 1H), 7.59 (dd, *J* = 11.4, 7.6 Hz, 2H), 7.49 (s, 1H), 7.27 (t, *J* = 7.5 Hz, 2H), 7.17 (dd, *J* = 13.1, 7.3 Hz, 3H), 7.02–6.91 (m, 4H), 6.47 (d, *J* = 8.9 Hz, 1H), 5.56 (t, *J* = 6.2 Hz, 1H), 5.15 (dd, *J* = 12.9, 5.5 Hz, 1H), 4.26 (tt, *J* = 12, 3.0 Hz, 1H), 4.15 (d, *J* = 6.1 Hz, 2H), 3.51 (s, 1H), 3.14 (t, *J* = 5.0 Hz, 4H), 2.94–2.84 (m, 1H), 2.64–2.54 (m, 2H), 2.52 (s, 2H), 2.52–2.50 (m,4H), 2.41 (t, *J* = 7.1 Hz, 2H), 2.10–2.04(m, 1H), 1.91 (d, *J* = 11.8 Hz, 2H), 1.84 (p, *J* = 7.1 Hz, 2H), 1.77 (d, *J* = 11.5 Hz, 2H), 1.31 (q, *J* = 13.0 Hz, 2H), 1.09 (q, *J* = 14.0, 13.3 Hz, 2H). ^13^C NMR (151 MHz, DMSO-*d*_6_) δ 173.22, 172.53, 170.25, 168.24, 167.12, 159.70, 157.27, 153.55, 150.61, 141.76, 137.13, 136.59, 131.92, 131.86, 128.58, 128.49 (4 C), 127.14 (4 C), 126.70, 126.61, 119.58, 118.66, 117.24, 115.77, 94.44, 57.36, 53.38, 53.11 (2 C), 49.37, 49.08, 48.05 (2 C), 43.93, 40.49, 35.18, 31.77 (2 C), 31.39, 30.65, 22.48, 22.43. HRMS (ESI) for C_47_H_50_N_10_O_6_ [M + H]^+^, calcd: 851.3988, found: 851.3970. HPLC analysis: MeOH-H_2_O (75:25), 8.58 min, 100% purity.

#### 5-(4-(4-(3-Benzyl-1-((1r,4r)-4-((5-cyanopyridin-2-yl)amino)cyclohexyl)ureido)phenyl)piperazin-1-yl)-*N*-(2-(2,6-dioxopiperidin-3-yl)-1,3-dioxoisoindolin-4-yl)pentanamide (**5d**)

Compound **5d** was synthesized by following a similar procedure as that of **5a**. ^1^H NMR (600 MHz, DMSO-*d*_6_) δ 11.17 (s, 1H), 9.71 (s, 1H), 8.48 (d, *J* = 8.4 Hz, 1H), 8.29 (d, *J* = 2.3 Hz, 1H), 7.89–7.80 (m, 1H), 7.61 (t, *J* = 7.9 Hz, 2H), 7.48 (s, 1H), 7.27 (t, *J* = 7.5 Hz, 2H), 7.17 (dd, *J* = 12.7, 7.3 Hz, 3H), 7.04–6.93 (m, 4H), 6.47 (d, *J* = 8.9 Hz, 1H), 5.57 (t, *J* = 6.2 Hz, 1H), 5.15 (dd, *J* = 12.9, 5.5 Hz, 1H), 4.26 (tt, *J* = 12.0, 3.6 Hz, 1H), 4.15 (d, *J* = 6.1 Hz, 2H), 3.51(s, 1H), 3.17 (t, *J* = 5.0 Hz, 4H), 2.94–2.84 (m, 1H), 2.64–2.53 (m, 2H), 2.53–2.49 (m, 6H), 2.36 (t, *J* = 7.2 Hz, 2H), 2.10–2.04 (m 1H), 1.91 (d, *J* = 9.9 Hz, 2H), 1.77 (d, *J* = 10.8 Hz, 2H), 1.67 (p, *J* = 7.4 Hz, 2H), 1.54 (p, *J* = 7.3 Hz, 2H), 1.31 (q, *J* = 13.5, 12.4 Hz, 2H), 1.10 (q, *J* = 12.5, 12.0 Hz, 2H). ^13^C NMR (151 MHz, DMSO-*d*_6_) δ 173.25, 172.51, 170.25, 168.15, 167.13, 159.69, 157.30, 153.54, 150.62, 141.73, 136.99, 136.60, 131.93, 128.54, 128.48 (4 C), 127.14 (4 C), 126.78, 126.71, 119.58, 118.80, 117.45, 115.78, 94.43, 7.23, 57.85, 56.51, 53.37, 53.23 (2 C), 49.36, 49.06, 48.06 (2 C), 43.92, 40.43, 36.82, 31.75 (2 C), 31.37, 30.65, 26.06, 23.24, 22.46. HRMS (ESI) for C_48_H_52_N_10_O_6_ [M + H]^+^, calcd: 865.4144, found: 865.4133. HPLC analysis: MeOH-H_2_O (75:25), 10.69 min, 97.9% purity.

#### 6-(4-(4-(3-Benzyl-1-((1r,4r)-4-((5-cyanopyridin-2-yl)amino)cyclohexyl)ureido)phenyl)piperazin-1-yl)-*N*-(2-(2,6-dioxopiperidin-3-yl)-1,3-dioxoisoindolin-4-yl)hexanamide (**5e**)

Compound **5e** was synthesized by following a similar procedure as that of **5a**. ^1^H NMR (600 MHz, DMSO-*d*_6_) δ 11.17 (s, 1H), 9.71 (s, 1H), 8.48 (d, *J* = 8.4 Hz, 1H), 8.29 (d, *J* = 2.3 Hz, 1H), 7.83 (t, *J* = 7.9 Hz, 1H), 7.64–7.56 (m, 2H), 7.48 (s, 1H), 7.27 (t, *J* = 7.5 Hz, 2H), 7.17 (dd, *J* = 13.2, 7.3 Hz, 3H), 7.02–6.93 (m, 4H), 6.47 (d, *J* = 8.9 Hz, 1H), 5.57 (t, *J* = 6.1 Hz, 1H), 5.15 (dd, *J* = 12.9, 5.5 Hz, 1H), 4.26 (tt, *J* = 12.0, 3.6 Hz, 1H), 4.15 (d, *J* = 6.0 Hz, 2H), 3.51 (s, 1H), 3.16 (t, *J* = 4.8 Hz, 4H), 2.94–2.84 (m, 1H), 2.65–2.52 (m, 2H), 2.50–2.45 (m, 6H), 2.32 (t, *J* = 7.4 Hz, 2H), 2.11–2.04 (m, 1H), 1.91 (d, *J* = 8.7 Hz, 2H), 1.77 (d, *J* = 10.8 Hz, 2H), 1.67 (p, *J* = 7.5 Hz, 2H), 1.51 (p, *J* = 7.5 Hz, 2H), 1.37 (p, *J* = 7.9 Hz, 2H), 1.30(q, *J* = 12.4 Hz, 2H), 1.10 (q, *J* = 12.4 Hz, 2H). ^13^C NMR (151 MHz, DMSO-*d*_6_) δ 173.24, 172.51, 170.25, 168.16, 167.13, 159.69, 157.29, 153.55, 150.63, 141.75, 137.00, 136.58, 131.93, 131.90, 128.55, 128.48 (4 C), 127.14 (4 C), 126.74, 126.70, 119.58, 118.78, 117.42, 115.77, 94.44, 58.15, 53.37, 53.28 (2 C), 49.3, 49.07, 48.09 (2 C), 43.92, 40.47, 36.96, 31.76 (2 C), 31.39, 30.65, 26.90, 26.41, 25.20, 22.46. HRMS (ESI) for C_49_H_54_N_10_O_6_ [M + H]^+^, calcd: 879.4301, found: 879.4284. HPLC analysis: MeOH-H_2_O (75:25), 13.27 min, 98.8% purity.

#### 7-(4-(4-(3-Benzyl-1-((1r,4r)-4-((5-cyanopyridin-2-yl)amino)cyclohexyl)ureido)phenyl)piperazin-1-yl)-*N*-(2-(2,6-dioxopiperidin-3-yl)-1,3-dioxoisoindolin-4-yl)heptanamide (**5f**)

Compound **5f** was synthesized by following a similar procedure as that of **5a**. ^1^H NMR (600 MHz, DMSO-*d*_6_) δ 11.17 (s, 1H), 9.71 (s, 1H), 8.48 (d, *J* = 8.4 Hz, 1H), 8.29 (d, *J* = 2.3 Hz, 1H), 7.83 (t, *J* = 7.9 Hz, 1H), 7.65–7.56 (m, 2H), 7.47 (s, 1H), 7.27 (t, *J* = 7.5 Hz, 2H), 7.17 (dd, *J* = 13.2, 7.3 Hz, 3H), 7.03–6.92 (m, 4H), 6.47 (d, *J* = 8.9 Hz, 1H), 5.58 (t, *J* = 6.2 Hz, 1H), 5.15 (dd, *J* = 12.9, 5.5 Hz, 1H), 4.26 (tt, *J* = 12.0, 3.6 Hz, 1H), 4.15 (d, *J* = 6.1 Hz, 2H), 3.51 (s, 1H), 3.16 (t, *J* = 4.8 Hz, 4H), 2.94–2.84 (m, 1H), 2.64–2.52 (m, 2H), 2.50–2.43 (m, 6H), 2.31 (t, *J* = 7.5 Hz, 2H), 2.10–2.04 (m, 1H), 1.90 (d, *J* = 10.0 Hz, 2H), 1.76 (d, *J* = 10.5 Hz, 2H), 1.64 (p, *J* = 7.3 Hz, 2H), 1.47 (p, *J* = 7.1 Hz, 2H), 1.41–1.26 (m, 6H), 1.10 (q, *J* = 12 Hz, 2H, 2H). ^13^C NMR (151 MHz, DMSO-*d*_6_) δ 173.25, 172.54, 170.25, 168.14, 167.13, 159.69, 157.31, 153.54, 150.61, 141.73, 136.99, 136.59, 131.93, 131.89, 128.53, 128.49 (4 C), 127.14 (4 C), 126.77, 126.71, 119.58, 118.80, 117.45, 115.78, 94.44, 58.28, 53.37, 53.28 (2 C), 49.37, 49.04, 48.05 (2 C), 43.92, 40.43, 36.97, 31.75 (2 C), 31.38, 30.65, 28.88, 27.15, 26.53, 25.23, 22.45. HRMS (ESI) for C_50_H_56_N_10_O_6_ [M + H]^+^, calcd: 893.4457, found: 893.4446. HPLC analysis: MeOH-H_2_O (75:25), 17.68 min, 99.6% purity.

#### 8-(4-(4-(3-Benzyl-1-((1r,4r)-4-((5-cyanopyridin-2-yl)amino)cyclohexyl)ureido)phenyl)piperazin-1-yl)-*N*-(2-(2,6-dioxopiperidin-3-yl)-1,3-dioxoisoindolin-4-yl)octanamide (**5g**)

Compound **5g** was synthesized by following a similar procedure as that of **5a**. ^1^H NMR (600 MHz, DMSO-*d*_6_) δ 11.18 (s, 1H), 9.71 (s, 1H), 8.47 (d, *J* = 8.4 Hz, 1H), 8.30 (d, *J* = 2.3 Hz, 1H), 7.86–7.81 (m, 1H), 7.61 (t, *J* = 7.2 Hz, 2H), 7.48 (s, 1H), 7.27 (t, *J* = 7.6 Hz, 2H), 7.17 (dd, *J* = 14.6, 7.2 Hz, 3H), 7.04–6.92 (m, 4H), 6.47 (d, *J* = 8.9 Hz, 1H), 5.58 (t, *J* = 6.1 Hz, 1H), 5.15 (dd, *J* = 12.9, 5.5 Hz, 1H), 4.26 (tt, *J* = 12, 3.6 Hz, 1H), 4.15 (d, *J* = 6.0 Hz, 2H), 3.51 (s, 1H), 3.17 (t, *J* = 4.9 Hz, 4H), 2.93–2.84 (m, 1H), 2.64–2.52 (m, 2H), 2.49–2.45 (m, 6H), 2.30 (t, *J* = 7.4 Hz, 2H), 2.10–2.04 (m, 1H), 1.90 (d, *J* = 8.5 Hz, 2H), 1.76 (d, *J* = 10.6 Hz, 2H), 1.63 (p, *J* = 7.2 Hz, 2H), 1.46 (p, *J* = 7.1 Hz, 2H), 1.38–1.20 (m, 8H), 1.10 (q, *J* = 12.9 Hz, 2H).^13^C NMR (151 MHz, DMSO-*d*_6_) δ 173.24, 172.53, 170.25, 168.15, 167.13, 159.70, 157.28, 153.56, 150.63, 141.77, 136.99, 136.58, 131.94, 131.91, 128.56, 128.48 (4 C), 127.14 (4 C), 126.77, 126.70, 119.59, 118.79, 117.45, 115.78, 94.44, 58.34, 53.36, 53.31 (2 C), 49.37, 49.06, 48.09 (2 C), 43.92, 40.48, 36.95, 31.76 (2 C), 31.39, 30.65, 29.08, 28.90, 27.28, 26.65, 25.20, 22.46. HRMS (ESI) for C_51_H_58_N_10_O_6_ [M + H]^+^, calcd: 907.4614, found: 907.4589. HPLC analysis: MeOH-H_2_O (80:20), 9.31 min, 98.8% purity.

#### 9-(4-(4-(3-Benzyl-1-((1r,4r)-4-((5-cyanopyridin-2-yl)amino)cyclohexyl)ureido)phenyl)piperazin-1-yl)-*N*-(2-(2,6-dioxopiperidin-3-yl)-1,3-dioxoisoindolin-4-yl)nonanamide (**5h**)

Compound **5h** was synthesized by following a similar procedure as that of **5a**. ^1^H NMR (600 MHz, DMSO-*d*_6_) δ 11.17 (s, 1H), 9.70 (s, 1H), 8.48 (d, *J* = 8.4 Hz, 1H), 8.29 (d, *J* = 2.3 Hz, 1H), 7.83 (t, *J* = 7.9 Hz, 1H), 7.61 (dd, *J* = 8.5, 5.5 Hz, 2H), 7.48 (s, 1H), 7.27 (t, *J* = 7.5 Hz, 2H), 7.17 (dd, *J* = 13.1, 7.3 Hz, 3H), 7.02–6.93 (m, 4H), 6.47 (d, *J* = 8.9 Hz, 1H), 5.58 (t, *J* = 6.2 Hz, 1H), 5.15 (dd, *J* = 12.9, 5.5 Hz, 1H), 4.26 (tt, *J* = 12, 3.6 Hz, 1H), 4.15 (d, *J* = 6.0 Hz, 2H), 3.51 (s, 1H), 3.17 (t, *J* = 4.9 Hz, 4H), 2.93–2.84 (m, 1H), 2.64–2.52 (m, 2H), 2.49–2.44 (m, 6H), 2.29 (t, *J* = 7.5 Hz, 2H), 2.10–2.03 (m, 1H), 1.91 (d, *J* = 11.9 Hz, 2H), 1.76 (d, *J* = 12.3 Hz, 2H), 1.63 (p, *J* = 7.1 Hz, 2H), 1.45 (p, *J* = 7.2 Hz, 2H), 1.36–1.27 (m, 10H), 1.10 (q, *J* = 11.8, 2H). ^13^C NMR (151 MHz, DMSO-*d*_6_) δ 173.22, 172.50, 170.24, 168.14, 167.12, 159.70, 157.26, 153.55, 150.62, 141.80, 137.01, 136.57, 131.94, 131.92, 128.58, 128.47 (4 C), 127.14 (4 C), 126.74, 126.68, 119.58, 118.76, 117.44, 115.77, 94.43, 58.39, 53.36, 53.32 (2 C), 49.37, 49.07, 48.11 (2 C), 43.93, 40.52, 36.99, 31.77 (2 C), 31.40, 30.66, 29.30, 29.16, 28.93, 27.41, 26.73, 25.24, 22.46. HRMS (ESI) for C_52_H_60_N_10_O_6_ [M + H]^+^, calcd: 921.4770, found: 921.4754. HPLC analysis: MeOH-H_2_O (75:25), 20.02 min, 99.0% purity.

#### 10-(4-(4-(3-Benzyl-1-((1r,4r)-4-((5-cyanopyridin-2-yl)amino)cyclohexyl)ureido)phenyl)piperazin-1-yl)-*N*-(2-(2,6-dioxopiperidin-3-yl)-1,3-dioxoisoindolin-4-yl)decanamide (**5i**)

Compound **5i** was synthesized by following a similar procedure as that of **5a**. ^1^H NMR (600 MHz, DMSO-*d*_6_) δ 11.18 (s, 1H), 9.70 (s, 1H), 8.47 (d, *J* = 8.4 Hz, 1H), 8.29 (d, *J* = 2.3 Hz, 1H), 7.83 (dd, *J* = 8.4, 7.3 Hz, 1H), 7.61 (t, *J* = 6.9 Hz, 2H), 7.48 (s, 1H), 7.27 (t, *J* = 7.6 Hz, 2H), 7.16 (dd, *J* = 7.9, 6.5 Hz, 3H), 6.98 (q, *J* = 9.0 Hz, 4H), 6.47 (d, *J* = 8.9 Hz, 1H), 5.57 (t, *J* = 6.2 Hz, 1H), 5.15 (dd, *J* = 12.9, 5.5 Hz, 1H), 4.26 (tt, *J* = 12, 3.6 Hz, 1H), 4.14 (d, *J* = 6.1 Hz, 2H), 3.51 (s, 1H), 3.17 (t, *J* = 5.0 Hz, 4H), 2.93–2.84 (m, 1H), 2.65–2.52 (m, 2H), 2.49–2.44 (m, 6H), 2.29 (t, *J* = 7.5 Hz, 2H), 2.10–2.03 (m, 1H), 1.90 (d, *J* = 11.7 Hz, 2H), 1.76 (d, *J* = 11.9 Hz, 2H), 1.63 (p, *J* = 7.3 Hz, 2H), 1.45 (p, *J* = 7.4 Hz, 2H), 1.34–1.26 (m, 12H), 1.10 (q, *J* = 12.6 Hz, 2H). ^13^C NMR (151 MHz, DMSO-*d*_6_) δ 173.25, 172.54, 170.25, 168.14, 167.13, 159.69, 157.30, 153.55, 150.63, 141.74, 136.98, 136.58, 131.94, 131.90, 128.54, 128.49 (4 C), 127.14 (4 C), 126.77, 126.70, 119.58, 118.79, 117.45, 115.78, 94.44, 58.38, 53.36, 53.29 (2 C), 49.36, 49.07, 48.07 (2 C), 43.92, 40.45, 36.98, 31.75 (2 C), 31.39, 30.65, 29.37, 29.30, 29.14, 28.91, 27.42, 26.71, 25.24, 22.45. HRMS (ESI) for C_53_H_62_N_10_O_6_ [M + H]^+^, calcd: 935.4927, found: 935.4903. HPLC analysis: MeOH-H_2_O (85:15), 8.70 min, 99.6% purity.

#### 3-Benzyl-1-((1r,4r)-4-((5-cyanopyridin-2-yl)amino)cyclohexyl)-1-(4-(4-(2-(2,6-dioxopiperidin-3-yl)-1,3-dioxoisoindolin-5-yl)piperazin-1-yl)phenyl)urea (**6a**)

To a solution of 2-(2,6-dioxopiperidin-3-yl)-5-fluoroisoindoline-1,3-dione **14** (42.9 mg, 0.16 mmol) in 6 mL of DMSO, DIPEA (25.1 mg, 0.19 mmol) and compound **4** (66 mg, 0.13 mmol) were added at room temperature. The resulting mixture was stirred at 120 °C for 8 h. The solvent was removed under vacuum to afford the crude material, which was purified by flash column chromatography to afford **6a** as a crimson solid (62 mg, yield 63%). ^1^H NMR (600 MHz, DMSO-*d*_6_) δ 11.10 (s, 1H), 8.30 (d, *J* = 2.3 Hz, 1H), 7.72 (d, *J* = 8.5 Hz, 1H), 7.60 (d, *J* = 8.2 Hz, 1H), 7.49 (s, 1H), 7.41 (s, 1H), 7.33 (dd, *J* = 8.6, 2.2 Hz, 1H), 7.27 (t, *J* = 7.5 Hz, 2H), 7.18 (dd, *J* = 10.1, 7.5 Hz, 3H), 7.05 (s, 4H), 6.47 (d, *J* = 8.9 Hz, 1H), 5.60 (t, *J* = 6.0 Hz, 1H), 5.09 (dd, *J* = 12.8, 5.5 Hz, 1H), 4.28 (tt, *J* = 12, 3.6 Hz, 1H), 4.16 (d, *J* = 6.0 Hz, 2H), 3.63 (t, *J* = 4.9 Hz, 4H), 3.51 (s, 1H), 3.40 (t, *J* = 4.9 Hz, 4H), 2.93–2.84 (m, 1H), 2.64–2.52 (m, 2H), 2.08–2.00 (m, 1H), 1.91 (d, *J* = 11.8 Hz, 2H), 1.78 (d, *J* = 11.8 Hz, 2H), 1.31 (q, *J* = 11.9 Hz, 2H), 1.11 (d, *J* = 12.5 Hz, 2H). ^13^C NMR (151 MHz, DMSO-*d*_6_) δ 173.28, 170.55, 168.00, 167.46, 159.70, 157.27, 155.49, 153.56, 150.19, 141.76, 134.32, 132.08, 128.94, 128.49 (4 C), 127.16 (4 C), 126.71, 125.41, 119.59, 119.00, 118.36, 115.99, 108.47, 94.44, 53.39, 49.26, 49.07, 47.67 (2 C), 47.20 (2 C), 43.94, 40.49, 31.76 (2 C), 31.45, 30.67, 22.65. HRMS (ESI) for C_43_H_43_N_9_O_5_ [M + H]^+^, calcd: 766.3460, found: 766.3431. HPLC analysis: MeOH-H_2_O (73:27), 9.53 min, 98.6% purity.

#### 3-Benzyl-1-((1r,4r)-4-((5-cyanopyridin-2-yl)amino)cyclohexyl)-1-(4-(4-(2-(2,6-dioxopiperidin-3-yl)-1,3-dioxoisoindoline-5-carbonyl)piperazin-1-yl)phenyl)urea (**6b**)

A mixture of 2-(2,6-dioxopiperidin-3-yl)-1,3-dioxoisoindoline-5-carboxylic acid (35.9 mg, 0.12), HATU (45.1 mg, 0.12 mmol), DIPEA (21.3 mg, 0.16 mmol), and compound **4** (55 mg, 0.11 mmol) in DMF (6 mL). The mixture was stirred at room temperature for 15 min, evaporated under vacuum, and purified by silica column chromatography to afford the title compound as a white solid (64 mg, yield 75%). ^1^H NMR (600 MHz, DMSO-*d*_6_) δ 11.17 (s, 1H), 8.29 (d, *J* = 2.3 Hz, 1H), 8.02 (d, *J* = 7.6 Hz, 1H), 7.97 (s, 1H), 7.93 (dd, *J* = 7.6, 1.4 Hz, 1H), 7.60 (d, *J* = 8.5 Hz, 1H), 7.48 (s, 1H), 7.27 (t, *J* = 7.6 Hz, 2H), 7.17 (dd, *J* = 13.6, 7.2 Hz, 3H), 7.02 (q, *J* = 9.1 Hz, 4H), 6.46 (d, *J* = 8.9 Hz, 1H), 5.58 (t, *J* = 6.1 Hz, 1H), 5.19 (dd, *J* = 12.9, 5.4 Hz, 1H), 4.26 (tt, *J* = 12, 3.0 Hz, 1H), 4.15 (d, *J* = 6.1 Hz, 2H), 3.81 (s, 2H), 3.51 (s, 1H), 3.48 (s, 2H), 3.34 (s, 2H), 3.19 (s, 2H), 2.93–2.84 (m, 1H), 2.66–2.52 (m, 2H), 2.13–2.04 (m, 1H), 1.90 (d, *J* = 10.6 Hz, 2H), 1.77 (d, *J* = 10.2 Hz, 2H), 1.30 (q, *J* = 11.2 Hz, 2H), 1.09 (q, *J* =12.0 Hz, 2H). ^13^C NMR (151 MHz, DMSO-*d*_6_) δ 173.26, 170.26, 167.54, 167.07, 167.03, 159.67, 157.28, 153.54, 150.30, 142.57, 141.66, 133.77, 132.17, 132.05, 132.02, 129.17, 128.50 (4 C), 127.14 (4 C), 126.73, 124.35, 122.19, 119.58, 116.49, 94.44, 55.33, 53.39, 49.59, 49.05, 48.43, 47.30, 43.92, 42.04, 40.40, 31.73 (2C), 31.37, 30.65, 22.41. HRMS (ESI) for C_44_H_43_N_9_O_6_ [M + H]^+^, calcd: 794.3409, found: 794.3384. HPLC analysis: MeOH-H_2_O (75:25), 4.48 min, 97.7% purity.

#### 4-(4-(3-Benzyl-1-((1r,4r)-4-((5-cyanopyridin-2-yl)amino)cyclohexyl)ureido)phenyl)-*N*-(2-(2,6-dioxopiperidin-3-yl)-1,3-dioxoisoindolin-5-yl)piperazine-1-carboxamide (**6c**)

To a solution of intermediate **4** (60 mg, 0.12 mmol) in CH_3_CN and DMF (2:1, 12 mL) were added phenyl (2-(2,6-dioxopiperidin-3-yl)-1,3-dioxoisoindolin-5-yl)carbamate **15** (55.5 mg, 0.14 mmol), DMAP (14 mg, 0.12 mmol), and *N*,*N*-diisopropylethylamine (18 mg, 0.14 mmol). The mixture was heated at 60 °C for 4 h. Then, the reaction was allowed to cool to room temperature and concentrated under reduced pressure. The residue was purified via silica gel chromatography to give **6c** as a white solid (42 mg, 44% yield). ^1^H NMR (600 MHz, DMSO-*d*_6_) δ 11.12 (s, 1H), 9.36 (s, 1H), 8.30 (d, *J* = 2.4 Hz, 1H), 8.14 (d, *J* = 1.9 Hz, 1H), 7.90 (dd, *J* = 8.3, 1.9 Hz, 1H), 7.81 (d, *J* = 8.3 Hz, 1H), 7.60 (d, *J* = 8.4 Hz, 1H), 7.48 (s, 1H), 7.27 (t, *J* = 7.6 Hz, 2H), 7.20–7.13 (m, 3H), 7.05 (s, 4H), 6.47 (d, *J* = 8.9 Hz, 1H), 5.60 (t, *J* = 5.9 Hz, 1H), 5.11 (dd, *J* = 12.9, 5.5 Hz, 1H), 4.27 (tt, *J* = 12, 3.6 Hz, 1H), 4.16 (d, *J* = 6.0 Hz, 2H), 3.66 (t, *J* = 5.1 Hz, 4H), 3.49 (s, 1H), 3.28 (t, *J* = 5.2 Hz, 4H), 2.93–2.84 (m, 1H), 2.64–2.52 (m, 2H), 2.08–2.02 (m, 1H), 1.91 (d, *J* = 11.6 Hz, 2H), 1.78 (d, *J* = 11.1 Hz, 2H), 1.31 (q, *J* = 12.2, 2H), 1.11 (q, *J* = 12.2 Hz, 2H). ^13^C NMR (151 MHz, DMSO-*d*_6_) δ 173.27, 170.46, 167.70, 167.41, 159.69, 157.27, 154.70, 153.55, 150.40, 147.39, 141.74, 133.08, 132.04, 129.07, 128.49 (4 C), 127.16 (4 C), 126.72, 124.80, 123.79, 123.71, 119.58, 116.32, 113.46, 94.45, 53.39, 49.35, 49.06, 48.16 (2 C), 44.17 (2 C), 43.94, 40.47, 31.76 (2 C), 31.42, 30.67, 22.56. HRMS (ESI) for C_44_H_44_N_10_O_6_ [M + H]^+^, calcd: 809.3518, found: 809.3490. HPLC analysis: MeOH-H_2_O (75:25), 5.39 min, 98.1% purity.

#### 2-(4-(4-(3-Benzyl-1-((1r,4r)-4-((5-cyanopyridin-2-yl)amino)cyclohexyl)ureido)phenyl)piperazin-1-yl)-*N*-(2-(2,6-dioxopiperidin-3-yl)-1,3-dioxoisoindolin-5-yl)acetamide (**6d**)

Compound **6d** was synthesized by following a similar procedure as that of **5a**. ^1^H NMR (600 MHz, DMSO-*d*_6_) δ 11.13 (s, 1H), 10.48 (s, 1H), 8.31 (d, *J* = 1.8 Hz, 1H), 8.29 (d, *J* = 2.3 Hz, 1H), 8.04 (dd, *J* = 8.3, 1.9 Hz, 1H), 7.89 (d, *J* = 8.2 Hz, 1H), 7.60 (d, *J* = 8.3 Hz, 1H), 7.48 (s, 1H), 7.27 (t, *J* = 7.6 Hz, 2H), 7.17 (dd, *J* = 13.2, 7.2 Hz, 3H), 7.05–6.96 (m, 4H), 6.47 (d, *J* = 8.9 Hz, 1H), 5.57 (t, *J* = 6.1 Hz, 1H), 5.13 (dd, *J* = 12.9, 5.5 Hz, 1H), 4.26 (tt, *J* = 12, 3.6 Hz, 1H), 4.15 (d, *J* = 6.1 Hz, 2H), 3.49 (s, 1H), 3.30 (s, 2H), 3.28 (t, *J* = 4.8 Hz, 4H), 2.93–2.84 (m, 1H), 2.70 (t, *J* = 4.8 Hz, 4H), 2.64–2.52 (m, 2H), 2.10–2.03 (m, 1H), 1.91 (d, *J* = 11.5 Hz, 2H), 1.77 (d, *J* = 9.6 Hz, 2H), 1.31 (q, *J* = 11.3, 9.8 Hz, 2H), 1.10 (q, *J* = 12.6 Hz, 2H). ^13^C NMR (151 MHz, DMSO-*d*_6_) δ 173.26, 170.38, 169.88, 167.46, 167.22, 159.69, 157.31, 153.55, 150.62, 144.95, 141.71, 133.15, 131.96, 128.65, 128.50 (4 C), 127.14 (4 C), 126.72, 125.62, 125.07, 124.54, 119.59, 115.96, 113.91, 94.44, 62.04, 53.38, 53.14 (2 C), 49.45, 49.07, 48.01 (2 C), 43.93, 40.43, 31.75 (2 C), 31.40, 30.66, 22.51. HRMS (ESI) for C_45_H_46_N_10_O_6_ [M + H]^+^, calcd: 823.3675, found: 823.3665. HPLC analysis: MeOH-H_2_O (75:25), 6.81 min, 98.3% purity.

#### 3-(4-(4-(3-Benzyl-1-((1r,4r)-4-((5-cyanopyridin-2-yl)amino)cyclohexyl)ureido)phenyl)piperazin-1-yl)-*N*-(2-(2,6-dioxopiperidin-3-yl)-1,3-dioxoisoindolin-5-yl)propanamide (**6e**)

Compound **6e** was synthesized by following a similar procedure as that of **5a**. ^1^H NMR (600 MHz, DMSO-*d*_6_) δ 11.12 (s, 1H), 10.81 (s, 1H), 8.29 (dd, *J* = 10.6, 2.0 Hz, 2H), 7.96–7.85 (m, 2H), 7.61 (d, *J* = 8.9 Hz, 1H), 7.49 (s, 1H), 7.31–7.25 (m, 2H), 7.20–7.14 (m, 3H), 7.08–6.97 (m, 4H), 6.47 (d, *J* = 8.9 Hz, 1H), 5.62–5.56 (m, 1H), 5.13 (dd, *J* = 12.9, 5.4 Hz, 1H), 4.30–4.23 (m, 1H), 4.15 (d, *J* = 6.5 Hz, 2H), 3.49 (s, 1H), 3.20 (t, *J* = 4.9 Hz, 4H), 2.93–2.84 (m, 1H), 2.74 (t, *J* = 7.1 Hz, 2H), 2.63 (t, *J* = 7.2 Hz, 2H), 2.62–2.52 (m, 6H), 2.09–2.03 (m, 1H), 1.91 (s, 2H), 1.77 (d, *J* = 12.2 Hz, 2H), 1.35–1.27 (m, 2H), 1.10 (q, *J* = 11.4 Hz, 2H). ^13^C NMR (151 MHz, DMSO-*d*_6_) δ 173.24, 171.79, 170.39, 167.49, 167.22, 159.70, 157.28, 153.55, 150.57, 145.49, 141.76, 133.26, 131.95, 128.64, 128.48 (4 C), 127.14 (4 C), 126.70, 125.28, 125.17, 124.02, 119.59, 115.87, 113.32, 94.44, 53.89, 53.37, 52.97 (2 C), 49.43, 49.08, 48.26, 48.13, 43.92, 40.47, 34.82, 31.76 (2 C), 31.41, 30.66, 22.52. HRMS (ESI) for C_46_H_48_N_10_O_6_ [M + H]^+^, calcd: 837.3831, found: 837.3818. HPLC analysis: MeOH-H_2_O (75:25), 5.94 min, 98.8% purity.

#### 5-(4-(4-(3-Benzyl-1-((1r,4r)-4-((5-cyanopyridin-2-yl)amino)cyclohexyl)ureido)phenyl)piperazin-1-yl)-*N*-(2-(2,6-dioxopiperidin-3-yl)-1,3-dioxoisoindolin-5-yl)pentanamide (**6f**)

Compound **6f** was synthesized by following a similar procedure as that of **5a**. ^1^H NMR (600 MHz, DMSO-*d*_6_) δ 11.13 (s, 1H), 10.69 (s, 1H), 8.29 (d, *J* = 5.7 Hz, 2H), 7.94 (d, *J* = 8.4 Hz, 1H), 7.87 (d, *J* = 8.2 Hz, 1H), 7.60 (d, *J* = 9.0 Hz, 1H), 7.50 (s, 1H), 7.27 (t, *J* = 7.4 Hz, 2H), 7.17 (dd, *J* = 13.8, 7.3 Hz, 3H), 6.99 (q, *J* = 8.7 Hz, 4H), 6.48 (d, *J* = 8.9 Hz, 1H), 5.58 (t, *J* = 6.1 Hz, 1H), 5.12 (dd, *J* = 12.9, 5.4 Hz, 1H), 4.26 (t, *J* = 11.7 Hz, 1H), 4.15 (d, *J* = 6.1 Hz, 2H), 3.51 (s, 1H), 3.18 (t, *J* = 4.9 Hz, 4H), 2.93–2.84 (m, 1H), 2.64–2.53 (m, 2H), 2.51–2.47 (m, 4H), 2.45 (t, *J* = 7.4 Hz, 2H), 2.35 (t, *J* = 7.2 Hz, 2H), 2.10–2.02 (m, 1H), 1.90 (d, *J* = 12.1 Hz, 2H), 1.76 (d, *J* = 12.0 Hz, 2H), 1.66 (p, *J* = 7.3 Hz, 2H), 1.53 (p, *J* = 7.3 Hz, 2H), 1.35–1.27 (m, 2H), 1.10 (q, *J* = 10.6, 2H). ^13^C NMR (151 MHz, DMSO-*d*_6_) δ 173.25, 172.84, 170.39, 167.51, 167.24, 159.70, 157.29, 153.55, 150.63, 145.64, 141.77, 133.23, 131.94, 128.57, 128.48 (4 C), 127.14(4 C), 126.69, 125.15, 125.12, 123.96, 119.59, 115.79, 113.30, 94.43, 57.98, 53.37 (2 C), 53.28, 49.42, 49.07, 48.10 (2 C), 43.92, 40.48, 36.82, 31.76 (2 C), 31.41, 30.65, 26.24, 23.28, 22.52. HRMS (ESI) for C_44_H_43_N_9_O_6_ [M + H]^+^, calcd: 794.3409, found: 794.3370. HPLC analysis: MeOH-H_2_O (73:27), 8.71 min, 99.2% purity.

#### 6-(4-(4-(3-Benzyl-1-((1r,4r)-4-((5-cyanopyridin-2-yl)amino)cyclohexyl)ureido)phenyl)piperazin-1-yl)-*N*-(2-(2,6-dioxopiperidin-3-yl)-1,3-dioxoisoindolin-5-yl)hexanamide (**6g**)

Compound **6g** was synthesized by following a similar procedure as that of **5a**. ^1^H NMR (600 MHz, DMSO-*d*_6_) δ 11.13 (s, 1H), 10.59 (s, 1H), 8.29 (d, *J* = 2.3 Hz, 1H), 8.26 (d, *J* = 1.7 Hz, 1H), 7.91 (dd, *J* = 8.2, 1.9 Hz, 1H), 7.87 (d, *J* = 8.2 Hz, 1H), 7.60 (d, *J* = 6.5 Hz, 0H), 7.48 (s, 1H), 7.27 (t, *J* = 7.6 Hz, 2H), 7.20–7.13 (m, 3H), 7.03–6.92 (m, 4H), 6.46 (d, *J* = 8.9 Hz, 1H), 5.57 (t, *J* = 6.1 Hz, 1H), 5.12 (dd, *J* = 12.9, 5.5 Hz, 1H), 4.25 (tt, *J* = 12, 3.0 Hz, 1H), 4.14 (d, *J* = 6.1 Hz, 2H), 3.51 (s, 1H), 3.16 (t, *J* = 4.8 Hz, 4H), 2.92–2.83 (m, 1H), 2.63–2.53 (m, 2H), 2.49 (t, *J* = 4.9 Hz, 4H), 2.41 (t, *J* = 7.4 Hz, 2H), 2.32 (t, *J* = 7.4 Hz, 2H), 2.08–2.02 (m, 1H), 1.90 (d, *J* = 11.8 Hz, 2H), 1.76 (d, *J* = 11.9 Hz, 2H), 1.65 (p, *J* = 7.5 Hz, 2H), 1.50 (p, *J* = 7.3 Hz, 2H), 1.35 (p, *J* = 8.1 Hz, 2H), 1.31 (q, *J* = 11.3, 9.8 Hz, 2H), 1.10 (q, *J* = 11.4 Hz, 2H). ^13^C NMR (151 MHz, DMSO-*d*_6_) δ 173.26, 172.86, 170.38, 167.50, 167.23, 159.68, 157.31, 153.54, 150.62, 145.57, 141.72, 133.23, 131.93, 128.53, 128.49 (4 C), 127.13 (4 C), 126.71, 125.18, 125.15, 123.97, 119.58, 115.78, 113.29, 94.43, 58.18, 53.36, 53.27 (2 C), 49.42, 49.06, 48.06 (2 C), 43.91, 40.42, 36.97, 31.74 (2 C), 31.39, 30.65, 27.00, 26.45, 25.26, 22.52. HRMS (ESI) for C_49_H_54_N_10_O_6_ [M + H]^+^, calcd: 879.4301, found: 879.4287. HPLC analysis: MeOH-H_2_O (75:25), 10.3 min, 98.7% purity.

#### 8-(4-(4-(3-Benzyl-1-((1r,4r)-4-((5-cyanopyridin-2-yl)amino)cyclohexyl)ureido)phenyl)piperazin-1-yl)-*N*-(2-(2,6-dioxopiperidin-3-yl)-1,3-dioxoisoindolin-5-yl)octanamide (**6h**)

Compound **6h** was synthesized by following a similar procedure as that of **5a**. ^1^H NMR (600 MHz, DMSO-*d*_6_) δ 11.12 (s, 1H), 10.58 (s, 1H), 8.29 (d, *J* = 2.3 Hz, 1H), 8.26 (d, *J* = 1.8 Hz, 1H), 7.91 (dd, *J* = 8.2, 1.9 Hz, 1H), 7.87 (d, *J* = 8.2 Hz, 1H), 7.60 (dd, *J* = 8.8, 2.4 Hz, 1H), 7.48 (s, 1H), 7.27 (t, *J* = 7.6 Hz, 2H), 7.19–7.14 (m, 3H), 7.02–6.93 (m, 4H), 6.47 (d, *J* = 8.9 Hz, 1H), 5.58 (t, *J* = 6.1 Hz, 1H), 5.12 (dd, *J* = 12.9, 5.5 Hz, 1H), 4.26 (tt, *J* = 12, 3.6 Hz, 1H), 4.15 (d, *J* = 6.1 Hz, 2H), 3.51 (s, 1H), 3.17 (t, *J* = 5.0 Hz, 4H), 2.92–2.84 (m, 1H), 2.63–2.52 (m, 2H), 2.47 (t, *J* = 5.0 Hz, 4H), 2.40 (t, *J* = 7.4 Hz, 2H), 2.30 (t, *J* = 7.4 Hz, 2H), 2.08–2.02 (m, 1H), 1.90 (d, *J* = 12.8 Hz, 2H), 1.76 (d, *J* = 10.9 Hz, 2H), 1.62 (p, *J* = 7.1 Hz, 2H), 1.46 (p, *J* = 7.1 Hz, 2H), 1.35–1.27 (m, 8H), 1.10 (q, *J* = 11.4 Hz, 2H). ^13^C NMR (151 MHz, DMSO-*d*_6_) δ 173.23, 172.85, 170.37, 167.49, 167.23, 159.70, 157.27, 153.55, 150.63, 145.60, 141.77, 133.24, 131.94, 128.56, 128.47 (4 C), 127.14 (4 C), 126.69, 125.16, 125.13, 123.93, 119.58, 115.77, 113.27, 94.44, 58.34, 56.49, 55.37, 53.36, 53.32, 49.42, 49.07, 48.10, 43.92, 40.49, 36.99, 31.76, 31.40, 30.65, 29.14, 29.01, 27.28, 26.67, 25.22, 22.52, 19.02. HRMS (ESI) for C_51_H_58_N_10_O_6_ [M + H]^+^, calcd: 907.4614, found: 907.4602. HPLC analysis: MeOH-H_2_O (80:20), 7.73 min, 95.8% purity.

#### 10-(4-(4-(3-Benzyl-1-((1r,4r)-4-((5-cyanopyridin-2-yl)amino)cyclohexyl)ureido)phenyl)piperazin-1-yl)-*N*-(2-(2,6-dioxopiperidin-3-yl)-1,3-dioxoisoindolin-5-yl)decanamide (**6i**)

Compound **6i** was synthesized by following a similar procedure as that of **5a**. ^1^H NMR (600 MHz, DMSO-*d*_6_) δ 11.12 (s, 1H), 10.80 (s, 1H), 8.29 (d, *J* = 2.1 Hz, 2H), 7.95 (dd, *J* = 8.3, 2.0 Hz, 1H), 7.86 (d, *J* = 8.2 Hz, 1H), 7.60 (d, *J* = 9.6 Hz, 1H), 7.52 (s, 1H), 7.27 (t, *J* = 7.5 Hz, 2H), 7.17 (dd, *J* = 13.2, 7.3 Hz, 3H), 6.98 (q, *J* = 8.9 Hz, 4H), 6.48 (d, *J* = 8.9 Hz, 1H), 5.58 (t, *J* = 6.2 Hz, 1H), 5.12 (dd, *J* = 12.9, 5.5 Hz, 1H), 4.26 (tt, *J* = 12, 3.0 Hz, 1H), 4.14 (d, *J* = 6.1 Hz, 2H), 3.51 (s, 1H), 3.17 (t, *J* = 4.7 Hz, 4H), 2.93–2.84 (m, 1H), 2.63–2.53 (m, 2H), 2.47 (t, *J* = 5.0 Hz, 4H), 2.41 (t, *J* = 7.4 Hz, 2H), 2.29 (t, *J* = 7.5 Hz, 2H), 2.10–2.02 (m, 1H), 1.90 (d, *J* = 11.8 Hz, 2H), 1.76 (d, *J* = 11.9 Hz, 2H), 1.62 (t, *J* = 7.0 Hz, 2H), 1.44 (t, *J* = 7.3 Hz, 2H), 1.34–1.22 (m, 12H),1.10 (q, *J* = 10.8 Hz, 2H). ^13^C NMR (151 MHz, DMSO-*d*_6_) δ 173.23, 173.02, 170.38, 167.53, 167.27, 159.72, 157.25, 153.54, 150.50, 145.87, 141.79, 133.16, 131.95, 128.68, 128.47 (4 C), 127.14 (4C), 126.67, 125.01 (2 C), 123.94, 119.61, 115.87, 113.30, 94.36, 58.14, 53.38 (2 C), 53.10, 49.40 (2 C), 49.03, 47.88, 43.92, 40.52, 36.93, 31.75(2 C), 31.42, 30.66, 29.31, 29.20, 29.04, 27.77, 27.33, 26.63, 25.34, 22.54. HRMS (ESI) for C_53_H_62_N_10_O_6_ [M + H]^+^, calcd: 935.4927, found: 935.4915. HPLC analysis: MeOH-H_2_O (80:20), 13.61 min, 99.5% purity.

#### 3-Benzyl-1-((1r,4r)-4-((5-cyanopyridin-2-yl)amino)cyclohexyl)-1-(4-(4-(3-(2-(2,6-dioxopiperidin-3-yl)-1,3-dioxoisoindolin-5-yl)propioloyl)piperazin-1-yl)phenyl)urea (**7a**)

TFA (3 mL) was added to a suspension of *tert*-butyl 3-(2-(2,6-dioxopiperidin-3-yl)-1,3-dioxoisoindolin-5-yl)propiolate **16a** (57 mg, 0.15 mmol) in DCM (6 mL). After stirring at room temperature for 2 h, the reaction mixture was quenched with water and extracted with ethyl acetate three times. The combined organic phases were concentrated to dryness under reduced pressure. The resultant crude material was added to a suspension of intermediate **4** (51 mg, 0.1 mmol), HATU (57 mg, 0.15 mmol), and DIPEA (39 mg, 0.3 mmol) in DMF (10 mL). The mixture was stirred at room temperature for 15 min, evaporated under vacuum, and purified by silica column chromatography to afford the title compound as a white solid (62 mg, yield 75%). ^1^H NMR (600 MHz, DMSO-*d*_6_) δ 11.17 (s, 1H), 8.30 (d, *J* = 2.3 Hz, 1H), 8.25 (s, 1H), 8.15 (dd, *J* = 7.7, 1.4 Hz, 1H), 8.05–8.00 (m, 1H), 7.63–7.57 (m, 1H), 7.48 (s, 1H), 7.27 (t, *J* = 7.6 Hz, 2H), 7.17 (dd, *J* = 11.1, 7.4 Hz, 3H), 7.08–7.00 (m, 4H), 6.47 (d, *J* = 8.9 Hz, 1H), 5.61 (t, *J* = 5.8 Hz, 1H), 5.20 (dd, *J* = 12.9, 5.5 Hz, 1H), 4.27 (tt, *J* = 12, 3.6 Hz, 1H), 4.15 (d, *J* = 6.0 Hz, 2H), 3.97 (t, *J* = 5.1 Hz, 2H), 3.71 (t, *J* = 5.2 Hz, 2H), 3.51 (s, 1H), 3.33 (t, *J* = 5.3 Hz, 2H), 3.27 (t, *J* = 5.4 Hz, 2H), 2.95–2.86 (m, 1H), 2.65–2.52 (m, 2H), 2.12–2.06 (m, 1H), 1.91 (d, *J* = 12.0 Hz, 2H), 1.78 (d, *J* = 10.6 Hz, 2H), 1.31 (q, *J* = 11.0 Hz, 2H), 1.11 (q, *J* = 11.0 Hz, 2H). ^13^C NMR (151 MHz, DMSO-*d*_6_) δ 173.19, 170.15, 166.78, 166.65, 159.69, 157.22, 153.56, 151.71, 150.20, 141.77, 139.12, 132.45, 132.24, 132.12, 129.37, 128.48 (4 C), 127.38, 127.17 (4C), 126.70, 126.30, 124.37, 119.58, 116.58, 94.45, 87.89, 84.67, 53.40, 49.68, 49.06, 48.74, 48.15, 46.77, 43.95, 41.58, 40.52, 31.77 (2 C), 31.38, 30.67, 22.37. HRMS (ESI) for C_46_H_43_N_9_O_6_ [M + H]^+^, calcd: 818.3409, found: 818.3375. HPLC analysis: MeOH-H_2_O (75:25), 5.35 min, 98.9% purity.

#### 3-Benzyl-1-((1r,4r)-4-((5-cyanopyridin-2-yl)amino)cyclohexyl)-1-(4-(4-((*E*)-3-(2-(2,6-dioxopiperidin-3-yl)-1,3-dioxoisoindolin-5-yl)acryloyl)piperazin-1-yl)phenyl)urea (**7b**)

Compound **7b** was synthesized by following a similar procedure as that of **7a**. ^1^H NMR (600 MHz, DMSO-*d*_6_) δ 11.16 (s, 1H), 8.47 (s, 1H), 8.29 (d, *J* = 2.2 Hz, 1H), 8.18 (dd, *J* = 7.8, 1.4 Hz, 1H), 7.95 (d, *J* = 7.7 Hz, 1H), 7.70 (s, 2H), 7.60 (d, *J* = 8.3 Hz, 1H), 7.48 (s, 1H), 7.27 (t, *J* = 7.6 Hz, 2H), 7.21–7.14 (m, 3H), 7.05 (s, 4H), 6.47 (d, *J* = 8.9 Hz, 1H), 5.60 (t, *J* = 6.1 Hz, 1H), 5.19 (dd, *J* = 12.9, 5.5 Hz, 1H), 4.27 (tt, *J* = 12, 3.6 Hz, 1H), 4.16 (d, *J* = 6.0 Hz, 2H), 3.94 (s, 2H), 3.75 (s, 2H), 3.49 (s, 1H), 3.27 (d, *J* = 14.4 Hz, 4H), 2.97–2.84 (m, 1H), 2.65–2.52 (m, 2H), 2.14–2.02 (m, 1H), 1.91 (d, *J* = 11.8 Hz, 2H), 1.78 (d, *J* = 11.7 Hz, 2H), 1.31 (q, *J* = 12.4 Hz, 2H), 1.11 (q, *J* = 12.2, 11.5 Hz, 2H). ^13^C NMR (151 MHz, DMSO-*d*_6_) δ 173.24, 170.29, 167.40, 167.21, 164.43, 159.69, 157.25, 153.55, 150.35, 142.25, 141.75, 140.12, 135.35, 132.57, 132.06, 131.64, 129.14, 128.48 (4 C), 127.16 (4 C), 126.70, 124.36, 122.84, 122.61, 119.58, 116.35, 94.45, 53.39, 49.56, 49.07, 48.90, 48.25, 45.36, 43.94, 42.17, 40.49, 31.76 (2 C), 31.41, 30.67, 22.45. HRMS (ESI) for C_46_H_45_N_9_O_6_ [M + H]^+^, calcd: 820.3566, found: 820.3551. HPLC analysis: MeOH-H_2_O (75:25), 5.32 min, 99.5% purity.

#### 3-Benzyl-1-((1r,4r)-4-((5-cyanopyridin-2-yl)amino)cyclohexyl)-1-(4-(4-(2-(2-(2,6-dioxopiperidin-3-yl)-1,3-dioxoisoindolin-5-yl)cyclopropane-1-carbonyl)piperazin-1-yl)phenyl)urea (**7c**)

Compound **7c** was synthesized by following a similar procedure as that of **7a**. ^1^H NMR (400 MHz, DMSO-*d*_6_) δ 11.11 (s, 1H), 8.29 (d, *J* = 2.3 Hz, 1H), 7.77 (d, *J* = 7.7 Hz, 1H), 7.71–7.66 (m, 1H), 7.66–7.63 (m, 1H), 7.60 (dd, *J* = 8.9, 2.4 Hz, 1H), 7.48 (d, *J* = 7.5 Hz, 1H), 7.27 (t, *J* = 7.4 Hz, 2H), 7.21–7.11 (m, 3H), 6.99 (dd, *J* = 8.9, 2.9 Hz, 2H), 6.91 (d, *J* = 8.7 Hz, 2H), 6.48 (d, *J* = 8.9 Hz, 1H), 5.55 (t, *J* = 6.0 Hz, 1H), 5.10 (dd, *J* = 12.7, 5.4 Hz, 1H), 4.25 (tt, *J* = 12, 3.6 Hz, 1H), 4.15 (d, *J* = 6.0 Hz, 2H), 3.821–3.71 (m, 1H), 3.70–3.61 (m, 1H), 3.55–3.40 (m, 3H), 3.30–3.20 (m, 1H), 3.16–3.05 (m, 1H), 2.92–2.73 (m, 3H), 2.73–2.64 (m, 1H), 2.63–2.52 (m, 3H), 2.08–2.198 (m, 1H), 1.90 (d, *J* = 11.7 Hz, 2H), 1.82–1.71 (m, 3H), 1.41 (td, *J* = 8.1, 4.9 Hz, 1H), 1.36–1.27 (m, 2H), 1.08 (q, *J* = 12.4 Hz, 2H). ^13^C NMR (151 MHz, DMSO-*d*_6_) δ 173.18, 170.31, 167.63, 167.44, 166.79, 159.70, 157.22, 153.54, 150.25, 146.72, 141.75, 135.16, 132.00, 131.54, 129.37, 129.18, 128.48 (4 C), 127.13 (4 C), 126.69, 123.39, 122.92, 119.59, 116.37, 94.44, 53.41, 49.40, 49.05, 48.87, 48.35, 45.01, 43.93, 41.72, 40.50, 31.76 (2 C), 31.39, 30.63, 24.48, 24.28, 22.48, 11.58. HRMS (ESI) for C_47_H_47_N_9_O_6_ [M + H]^+^, calcd: 834.3722, found: 834.3688. HPLC analysis: MeOH-H_2_O (70:30), 6.81 min, 100% purity.

#### 3-Benzyl-1-((1r,4r)-4-((5-cyanopyridin-2-yl)amino)cyclohexyl)-1-(4-(4-(3-(2-(2,6-dioxopiperidin-3-yl)-1,3-dioxoisoindolin-5-yl)propanoyl)piperazin-1-yl)phenyl)urea (**7d**)

Compound **7d** was synthesized by following a similar procedure as that of **7a**. ^1^H NMR (600 MHz, DMSO-*d*_6_) δ 11.14 (s, 1H), 8.29 (d, *J* = 2.3 Hz, 1H), 7.86 (s, 1H), 7.83 (d, *J* = 7.7 Hz, 1H), 7.78 (d, *J* = 7.7 Hz, 1H), 7.60 (d, *J* = 6.7 Hz, 1H), 7.49 (s, 1H), 7.27 (t, *J* = 7.6 Hz, 2H), 7.17 (dd, *J* = 11.6, 7.4 Hz, 3H), 7.01 (q, *J* = 9.0 Hz, 4H), 6.47 (d, *J* = 8.9 Hz, 1H), 5.58 (t, *J* = 6.2 Hz, 1H), 5.14 (dd, *J* = 12.9, 5.5 Hz, 1H), 4.26 (tt, *J* = 11.7, 3.6 Hz, 1H), 4.15 (d, *J* = 6.0 Hz, 2H), 3.60 (t, *J* = 5.2 Hz, 4H), 3.49 (s, 1H), 3.17 (dt, *J* = 17.2, 5.2 Hz, 4H), 3.04 (t, *J* = 7.4 Hz, 2H), 2.95–2.84 (m, 1H), 2.81 (t, *J* = 7.5 Hz, 2H), 2.64–2.52 (m, 2H), 2.10–2.01 (m, 1H), 1.90 (d, *J* = 11.9 Hz, 2H), 1.77 (d, *J* = 9.6 Hz, 2H), 1.31 (q, *J* = 13.2, 11.2 Hz, 2H), 1.09 (q, *J* = 12.1, 11.4 Hz, 2H). ^13^C NMR (151 MHz, DMSO-*d*_6_) δ 173.24, 170.35, 170.08, 167.77, 167.58, 159.69, 157.24, 153.55, 150.35, 150.24, 141.74, 135.47, 132.03, 131.98, 129.47, 129.07, 128.48 (4 C), 127.15 (4 C), 126.70, 124.04, 123.79, 119.58, 116.28, 94.44, 53.39, 49.43, 49.06, 48.48, 48.19, 45.04, 43.94, 41.46, 40.49, 33.72, 31.76 (2 C), 31.42, 31.17, 30.66, 22.50. HRMS (ESI) for C_46_H_47_N_9_O_6_[M + H]^+^, calcd: 822.3722, found: 822.3692. HPLC analysis: MeOH-H_2_O (75:25), 4.99 min, 99.4% purity.

#### 3-Benzyl-1-((1r,4r)-4-((5-cyanopyridin-2-yl)amino)cyclohexyl)-1-(4-(4-(2-((2-(2,6-dioxopiperidin-3-yl)-1,3-dioxoisoindolin-5-yl)oxy)acetyl)piperazin-1-yl)phenyl)urea (**7e**)

Compound **7e** was synthesized by following a similar procedure as that of **7a**. ^1^H NMR (600 MHz, DMSO-*d*_6_) δ 11.12 (s, 1H), 8.29 (d, *J* = 2.3 Hz, 1H), 7.84 (d, *J* = 8.3 Hz, 1H), 7.60 (d, *J* = 8.8 Hz, 1H), 7.50 (s, 1H), 7.47 (d, *J* = 2.3 Hz, 1H), 7.37 (dd, *J* = 8.3, 2.3 Hz, 1H), 7.27 (t, *J* = 7.5 Hz, 2H), 7.17 (dd, *J* = 12.6, 7.3 Hz, 3H), 7.03 (s, 4H), 6.48 (d, *J* = 8.9 Hz, 1H), 5.60 (t, *J* = 6.3 Hz, 1H), 5.20 (s, 2H), 5.12 (dd, *J* = 12.9, 5.4 Hz, 1H), 4.26 (tt, *J* = 12.0, 3.6 Hz, 1H), 4.15 (d, *J* = 6.1 Hz, 2H), 3.62 (t, *J* = 4.9 Hz, 4H), 3.51 (s, 1H), 3.30 (t, *J* = 5.1 Hz, 2H), 3.22 (t, *J* = 5.1 Hz, 2H), 2.93–2.84 (m, 1H), 2.63–2.52 (m, 2H), 2.09–2.01 (m, 1H), 1.91 (d, *J* = 11.9 Hz, 2H), 1.78 (d, *J* = 11.8 Hz, 2H), 1.31 (q, *J* = 10.2 Hz, 2H), 1.10 (q, *J* = 11.9 Hz, 2H). ^13^C NMR (151 MHz, DMSO-*d*_6_) δ 173.26, 170.40, 167.40, 167.24, 165.66, 164.14, 159.69, 157.26, 153.55, 150.31, 141.73, 134.16, 132.06, 129.14, 128.49 (4 C), 127.15 (4 C), 126.71, 125.63, 123.69, 121.68, 119.59, 116.37, 109.56, 94.43, 66.60, 53.39, 49.44, 49.06, 48.35, 48.15, 44.26, 43.93, 41.62, 40.46, 31.75 (2 C), 31.41, 30.66, 22.51. HRMS (ESI) for C_45_H_45_N_9_O_7_ [M + H]^+^, calcd: 824.3515, found: 824.3475. HPLC analysis: MeOH-H_2_O (78:22), 4.92 min, 98.9% purity.

#### 3-Benzyl-1-((1r,4r)-4-((5-cyanopyridin-2-yl)amino)cyclohexyl)-1-(4-(4-((2-(2,6-dioxopiperidin-3-yl)-1,3-dioxoisoindolin-5-yl)glycyl)piperazin-1-yl)phenyl)urea (**7f**)

Compound **7f** was synthesized by following a similar procedure as that of **7a**. ^1^H NMR (600 MHz, DMSO-*d*_6_) δ 11.07 (s, 1H), 8.30 (d, *J* = 2.5 Hz, 1H), 7.60 (t, *J* = 9.4 Hz, 2H), 7.50 (s, 1H), 7.28 (t, *J* = 7.5 Hz, 2H), 7.20–7.12 (m, 5H), 7.04 (m, 5H), 6.48 (d, *J* = 8.9 Hz, 1H), 5.61 (t, *J* = 6.2 Hz, 1H), 5.05 (dd, *J* = 12.8, 5.5 Hz, 1H), 4.27 (tt, *J* = 11.9, 3.6 Hz, 1H), 4.21 (d, *J* = 5.1 Hz, 2H), 4.16 (d, *J* = 6.0 Hz, 2H), 3.68 (dt, *J* = 14.7, 5.3 Hz, 4H), 3.51 (s, 1H), 3.30 (d, *J* = 4.9 Hz, 2H), 3.22 (t, *J* = 5.2 Hz, 2H), 2.93–2.84 (m, 1H), 2.62–2.53 (m, 2H), 2.05–1.98 (m, 1H), 1.91 (d, *J* = 11.9 Hz, 2H), 1.78 (d, *J* = 11.8 Hz, 2H), 1.31 (q, *J* = 11.1 Hz, 2H), 1.11 (q, *J* = 12.5 Hz, 2H).^13^C NMR (151 MHz, DMSO-*d*_6_) δ 173.29, 170.63, 168.21, 167.67, 167.32, 159.70, 157.24, 154.49, 153.56, 150.35, 141.76, 134.48, 132.07, 129.15, 128.49 (4 C), 127.16 (4 C), 126.70, 125.24, 119.59, 117.13, 116.37 (2 C), 94.44, 53.39, 49.11, 48.42, 48.18, 44.75, 44.29, 43.94, 41.81, 40.88, 40.50, 31.76 (2 C), 31.45, 30.67, 22.70. HRMS (ESI) for C_45_H_46_N_10_O_6_[M + H]^+^, calcd: 823.3675, found: 823.3671. HPLC analysis: MeOH-H_2_O (75:25), 5.00 min, 100% purity.

#### 3-Benzyl-1-((1r,4r)-4-((5-cyanopyridin-2-yl)amino)cyclohexyl)-1-(4-(4-((2-(2,6-dioxopiperidin-3-yl)-3-oxoisoindolin-5-yl)glycyl)piperazin-1-yl)phenyl)urea (**8a**)

Compound **8a** was synthesized by following a similar procedure as that of **7a**. ^1^H NMR (400 MHz, DMSO-*d*_6_) δ 10.94 (s, 1H), 8.29 (d, *J* = 2.3 Hz, 1H), 7.60 (dd, *J* = 8.9, 2.4 Hz, 1H), 7.52 (d, *J* = 7.6 Hz, 1H), 7.32–7.24 (m, 3H), 7.21–7.13 (m, 3H), 7.09–6.99 (m, 5H), 6.93 (d, *J* = 2.2 Hz, 1H), 6.49 (d, *J* = 8.9 Hz, 1H), 5.97 (t, *J* = 5.1 Hz, 1H), 5.59 (t, *J* = 6.0 Hz, 1H), 5.08 (dd, *J* = 13.3, 5.1 Hz, 1H), 4.28 (d, *J* = 16.3 Hz, 2H), 4.20–4.13 (m, 3H), 4.06 (d, *J* = 5.0 Hz, 2H), 3.68 (d, *J* = 17.6 Hz, 4H), 3.48 (s, 1H), 3.28 (s, 2H), 3.21 (s, 2H), 2.97–2.84 (m, 1H), 2.63–2.55 (m, 1H), 2.43–2.30 (m, 1H), 2.03–1.94 (m, 1H), 1.91 (d, *J* = 11.8 Hz, 2H), 1.78 (d, *J* = 11.8 Hz, 2H), 1.32 (q, *J* = 12.7, 12.2 Hz, 2H), 1.11 (q, *J* = 13.5, 12.6 Hz, 2H). ^13^C NMR (151 MHz, DMSO-*d*_6_) δ 173.38, 171.61, 169.25, 168.22, 159.71, 157.24, 153.56, 150.37, 148.92, 141.78, 132.97, 132.06, 130.07, 129.14, 128.48 (4 C), 127.16 (4 C), 126.70, 123.91, 119.60, 118.38, 116.35, 104.98, 94.41, 53.40, 52.06, 49.05, 48.49, 48.20, 47.07, 45.31, 44.31, 43.94, 41.77, 40.51, 31.76 (2 C), 31.71, 30.67, 23.01. HRMS (ESI) for C_45_H_48_N_10_O_5_ [M + H]^+^, calcd: 809.3882, found: 809.3865. HPLC analysis: MeOH-H_2_O (70:30), 6.29 min, 96.2% purity.

#### 3-Benzyl-1-((1r,4r)-4-((5-cyanopyridin-2-yl)amino)cyclohexyl)-1-(4-(4-((2-(2,6-dioxopiperidin-3-yl)-1-oxoisoindolin-5-yl)glycyl)piperazin-1-yl)phenyl)urea (**8b**)

Compound **8b** was synthesized by following a similar procedure as that of **7a**. ^1^H NMR (400 MHz, DMSO-*d*_6_) δ 10.92 (s, 1H), 8.30 (d, *J* = 2.3 Hz, 1H), 7.60 (dd, *J* = 8.9, 2.4 Hz, 1H), 7.48 (d, *J* = 7.6 Hz, 1H), 7.42 (d, *J* = 8.4 Hz, 1H), 7.31–7.24 (m, 2H), 7.22–7.13 (m, 3H), 7.07–6.99 (m, 4H), 6.82 (dd, *J* = 8.4, 2.0 Hz, 1H), 6.77 (s, 1H), 6.48 (d, *J* = 8.9 Hz, 1H), 6.38 (t, *J* = 5.1 Hz, 1H), 5.58 (t, *J* = 6.1 Hz, 1H), 5.02 (dd, *J* = 13.2, 5.1 Hz, 1H), 4.33–4.22 (m, 2H), 4.20–4.12 (m, 3H), 4.08 (d, *J* = 5.0 Hz, 2H), 3.66 (s, 4H), 3.49 (s, 1H), 3.29 (s, 2H), 3.22 (s, 2H), 2.96–2.82 (m, 1H), 2.63–2.54 (m, 1H), 2.36 (qd, *J* = 13.1, 4.4 Hz, 1H), 2.00–1.85 (m, 3H), 1.78 (d, *J* = 11.5 Hz, 2H), 1.32 (q, *J* = 11.3 Hz, 2H), 1.11 (q, *J* = 12.4 Hz, 2H). ^13^C NMR (151 MHz, DMSO-*d*_6_) δ 173.43, 171.86, 169.14, 167.85, 159.70, 157.23, 153.56, 152.06, 150.35, 144.72, 141.77, 132.06, 129.15, 128.49 (4 C), 127.16 (4 C), 126.70, 124.26, 119.87, 119.59, 116.36 (2 C), 113.47, 105.59, 94.45, 53.39, 51.77, 49.08, 48.42, 48.18, 47.25, 44.94, 44.30, 43.94, 41.79, 40.51, 31.76 (3 C), 30.67, 23.09. HRMS (ESI) for C_45_H_48_N_10_O_5_ [M + H]^+^, calcd: 809.3882, found: 809.3897. HPLC analysis: MeOH-H_2_O (70:30), 5.87 min, 97.7% purity.

#### 3-Benzyl-1-((1r,4r)-4-((5-cyanopyridin-2-yl)amino)cyclohexyl)-1-(4-(4-((2-(1-methyl-2,6-dioxopiperidin-3-yl)-1,3-dioxoisoindolin-5-yl)glycyl)piperazin-1-yl)phenyl)urea (**8c**)

Compound **8c** was synthesized by following a similar procedure as that of **7a**. ^1^H NMR (400 MHz, DMSO-*d*_6_) δ 8.30 (d, *J* = 2.3 Hz, 1H), 7.65–7.55 (m, 2H), 7.48 (d, *J* = 7.5 Hz, 1H), 7.33–7.23 (m, 2H), 7.22–7.11 (m, 5H), 7.10–6.98 (m, 5H), 6.47 (d, *J* = 8.9 Hz, 1H), 5.60 (t, *J* = 6.1 Hz, 1H), 5.11 (dd, *J* = 13.0, 5.4 Hz, 1H), 4.32–4.24 (m, 1H), 4.21 (d, *J* = 5.0 Hz, 2H), 4.16 (d, *J* = 6.0 Hz, 2H), 3.66 (s, 4H), 3.48 (s, 1H), 3.30 (s, 2H), 3.23 (s, 2H), 3.01 (s, 3H), 2.98–2.87 (m, 1H), 2.80–2.71 (m, 1H), 2.61–2.53 (m, 1H), 2.08–1.97 (m, 1H), 1.91 (d, *J* = 11.6 Hz, 2H), 1.78 (d, *J* = 11.5 Hz, 2H), 1.31 (q, *J* = 13.7, 13.1 Hz, 2H), 1.11 (q, *J* = 11.5 Hz, 2H). ^13^C NMR (151 MHz, DMSO-*d*_6_) δ 172.27, 170.38, 168.21, 167.66, 167.31, 159.70, 157.23, 154.51, 153.56, 150.35, 141.77, 134.46, 132.07, 129.16, 128.48, 127.16, 126.70, 125.27, 119.58, 117.11, 116.37, 94.45, 53.39, 49.70, 49.07, 48.42, 48.18, 44.76, 44.29, 43.95, 41.81, 40.52, 31.77 (2 C), 31.62, 30.67, 27.06, 21.89. HRMS (ESI) for C_46_H_48_N_10_O_6_ [M + H]^+^, calcd: 837.3831, found: 837.3804. HPLC analysis: MeOH-H_2_O (70:30), 10.13 min, 98.9% purity.

### Protein Expression and Purification

Synthetic genes comprising the kinase domain of human CDK12 (UniProt accession number Q9NYV4, residues 715–1052), kinase domain of human CDK13 (UniProt accession number Q14004, residues 694–1039), the cyclin box domain of human Cyclin K (UniProt accession number O75909, residues 1–267), and full-length CAK1 from *Saccharomyces cerevisiae* (UniProt accession number P43568, residues 1–368) were codon-optimized for expression in Sf9 insect cells. CDK12 and Cyclin K were fused with an N-terminal hexahistidine tag followed by a tobacco etch virus (TEV) protease cleavage site and were co-cloned into a pFastBac Dual expression vector while CAK1 was cloned into a pFastBac1 expression vector using the Invitrogen Baculovirus Expression System. DNA was prepared in *Escherichia coli* strain DH10Bac and used to generate baculovirus in Sf9 insect cells. Baculoviruses of CDK12-cyclin K and CAK1 were used to co-infect Sf9 cells. The cultures were centrifuged for 10 min at 5000 rpm at 4 °C for 48–72 h post infection. The cell pellet was suspended in lysis buffer (25 mM HEPES, 300 mM NaCl, 1 mM TCEP, pH 7.5) and lysed by a high-pressure homogenizer. The cell debris was then removed by centrifugation at 18,000 rpm at 4 °C for 1 h. Recombinant proteins in the supernatant were purified using nickel-sepharose resin (GE Healthcare) and eluted stepwise with imidazole. Fractions containing the CDK12-cyclin K complex were treated with TEV protease for cleavage of the N-terminal hexahistidine tags. Proteins were further purified using the reverse nickel-affinity method. For final purification, proteins were applied to a Superdex 200 size-exclusion column equilibrated with storage buffer (25 mM HEPES, 150 mM NaCl, 2 mM DTT, pH 7.5). Proteins were pooled, concentrated to 10 mg/mL, flash-frozen in liquid nitrogen, and stored at −80 °C. The expression and purification of CDK13/Cyclin K are similar to CDK12/Cyclin K.

### Biolayer Interferometry

All measurements of binding kinetics and dissociation constants were performed by a biolayer interferometry assay using an Octet Red 96 (Forté Bio). Proteins of CDK12-Cyclin K and CDK13-CycK were biotinylated by EZ-Link NHS-Biotin (ThermoFisher Scientific). All assays were run at 28 °C using phosphate-buffered saline (PBS) (pH 7.4, 0.02% Tween 20) as the assay buffer. Super Streptavidin (SSA) biosensor tips (ForteBio) were used to immobilize the biotinylated proteins after prewetting with the assay buffer. The equilibrated SSA biosensors were loaded with proteins (50 μg/mL). Background binding controls used a duplicate set of sensors that were incubated in a buffer without proteins. Association–dissociation cycles were performed by moving and dipping tips into compound solution wells and then into pure assay buffer wells. The signals were analyzed by a double reference subtraction protocol to deduce nonspecific and background signals and signal drifts caused by biosensor variability. A 1:1 binding model was used to fit the association and dissociation rates. Equilibrium dissociation constant (*K*_D_) values were calculated from the ratio of *K*_off_ to *K*_on_.

### Cell Culture of Human Normal Breast and TNBC Cell Lines

MDA-MD-231, MFM223, MDA-MB-436, and HCC1395 cells were purchased from ATCC, and each cell line was cultured according to ATCC guidelines. Cell lines were tested for mycoplasma using the Lonza MycoAlert kit following the manufacturer’s protocol. Cell line authentication was provided by Labcorp (Burlington NC).

### Western Blot Analysis

Cell lysates were prepared in Cell Lysis buffers (ThermoFisher Scientific) supplemented with completeTM protease inhibitor cocktail tablets (Sigma-Aldrich). The protein concentration was tested using a Pierce bicinchoninic acid (BCA) Protein Assay Kit (ThermoFisher Scientific). An equal amount of protein was resolved in Tris-Acetate Protein Gel (ThermoFisher Scientific) and blotted with primary antibodies. Following incubation with HRP-conjugated secondary antibodies, membranes were imaged on an Odyssey CLx Imager (LiCOR Biosciences).

### Cellular Proliferation Assays

Cells were plated in 96-well plates and incubated at 37 °C and 5% CO_2_. After overnight incubation, serial dilutions of compounds were added to the plate. After 5 days, the assay was stopped with Cell Titer-Glo (Promega). The luminescence signal was detected by the Infinite M1000 Pro plate reader (Tecan), and data were analyzed using GraphPad Prism software (GraphPad).

### TMT-Labeled Quantitative Proteomics Assay

MFM223 cells were seeded at 5 × 10^6^ cells in 100 mm plates. Compound **7f** was added and incubated for 5 h. Cell lysates were prepared in radioimmunoprecipitation assay (RIPA) buffer (Thermo Fisher Scientific), and the protein concentration was determined by a Pierce BCA Protein Assay Kit (ThermoFisher Scientific). The cell lysates were proteolyzed and labeled with a TMT 10-plex Isobaric Label Reagent (Thermo Fisher Scientific, 90110) following the manufacturer’s protocol. Briefly, upon reduction and alkylation of cysteines, the proteins were precipitated by adding 6 volumes of ice-cold acetone followed by overnight incubation at −20 °C. The precipitate was pelleted by centrifugation, and the protein pellet was allowed to air dry. The pellet was resuspended in 0.1 M TEAB and digested overnight with trypsin (1:50 enzyme:protein) at 37 °C with constant mixing using a thermomixer. The TMT 10-plex reagents were dissolved in 41 mL of anhydrous acetonitrile, and labeling was performed by transferring the entire digest to the TMT reagent vial and incubating it at room temperature for 1 h. The reaction was quenched by adding 8 mL of 5% hydroxylamine and followed by a 15 min incubation. Labeled samples were mixed together and dried using a vacufuge. An offline fractionation of the combined sample (200 mg) into 10 fractions was performed using a high-pH reversed-phase peptide fractionation kit according to the manufacturer’s protocol (Pierce, 84868). Fractions were dried and reconstituted in 12 mL of 0.1% formic acid/2% acetonitrile in preparation for LC-MS/MS analysis.

### Computational Modeling

The structures of CDK12 (PDB ID: 6CKX), CDK13 (PDB ID: 5EFQ), and CRBN (PDB ID: 4TZ4) were prepared using Protein Preparation Wizard (Schrödinger, LLC, New York, NY, 2021). The compound **4** was prepared using Ligprep and docked into the ATP binding pocket of CDK12 and CDK13 via Glide with default settings. In line with previous work,^28^ protein–protein docking was carried out by ROSETTA. The complex of thalidomide in CRBN was docked to the complex structures of compound **4** in CDK12 and CDK13. A flat-bottom harmonic potential was applied to the distance between the 4-position of the benzene ring in thalidomide and the piperidine nitrogen in compound **4**. With this potential, no force was added between the two atoms if the distance is between 2 and 17 Å. For the protein–protein docking, 15,000 complexes were sampled for each system and 1500 top-scored conformations were selected for further analysis. The conformations were further clustered by the fraction of common contact (FCC) clustering method^61^ and selected based on the distance and spatial relative position of compound **4** and lenalidomide. Next, compound **7f** was docked into the predicted CDK12/13 and CRBN complexes by the Autodock Vina via AMdock tool^62^ with default settings to give the primary structures of the ternary complex. The obtained structures were then submitted to 500 ns molecular dynamics (MD) simulations using Desmond. The OPLS4 force field was used for the proteins and small molecules. The SPC solvent model was used to solvate the system. Before simulation, the default protocol was used to relax the systems. The NPT ensembles with the temperature at 300 K and pressure at 1 atm were used for the MD simulations.

### Animal Experiment

All of the animal experiments were performed under an approved animal protocol (Protocol ID: PRO00010006, PI, Arul Chinnaiyan) by the Institutional Animal Care & Use Committee of the University of Michigan. Six- to eight-week-old NSG (Jackson Laboratory) or CB17SCID female (Charles River Laboratory) mice were in a regular SPF housing room prior to cell injection. Briefly, 5 × 10^6^ cells of MDA-MB-436 or MDA-MB-231 were injected orthotopically into the mammary fat pad of NSG or CB17SCID mice, respectively. After tumor size reached approximately 200–400 mm^3^, animals were subjected to drug treatment. 50 mg/kg of compound **7b** was administered to animals by i.v. injection for 6 h. Vehicle consisted of 20% PEG400, 6% Cremophor EL, and 74% PBS solution. Tumors were collected at the end of the experiment for Western blot analysis.

## Supporting Information Available

Western blot analysis of CDK12 and 13 proteins treated with all degraders in MDA-MB-231 cells; Pharmacokinetic profiles of Compound 7f in Sprague-Dawley (SD) rats; Pharmacodynamic study of **7b** in the MDA-MB-231 xenograft mouse model; Synthetic procedures of intermediates; ^1^H NMR and ^13^C NMR spectra, HRMS and HPLC traces of all degraders and negative control (PDF)
Molecular formula strings (CSV)
MD-simulated structure of the ternary complex CDK12-**7f**-CRBN (PDB)
MD-simulated structure of the ternary complex CDK13-**7f**-CRBN (PDB)

## Supplementary Material

jm2c00384_si_002.csv

jm2c00384_si_003.pdb

jm2c00384_si_004.pdb

## Abbreviations Used

ADC: antibody–drug conjugate
CDK: cyclin-dependent kinase
CCNK: cyclin K
CRBN: cereblon
CTD: C-terminal domain
DDR: DNA damage response
DC_50_: the half maximal degradation concentration
DC_90_: the concentration of degrading 90% protein
DIPEA: *N,N*-diisopropylethylamine
DMAP: 4-dimethylaminopyridine
DMF: *N,N*-dimethylformamide
DMSO: dimethyl sulfoxide
ER: estrogen receptor
HER2: human epidermal growth factor receptor 2
HR: homologous recombination
HATU: 2-(7-azabenzotriazol-1-yl)-*N,N,N*′*,N*′-tetramethyluronium hexafluorophosphate
MD: molecular dynamics
PROTAC: proteolysis targeting chimeras
PR: progesterone receptor
PARP: poly(ADP-ribose) polymerase
PD: pharmacokinetic properties
POI: protein of interest
PPI: protein–protein interaction
RNAP II: RNA polymerase II
TFA: trifluoroacetic acid
TNBC: triple-negative breast cancer
