# Supplementary material for: Discovery of a Highly Potent and Selective Dual PROTAC Degrader of CDK12 and CDK13
Source: J Med Chem. 2022 Aug 8;65(16):11066–83. doi: 10.1021/acs.jmedchem.2c00384 (PMC9876424; doi:10.1021/acs.jmedchem.2c00384)
Supplement: Supplementary file 1 — jm2c00384_si_001.pdf [file jm2c00384_si_001.pdf]

Supporting Information

Discovery of a Highly Potent and Selective Dual PROTAC Degrader of CDK12 and CDK13

Jianzhang Yang[1,#], Yu Chang[1,2,#], Jean Ching-Yi Tien[2,4], Zhen Wang[3], Yang Zhou[1], Pujuan Zhang[3], Weixue Huang[3], Josh Vo[2], Ingrid J. Apel[2], Cynthia Wang[2], Victoria Zhixuan Zeng[2], Yunhui Cheng[2], Shuqin Li[2], George (Xiaoju) Wang[2,4,5,*], Arul M. Chinnaiyan[2,4,5,6,7,*], Ke Ding[1,3,8,9,*]

[1]International Cooperative Laboratory of Traditional Chinese Medicine Modernization and Innovative Drug Discovery of Chinese Ministry of Education (MOE), Guangzhou City Key Laboratory of Precision Chemical Drug Development, College of Pharmacy, Jinan University, 855 Xingye Avenue East, Guangzhou 511400, People's Republic of China

[2]Michigan Center for Translational Pathology, University of Michigan, Ann Arbor, MI 48109, USA.

[3]State Key Laboratory of Bioorganic and Natural Products Chemistry, Shanghai Institute of Organic Chemistry, Chinese Academy of Sciences, #345 Ling Ling Road, Shanghai 200032, People's Republic of China

[4]Department of Pathology, University of Michigan, Ann Arbor, MI 48109, USA.

[5]Department of Computational Medicine and Bioinformatics, University of Michigan, Ann Arbor, MI 48109, USA.

[6]Howard Hughes Medical Institute, University of Michigan, Ann Arbor, MI, USA.

[7]Department of Urology, University of Michigan, Ann Arbor, MI 48109, USA.

[8]Institute of Basic Medicine and Cancer (IBMC), Chinese Academy of Sciences, Hangzhou, Zhejiang 310022, People's Republic of China

[9]The First Affiliated Hospital (Huaqiao Hospital), Jinan University, 601 Huangpu Avenue West, Guangzhou 510632, China

Corresponding Authors

*Email:xiaojuw@med.umich.edu (X.W.)

*Email: arul@med.umich.edu (A.M.C)

*Email: dingke@jnu.edu.cn; Tel: +86-20-85221523; Fax: +86-20-85224766 (K.D.)

Table of Contents

Figure S1. .... S3

Figure S2. .... S3

Figure S3. .... S4

Figure S4. .... S4

Figure S5. .... S5

Table S1 .... S5

Table S2 .... S6-S7

Synthesis of Intermediate Compounds 13a-13o, 16a-16j and 15. .... S7-S15

The ^{1}H NMR, ^{13}C NMR, HRMS and HPLC traces of Final Compounds. S16-S69

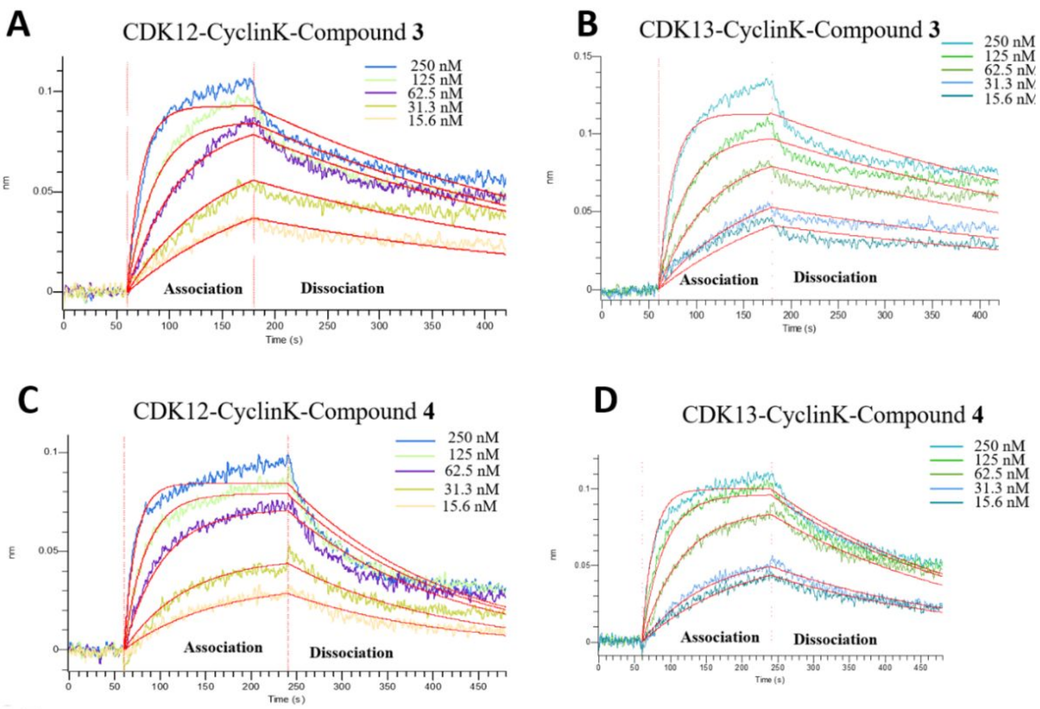

E

Compds	CDK12			CDK13		
	K_d (nM)	K_{on} (1/Ms)	K_{off} (1/s)	K_d (nM)	K_{on} (1/Ms)	K_{off} (1/s)
3	16.2	2.07E+05	3.35E-03	8.6	2.18E+05	1.87E-03
4	27.3	2.27E+05	6.17E-03	17.0	2.02E+05	3.44E-03

Figure S1. Determination of K_d, K_{on}, and K_{off} values for compounds 3 and 4 using an Octet RED System. (A) Association and dissociation kinetic curves of 3 with CDK12-CyclinK. (B) Association and dissociation kinetic curves of 3 with CDK13-CyclinK. (C) Association and dissociation kinetic curves of 4 with CDK12-CyclinK. (D) Association and dissociation kinetic curves of 4 with CDK13-CyclinK. (E) Kinetic parameters of compounds 3 and 4 with CDK12 and CDK13.

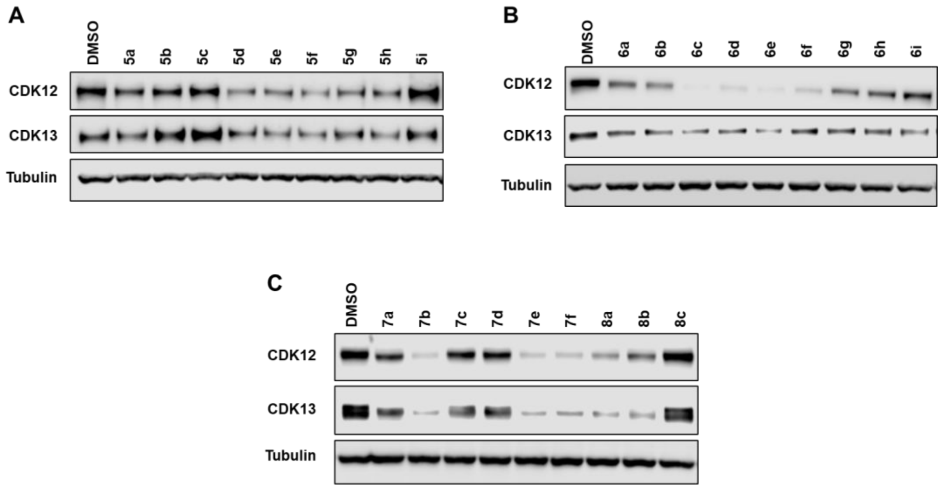

Figure S2. Screening and characterization of potent CDK12/13 degraders. (A, B, C) CDK12 and CDK13 levels in MDA-MB-231 cells upon 15 h PROTACs treatment at 1 μM.

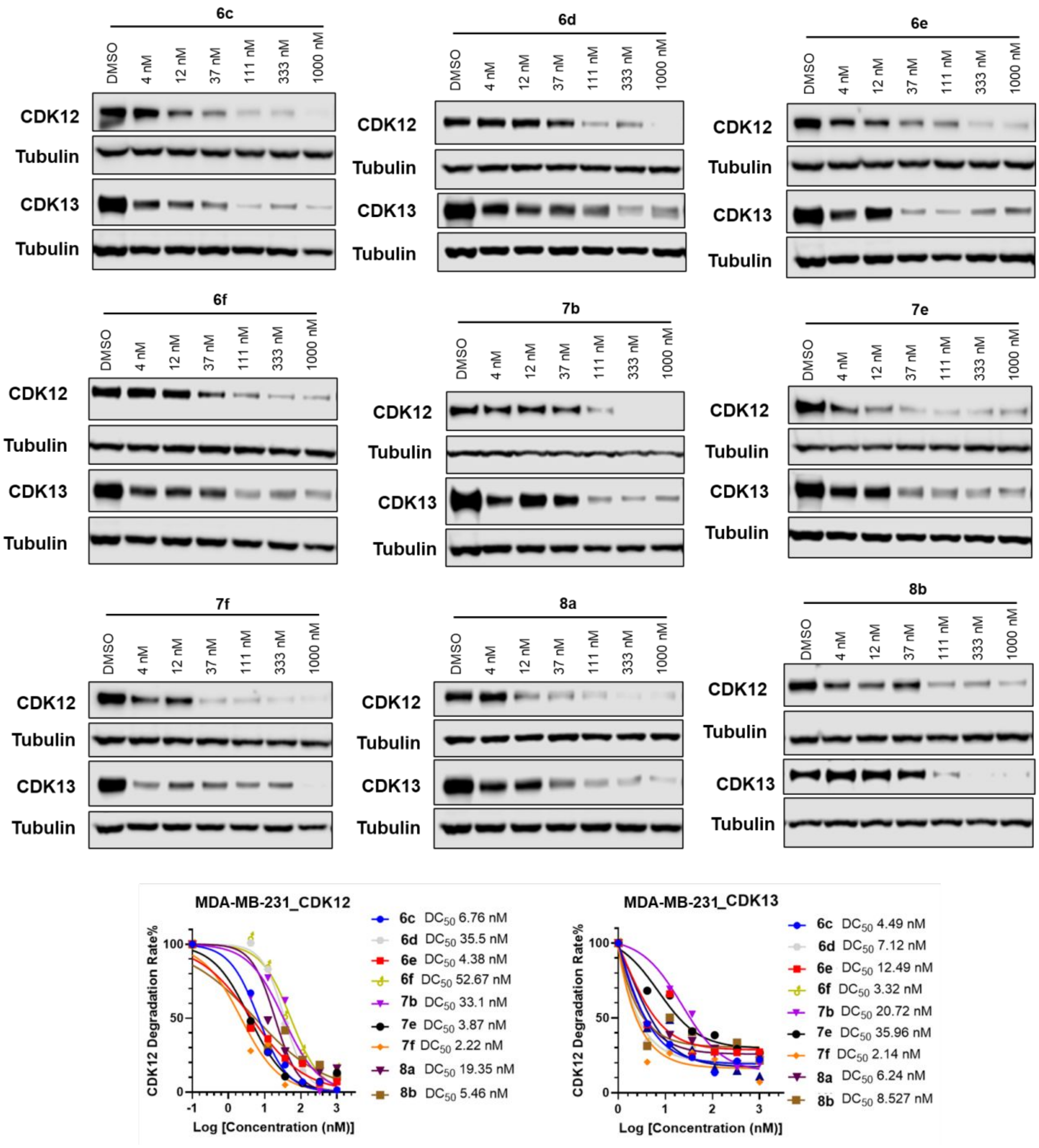

Figure S3. Western blotting analysis of CDK12, CDK13 proteins following 15 h treatment with the indicated concentrations of PROTACs in MDA-MB-231 cells.

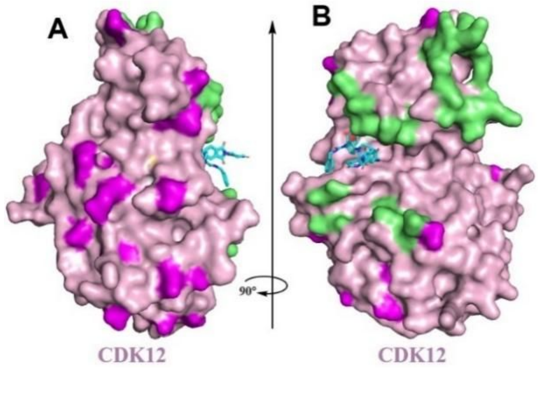

Figure S4. Non-conserved residues in the kinase domains of CDK12 and CDK13 are highlighted as purple, PPI interfaces between CDK12 and CRBN are highlighted as green. A and B are approximately a 90° rotation from each other.

Table S1. Pharmacokinetic Profiles of Compound 7f in Rats

parameters	**7f**		**7f**	
	oral (20 mg/kg)	iv (10 mg/kg)	ip (20mg/kg)	iv (2.5 mg/kg)
$T_{1/2}$ (h)	NA	5.28	10.85	5.8
T_{max} (h)	5.33	0.08	2.17	0.08
C_{max} (ng/mL)	7.73	19892.4	24.79	1498.5
AUC (0-t) (h*ng/mL)	21.83	7193.3	284.8	383.9
AUC ($_{0-\infty}$) (h*ng/mL)	NA	7242.7	318.5	391.55
CL (mL/h/kg)		1406.5		6495.4
F%	0.15		10.63	

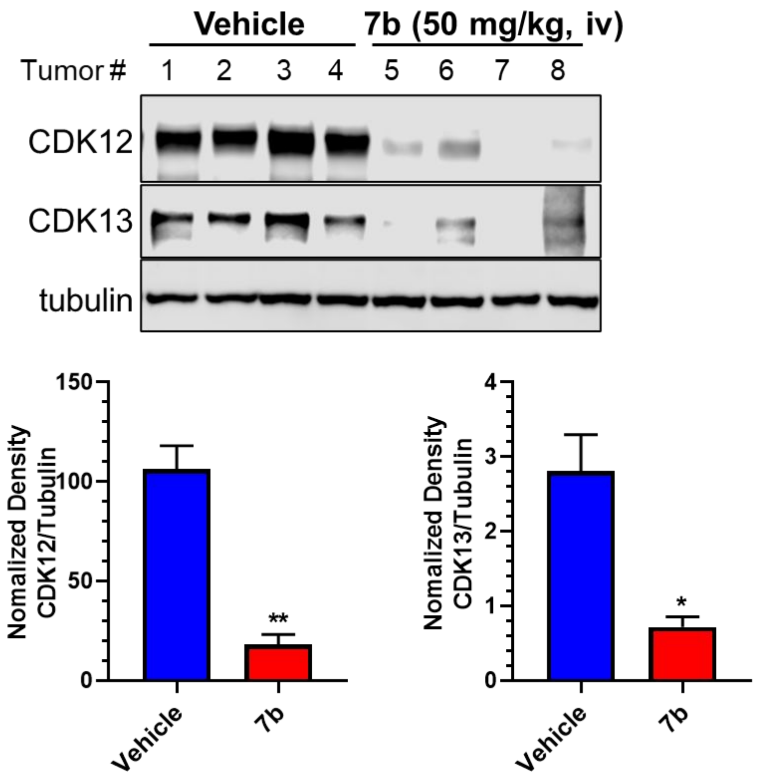

Figure S5. Preliminary pharmacodynamic study of **7b** in MDA-MB-231 xenografted mouse model.

Table S2. Top increased proteins after treatment with **7f** in MFM223 cells.

Gene symbol	description	$log_2(FC)$	-log(p-value)	Significance
A2M	Alpha-2-Macroglobulin	0.63	17.00	up
ALB	Albumin	0.64	17.00	up
CPNE4	Copine 4	0.64	9.50	up
C3	complement component 3	0.65	9.26	up
TMSB4X	Thymosin Beta 4 X-Linked	0.66	17.00	up
AFP	alpha-fetoprotein	0.66	12.00	up
VEZF1	Vascular Endothelial Zinc Finger 1	0.68	6.25	up
RRAGB	Ras-related GTP-binding protein B	0.69	7.38	up
SSBP1	Single-stranded DNA-binding protein	0.7	17.00	up
AHSG	Alpha-2-HS-glycoprotein	0.75	17.00	up
CNBP	CCHC-type zinc finger nucleic acid binding protein	0.8	14.06	up
UCKL1	Uridine-cytidine kinase-like 1	0.85	7.78	up
PML	Protein PML	0.85	6.64	up
TMSB10	Thymosin beta-10	0.86	15.65	up
GC	Vitamin D-binding protein	0.89	17.00	up
NID1	Nidogen-1	0.95	12.01	up
COMP	Cartilage oligomeric matrix protein	0.95	9.46	up
TSPAN31	Tetraspanin-31	1.08	9.08	up
NISCH	Nischarin	1.11	12.18	up
SS18	Protein SSXT	1.25	8.09	up
JAK1	Tyrosine-protein kinase	1.3	7.84	up
SNRPF	Small nuclear ribonucleoprotein F	1.31	17.00	up
MYO1F	Unconventional myosin-If	1.32	8.84	up
WDR54	WD repeat-containing protein 54	1.34	6.51	up
LTF	Lactotransferrin	1.46	17.00	up
MAZ	Myc-associated zinc finger protein	1.55	8.98	up
CLIP2	CAP-Gly domain-containing linker protein 2	1.6	11.51	up
ELOF1	Transcription elongation factor 1 homolog	1.69	17.00	up
RNF126	E3 ubiquitin-protein ligase	1.83	13.10	up
MSN	Moesin	1.96	13.50	up

Scheme S1. Synthesis of Intermediate Compounds 13a-o, 16a-j and 15.

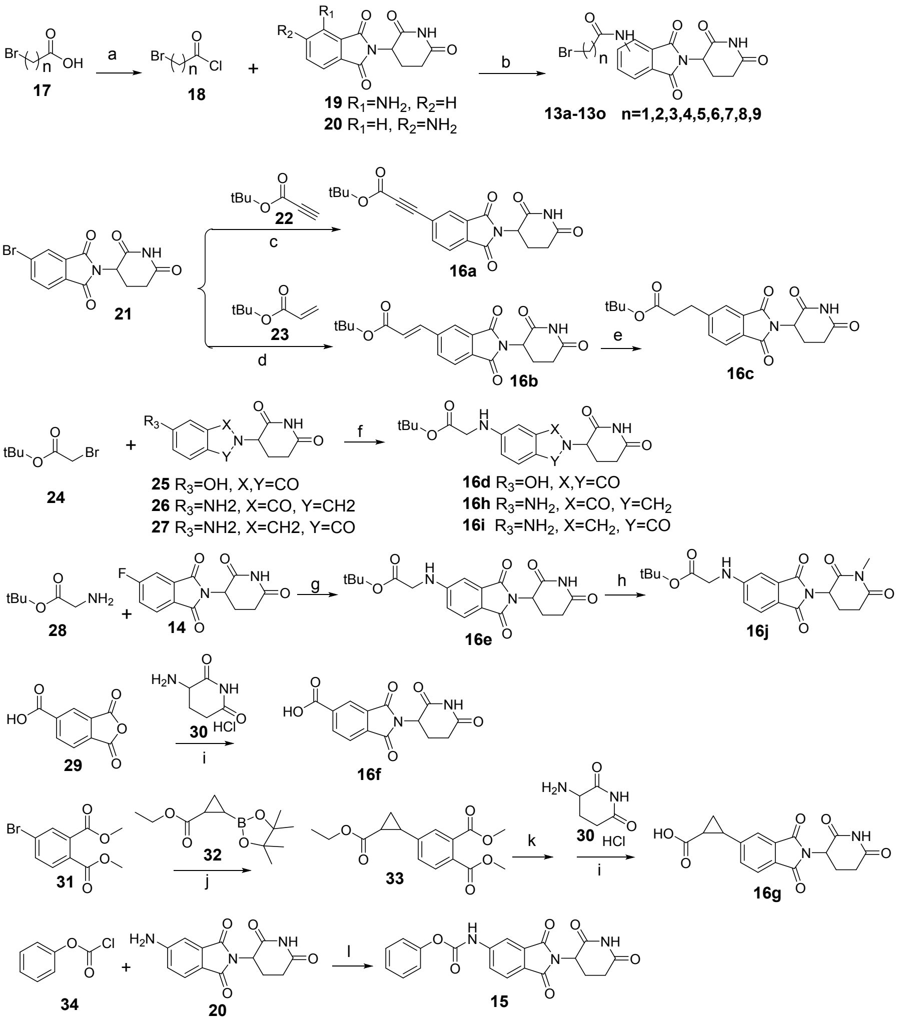

Reagents and conditions: (a) Oxalyl chloride, dry DCM, rt, 3 h; (b) THF, reflux, 5 h, 75-92%; (c) DIPEA, $PdCl_2(PPh_3)_2$, CuI, DMF, 100 °C, overnight, 65%; (d) DIPEA, $Pd(OAc)_2$, $P(ph)_3$, DMF, 100 °C, overnight, 79%; (e) Pd/C, H_2, MeOH, rt, 4 h, 75%; (f) DMF, $KHCO_3$, 90 °C, overnight, 70-90%; (g) DMSO, DIPEA, 120 °C, 8 h, 63%; (h) CH_3I, K_2CO_3, DMF, 0 °C to 50 °C, 5 h, 34%; (i) AcOH, KOAcO, 120 °C, 7 h, 52%; (j) K_3PO_4, $Pd(OAc)_2$, toluene/H_2O (7:3), tricyclohexyl phosphine, 100 °C, 6 h, 28%; (k) LiOH, THF/H2O/MeOH (10:5:1); (l) DIPEA, THF, rt to 75 °C, 5 h, 72%.

The synthetic procedures and characterization data of **13a-13o**, **16a-16j** and **15**.

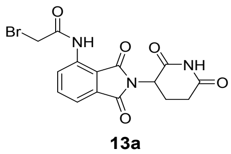

13a

2-bromo-N-(2-(2,6-dioxopiperidin-3-yl)-1,3-dioxoisoindolin-4-yl)acetamide (13a)

Step 1. To a solution of bromoacetic acid **17** (1 g, 7.2 mmol) in anhydrous DCM (20 mL) at 0 °C was added oxalyl chloride (1.1 g, 8.6 mmol) and DMF (30 μL). The reaction was then warm to room temperature and stirred for an additional 3 h. The reaction mixture was then concentrated to dryness under reduced pressure.

Step 2. The resultant crude material form step 1 was dissolved in anhydrous THF (20 mL), pomalidomide (1.64 g, 6 mmol) added and the reaction mixture was stirred for 5 h at 75 °C. The reaction mixture was then diluted with EtOAc/water (1:4, 200 mL), the layers separated, and the aqueous extracted with EtOAc (3×40 mL). The combined organic layers were dried over MgSO4, filtered, and the solvent removed under reduced pressure. The crude material was purified by silica column chromatography to give the title compound as a white solid (1.9 g, yield 80%). ^{1}H NMR (400 MHz, DMSO-d_6) δ 11.16 (s, 1H), 10.31 & 10.25 (2s, 1H), 8.54 & 8.47 (2d, *J* = 8.4 Hz, 1H), 7.89 &7.88 (2t, *J* = 8Hz, 1H), 7.68 (d, *J* = 7.3 Hz, 1H), 5.17 (dd, *J* = 12.8, 5.4 Hz, 1H), 4.54 & 4.34 (2s, 2H), 2.96 – 2.83 (m, 1H), 2.68 – 2.52 (m, 2H), 2.14 – 2.04 (m, 1H). MS (ESI), *m/z*: 391.6[M-H]$^-$.

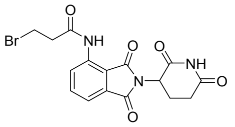

13b

3-bromo-N-(2-(2,6-dioxopiperidin-3-yl)-1,3-dioxoisoindolin-4-yl)propanamide (13b)

Compound **13b** was synthesized by following a similar procedure as that of **13a**. ^{1}H NMR (400 MHz, DMSO-d_6) δ 11.15 (s, 1H), 9.89 (s, 1H), 8.43 (d, *J* = 8.3 Hz, 1H), 7.85 (t, *J* = 7.9 Hz, 1H), 7.65 (d, *J* = 7.3 Hz, 1H), 5.15 (dd, *J* = 12.7, 5.4 Hz, 1H), 3.90 & 3.74 (2t, *J* = 6.3 Hz, 2H), 3.13 & 3.00 (t, *J* = 6.2 Hz, 2H), 2.96 – 2.84 (m, 1H), 2.66 – 2.52 (m, 2H), 2.12 – 2.04 (m, 1H). MS (ESI), *m/z*: 405.8[M-H]$^-$.

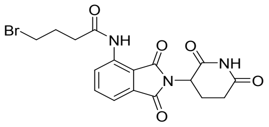

13c

4-bromo-N-(2-(2,6-dioxopiperidin-3-yl)-1,3-dioxoisoindolin-4-yl)butanamide (13c)

Compound **13c** was synthesized by following a similar procedure as that of **13a**. ^{1}H NMR (400 MHz, DMSO-d_6) δ 11.15 (s, 1H), 9.81 (s, 1H), 8.40 (d, *J* = 8.4 Hz, 1H), 7.83 (t, *J* = 7.9 Hz, 1H), 7.63 (d, *J* = 7.3 Hz, 1H), 5.15 (dd, *J* = 12.7, 5.4 Hz, 1H), 3.62 (t, *J* = 6.6 Hz, 2H), 2.96 – 2.83 (m, 1H), 2.68 – 2.52 (m, 4H), 2.16 (p, *J* = 6.9 Hz, 2H), 2.13 – 2.02 (m, 1H). MS (ESI), *m/z*: 419.8[M-H]$^-$.

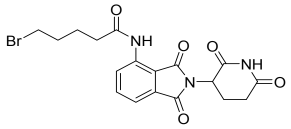

13d

5-bromo-N-(2-(2,6-dioxopiperidin-3-yl)-1,3-dioxoisoindolin-4-yl)pentanamide (13d)

Compound **13d** was synthesized by following a similar procedure as that of **13a**. ^{1}H NMR (400 MHz, DMSO-d_6) δ 11.15 (s, 1H), 9.72 (s, 1H), 8.46 (d, *J* = 8.4 Hz, 1H), 7.83 (dd, *J* = 8.4, 7.3 Hz, 1H), 7.62 (d, *J* = 6.6 Hz, 1H), 5.15 (dd, *J* = 12.7, 5.4 Hz, 1H), 3.58 (t, *J* = 6.6 Hz, 2H), 2.96 – 2.83 (m, 1H), 2.66 – 2.51 (m, 3H), 2.13 – 2.02 (m, 1H), 1.93 – 1.84 (m, 2H), 1.80 – 1.70 (m, 2H). MS (ESI), *m/z*: 433.8[M-H]$^-$.

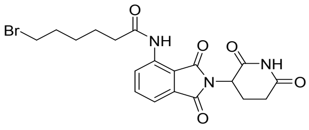

13e

6-bromo-N-(2-(2,6-dioxopiperidin-3-yl)-1,3-dioxoisoindolin-4-yl)hexanamide (13e)

Compound **13e** was synthesized by following a similar procedure as that of **13a**. 1H NMR (400 MHz, DMSO-d_6) δ 11.15 (s, 1H), 9.71 (s, 1H), 8.47 (d, *J* = 8.4 Hz, 1H), 7.83 (dd, *J* = 8.4, 7.3 Hz, 1H), 7.62 (dd, *J* = 7.3, 0.8 Hz, 1H), 5.15 (dd, *J* = 12.7, 5.4 Hz, 1H), 3.54 (t, *J* = 6.7 Hz, 2H), 2.96 – 2.83 (m, 1H), 2.66 – 2.52 (m, 2H), 2.50 – 2.46 (m, 2H), 2.11 – 2.02 (m, 1H), 1.84 (p, *J* = 7.6 Hz, 2H), 1.66 (p, *J* = 7.5 Hz, 2H), 1.50 – 1.41 (m, 2H). MS (ESI), m/z: 447.9[M-H]-.

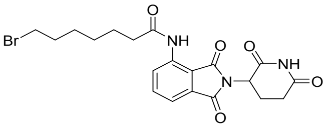

13f

7-bromo-N-(2-(2,6-dioxopiperidin-3-yl)-1,3-dioxoisoindolin-4-yl)heptanamide (13f)

Compound **13f** was synthesized by following a similar procedure as that of **13a**. ^{1}H NMR (400 MHz, DMSO-d_6) δ 11.15 (s, 1H), 9.69 (s, 1H), 8.47 (d, *J* = 8.4 Hz, 1H), 7.87 – 7.78 (m, 1H), 7.61 (d, *J* = 7.3 Hz, 1H), 5.15 (dd, *J* = 12.8, 5.4 Hz, 1H), 3.53 (t, *J* = 6.7 Hz, 2H), 2.96 – 2.83 (m, 1H), 2.67 – 2.52 (m, 2H), 2.47 (t, *J* = 7.4 Hz, 2H), 2.11 – 2.02 (m, 1H), 1.81 (p, *J* = 6.8 Hz, 2H), 1.63 (p, *J* = 7.4 Hz, 2H), 1.47 – 1.31 (m, 4H). MS (ESI), *m/z*: 461.8[M-H]$^-$.

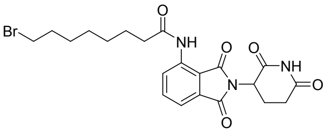

13g

8-bromo-N-(2-(2,6-dioxopiperidin-3-yl)-1,3-dioxoisoindolin-4-yl)octanamide (13g)

Compound **13g** was synthesized by following a similar procedure as that of **13a**. ^{1}H NMR (400 MHz, DMSO-d_6) δ 11.15 (s, 1H), 9.69 (s, 1H), 8.47 (d, *J* = 8.4 Hz, 1H), 7.83 (dd, *J* = 8.4, 7.4 Hz, 1H), 7.61 (d, *J* = 7.3 Hz, 1H), 5.15 (dd, *J* = 12.7, 5.4 Hz, 1H), 3.53 (t, *J* = 6.7 Hz, 2H), 2.96 – 2.83 (m, 1H), 2.66 – 2.52 (m, 2H), 2.47 (t, *J* = 7.4 Hz, 2H), 2.12 – 2.01 (m, 1H), 1.80 (p, *J* = 6.8 Hz, 2H), 1.63 (p, *J* = 7.1 Hz, 2H), 1.43 – 1.36 (m, 2H), 1.36 – 1.28 (m, 4H). MS (ESI), *m/z*: 475.8[M-H]$^-$.

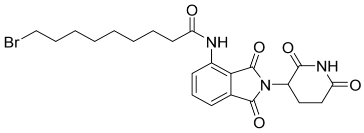

13h

9-bromo-N-(2-(2,6-dioxopiperidin-3-yl)-1,3-dioxoisoindolin-4-yl)nonanamide (13h)

Compound **13h** was synthesized by following a similar procedure as that of **13a**. ^{1}H NMR (400 MHz, DMSO-d_6) δ 11.15 (s, 1H), 9.68 (s, 1H), 8.48 (d, *J* = 8.4 Hz, 1H), 7.83 (dd, *J* = 8.4, 7.3 Hz, 1H), 7.61 (d, *J* = 7.2 Hz, 1H), 5.15 (dd, *J* = 12.7, 5.4 Hz, 1H), 3.51 (t, *J* = 6.7 Hz, 2H), 2.96 – 2.84 (m, 1H), 2.68 – 2.52 (m, 2H), 2.46 (t, *J* = 7.4 Hz, 2H), 2.11 – 2.03 (m, 1H), 1.79 (p, *J* = 6.9 Hz, 2H), 1.63 (p, *J* = 7.7, 7.2 Hz, 2H), 1.34 (m, 8H). MS (ESI), *m/z*: 489.8[M-H]$^-$.

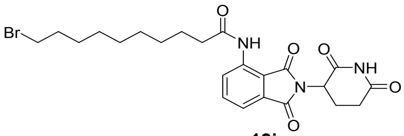

13i

10-bromo-N-(2-(2,6-dioxopiperidin-3-yl)-1,3-dioxoisoindolin-4-yl)decanamide (13i)

Compound **13i** was synthesized by following a similar procedure as that of **13a**. ^{1}H NMR (400 MHz, DMSO-d_6) δ 11.15 (s, 1H), 9.69 (s, 1H), 8.47 (d, *J* = 8.4 Hz, 1H), 7.83 (dd, *J* = 8.4, 7.4 Hz, 1H), 7.61 (d, *J* = 7.3 Hz, 1H), 5.15 (dd, *J* = 12.7, 5.4 Hz, 1H), 3.51 (t, *J* = 6.7 Hz, 2H), 2.96 – 2.83 (m, 1H), 2.66 – 2.52 (m, 2H), 2.46 (t, *J* = 7.4 Hz, 2H), 2.11 – 2.02 (m, 1H), 1.78 (p, *J* = 6.8 Hz, 2H), 1.62 (p, *J* = 7.2 Hz, 2H), 1.42 – 1.23 (m, 10H). MS (ESI), *m/z*: 503.9[M-H]$^-$.

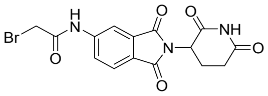

13j

2-bromo-N-(2-(2,6-dioxopiperidin-3-yl)-1,3-dioxoisoindolin-5-yl)acetamide (13j)

Compound **13j** was synthesized by following a similar procedure as that of **13a**. ^{1}H NMR (400 MHz, DMSO-d_6) δ 11.12 (s, 1H), 11.05 (s, 1H), 8.22 (s, 1H), 7.91 (s, 2H), 5.14 (dd, *J* = 12.8, 5.4 Hz, 1H), 4.13 (s, 2H), 2.96 – 2.83 (m, 1H), 2.65 – 2.52 (m, 2H), 2.11 – 2.02 (m, 1H). HRMS (ESI) for $C_{15}H_{12}BrN_3O_5$ [M+H]$^+$, calcd: 394.0033, found: 394.0039.

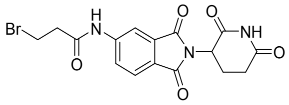

13k

3-bromo-N-(2-(2,6-dioxopiperidin-3-yl)-1,3-dioxoisoindolin-5-yl)propanamide (13k)

Compound **13k** was synthesized by following a similar procedure as that of **13a**. ^{1}H NMR (400 MHz, DMSO-d_6) δ 11.12 (s, 1H), 10.74 (s, 1H), 8.25 (s, 1H), 7.94 – 7.87 (m, 2H), 5.13 (dd, *J* = 12.9, 5.4 Hz, 1H), 3.76 (t, *J* = 6.3 Hz, 2H), 3.06 (t, *J* = 6.3 Hz, 2H), 2.95 – 2.82 (m, 1H), 2.66 – 2.52 (m, 2H), 2.12 – 2.00 (m, 1H). HRMS (ESI) for $C_{16}H_{14}BrN_3O_5$ [M+H]$^+$, calcd: 408.0190, found: 408.0191.

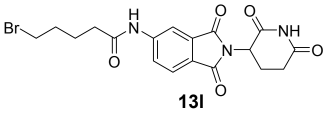

13l

5-bromo-N-(2-(2,6-dioxopiperidin-3-yl)-1,3-dioxoisoindolin-5-yl)pentanamide (13l)

Compound **13l** was synthesized by following a similar procedure as that of **13a**. ^{1}H NMR (400 MHz, DMSO-d_6) δ 11.12 (s, 1H), 10.59 (s, 1H), 8.25 (d, *J* = 1.7 Hz, 1H), 7.93 – 7.84 (m, 2H), 5.12 (dd, *J* = 12.9, 5.4 Hz, 1H), 3.57 (t, *J* = 6.5 Hz, 2H), 2.96 – 2.83 (m, 1H), 2.64 – 2.53 (m, 2H), 2.45 (t, *J* = 7.3 Hz, 2H), 2.11 – 2.02 (m, 1H), 1.87 (p, *J* = 6.6 Hz, 2H), 1.74 (p, *J* = 7.5 Hz, 2H). HRMS (ESI) for $C_{18}H_{18}BrN_3O_5$ [M+H]$^+$, calcd: 436.0503, found: 436.0501.

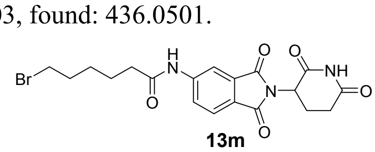

13m

6-bromo-N-(2-(2,6-dioxopiperidin-3-yl)-1,3-dioxoisoindolin-5-yl)octanamide (13m)

Compound **13m** was synthesized by following a similar procedure as that of **13a**. ^{1}H NMR (400 MHz, DMSO-d_6) δ 11.12 (s, 1H), 10.68 (s, 1H), 8.27 (d, *J* = 1.7 Hz, 1H), 7.93 (dd, *J* = 8.2, 1.9 Hz, 1H), 7.86 (d, *J* = 8.2 Hz, 1H), 5.12 (dd, *J* = 12.9, 5.4 Hz, 1H), 3.54 (t, *J* = 6.6 Hz, 2H), 2.96 – 2.83 (m, 1H), 2.65 – 2.52 (m, 2H), 2.42 (t, *J* = 7.4 Hz, 2H), 2.11 – 2.02 (m, 1H), 1.83 (p, *J* = 6.8 Hz, 2H), 1.64 (p, *J* = 7.5 Hz, 2H), 1.44 (tt, *J* = 9.7, 6.1 Hz, 2H). MS (ESI), *m/z*: 447.6[M-H]$^-$.

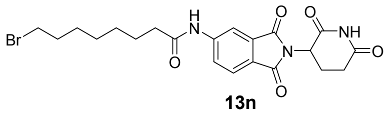

13n

8-bromo-N-(2-(2,6-dioxopiperidin-3-yl)-1,3-dioxoisoindolin-5-yl)octanamide (13n)

Compound **13n** was synthesized by following a similar procedure as that of **13a**. ^{1}H NMR (400 MHz, DMSO-d_6) δ 11.11 (s, 1H), 10.60 (s, 1H), 8.27 (s, 1H), 7.93 (d, *J* = 8.3 Hz, 1H), 7.84 (d, *J* = 8.2 Hz, 1H), 5.11 (dd, *J* = 12.9, 5.4 Hz, 1H), 3.50 (t, *J* = 6.7 Hz, 2H), 2.96 – 2.82 (m, 1H), 2.65 – 2.52 (m, 2H), 2.40 (t, *J* = 7.4 Hz, 2H), 2.10 – 2.00 (m, 1H), 1.83 – 1.66 (m, 2H), 1.59 (q, *J* = 7.1 Hz, 2H), 1.38 (q, *J* = 7.4, 6.1 Hz, 2H), 1.33 – 1.22 (m, 4H). MS (ESI), *m/z*: 475.6[M-H]$^-$.

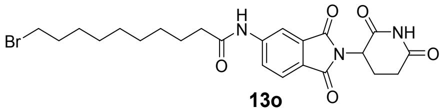

13o

10-bromo-N-(2-(2,6-dioxopiperidin-3-yl)-1,3-dioxoisoindolin-5-yl)decanamide (13o)

Compound **13o** was synthesized by following a similar procedure as that of **13a**. ^{1}H NMR (400 MHz, DMSO-d_6) δ 11.11 (s, 1H), 10.58 (s, 1H), 8.26 (d, *J* = 1.7 Hz, 1H), 7.91 (dd, *J* = 8.3, 1.8 Hz, 1H), 7.85 (d, *J* = 8.2 Hz, 1H), 5.11 (dd, *J* = 12.8, 5.4 Hz, 1H), 2.95 – 2.82 (m, 1H), 2.66 – 2.53 (m, 2H), 2.39 (t, *J* = 7.4 Hz, 2H), 2.10 – 2.00 (m, 1H), 1.77 (p, *J* = 6.9 Hz, 2H), 1.59 (q, *J* = 7.1 Hz, 2H), 1.36 (q, *J* = 7.8, 7.3 Hz, 2H), 1.32 – 1.20 (m, 10H). MS (ESI), *m/z*: 503.7[M-H]$^-$.

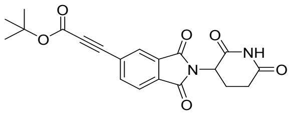

16a

***tert*-butyl 3-(2-(2,6-dioxopiperidin-3-yl)-1,3-dioxoisoindolin-5-yl)propiolate (16a)**

A mixture of 5-bromo-2-(2,6-dioxopiperidin-3-yl)isoindoline-1,3-dione **21** (1.5 g, 4.43 mmol), *tert*-butyl propiolate **22** (1.68 g, 13.3mmol), DIPEA (1.7 g, 13.3 mmol), $PdCl_2(PPh_3)_2$ (155 mg, 0.22 mmol), CuI (67 mg, 0.35 mmol) and anhydrous DMF (50 mL) were added to a 100 mL round-bottom flask. The flask was evacuated and backfilled with argon (3 cycles). After stirring at 100 °C overnight, the mixture was then filtered and the solvent removed under vacuum. The crude material was purified by column chromatography to give **16a** as white solid (1.1 g, yield 65%). ^{1}H NMR (400 MHz, DMSO-d_6) δ 11.16 (s, 1H), 8.17 (s, 1H), 8.14 (dd, *J* = 7.7, 1.4 Hz, 1H), 8.00 (d, *J* = 7.8 Hz, 1H), 5.19 (dd, *J* = 12.8, 5.4 Hz, 1H), 2.96 – 2.84 (m, 1H), 2.66 – 2.51 (m, 2H), 2.12 – 2.03 (m, 1H), 1.51 (s, 9H). MS (ESI), *m/z*: 380.7[M-H]$^-$.

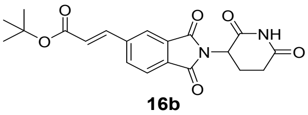

16b

***tert*-butyl (E)-3-(2-(2,6-dioxopiperidin-3-yl)-1,3-dioxoisoindolin-5-yl)acrylate (16b)**

A mixture of 5-bromo-2-(2,6-dioxopiperidin-3-yl)isoindoline-1,3-dione **21** (1 g, 2.95 mmol), *tert*-butyl acrylate **23** (757.3 mg, 5.9 mmol), DIPEA (762.2 mg, 5.9 mmol), $Pd(OAc)_2$ (33.2 mg, 0.15 mmol), $P(ph)_3$ (77.5 mg, 0.3 mmol) and anhydrous DMF (40 mL) were added to a 100 mL round-bottom flask. The flask was evacuated and backfilled with argon (3 cycles). After stirring at 100 °C overnight, the mixture was then filtered and the solvent removed under vacuum. The crude material was purified by column chromatography to give **16b** as white solid (900 mg, yield 79%). ^{1}H NMR (400 MHz, DMSO-d_6) δ 11.14 (s, 1H), 8.30 (s, 1H), 8.19 (d, *J* = 7.8 Hz, 1H), 7.93 (d, *J* = 7.7 Hz, 1H), 7.74 (d, *J* = 16.1 Hz,

1H), 6.85 (d, J = 16.1 Hz, 1H), 5.17 (dd, J = 12.9, 5.4 Hz, 1H), 2.96 – 2.84 (m, 1H), 2.65 – 2.52 (m, 2H), 2.12 – 2.02 (m, 1H), 1.50 (s, 9H). MS (ESI), *m/z*: 382.8[M-H]$^-$.

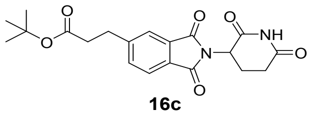

16c

***tert*-butyl 3-(2-(2,6-dioxopiperidin-3-yl)-1,3-dioxoisoindolin-5-yl)propanoate (16c)**

To a solution of the compound **16b** (200 mg, 0.78 mmol) in methanol (10 mL) was added 10% Pd/C (20 mg, 10% w/w) at room temperature. The reaction mixture was stirred under hydrogen balloon for 4 h and then filtered through celite. The filtrate was concentrated to give the crude material which was purified by silica column chromatography to give **16c** (150mg, yield 75%). ^{1}H NMR (400 MHz, DMSO-d_6) δ 11.12 (s, 1H), 7.84 (d, J = 7.7 Hz, 1H), 7.81 (s, 1H), 7.75 (dd, J = 7.7, 1.5 Hz, 1H), 5.14 (dd, J = 12.9, 5.4 Hz, 1H), 3.01 (t, J = 7.3 Hz, 2H), 2.96 – 2.84 (m, 1H), 2.66 – 2.52 (m, 4H), 2.11 – 2.02 (m, 1H), 1.36 (s, 9H). MS (ESI), *m/z*: 384.9[M-H]$^-$.

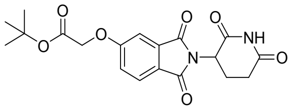

16d

***tert*-butyl 2-((2-(2,6-dioxopiperidin-3-yl)-1,3-dioxoisoindolin-5-yl)oxy)acetate (16d)**

To a solution of 2-(2,6-dioxopiperidin-3-yl)-5-hydroxyisoindoline-1,3-dione **25** (1 g, 3.65 mmol) in 20 mL DMF, $KHCO_3$ (547 mg, 5.47 mmol) and *tert*-butyl 2-bromoacetate **24** (711 mg, 3.65 mmol) were added at room temperature. After stirring at 90 °C overnight, the reaction mixture was then filtered and the solvent removed under reduced pressure. The crude material was purified by column chromatography to afford **16d** as white solid (1.25 g, yield 88%). ^{1}H NMR (400 MHz, DMSO-d_6) δ 11.12 (s, 1H), 7.86 (d, J = 8.3 Hz, 1H), 7.41 (d, J = 2.3 Hz, 1H), 7.36 (dd, J = 8.3, 2.3 Hz, 1H), 5.12 (dd, J = 12.9, 5.4 Hz, 1H), 4.94 (s, 2H), 2.96 – 2.82 (m, 1H), 2.65 – 2.52 (m, 2H), 2.11 – 2.01 (m, 1H). MS (ESI), *m/z*: 386.8[M-H]$^-$.

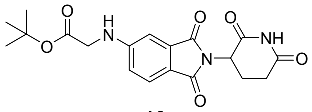

16e

***tert*-butyl (2-(2,6-dioxopiperidin-3-yl)-1,3-dioxoisoindolin-5-yl)glycinate (16e)**

To a solution of 2-(2,6-dioxopiperidin-3-yl)-5-fluoroisoindoline-1,3-dione **14** (250 mg, 0.91 mmol) in 10 mL DMSO, DIPEA (175.1 mg, 1.36 mmol) and *tert*-butyl glycinate **28** (142.5 mg, 1.09 mmol) were added at room temperature. The resulting mixture was stirred at 120 °C for 8 h. The solvent was removed under vacuum to afford crude material which was purified by flash column chromatography to afford **16e** as a yellow solid (220 mg, yield 63%). ^{1}H NMR (400 MHz, DMSO-d_6) δ 11.07 (s, 1H), 7.59 (d, J = 8.3 Hz, 1H), 7.39 (t, J = 6.3 Hz, 1H), 6.97 (s, 1H), 6.88 (dd, J = 8.4, 2.2 Hz, 1H), 5.04 (dd, J = 12.9, 5.4 Hz, 1H), 4.01 (d, J = 6.2 Hz, 2H), 2.96 – 2.82 (m, 1H), 2.62 – 2.52 (m, 2H), 2.05 – 1.96 (m, 1H), 1.43 (s, 9H). MS (ESI), *m/z*: 385.8[M-H]$^-$.

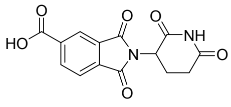

16f

dimethyl 4-(2-(ethoxycarbonyl)cyclopropyl)phthalate (16f)

1,3-dioxo-1,3-dihydroisobenzofuran-5-carboxylic acid **29** (500 mg, 2.6 mmol) was dissolved in acetic acid (25 mL), KOAc (766 mg, 7.8 mmol) and 3-aminopiperidine-2,6-dione hydrochlor ide **30** (1.04 g, 5.2 mmol) were added. The reaction mixture was stirred at 120 °C for 7 h an d then filtered through celite. The solvent removed under reduced pressure, the resulting solid was triturated with EtOAc and then filtered. The residues were washed with water and EtOAc to afford the desired product **16f** as grey solid (408 mg, yield 52%). ^{1}H NMR (400 MHz, D MSO-d_6) δ 11.14 (s, 1H), 8.40 (dd, *J* = 7.7, 1.3 Hz, 1H), 8.28 (s, 1H), 8.04 (d, *J* = 7.8 Hz, 1H), 5.20 (dd, *J* = 12.8, 5.4 Hz, 1H), 2.97 – 284 (m, 1H), 2.67 – 2.52 (m, 2H), 2.14 – 2.04 (m, 1H). MS (ESI), *m/z*: 301.0[M-H]$^-$.

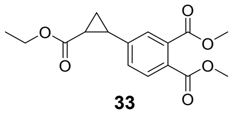

33

dimethyl 4-(2-(ethoxycarbonyl)cyclopropyl)phthalate (33)

To a solution of dimethyl 4-bromophthalate **31** (1.36 g, 5 mol) in toluene/H_2O (10:1, 44mL) was added ethyl 2-(4,4,5,5-tetramethyl-1,3,2-dioxaborolan-2-yl)cyclopropane-1-carboxylate **32** (1 g, 4.16 mol), K_3PO_4 (1.77 g, 8.3 mmol), tricyclohexyl phosphine (116.6 mg, 0.41 mmol) and $Pd(OAc)_2$ (46.7 mg, 0.21 mmol). The mixture was evacuated and backfilled with argon. After stirring at 100 °C for 6 h, the solvent was removed under vacuum, and the resultant crude residue redissolved in EtOAc (20 mL), which was washed with H_2O (60 mL), the layers separated and the aqueous phase extracted with EtOAc (2 x 20 mL). The combined EtOAc layers were subsequently, dried over $MgSO_4$, and the solvent removed under reduced pressure. The crude material was purified by silica column chromatography to afford the title compound as a clear oil (350 mg, yield 28%). ^{1}H NMR (400 MHz, Chloroform-*d*) δ 7.67 (d, *J* = 8.0 Hz, 1H), 7.62 (s, 1H), 7.44 (d, *J* = 8.0 Hz, 1H), 3.96 – 3.86 (m, 8H), 2.59 (q, *J* = 8.5 Hz, 1H), 2.16 (q, *J* = 8.4, 7.8 Hz, 1H), 1.77 (q, *J* = 6.5 Hz, 1H), 1.42 (q, *J* = 8.1 Hz, 1H), 1.03 (t, *J* = 7.1 Hz, 3H). MS (ESI), *m/z*: 328.9[M+Na]$^+$.

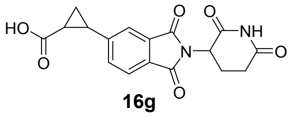

16g

2-(2-(2,6-dioxopiperidin-3-yl)-1,3-dioxoisoindolin-5-yl)cyclopropane-1-carboxylic acid (16g)

Step 1. Lithium hydroxide monohydrate (226 mg, 5.4 mmol) was added to a solution of dimethyl 4-(2-(ethoxycarbonyl)cyclopropyl)phthalate **33** (330 mg, 1.08 mmol) in THF/H2O/MeOH (10:5:1, 16 mL) and stirred at room temperature for overnight. The solvent evaporated under reduced pressure. The resultant crude material was then diluted with 1M HCl (35 mL), the aqueous was subsequently extracted with EtOAc (3×15 mL), and the combined organic layers was concentrated to dryness (260 mg).

Step 2. The crude material from step 1 was dissolved in AcOH (20 mL), Potassium Acetate (317 mg, 3.234 mmol) and 3-aminopiperidine-2,6-dione hydrochloride **30** (265 mg, 1.62 mmol) were added. The reaction mixture was then stirred at 120 °C for 7 h. The solvent removed under reduced pressure, then extracted by EtOAc and washed with H_2O followed by brine. The organic layers were dried over anhydrous Na_2SO_4, filtered and evaporated. The crude material was purified by silica gel column chromatography to give the compound **16g** as white solid (140 mg, yield 39%). ^{1}H NMR (400 MHz, DMSO-d_6) δ 11.12 (s, 1H), 7.82 (d, *J* = 7.6 Hz, 1H), 7.79 – 7.71 (m, 2H), 5.14 (dd, *J* = 13.0, 5.3 Hz, 1H), 2.95 – 2.83 (m, 1H), 2.79 (q, *J* = 8.5 Hz, 1H), 2.65 – 2.53 (m, 2H), 2.15 (q, *J* = 7.6 Hz, 1H), 2.11-2.01 (m, 1H), 1.67 (q, *J* = 6.1 Hz, 1H), 1.43 (q, *J* = 7.7 Hz, 1H). MS (ESI), *m/z*: 340.8[M-H]$^-$.

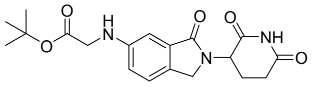

16h

***tert*-butyl (2-(2,6-dioxopiperidin-3-yl)-3-oxoisoindolin-5-yl)glycinate (16h)**

Compound **16h** was synthesized by following a similar procedure as that of **16d**. ^{1}H NMR (400 MHz, DMSO-d_6) δ 10.95 (s, 1H), 7.29 (d, J = 8.2 Hz, 1H), 6.87 (dd, J = 8.3, 2.3 Hz, 1H), 6.78 (d, J = 2.3 Hz, 1H), 6.26 (t, J = 6.4 Hz, 1H), 5.06 (dd, J = 13.2, 5.1 Hz, 1H), 4.28 (d, J = 16.4 Hz, 1H), 4.16 (d, J = 16.4 Hz, 1H), 3.84 (d, J = 6.4 Hz, 2H), 2.97 – 2.82 (m, 1H), 2.65 – 2.54 (m, 1H), 2.44 – 2.27 (m, 1H), 2.05 – 1.90 (m, 1H), 1.47 – 1.35 (s, 9H). MS (ESI), *m/z*: 372.0[M-H]$^-$.

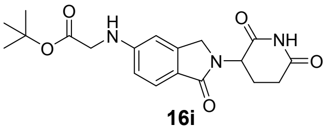

16i

***tert*-butyl (2-(2,6-dioxopiperidin-3-yl)-1-oxoisoindolin-5-yl)glycinate (16i)**

Compound **16i** was synthesized by following a similar procedure as that of **16d**. ^{1}H NMR (400 MHz, DMSO-d_6) δ 10.91 (s, 1H), 7.42 (d, J = 8.3 Hz, 1H), 6.71 – 6.63 (m, 2H), 6.62 (d, J = 1.9 Hz, 1H), 5.02 (dd, J = 13.3, 5.1 Hz, 1H), 4.28 (d, J = 16.7 Hz, 1H), 4.15 (d, J = 16.7 Hz, 1H), 3.87 (d, J = 6.3 Hz, 2H), 2.96 – 2.83 (m, 1H), 2.64 – 2.55 (m, 1H), 2.35 (qd, J = 13.1, 4.4 Hz, 1H), 2.00 – 1.90 (m, 1H), 1.43 (s, 9H). MS (ESI), *m/z*: 372.2[M-H]$^-$.

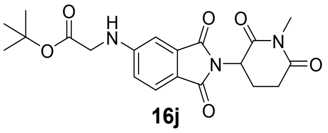

16j

***tert*-butyl (2-(1-methyl-2,6-dioxopiperidin-3-yl)-1,3-dioxoisoindolin-5-yl)glycinate (16j)**

CH_3I (439 mg, 3.1 mmol) was added to a solution of **16e** (400 mg, 1.03 mmol) and K_2CO_3 (213 mg, 1.55 mmol) in DMF (10 mL) at 0 °C. The reaction was subsequently allowed to warm to 50 °C and stirred for 5 h. The reaction mixture was then filtered, and concentrated to dryness under reduced pressure. The resultant crude material was purified by column chromatography to give the title compound **16j** as a white solid (140 mg, yield 34%). ^{1}H NMR (400 MHz, DMSO-d_6) δ 7.60 (d, J = 8.3 Hz, 1H), 7.36 (t, J = 6.2 Hz, 1H), 6.97 (s, 1H), 6.89 (dd, J = 8.4, 2.1 Hz, 1H), 5.11 (dd, J = 13.0, 5.4 Hz, 1H), 4.01 (d, J = 6.2 Hz, 2H), 3.01 (s, 3H), 2.98 – 2.88 (m, 1H), 2.80 – 2.71 (m, 1H), 2.60 – 2.52 (m, 1H), 2.06 – 2.00 (m, 1H), 1.43 (s, 9H). MS (ESI), *m/z*:400.2[M-H]$^-$.

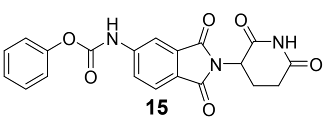

15

phenyl (2-(2,6-dioxopiperidin-3-yl)-1,3-dioxoisoindolin-5-yl)carbamate (15)

To a solution of 5-amino-2-(2,6-dioxopiperidin-3-yl)isoindoline-1,3-dione **20** (300 mg, 1.1 mmol) and DIPEA (170 mg, 1.32 mmol) in THF (20 mL) was slowly added phenyl carbonochloridate **34** (189.4 mg, 1.21 mmol) at room temperature. After stirred at room temperature for 5 mins, then the reaction mixture was stirred for 5 h at 75 °C. The mixture was then filtered and the solvent removed under vacuum. The crude material was purified by column chromatography to give **15** as white solid (311 mg, yield 72%). ^{1}H NMR (400 MHz, DMSO-d_6) δ 11.13 (s, 1H), 11.00 (s, 1H), 8.07 (s, 1H), 7.91 (s, 2H), 7.46 (t, J = 7.9 Hz, 2H), 7.30 (dd, J = 8.1, 6.6 Hz, 3H), 5.14 (dd, J = 12.9, 5.3 Hz, 1H), 2.96 – 2.83 (m, 1H), 2.65 – 2.52 (m, 2H), 2.11 – 2.02 (m, 1H). MS (ESI), *m/z*: 503.7[M-H]$^-$.

The ^{1}H NMR, ^{13}C NMR, HRMS and HPLC traces of Compounds 5a-5i, 6a-i, 7a-f and 8a-c.

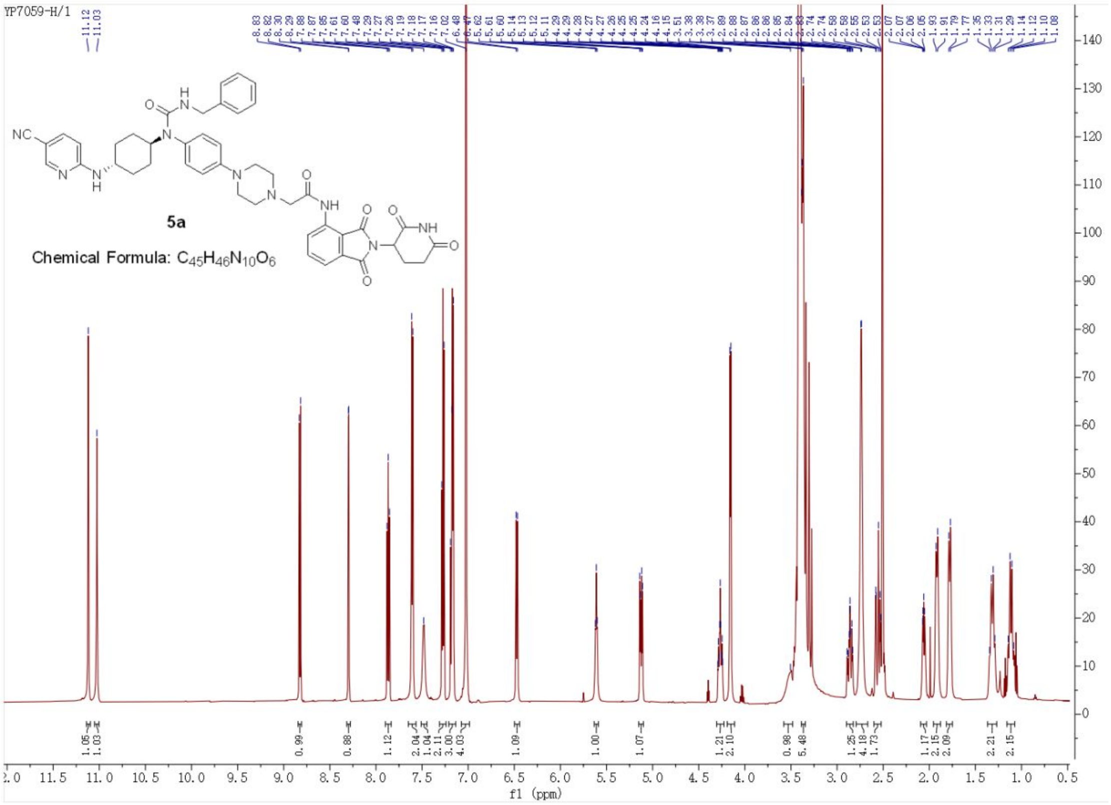

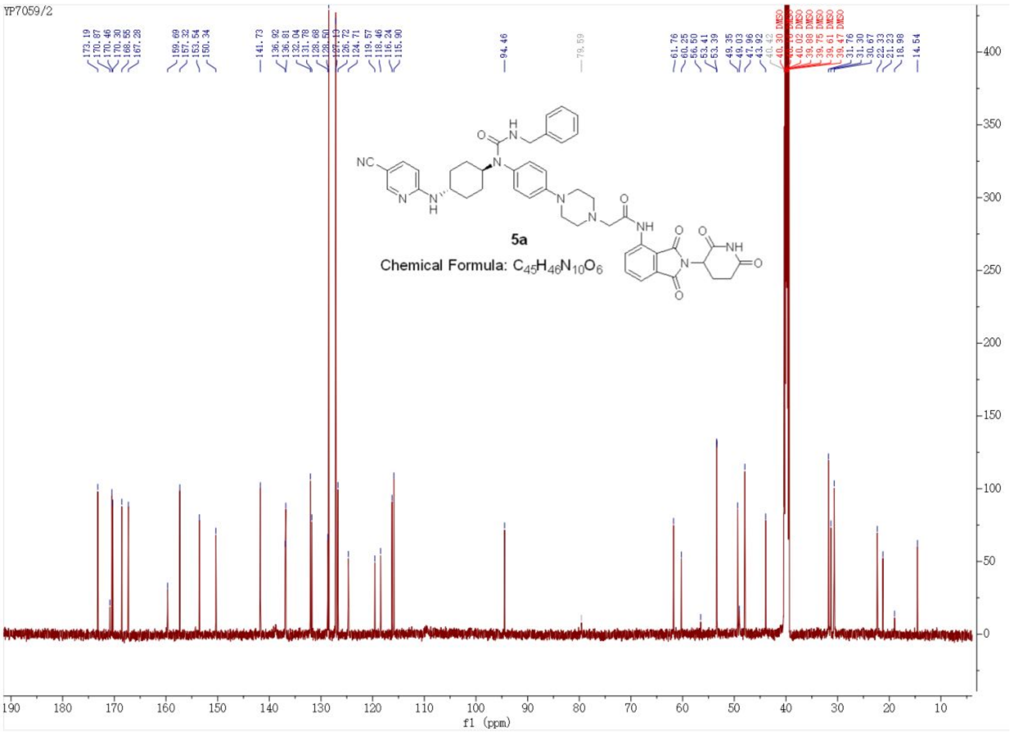

Hit	Formula	m/z	RDB	ppm	MS Rank	MSMS ppm	MSMS Rank	Found
1	C45H46N10O6	823.3675	28.0	-2.7	1			NA/NA

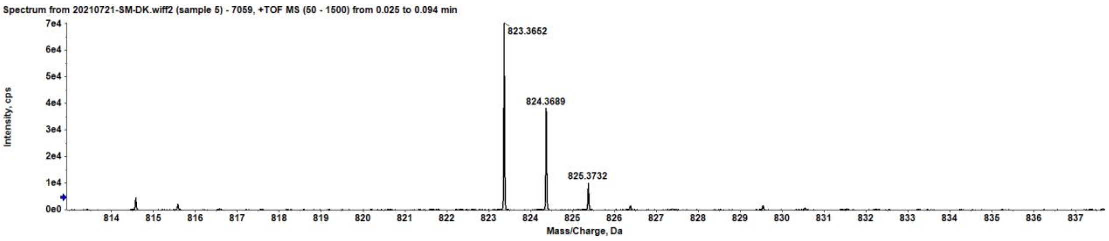


```
Data File E:\DK\ZZ\20210624-ZZ-YJZ-ZFT\YJZ1901DEF_LC 2021-06-24 20-23-22\7059.D
Sample Name: 7059

=====================================================================
Acq. Operator   : 系统                          Seq. Line :  57
Acq. Instrument : 1260LC                         Location :   25
Injection Date  : 26/06/2021 03:09:45                 Inj :   1
                                               Inj Volume : 10.000 µl
Method          : E:\DK\ZZ\20210624-ZZ-YJZ-ZFT\YJZ1901DEF_LC 2021-06-24 20-23-22\75C-25A-
                  30min-1u.M (Sequence Method)
Last changed    : 24/06/2021 20:23:22 by 系统
```

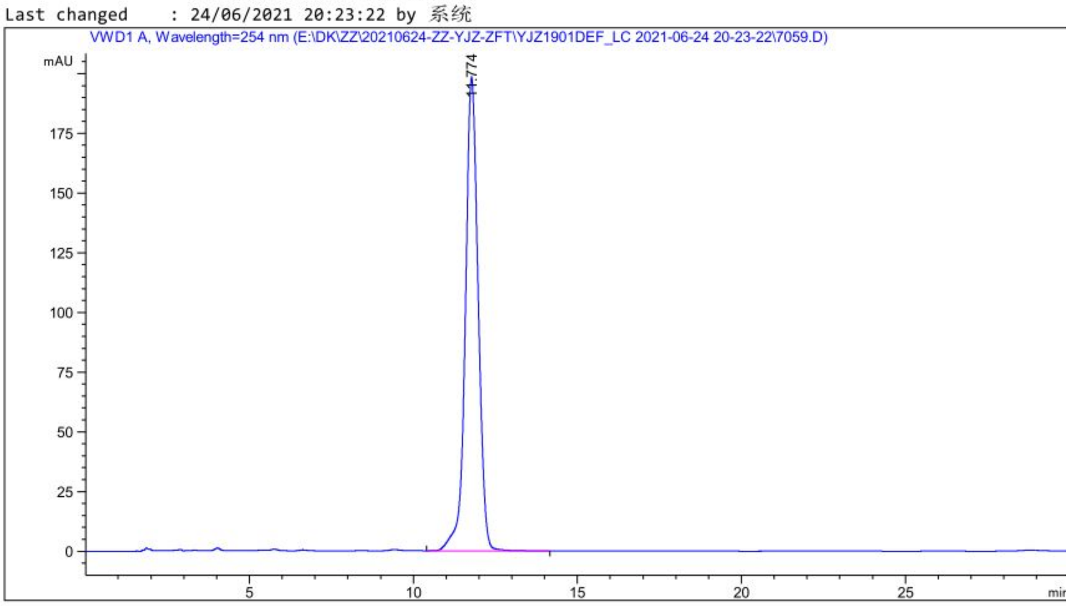


```
=====================================================================
                         Area Percent Report
=====================================================================

Sorted By             :      Signal
Multiplier            :      1.0000
Dilution              :      1.0000
Use Multiplier & Dilution Factor with ISTDs


Signal 1: VWD1 A, Wavelength=254 nm
```

Peak #	RetTime [min]	Type	Width [min]	Area [mAU*s]	Height [mAU]	Area %
1	11.774	BB	0.4125	5399.89014	198.44347	100.0000

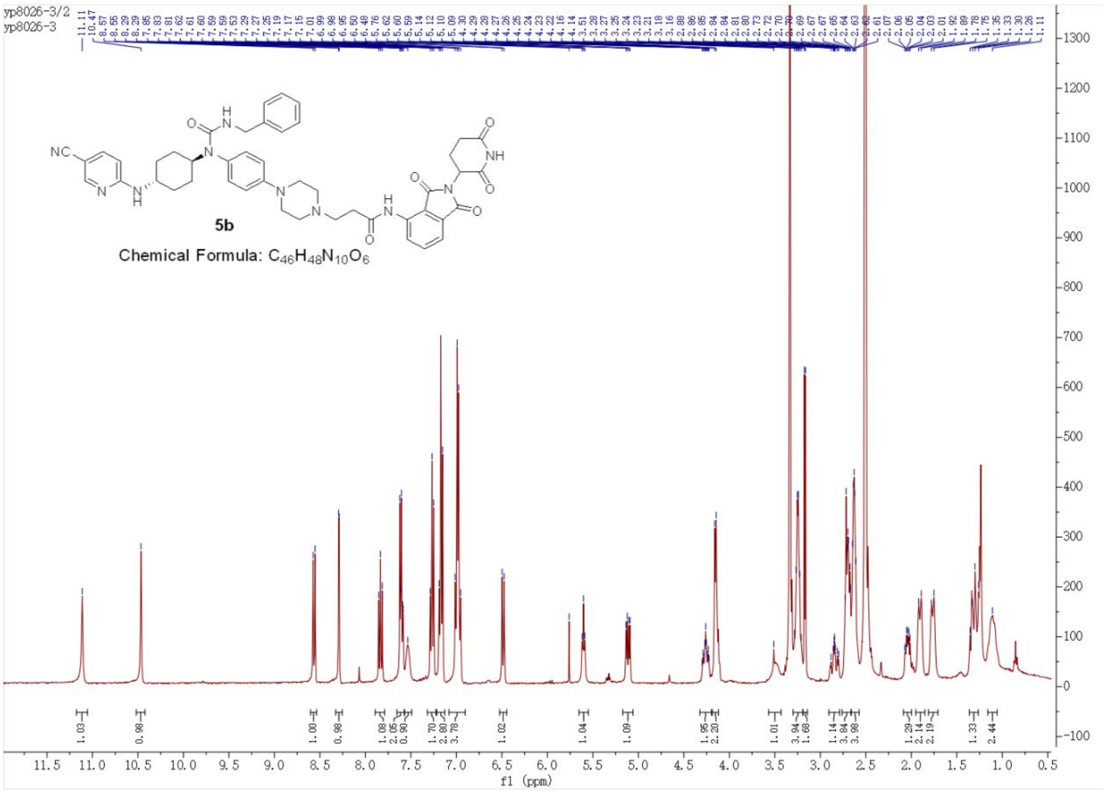

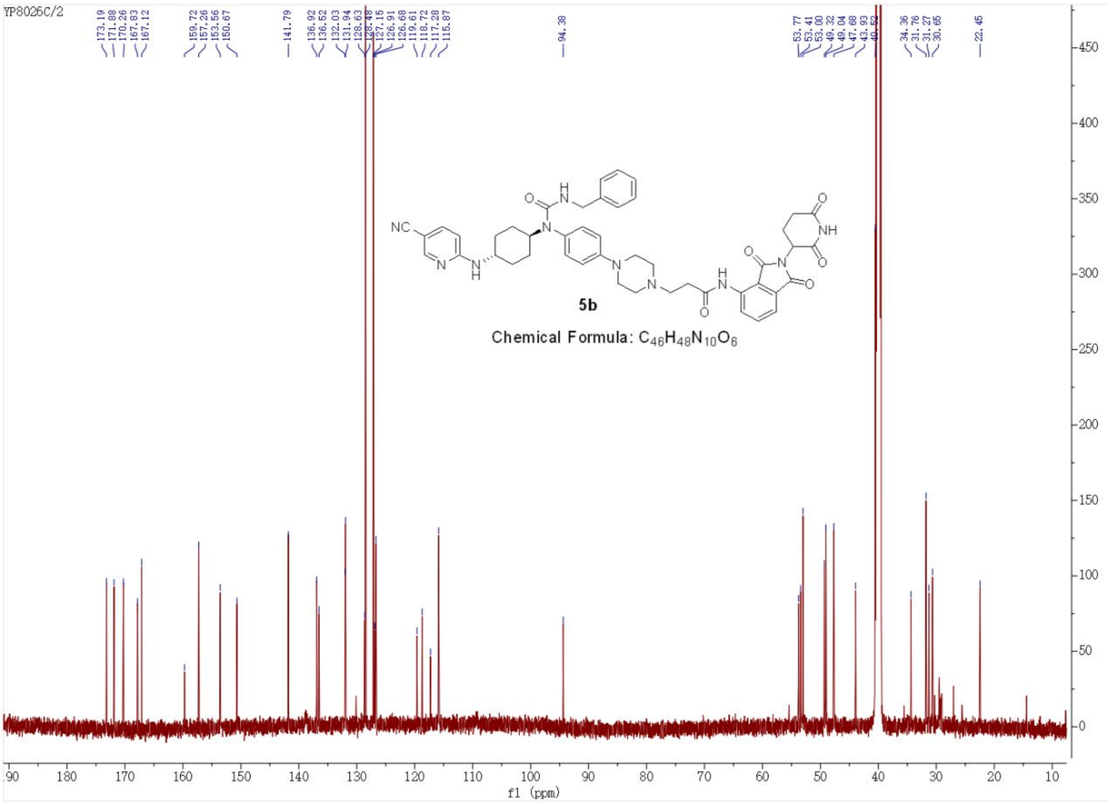

Hit	Formula	m/z	RDB	ppm	MS Rank	MSMS ppm	MSMS Rank	Found
1	C46H48N10O6	837.3831	28.0	-3.5	1			NA/NA

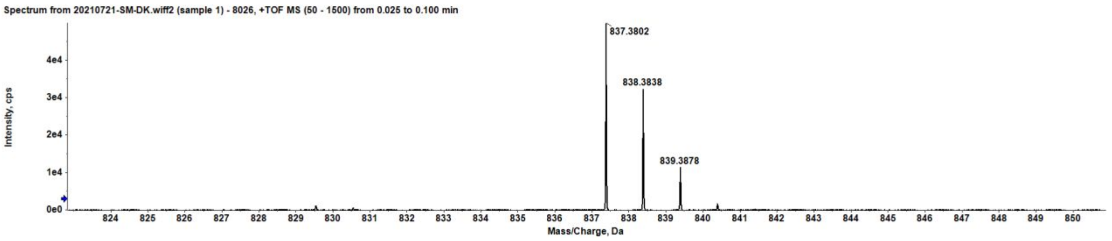

Sample Name: 8026

===

Acq. Operator : 系统
Sample Operator : 系统
Acq. Instrument : 1260LC Location : 35
Injection Date : 16/11/2020 15:34:00
Inj Volume : 10.000 µl
Acq. Method : C:\CHEM32\1\METHODS\80C-20D-30MIN-10UL.M
Last changed : 16/11/2020 15:11:21 by 系统
(modified after loading)
Analysis Method : E:\DK\TL\方法\80C-20A-30min-1u.M
Last changed : 06/04/2021 10:23:35 by 系统

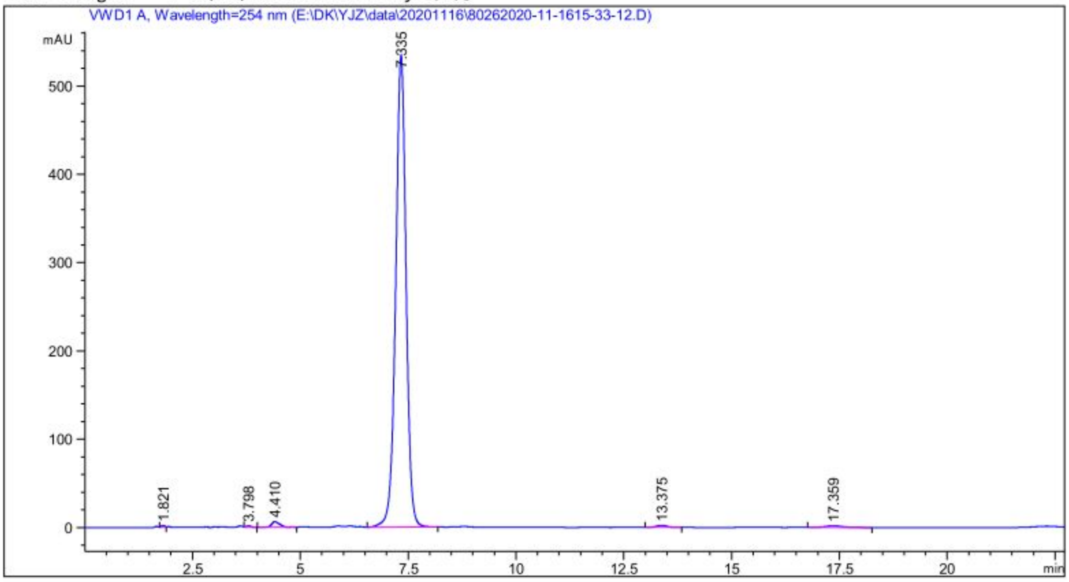

===

Area Percent Report

===

Sorted By : Signal
Multiplier : 1.0000
Dilution : 1.0000
Sample Amount: : 10.00000 [ng/ul] (not used in calc.)
Use Multiplier & Dilution Factor with ISTDs

Signal 1: VWD1 A, Wavelength=254 nm

Peak #	RetTime [min]	Type	Width [min]	Area [mAU*s]	Height [mAU]	Area %
1	1.821	VV	0.0948	14.36778	2.40072	0.1571
2	3.798	VB	0.1229	14.01536	1.74106	0.1532
3	4.410	BV R	0.1833	81.59206	6.37488	0.8921
4	7.335	BB	0.2550	8939.28516	534.71930	97.7406
5	13.375	BB	0.2739	43.72247	2.46539	0.4781
6	17.359	BB	0.4329	52.94258	1.82268	0.5789

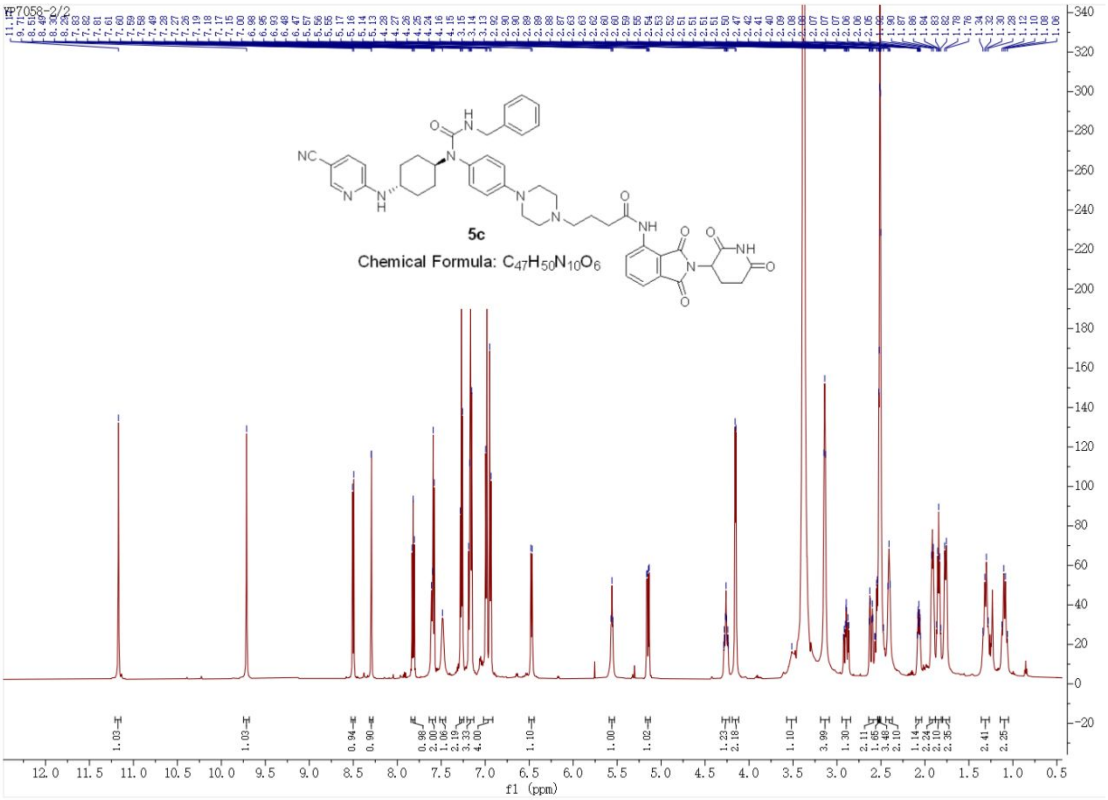
YP7058-2/2
5c
Chemical Formula: C47H50N10O6
f1 (ppm)

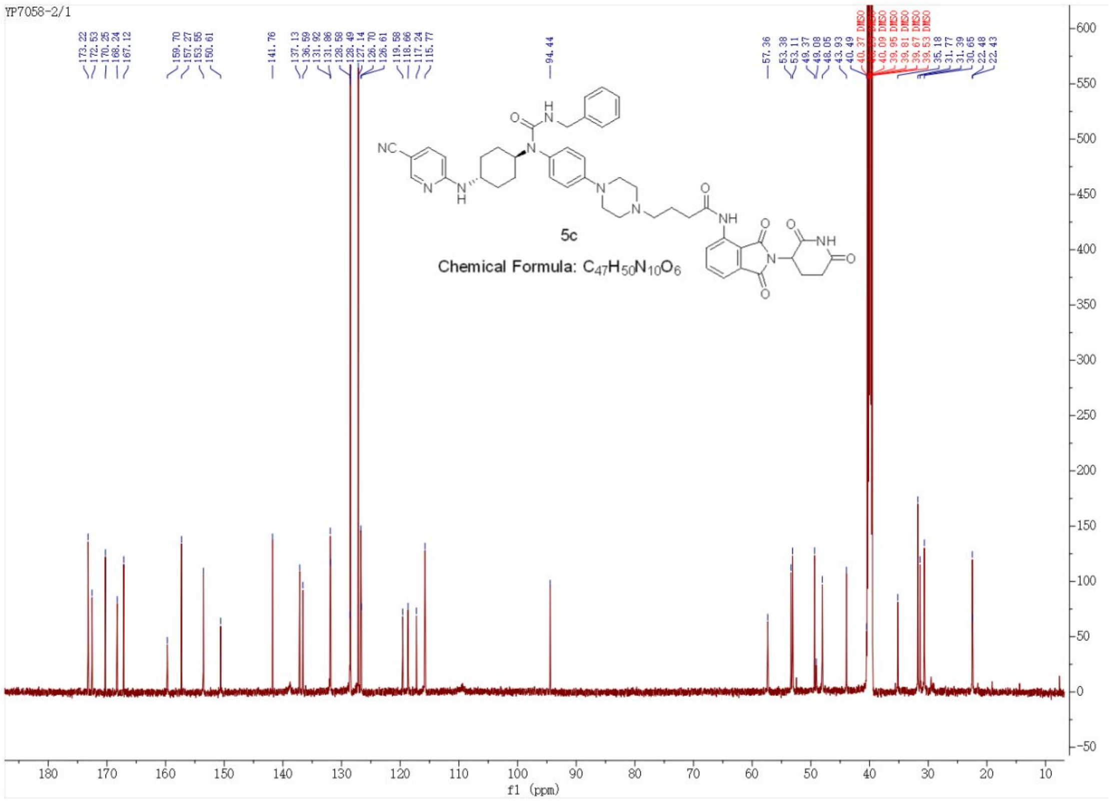
YP7058-2/1
5c
Chemical Formula: C47H50N10O6
f1 (ppm)

Hit	Formula	m/z	RDB	ppm	MS Rank	MSMS ppm	MSMS Rank	Found
1	C47H50N10O6	851.3988	28.0	-2.1	1			NA/NA

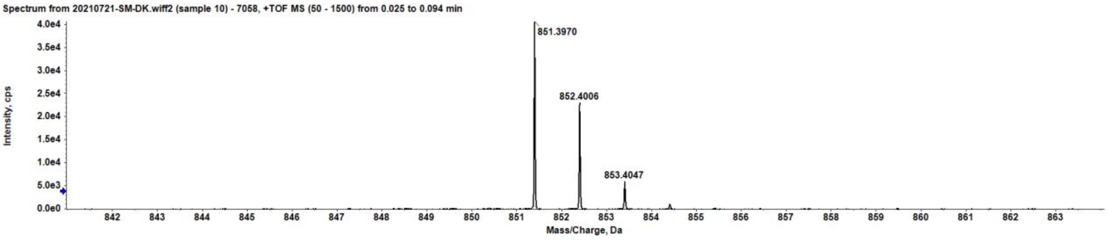

Data File E:\DK\YJZ\data\20210624-ZZ-YJZ-ZFT\YJZ1901DEF_LC 2021-06-26 14-59-43\7058.D
Sample Name: 7058

===
Acq. Operator : 系统 Seq. Line : 7
Acq. Instrument : 1260LC Location : 27
Injection Date : 26/06/2021 18:32:50 Inj : 1
Inj Volume : 15.000 µl
Different Inj Volume from Sample Entry! Actual Inj Volume : 5.000 µl
Method : E:\DK\YJZ\data\20210624-ZZ-YJZ-ZFT\YJZ1901DEF_LC 2021-06-26 14-59-43\75C-25A-30MIN-.M (Sequence Method)
Last changed : 26/06/2021 15:02:42 by 系统

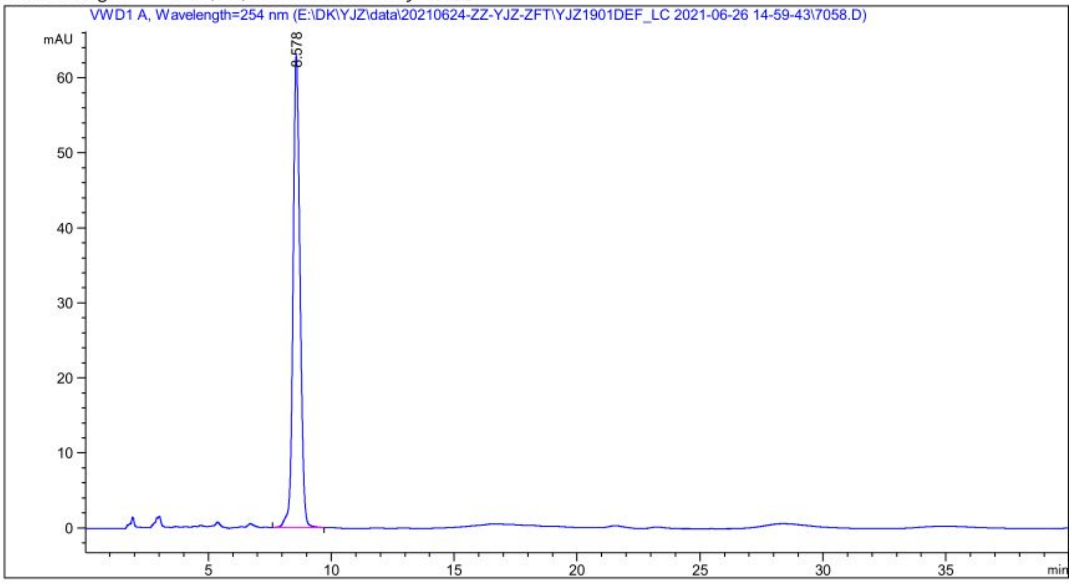

===
Area Percent Report
===

Sorted By : Signal
Multiplier : 1.0000
Dilution : 1.0000
Use Multiplier & Dilution Factor with ISTDs

Signal 1: VWD1 A, Wavelength=254 nm

Peak #	RetTime [min]	Type	Width [min]	Area [mAU*s]	Height [mAU]	Area %
1	8.578	BB	0.3004	1238.98022	63.00480	100.0000

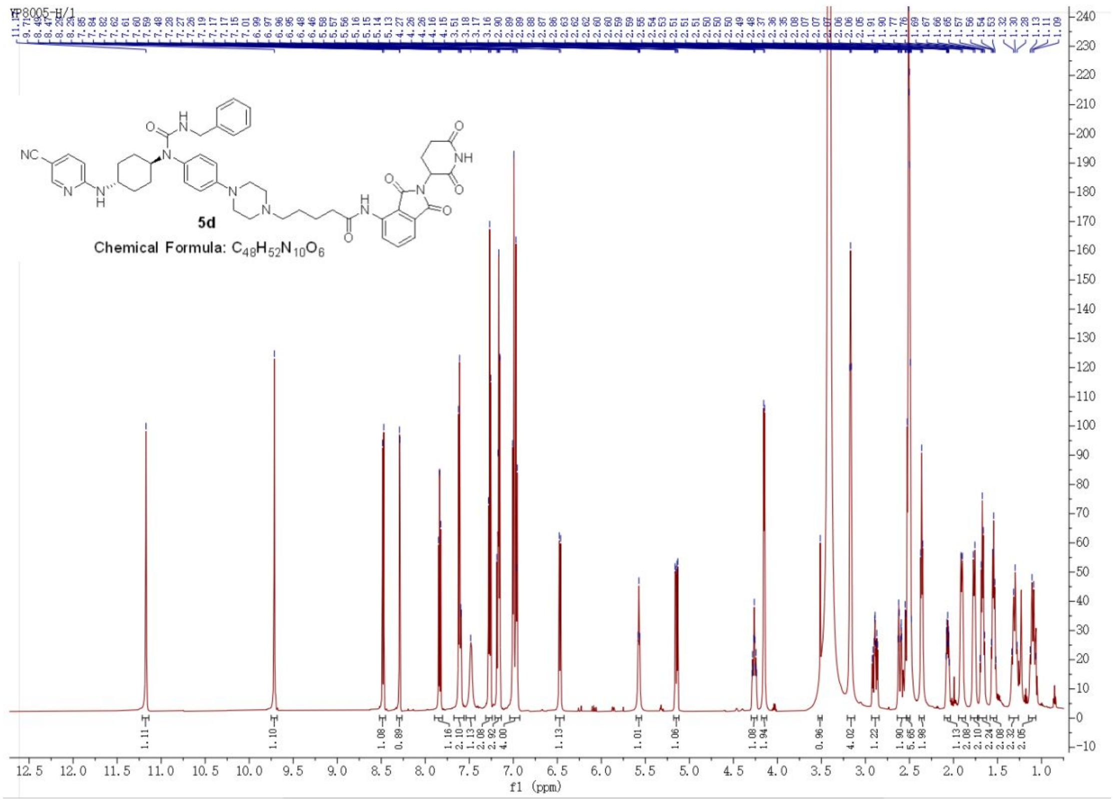
YP8005-H/1
5d
Chemical Formula: C48H52N10O6
f1 (ppm)

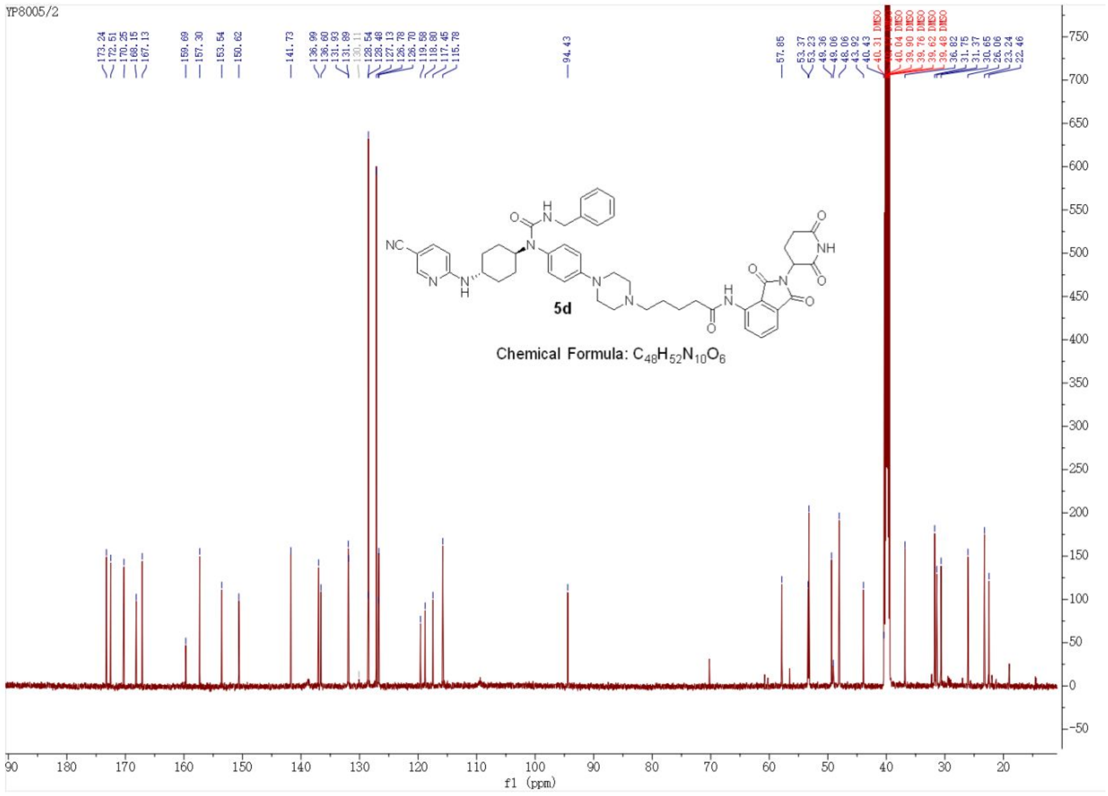
YP8005/2
5d
Chemical Formula: C48H52N10O6
f1 (ppm)

Hit	Formula	m/z	RDB	ppm	MS Rank	MSMS ppm	MSMS Rank	Found
1	C48H52N10O6	865.4144	28.0	-1.3	1			NA/NA

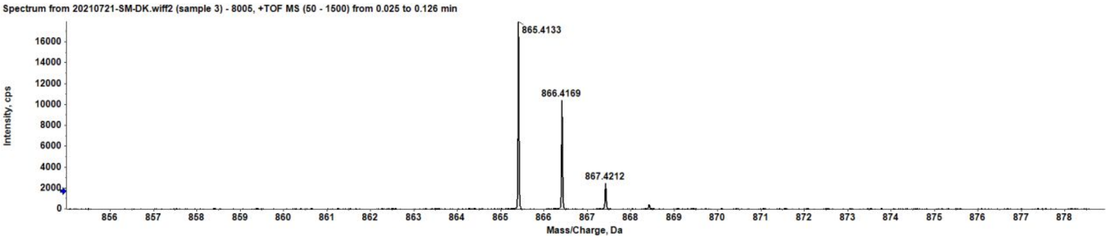

Data File E:\DK\ZZ\20210624-ZZ-YJZ-ZFT\YJZ1901DEF_LC 2021-06-24 20-23-22\8005.D
Sample Name: 8005

```
=====================================================================
Acq. Operator   : 系统                            Seq. Line :  56
Acq. Instrument : 1260LC                          Location :   24
Injection Date  : 26/06/2021 02:38:56                  Inj :   1
                                              Inj Volume : 10.000 µl
Method          : E:\DK\ZZ\20210624-ZZ-YJZ-ZFT\YJZ1901DEF_LC 2021-06-24 20-23-22\75C-25A-
                  30min-1u.M (Sequence Method)
Last changed    : 24/06/2021 20:23:22 by 系统
```

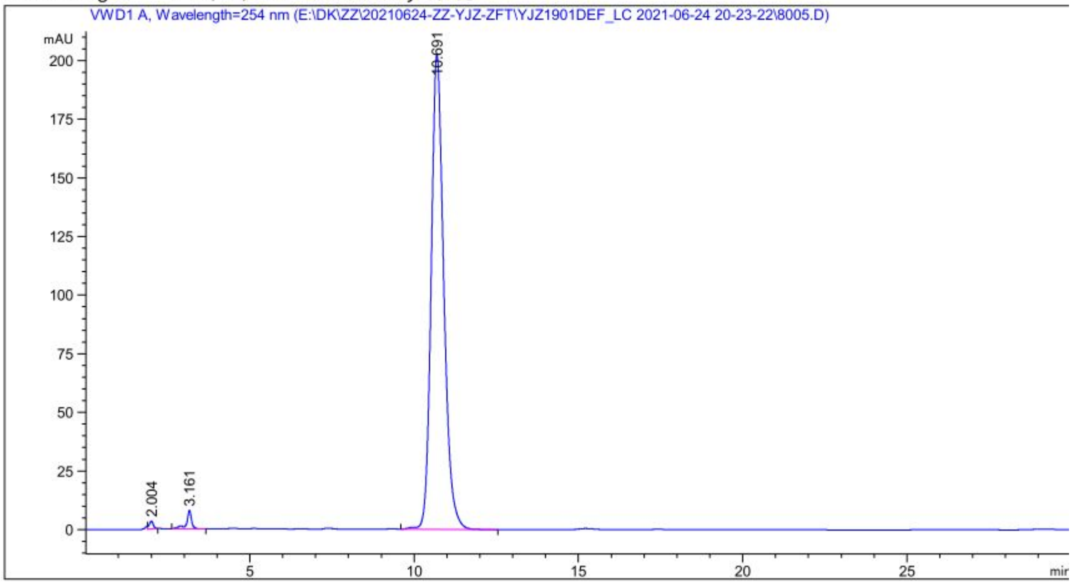


```
=====================================================================
                         Area Percent Report
=====================================================================

Sorted By             :      Signal
Multiplier            :      1.0000
Dilution              :      1.0000
Use Multiplier & Dilution Factor with ISTDs


Signal 1: VWD1 A, Wavelength=254 nm

Peak RetTime Type  Width     Area      Height     Area
  #   [min]        [min]   [mAU*s]     [mAU]        %
----|-------|----|-------|----------|----------|--------|
   1   2.004 VB    0.1334   29.17232    3.32326   0.5293
   2   3.161 VB R  0.1489   82.95723    7.87566   1.5051
   3  10.691 BB    0.4055 5399.76270  202.25615  97.9657
```

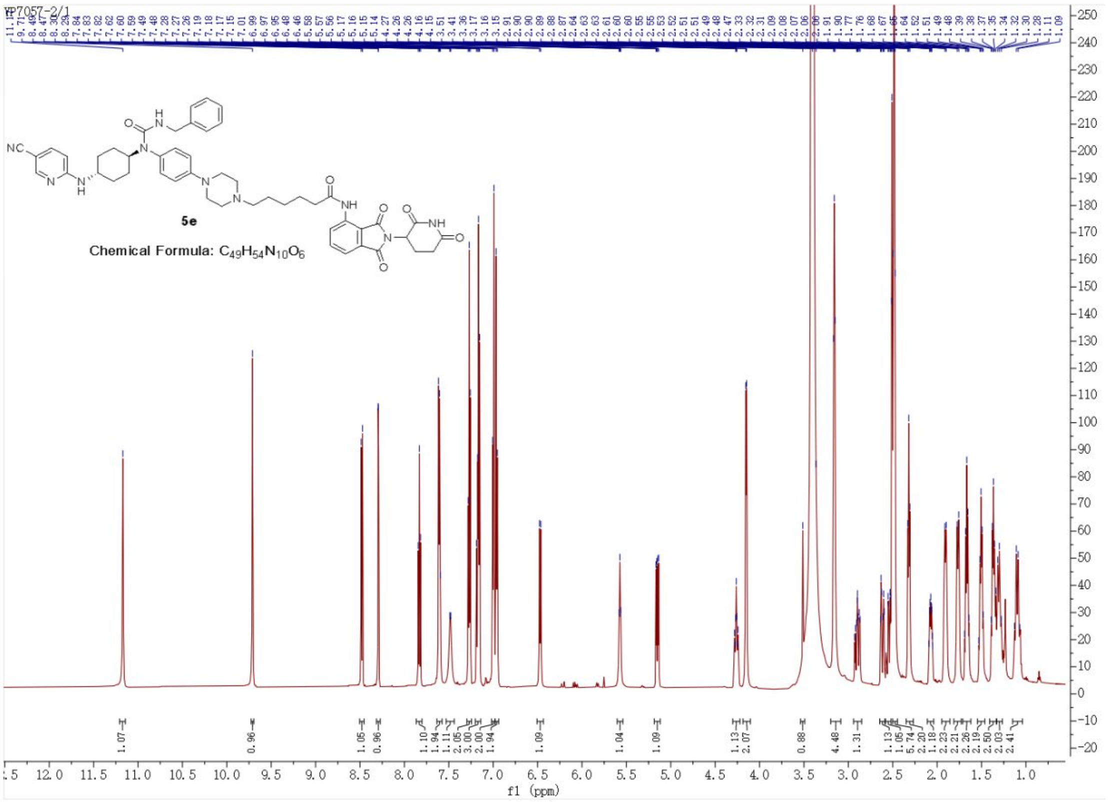
YP7057-2/1
5e
Chemical Formula: C49H54N10O6
f1 (ppm)

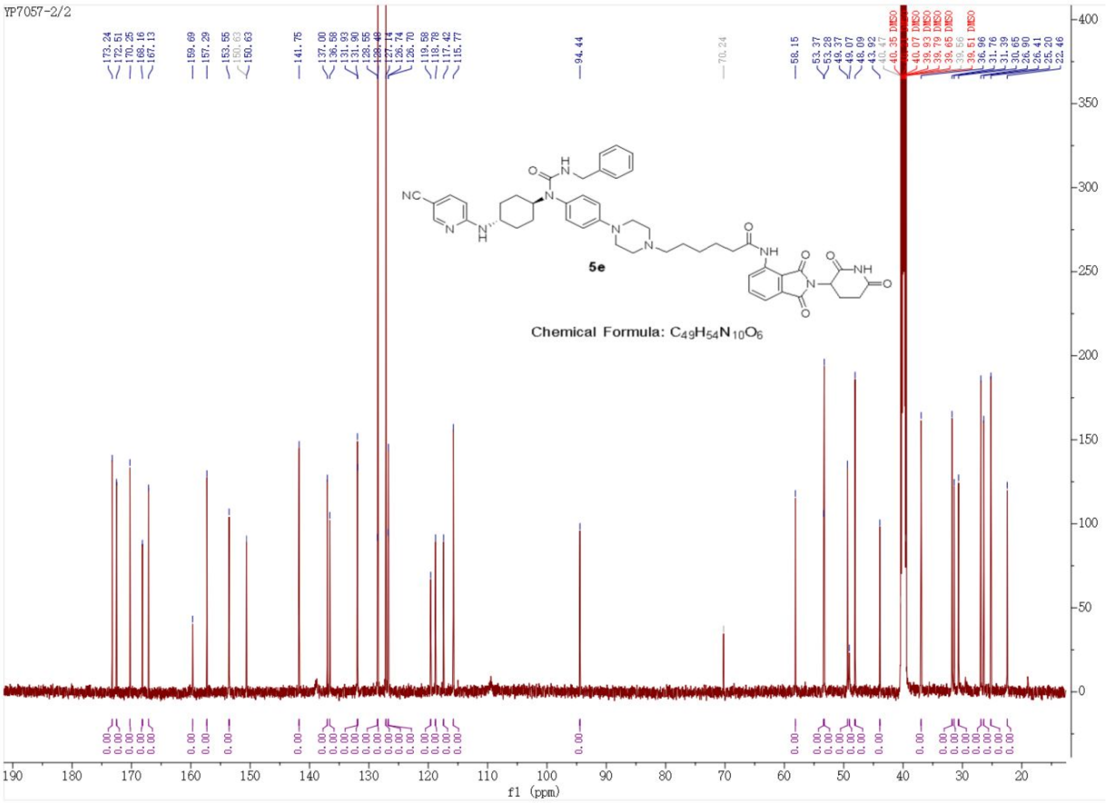
YP7057-2/2
5e
Chemical Formula: C49H54N10O6
f1 (ppm)

Hit	Formula	m/z	RDB	ppm	MS Rank	MSMS ppm	MSMS Rank	Found
1	C49H54N10O6	879.4301	28.0	-1.9	1			NA/NA

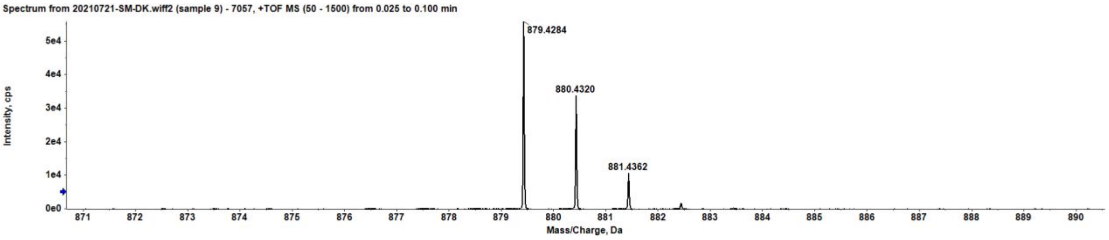

Data File E:\DK\ZZ\20210624-ZZ-YJZ-ZFT\YJZ1901DEF_LC 2021-06-24 20-23-22\7057-1.D
Sample Name: 7057

===

Acq. Operator : 系统 Seq. Line : 67
Acq. Instrument : 1260LC Location : 94
Injection Date : 26/06/2021 08:18:41 Inj : 1
Inj Volume : 10.000 µl
Method : E:\DK\ZZ\20210624-ZZ-YJZ-ZFT\YJZ1901DEF_LC 2021-06-24 20-23-22\75C-25A-30min-1u.M (Sequence Method)
Last changed : 24/06/2021 20:23:22 by 系统

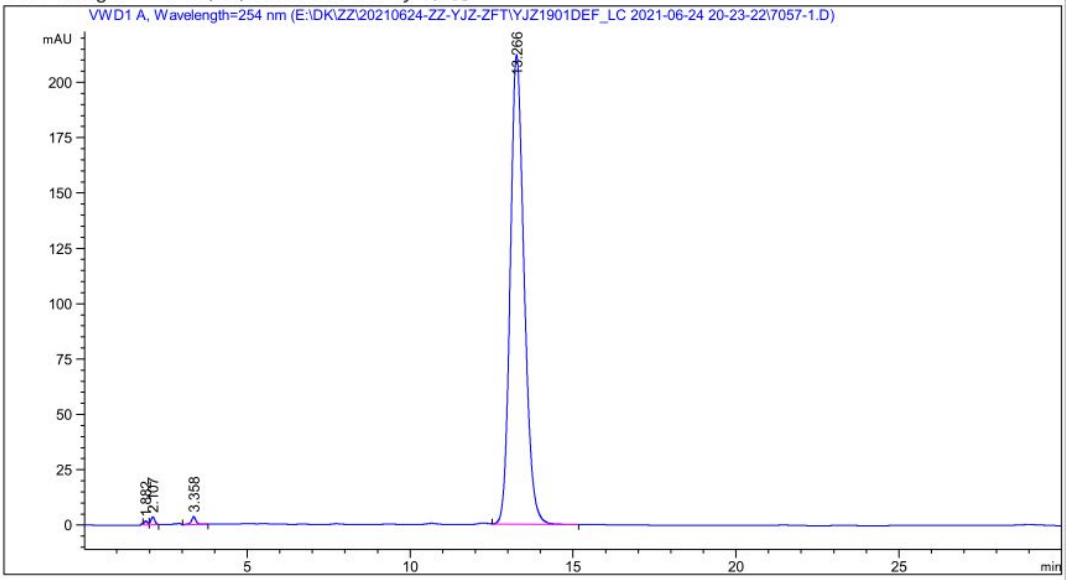

===

Area Percent Report

===

Sorted By : Signal
Multiplier : 1.0000
Dilution : 1.0000
Use Multiplier & Dilution Factor with ISTDs

Signal 1: VWD1 A, Wavelength=254 nm

Peak #	RetTime [min]	Type	Width [min]	Area [mAU*s]	Height [mAU]	Area %
1	1.882	VV	0.1054	14.14606	1.95501	0.2228
2	2.107	VB	0.1307	28.51408	3.44194	0.4490
3	3.358	BB	0.1452	35.92309	3.60558	0.5657
4	13.266	BB	0.4503	6271.90088	211.92545	98.7626

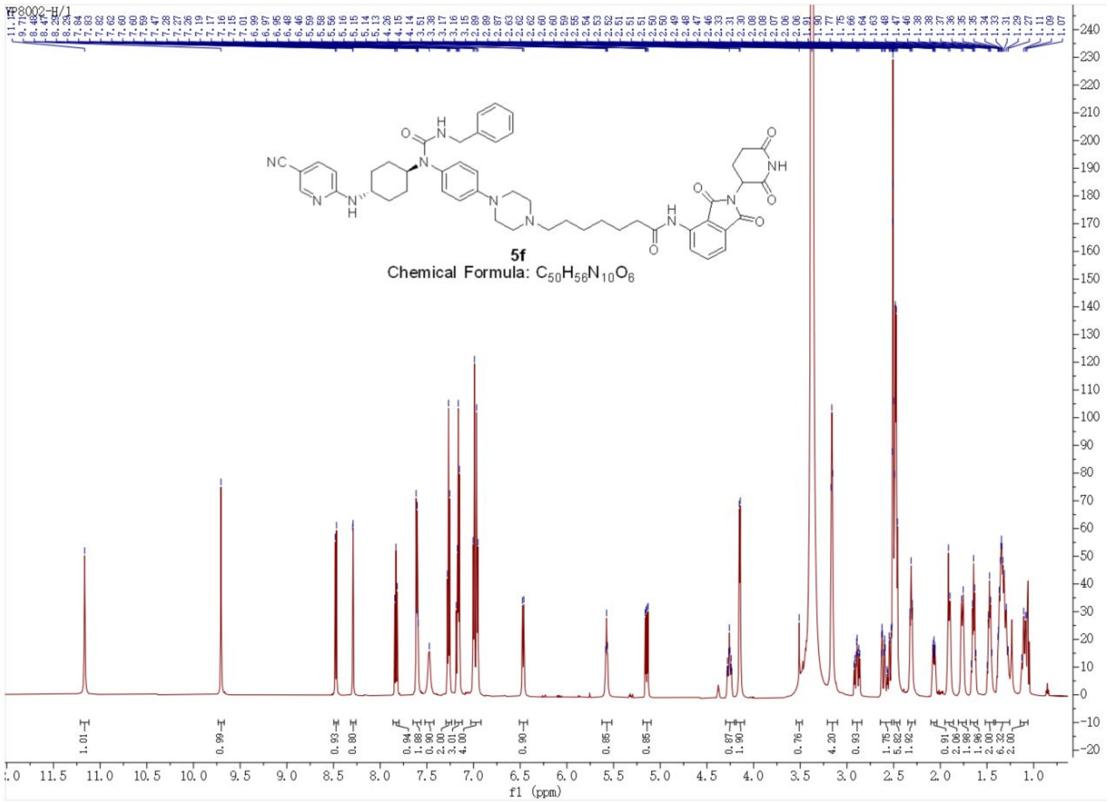
YP8002-H/1
5f
Chemical Formula: C50H56N10O6
f1 (ppm)

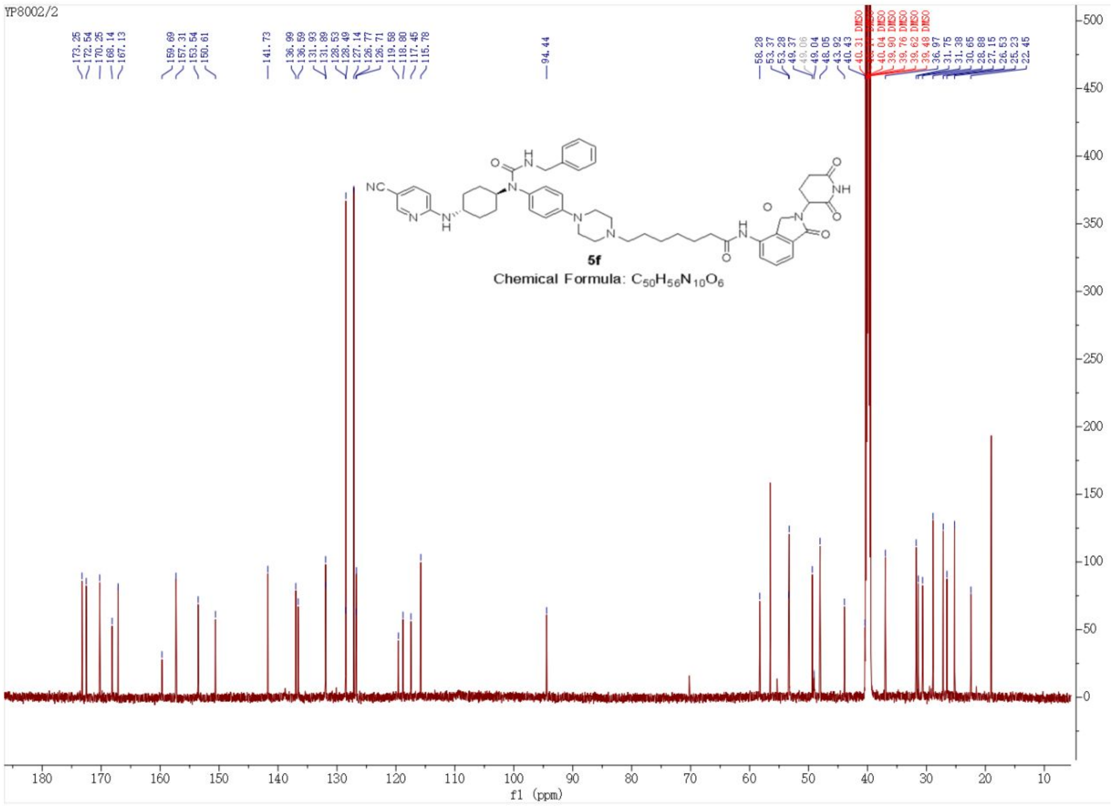
YP8002/2
5f
Chemical Formula: C50H56N10O6
f1 (ppm)

Hit	Formula	m/z	RDB	ppm	MS Rank	MSMS ppm	MSMS Rank	Found
1	C50H56N10O6	893.4457	28.0	-1.2	1			NA/NA

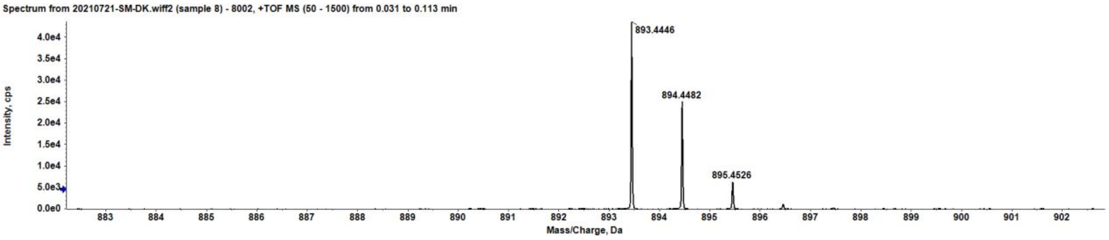

Data File E:\DK\ZZ\20210624-ZZ-YJZ-ZFT\YJZ1901DEF_LC 2021-06-24 20-23-22\8002-1.D
Sample Name: 8002

===

Acq. Operator : 系统 Seq. Line : 69
Acq. Instrument : 1260LC Location : 96
Injection Date : 26/06/2021 09:20:24 Inj : 1
Inj Volume : 10.000 µl
Acq. Method : E:\DK\ZZ\20210624-ZZ-YJZ-ZFT\YJZ1901DEF_LC 2021-06-24 20-23-22\75C-25A-30min-1u.M
Last changed : 24/06/2021 20:23:22 by 系统
Analysis Method : E:\DK\ZZ\20210624-ZZ-YJZ-ZFT\YJZ1901DEF_LC 2021-06-24 20-23-22\75C-25A-30min-1u.M (Sequence Method)
Last changed : 26/06/2021 10:55:14 by 系统

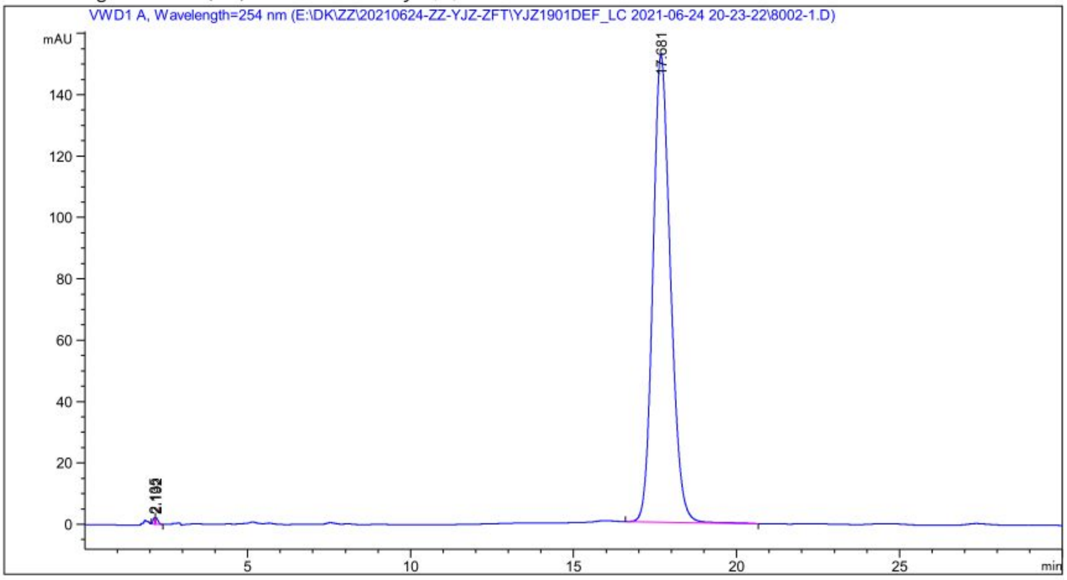

===
Area Percent Report
===

Sorted By : Signal
Multiplier : 1.0000
Dilution : 1.0000
Use Multiplier & Dilution Factor with ISTDs

Signal 1: VWD1 A, Wavelength=254 nm

Peak #	RetTime [min]	Type	Width [min]	Area [mAU*s]	Height [mAU]	Area %
1	2.135	BV	0.0696	8.82682	1.99687	0.1542
2	2.192	VB	0.0916	12.24049	2.02341	0.2138
3	17.681	BB	0.5709	5704.62354	152.32877	99.6321

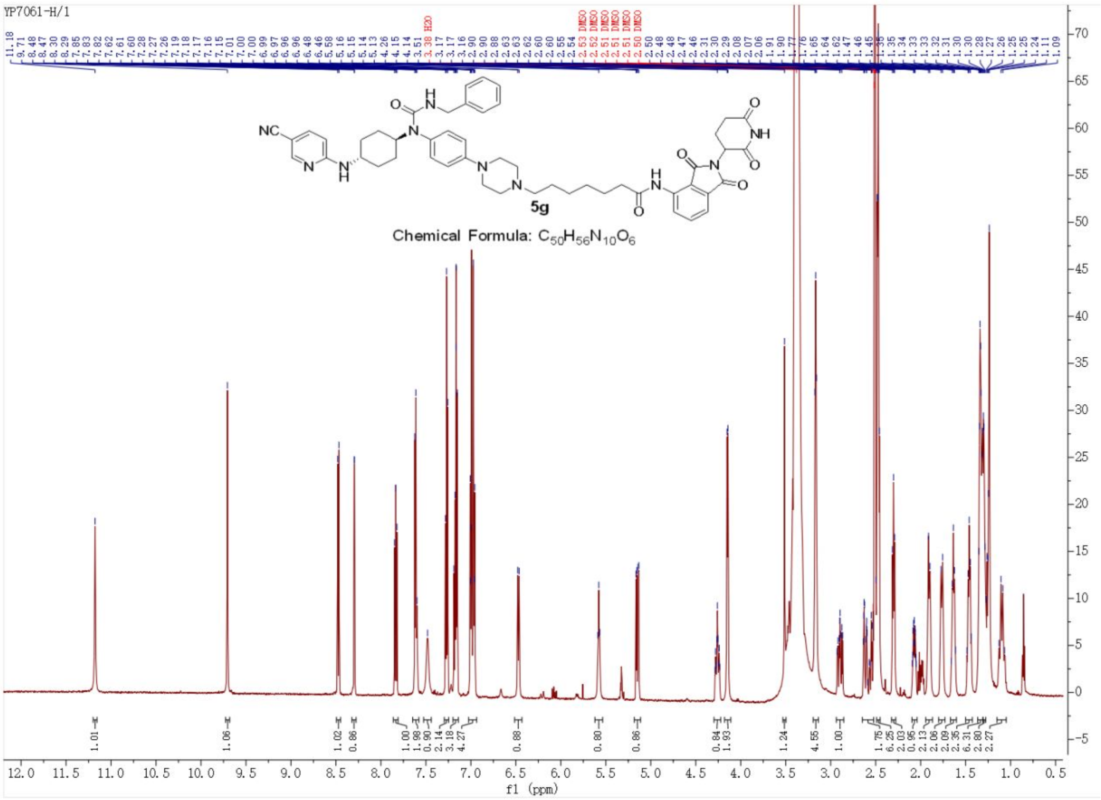
YP7061-H/1
5g
Chemical Formula: $C_{50}H_{56}N_{10}O_6$
f1 (ppm)

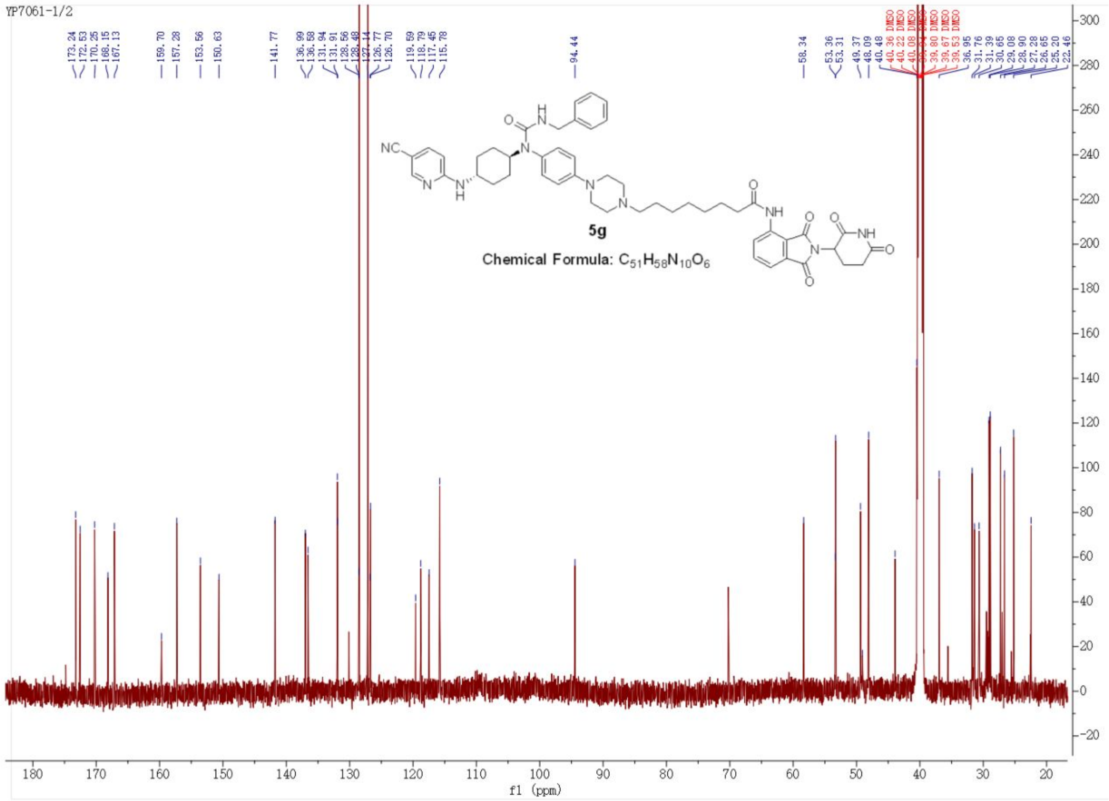
YP7061-1/2
5g
Chemical Formula: $C_{51}H_{58}N_{10}O_6$
f1 (ppm)

Hit	Formula	m/z	RDB	ppm	MS Rank	MSMS ppm	MSMS Rank	Found
1	C51H58N1006	907.4614	28.0	-2.7	1			NA/NA

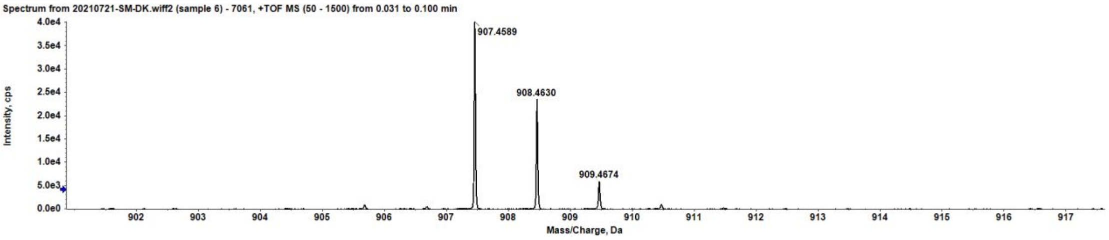


```
Data File E:\DK\YJZ\data\20210624-ZZ-YJZ-ZFT\YJZ1901DEF_LC 2021-06-26 14-59-43\7061.D
Sample Name: 7061

    =====================================================================
    Acq. Operator   : 系统                          Seq. Line :   4
    Acq. Instrument : 1260LC                         Location :   25
    Injection Date  : 26/06/2021 16:40:21                 Inj :   1
                                                   Inj Volume : 6.000 µl
    Different Inj Volume from Sample Entry!  Actual Inj Volume : 5.000 µl
    Sequence File   : E:\DK\YJZ\data\20210624-ZZ-YJZ-ZFT\YJZ1901DEF_LC 2021-06-26 14-59-43
                      \YJZ1901DEF_LC.S
    Method          : E:\DK\YJZ\data\20210624-ZZ-YJZ-ZFT\YJZ1901DEF_LC 2021-06-26 14-59-43\80C-
                      20A-30min-1u.M (Sequence Method)
    Last changed    : 26/06/2021 15:04:42 by 系统
```

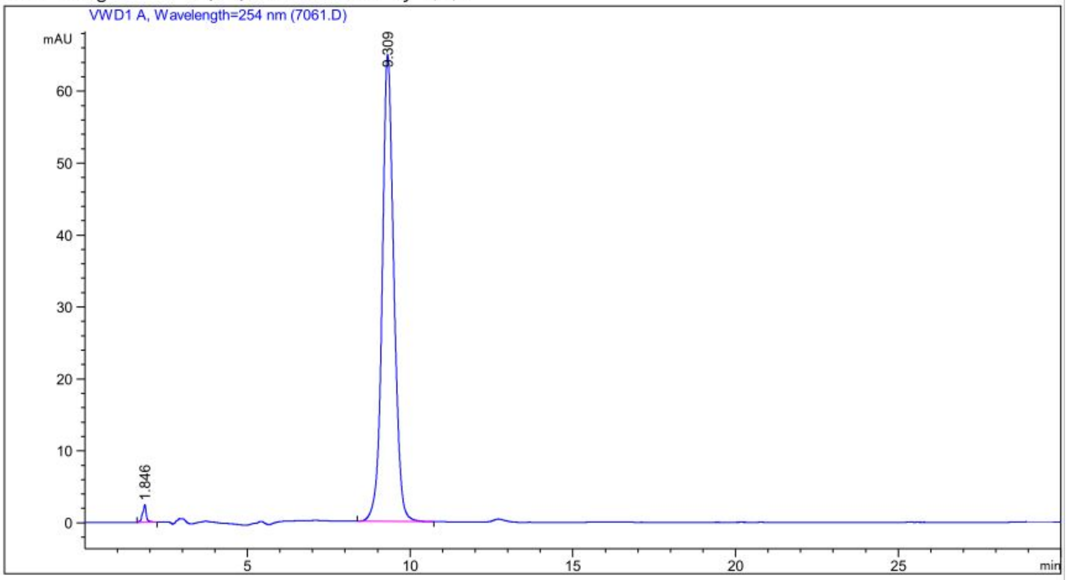


```
    =====================================================================
                             Area Percent Report
    =====================================================================

    Sorted By             :      Signal
    Multiplier            :      1.0000
    Dilution              :      1.0000
    Use Multiplier & Dilution Factor with ISTDs


    Signal 1: VWD1 A, Wavelength=254 nm

    Peak RetTime Type  Width     Area      Height     Area
      #   [min]        [min]   [mAU*s]     [mAU]        %
    ----|-------|----|-------|----------|----------|--------|
       1   1.846 BB    0.1121   19.61035    2.45728   1.1956
       2   9.309 BB    0.3744 1620.64746   64.88203  98.8044
```

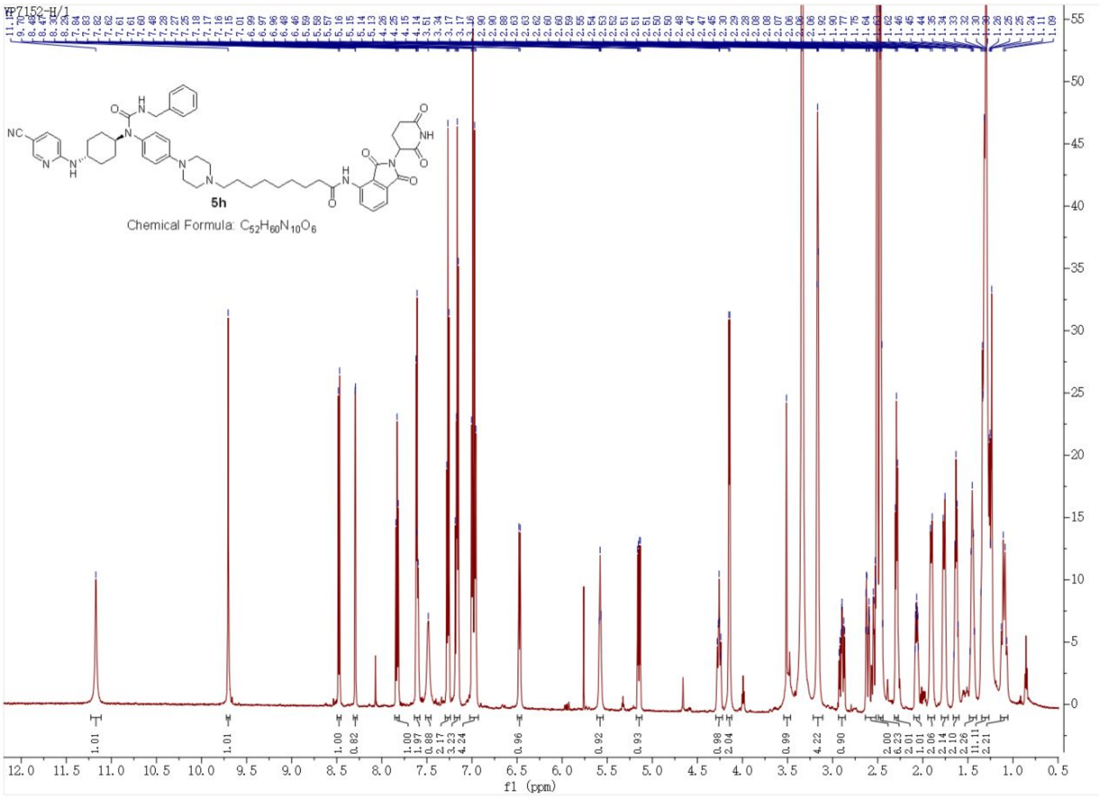
YP7152-H/1
5h
Chemical Formula: $C_{52}H_{60}N_{10}O_6$
f1 (ppm)

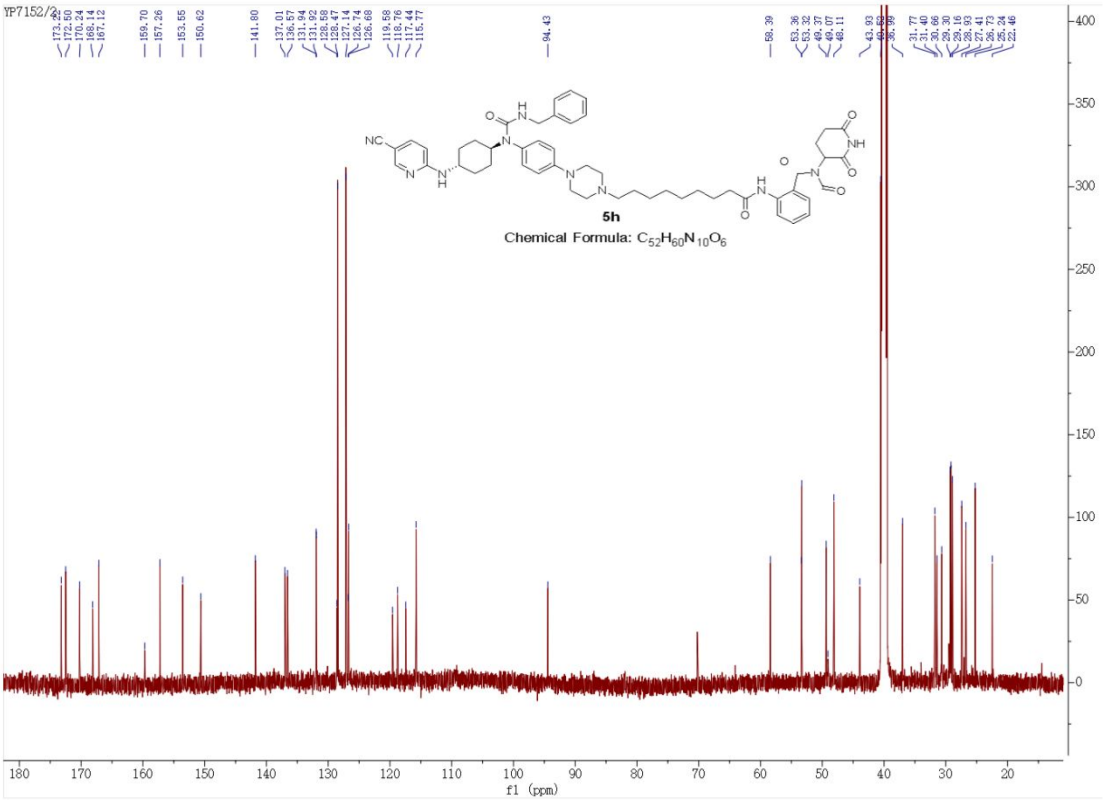
YP7152/2
5h
Chemical Formula: $C_{52}H_{60}N_{10}O_6$
f1 (ppm)

Hit	Formula	m/z	RDB	ppm	MS Rank	MSMS ppm	MSMS Rank	Found
1	C52H60N10O6	921.4770	28.0	-1.7	1			NA/NA

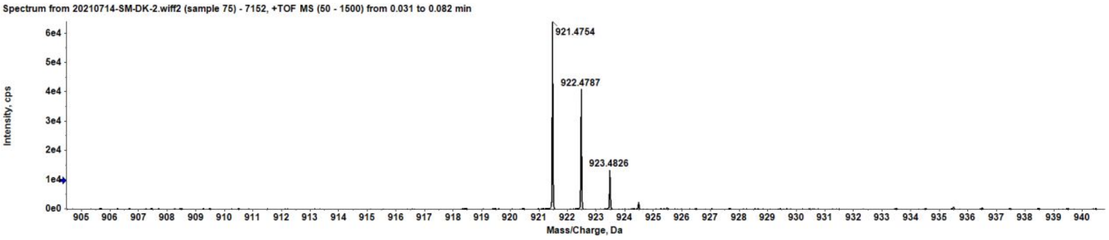

Data File E:\DK\YJZ\data\20210628\7152-.D
Sample Name: 7152-

===

Acq. Operator : 系统
Sample Operator : 系统
Acq. Instrument : 1260LC Location : 93
Injection Date : 29/06/2021 13:14:03
Inj Volume : 15.000 µl
Acq. Method : E:\DK\TL\方法\75C-25A-30MIN-.M
Last changed : 29/06/2021 12:07:29 by 系统
(modified after loading)
Analysis Method : E:\DK\TL\方法\80C-20A-30min-1u.M
Last changed : 06/04/2021 10:23:35 by 系统

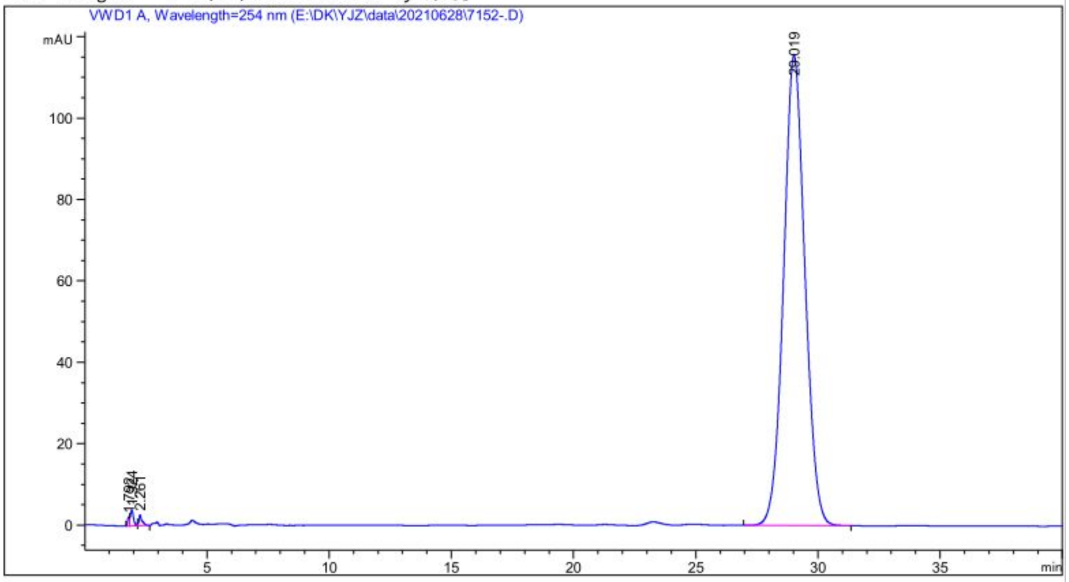

===

Area Percent Report

===

Sorted By : Signal
Multiplier : 1.0000
Dilution : 1.0000
Sample Amount: : 10.00000 [ng/ul] (not used in calc.)
Use Multiplier & Dilution Factor with ISTDs

Signal 1: VWD1 A, Wavelength=254 nm

Peak #	RetTime [min]	Type	Width [min]	Area [mAU*s]	Height [mAU]	Area %
1	1.792	BV	0.0773	10.64758	2.13157	0.1509
2	1.924	VV R	0.1396	36.32980	3.90444	0.5148
3	2.261	VB	0.1213	22.49001	2.61590	0.3187
4	29.019	BB	0.9186	6988.20996	115.54807	99.0157

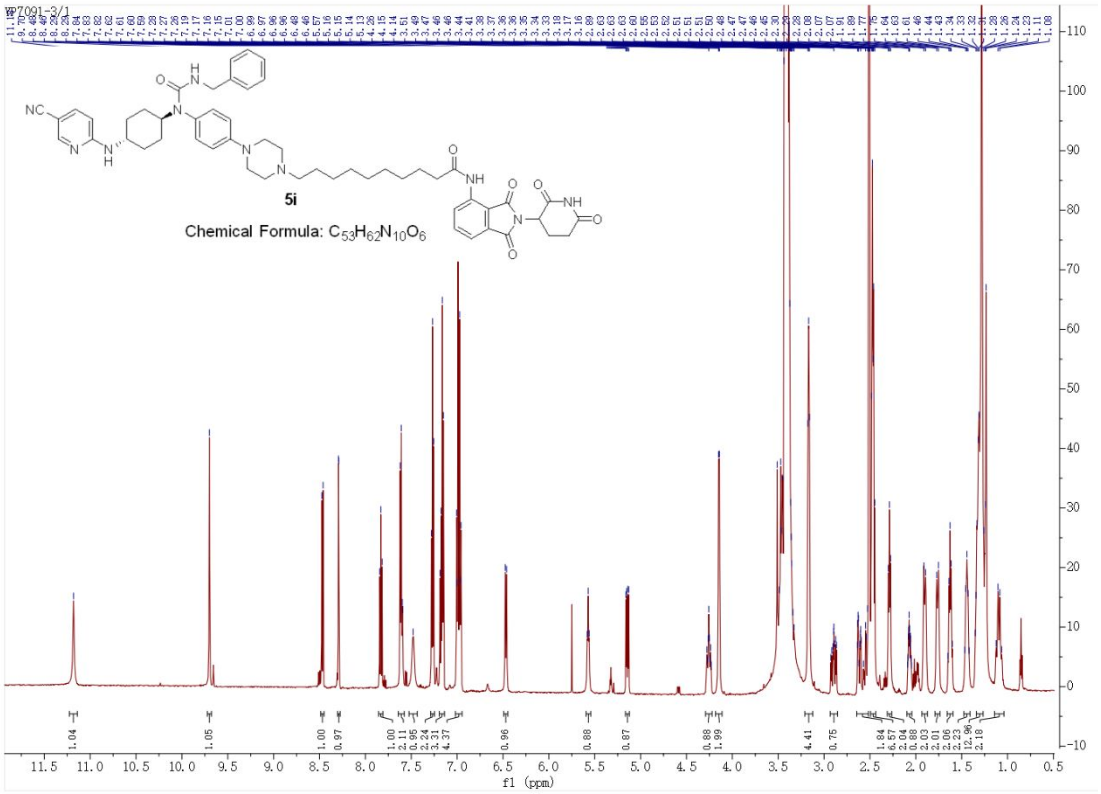
YP7091-3/1
5i
Chemical Formula: $C_{53}H_{62}N_{10}O_6$
f1 (ppm)

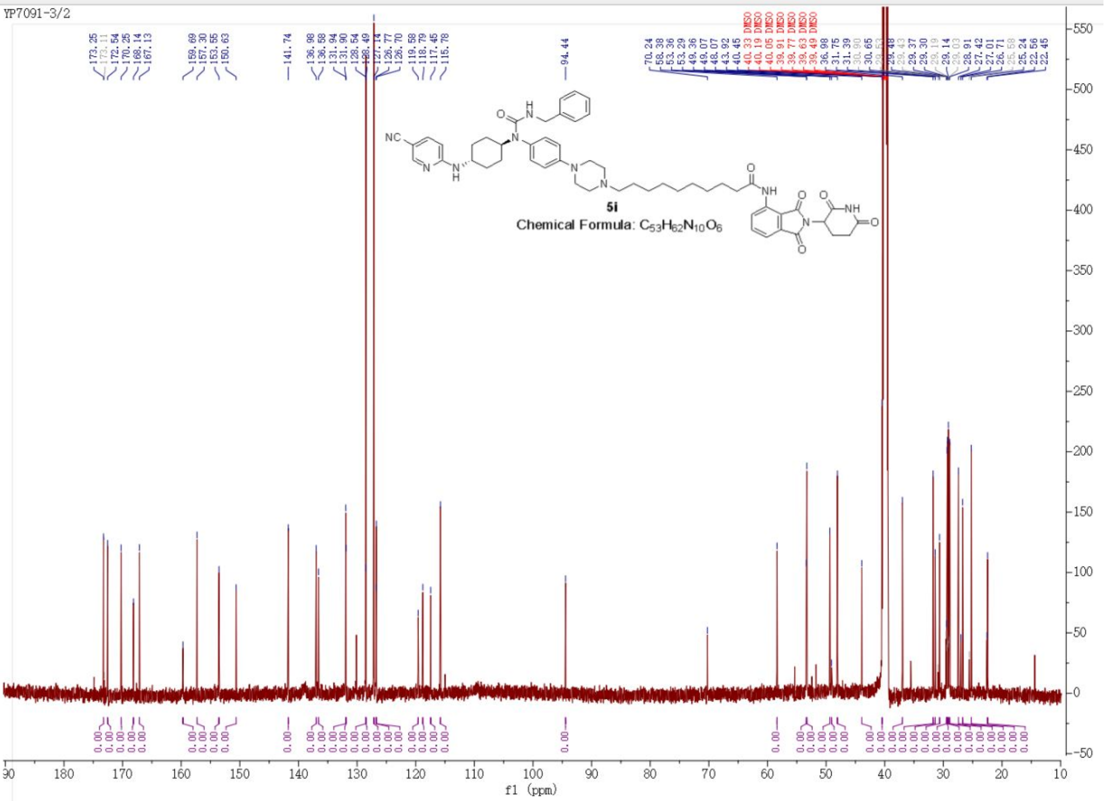
YP7091-3/2
5i
Chemical Formula: $C_{53}H_{62}N_{10}O_6$
f1 (ppm)

Hit	Formula	m/z	RDB	ppm	MS Rank	MSMS ppm	MSMS Rank	Found
1	C53H62N10O6	935.4927	28.0	-2.5	1			NA/NA

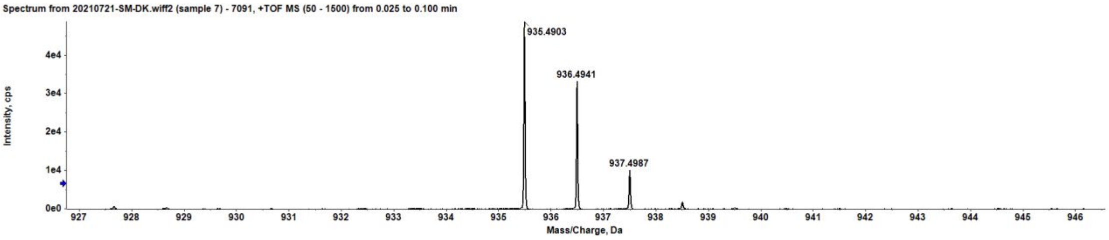


```
Data File E:\DK\YJZ\data\20210624-ZZ-YJZ-ZFT\YJZ1901DEF_LC 2021-06-26 14-59-43\7091.D
Sample Name: 7091

    =====================================================================
    Acq. Operator   : 系统                           Seq. Line :   3
    Acq. Instrument : 1260LC                          Location :   24
    Injection Date  : 26/06/2021 16:10:31                  Inj :   1
                                                    Inj Volume : 15.000 µl
    Different Inj Volume from Sample Entry!  Actual Inj Volume : 5.000 µl
    Sequence File   : E:\DK\YJZ\data\20210624-ZZ-YJZ-ZFT\YJZ1901DEF_LC 2021-06-26 14-59-43
                      \YJZ1901DEF_LC.S
    Method          : E:\DK\YJZ\data\20210624-ZZ-YJZ-ZFT\YJZ1901DEF_LC 2021-06-26 14-59-43\85C-
                      15A-30min-1u.M (Sequence Method)
    Last changed    : 26/06/2021 16:34:15 by 系统
                      (modified after loading)
```

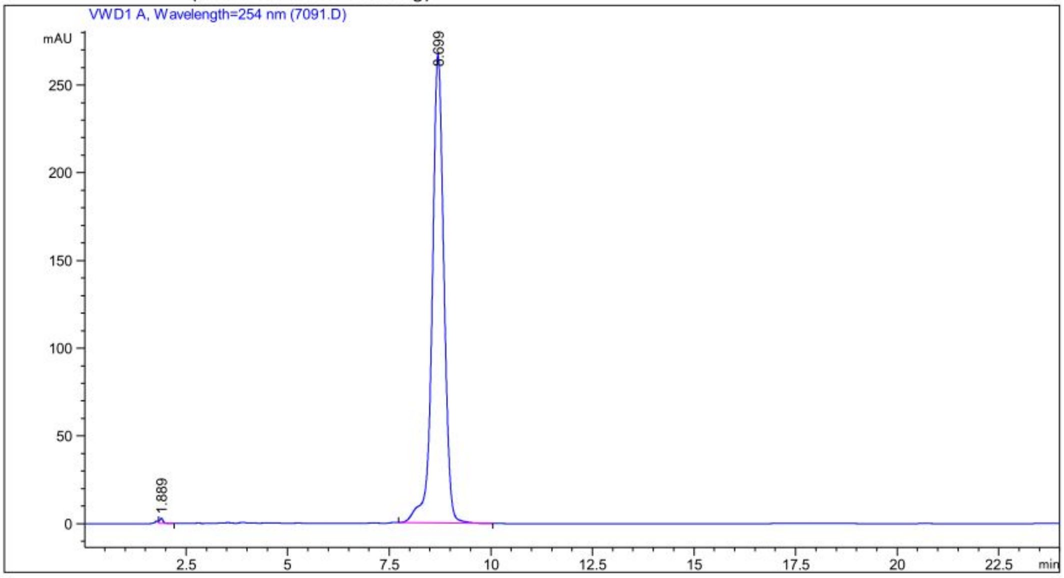


```
    =====================================================================
                             Area Percent Report
    =====================================================================

    Sorted By             :      Signal
    Multiplier            :      1.0000
    Dilution              :      1.0000
    Use Multiplier & Dilution Factor with ISTDs


    Signal 1: VWD1 A, Wavelength=254 nm

    Peak RetTime Type  Width     Area      Height     Area
      #   [min]        [min]   [mAU*s]     [mAU]        %
    ----|-------|----|-------|----------|----------|--------|
       1   1.889 VB    0.0908   18.86967    3.06401   0.3518
       2   8.699 BB    0.3023 5345.37451  267.29733  99.6482
```

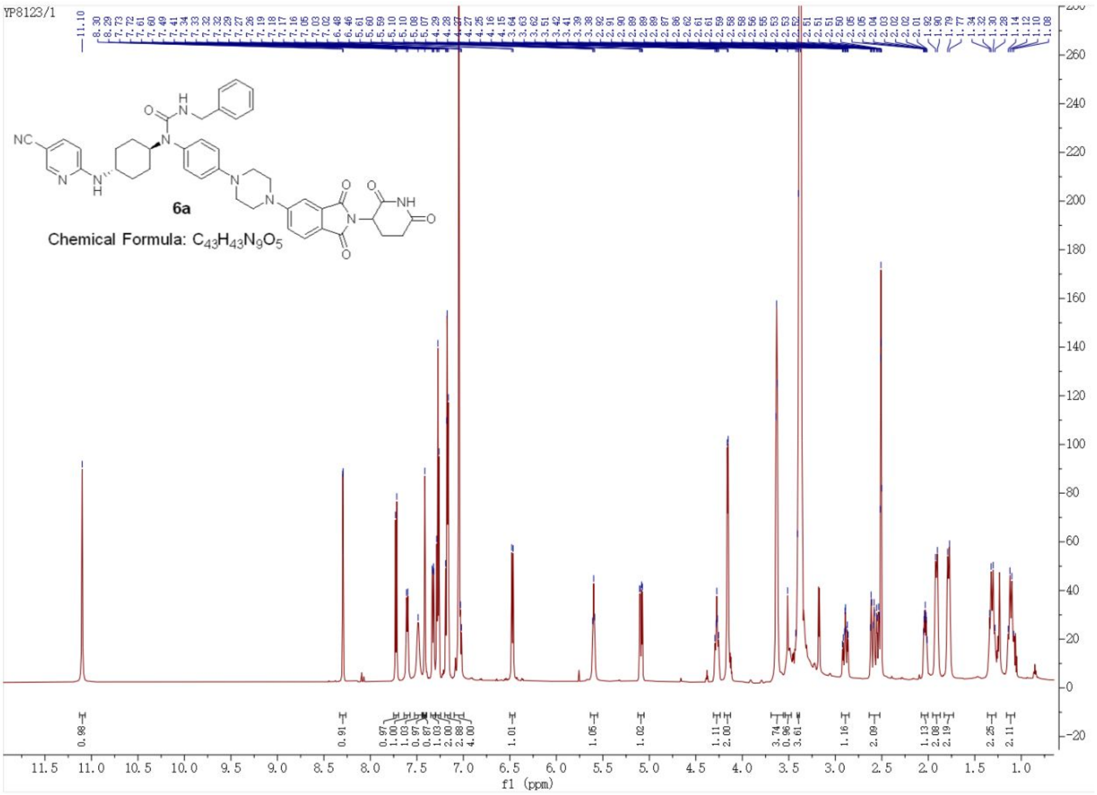

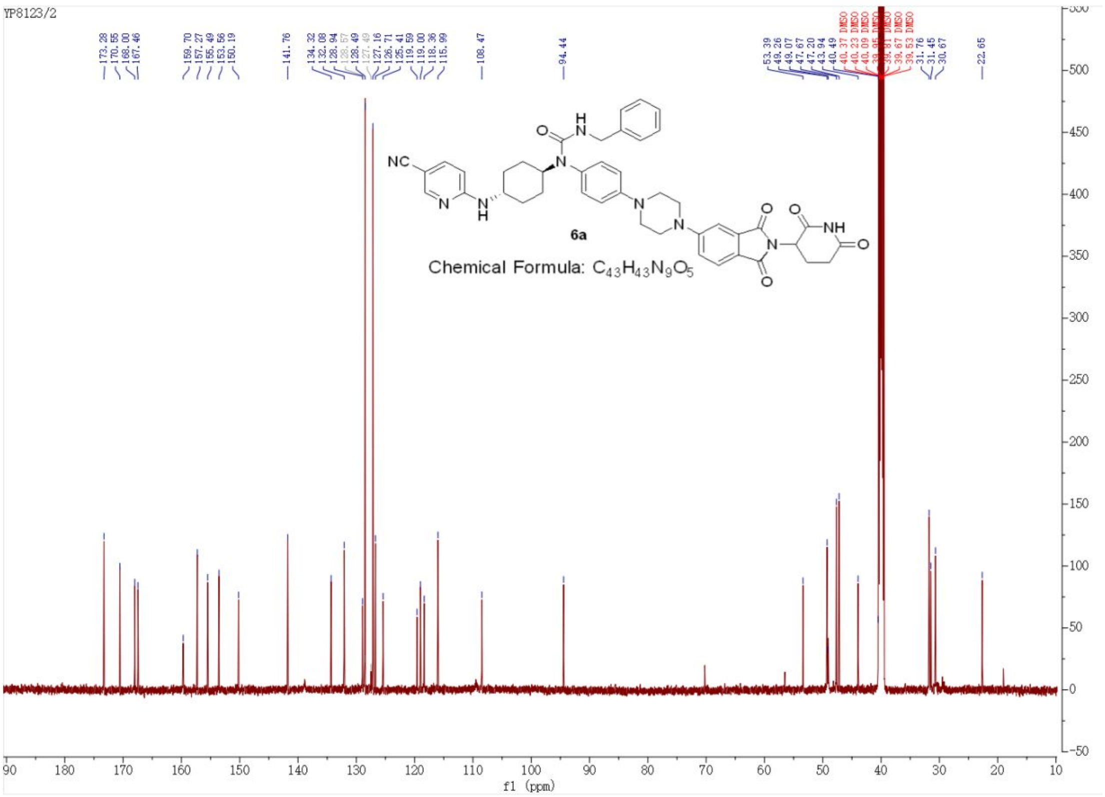

Hit	Formula	m/z	RDB	ppm	MS Rank	MSMS ppm	MSMS Rank	Found
1	C43H43N905	766.3460	27.0	-3.8	1			NA/NA

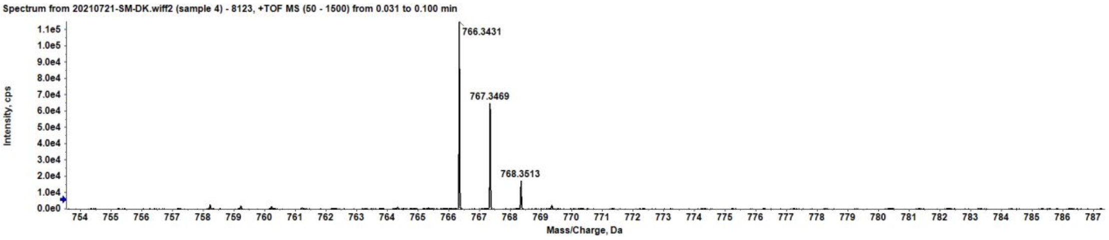

Data File E:\DK\YJZ\data\20210627\YJZ1901DEF_LC 2021-06-27 17-33-40\8123-.D
Sample Name: 8123-

===

```
Acq. Operator   : 系统                           Seq. Line :  18
Acq. Instrument : 1260LC                          Location :   22
Injection Date  : 28/06/2021 02:37:58                  Inj :   1
                                              Inj Volume : 10.000 µl
Different Inj Volume from Sample Entry!  Actual Inj Volume : 4.000 µl
Method          : E:\DK\YJZ\data\20210627\YJZ1901DEF_LC 2021-06-27 17-33-40\73C.27AM.M (
                  Sequence Method)
Last changed    : 27/06/2021 22:45:45 by 系统
```

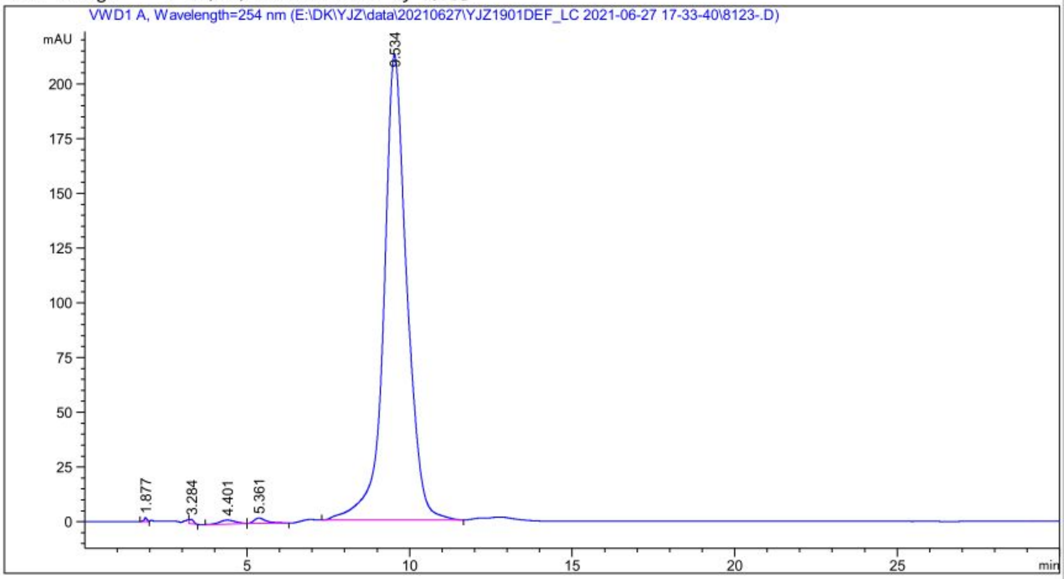

===

Area Percent Report

===

```
Sorted By            :      Signal
Multiplier           :      1.0000
Dilution             :      1.0000
Use Multiplier & Dilution Factor with ISTDs
```

Signal 1: VWD1 A, Wavelength=254 nm

Peak #	RetTime [min]	Type	Width [min]	Area [mAU*s]	Height [mAU]	Area %
1	1.877	BV	0.0980	11.49230	1.74023	0.1084
2	3.284	VB	0.1413	17.53938	1.88884	0.1654
3	4.401	BB	0.4232	57.90798	1.77705	0.5460
4	5.361	BB	0.3881	62.64831	2.39534	0.5907
5	9.534	BB	0.7152	1.04569e4	212.48177	98.5897

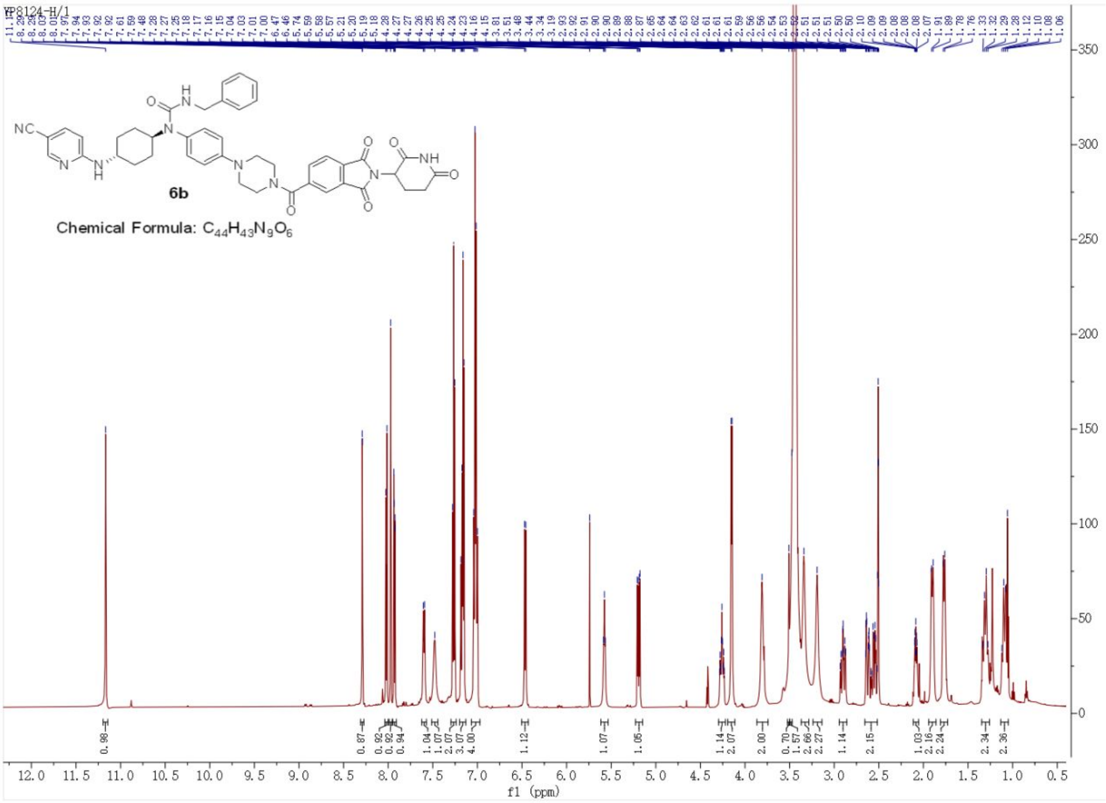
YP8124-H/1
6b
Chemical Formula: C44H43N9O6
f1 (ppm)

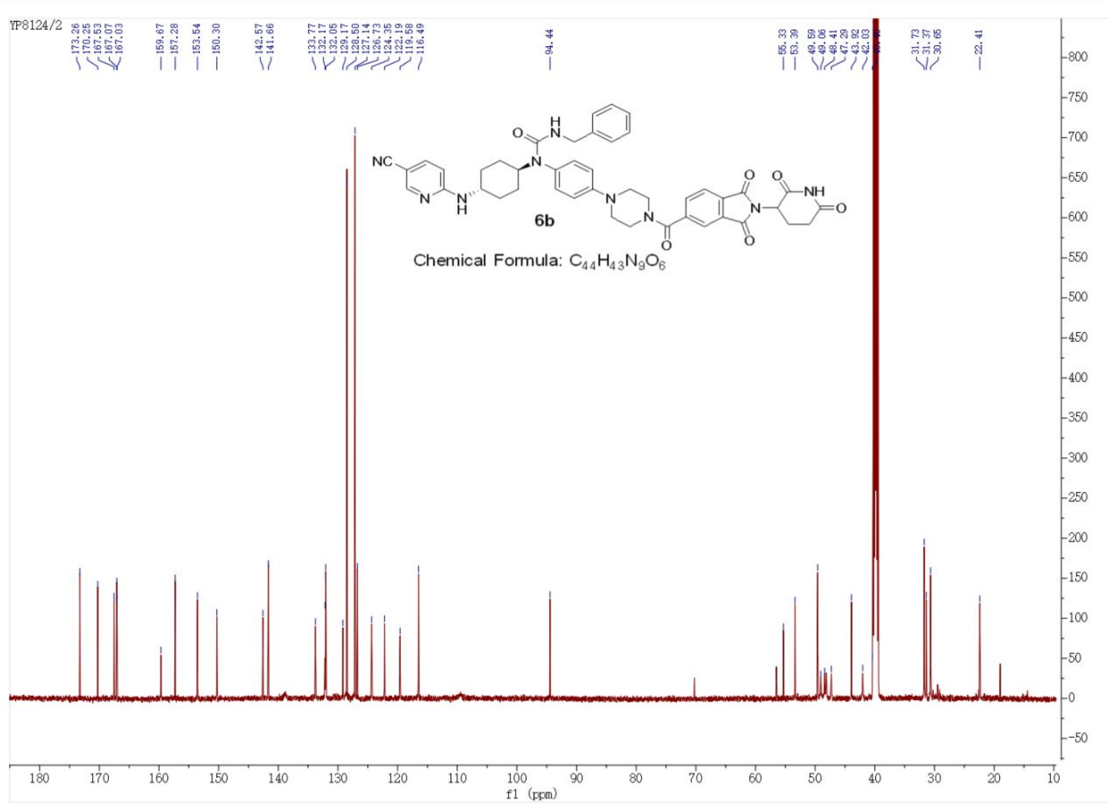
YP8124/2
6b
Chemical Formula: C44H43N9O6
f1 (ppm)

Hit	Formula	m/z	RDB	ppm	MS Rank	MSMS ppm	MSMS Rank	Found
1	C44H43N9O6	794.3409	28.0	-3.2	1			NA/NA

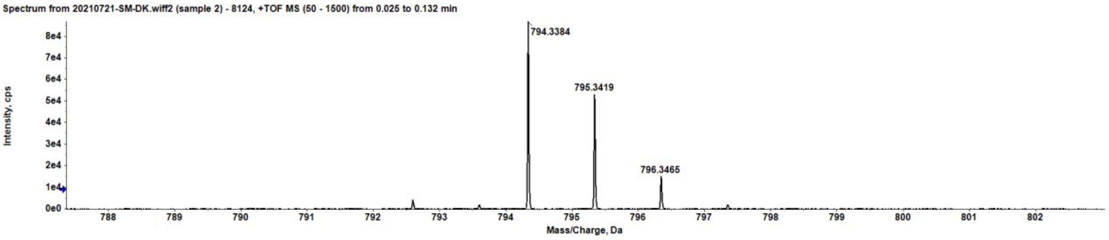


```
Data File E:\DK\YJZ\data\20210624-ZZ-YJZ-ZFT\YJZ1901DEF_LC 2021-06-26 14-59-43\7124.D
Sample Name: 8124

    =====================================================================
    Acq. Operator   : 系统                          Seq. Line :   6
    Acq. Instrument : 1260LC                         Location :   26
    Injection Date  : 26/06/2021 17:51:59                 Inj :   1
                                                   Inj Volume : 15.000 µl
    Different Inj Volume from Sample Entry!  Actual Inj Volume : 5.000 µl
    Method          : E:\DK\YJZ\data\20210624-ZZ-YJZ-ZFT\YJZ1901DEF_LC 2021-06-26 14-59-43\75C-
                      25A-30MIN-.M (Sequence Method)
    Last changed    : 26/06/2021 15:02:42 by 系统
```

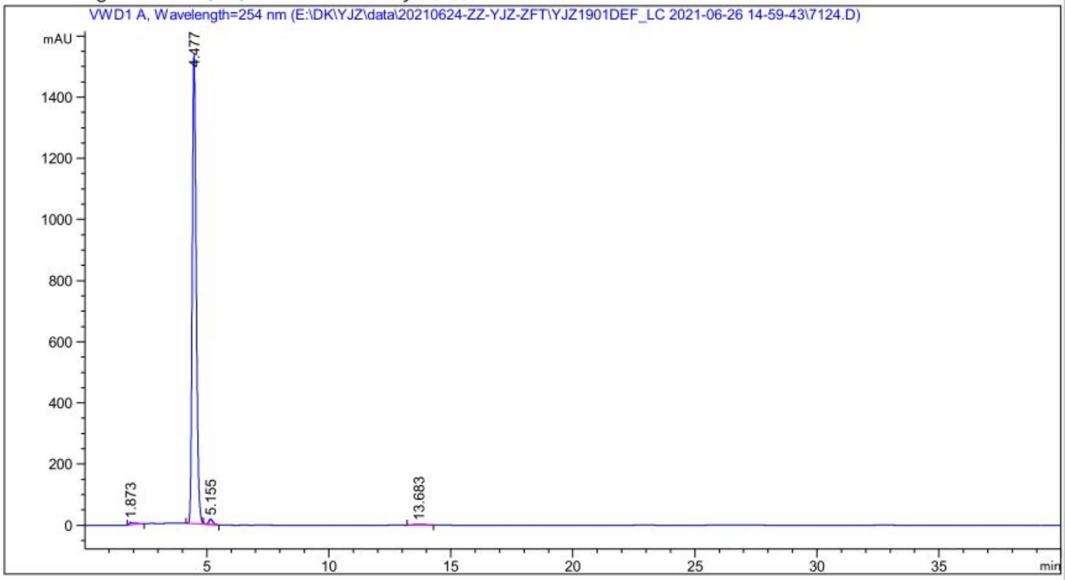


```
    =====================================================================
                         Area Percent Report
    =====================================================================

    Sorted By             :      Signal
    Multiplier            :      1.0000
    Dilution              :      1.0000
    Use Multiplier & Dilution Factor with ISTDs


    Signal 1: VWD1 A, Wavelength=254 nm

    Peak RetTime Type  Width     Area      Height     Area
      #   [min]        [min]   [mAU*s]     [mAU]        %
    ----|-------|----|-------|----------|----------|--------|
       1   1.873 BB    0.1929  121.53476    8.29893   0.6890
       2   4.477 BV R  0.1721 1.72375e4  1534.27258  97.7285
       3   5.155 VB E  0.1775  204.37643   17.33623   1.1587
       4  13.683 BB    0.4433   74.74305    2.54718   0.4238
```

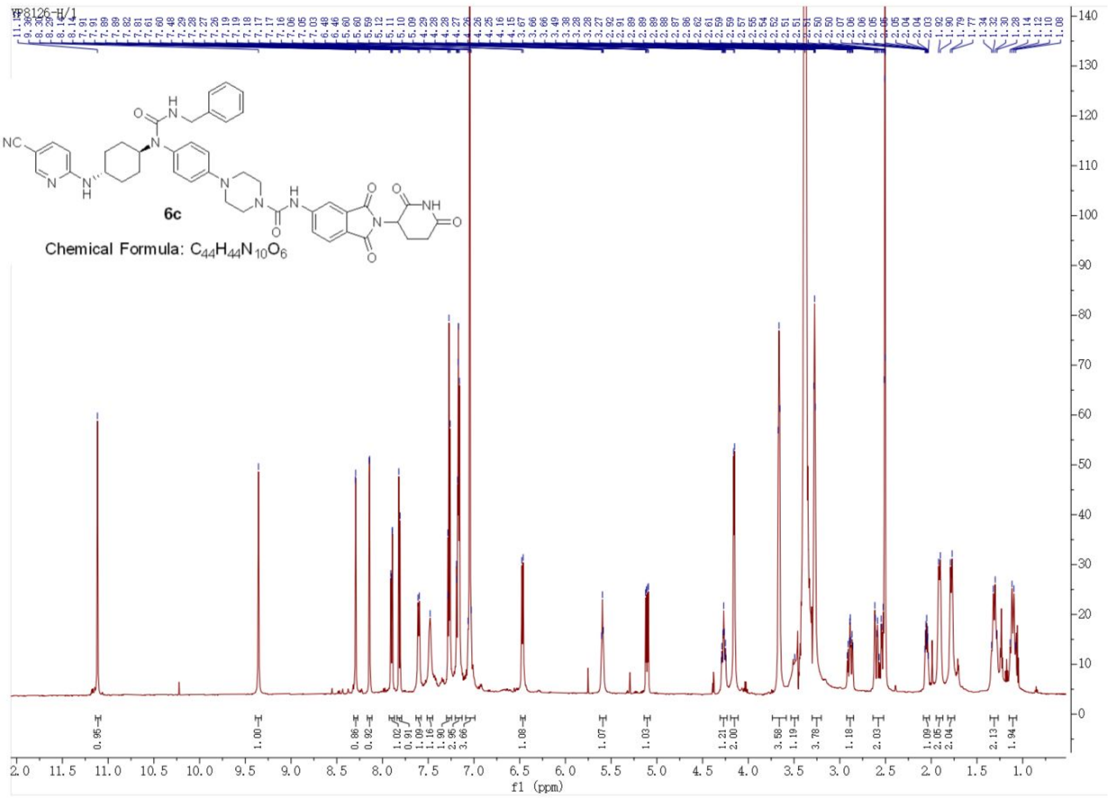
YP8126-H/1
6c
Chemical Formula: C44H44N10O6
f1 (ppm)

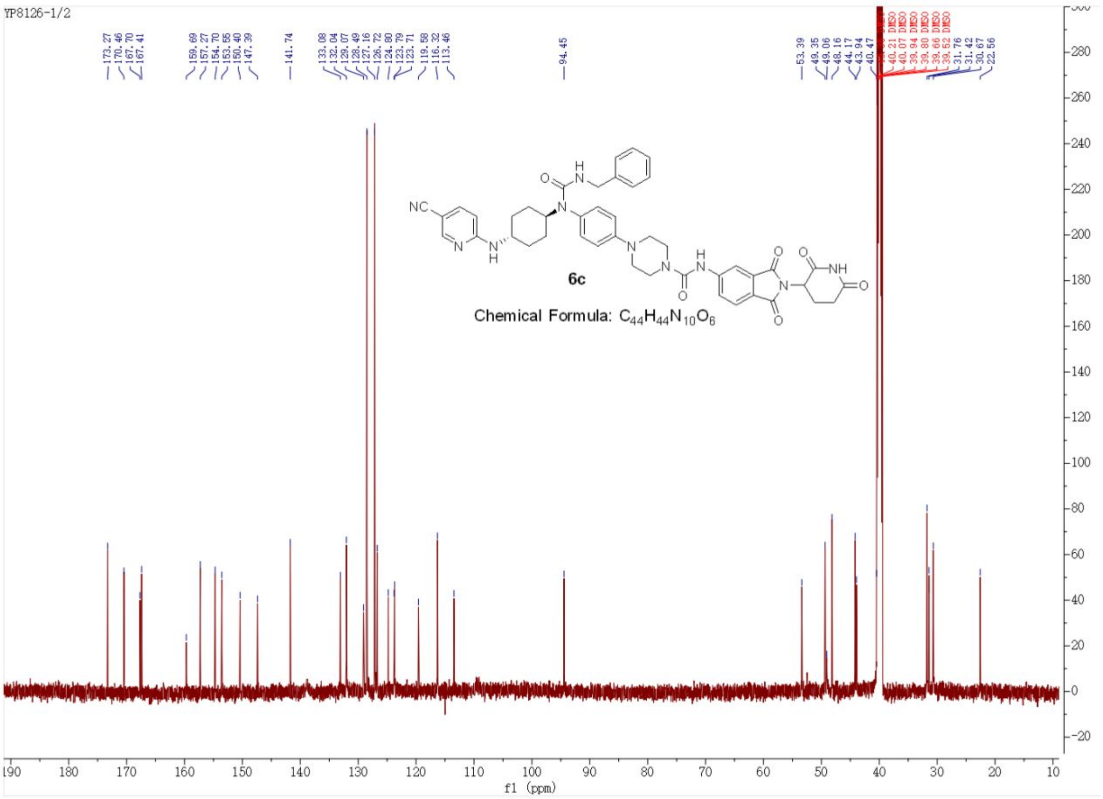
YP8126-1/2
6c
Chemical Formula: C44H44N10O6
f1 (ppm)

Hit	Formula	m/z	RDB	ppm	MS Rank	MSMS ppm	MSMS Rank	Found
1	C44H44N10O6	809.3518	28.0	-3.5	1			NA/NA

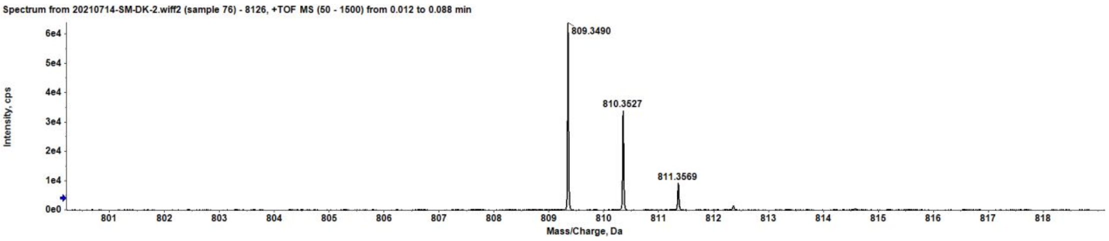

Data File E:\DK\YJZ\data\20210130\81262021-01-3115-51-00.D
Sample Name: 8126

===
Acq. Operator : 系统
Sample Operator : 系统
Acq. Instrument : 1260LC Location : 3
Injection Date : 31/01/2021 15:51:41
Inj Volume : 10.000 µl
Acq. Method : E:\DK\TL\方法\75C-25A-30min-1u.M
Last changed : 26/01/2021 10:20:57 by 系统
Analysis Method : E:\DK\TL\方法\80C-20A-30min-1u.M
Last changed : 06/04/2021 10:23:35 by 系统

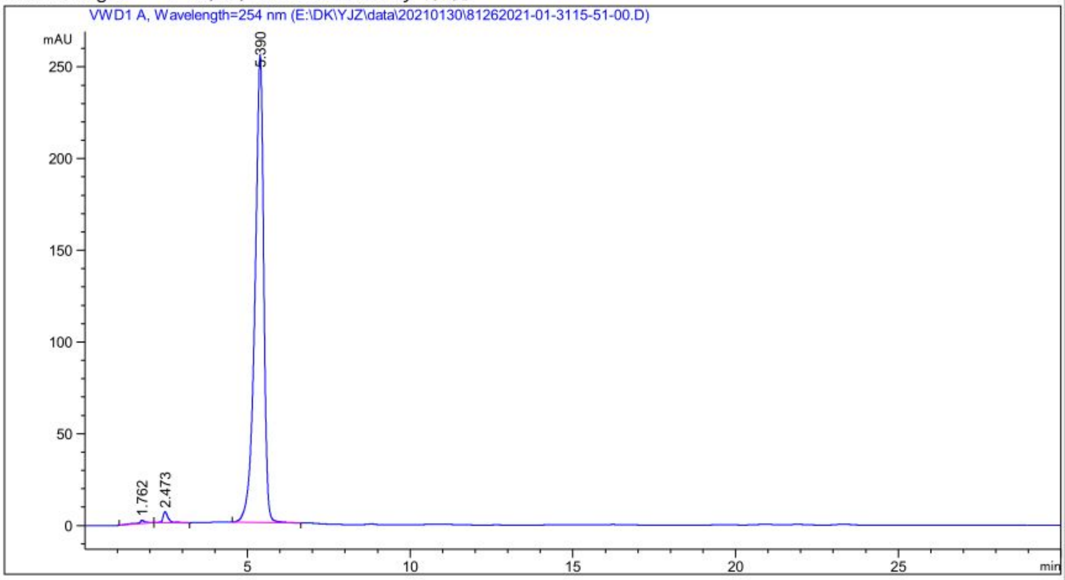

===
Area Percent Report
===

Sorted By : Signal
Multiplier : 1.0000
Dilution : 1.0000
Sample Amount: : 10.00000 [ng/ul] (not used in calc.)
Use Multiplier & Dilution Factor with ISTDs

Signal 1: VWD1 A, Wavelength=254 nm

Peak #	RetTime [min]	Type	Width [min]	Area [mAU*s]	Height [mAU]	Area %
1	1.762	BB	0.2033	26.53276	1.73607	0.5471
2	2.473	BV R	0.1684	65.40930	5.89663	1.3486
3	5.390	BB	0.2758	4758.06250	254.92548	98.1043

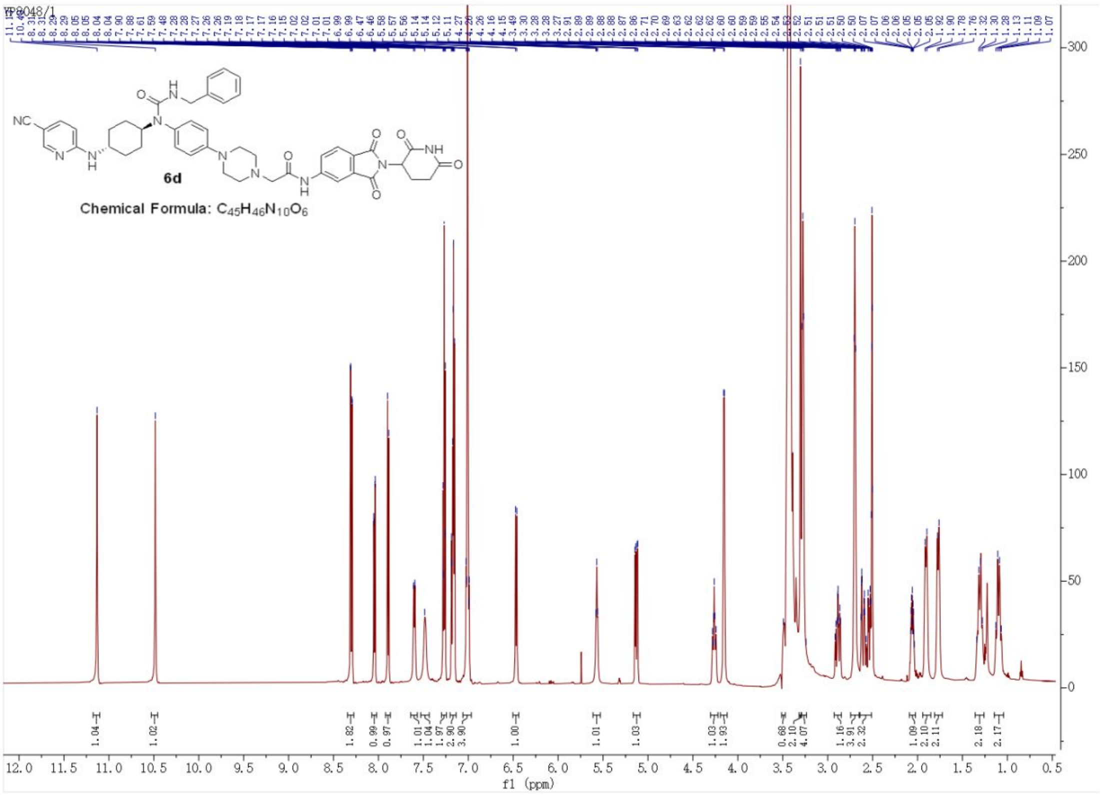

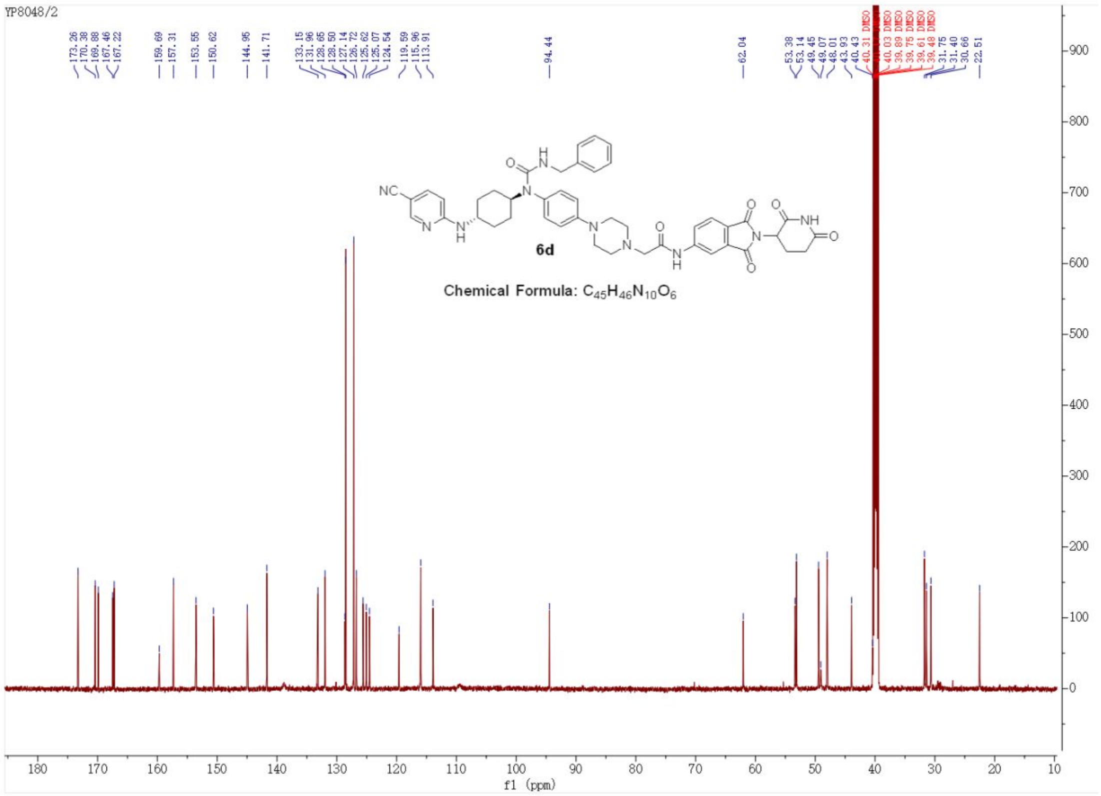

Hit	Formula	m/z	RDB	ppm	MS Rank	MSMS ppm	MSMS Rank	Found
1	C45H46N10O6	823.3675	28.0	-1.2	1			NA/NA

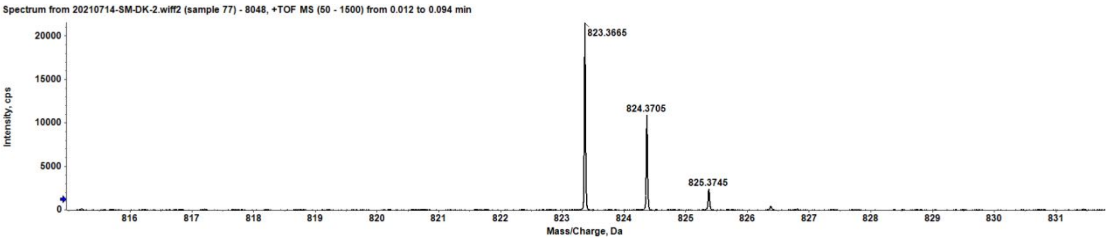

Data File E:\DK\ZZ\20210624-ZZ-YJZ-ZFT\YJZ1901DEF_LC 2021-06-24 20-23-22\8048.D
Sample Name: 8048

```
=====================================================================
Acq. Operator   : 系统                          Seq. Line :  55
Acq. Instrument : 1260LC                         Location :   23
Injection Date  : 26/06/2021 02:08:05                 Inj :   1
                                              Inj Volume : 10.000 µl
Method          : E:\DK\ZZ\20210624-ZZ-YJZ-ZFT\YJZ1901DEF_LC 2021-06-24 20-23-22\75C-25A-
                  30min-1u.M (Sequence Method)
Last changed    : 24/06/2021 20:23:22 by 系统
```

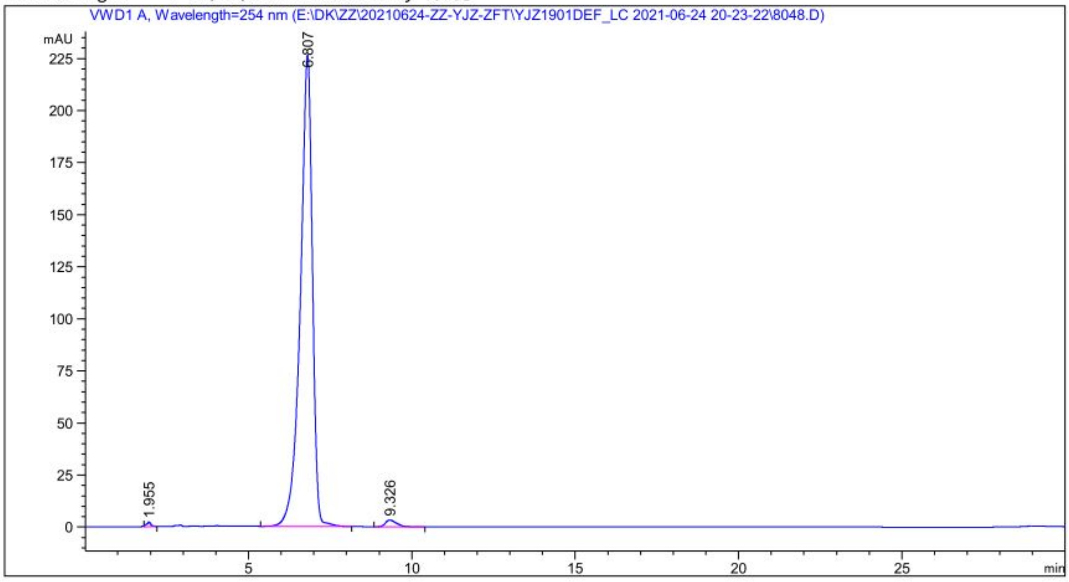


```
=====================================================================
                         Area Percent Report
=====================================================================

Sorted By             :      Signal
Multiplier            :      1.0000
Dilution              :      1.0000
Use Multiplier & Dilution Factor with ISTDs


Signal 1: VWD1 A, Wavelength=254 nm

Peak RetTime Type  Width     Area      Height     Area
  #   [min]        [min]   [mAU*s]     [mAU]        %
----|-------|----|-------|----------|----------|--------|
   1   1.955 VB    0.1319   20.01968    2.14474   0.3388
   2   6.807 BB    0.3784 5811.97119  226.46126  98.3484
   3   9.326 BB    0.3462   77.58080    3.27686   1.3128
```

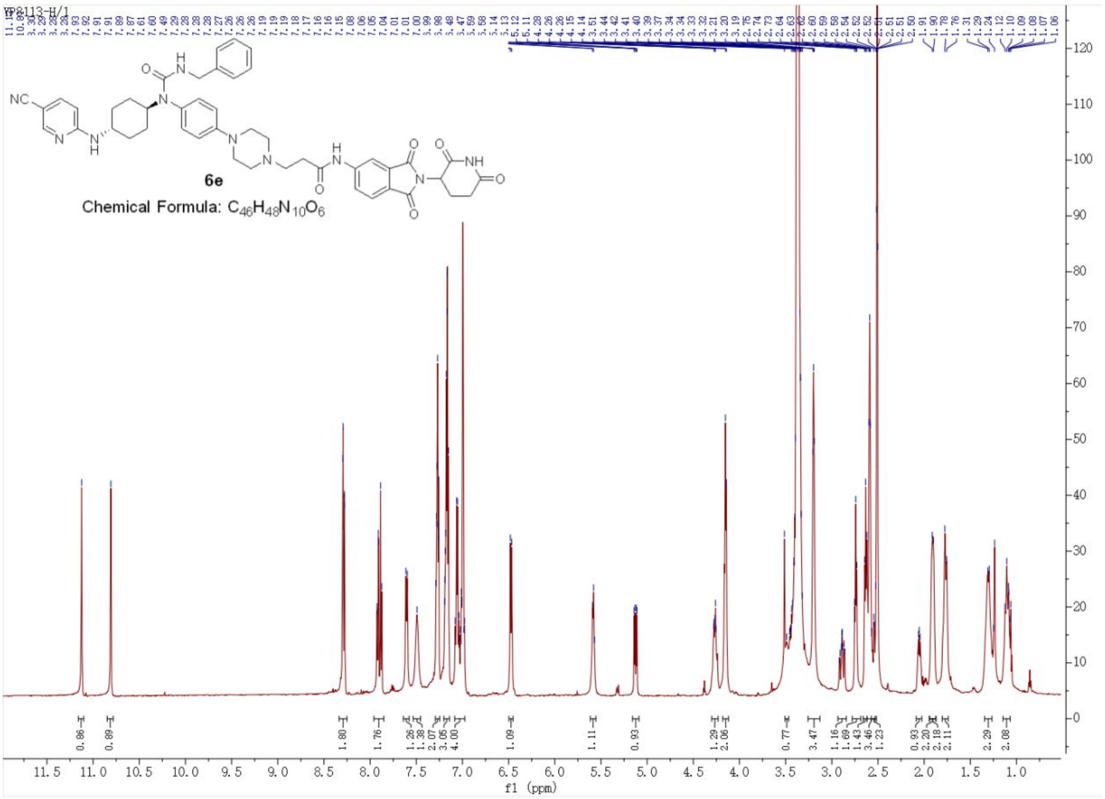
YP8113-H/1
NC
N
H
O
6e
Chemical Formula: C46H48N10O6
f1 (ppm)

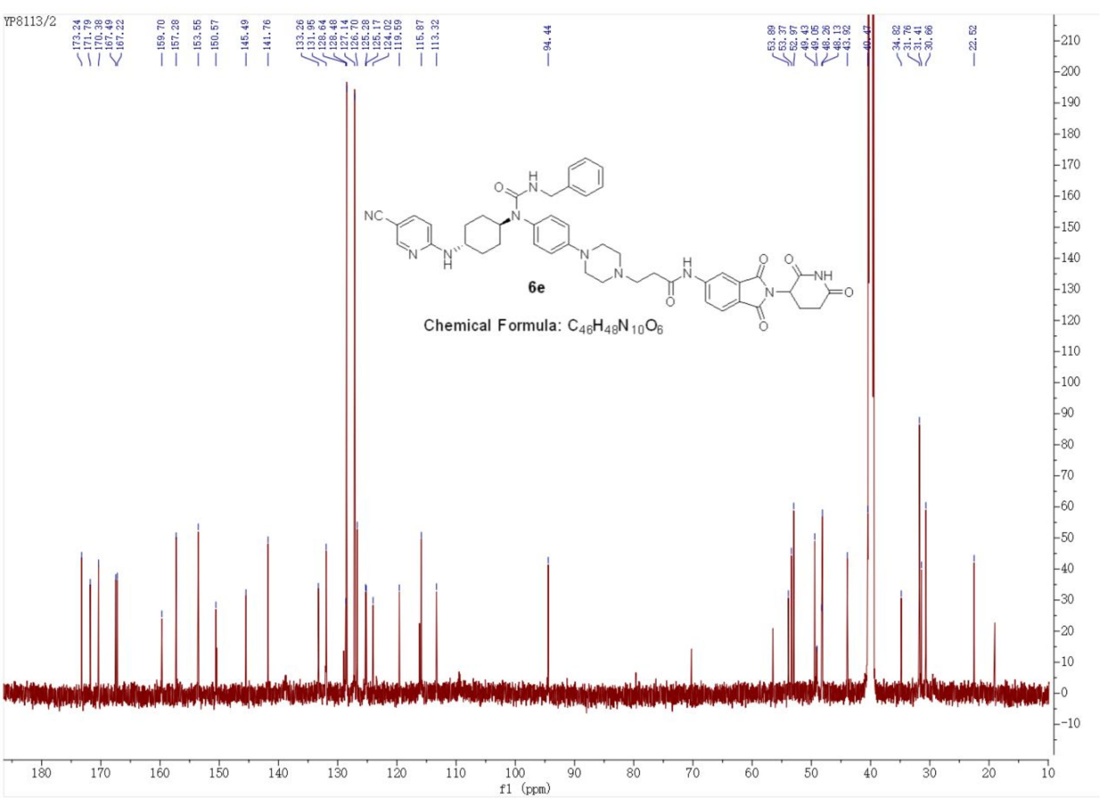
YP8113/2
NC
N
H
O
6e
Chemical Formula: C46H48N10O6
f1 (ppm)

Hit	Formula	m/z	RDB	ppm	MS Rank	MSMS ppm	MSMS Rank	Found
1	C46H48N10O6	837.3831	28.0	-1.6	1			NA/NA

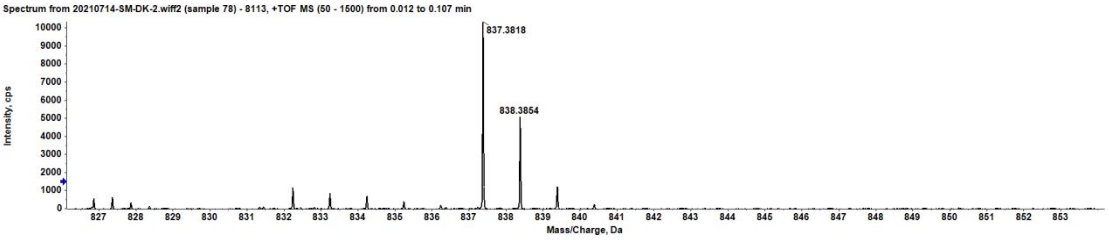


```
Data File E:\DK\YJZ\data\20210130\81132021-01-3116-42-07.D
Sample Name: 8113

    =====================================================================
    Acq. Operator   : 系统
    Sample Operator : 系统
    Acq. Instrument : 1260LC                          Location :   12
    Injection Date  : 31/01/2021 16:42:58
                                                    Inj Volume : 10.000 µl
    Acq. Method     : E:\DK\TL\方法\75C-25A-30min-1u.M
    Last changed    : 26/01/2021 10:20:57 by 系统
    Analysis Method : E:\DK\TL\方法\80C-20A-30min-1u.M
    Last changed    : 06/04/2021 10:23:35 by 系统
```

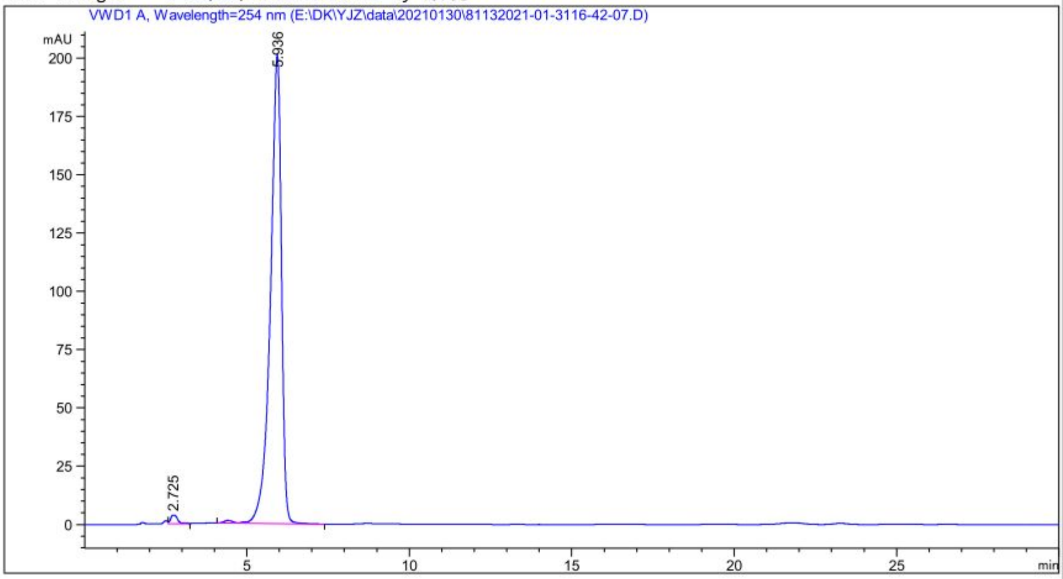


```
    =====================================================================
                            Area Percent Report
    =====================================================================

    Sorted By             :      Signal
    Multiplier            :      1.0000
    Dilution              :      1.0000
    Sample Amount:               :      10.00000  [ng/ul]   (not used in calc.)
    Use Multiplier & Dilution Factor with ISTDs


    Signal 1: VWD1 A, Wavelength=254 nm

    Peak RetTime Type  Width     Area      Height     Area
      #   [min]        [min]   [mAU*s]     [mAU]       %
    ----|-------|----|-------|----------|----------|--------|
       1   2.725 VV R  0.2064   56.09426    3.58825   1.1672
       2   5.936 VB R  0.3515 4749.77881  201.13483  98.8328
```

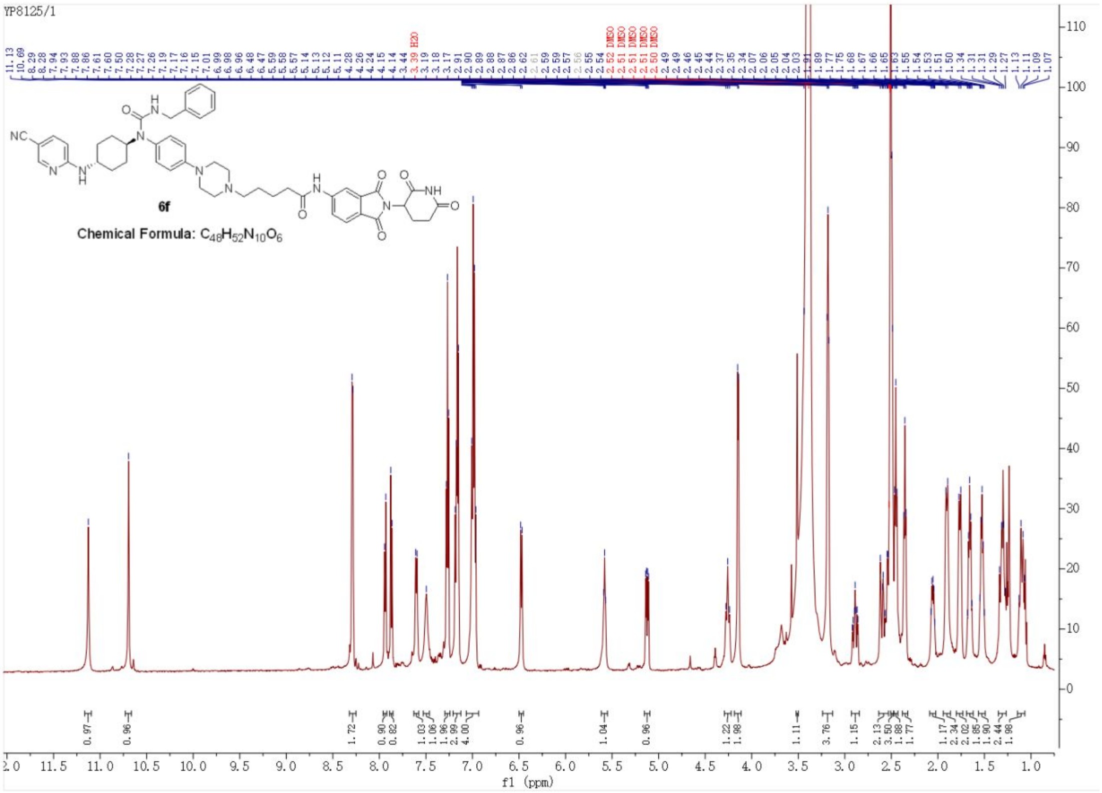

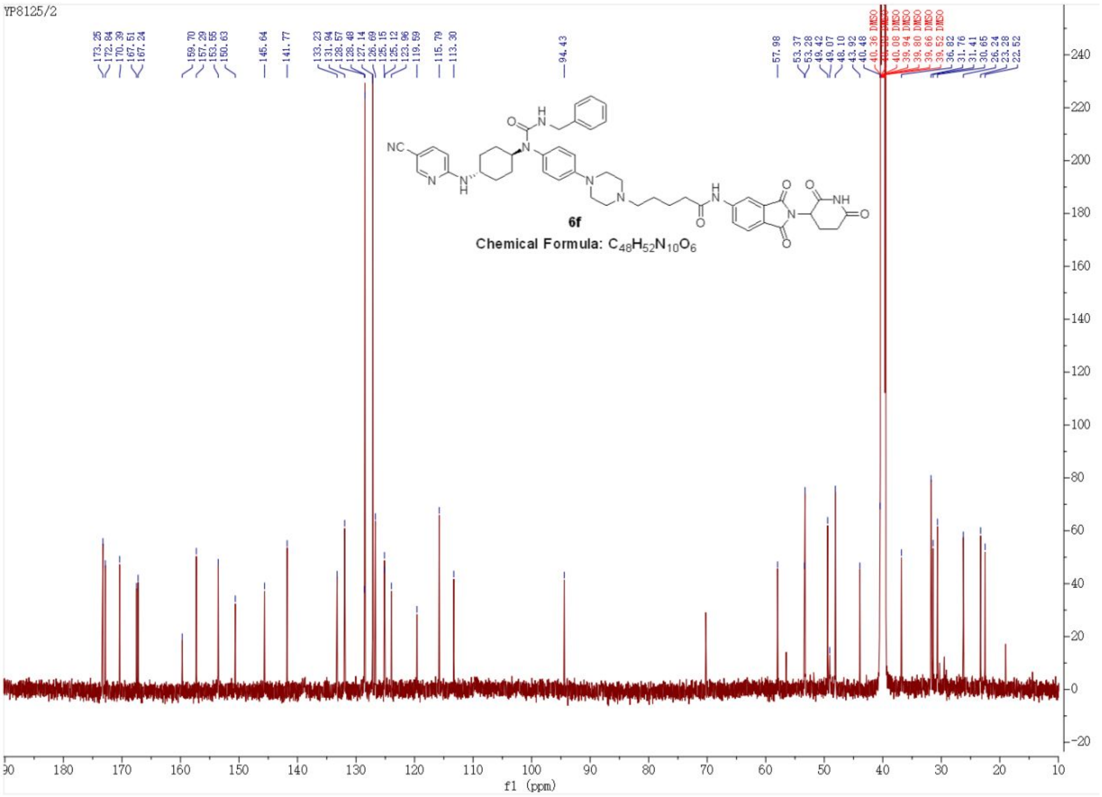

Hit	Formula	m/z	RDB	ppm	MS Rank	MSMS ppm	MSMS Rank	Found
1	C44H43N9O6	794.3409	28.0	-4.9	1			NA/NA

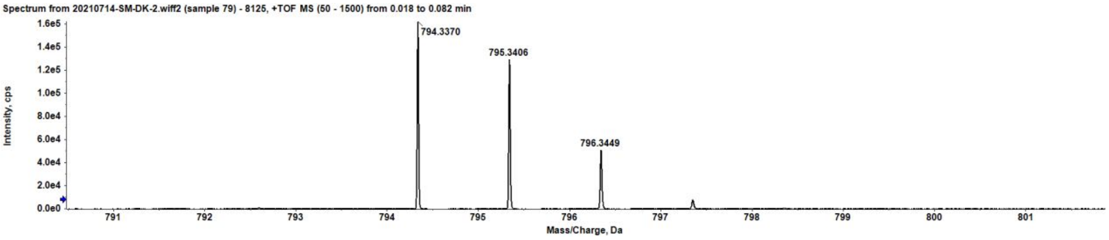

Data File E:\DK\YJZ\data\20210719\DELL-PC3880.D
Sample Name: 8125

===
Acq. Operator : 系统
Sample Operator : 系统
Acq. Instrument : 1260LC Location : 4
Injection Date : 20/07/2021 13:24:43
Inj Volume : 5.000 µl
Method : E:\DK\YJZ\METHOD\73B-27A-30min-1u.M
Last changed : 20/07/2021 13:21:41 by 系统
(modified after loading)

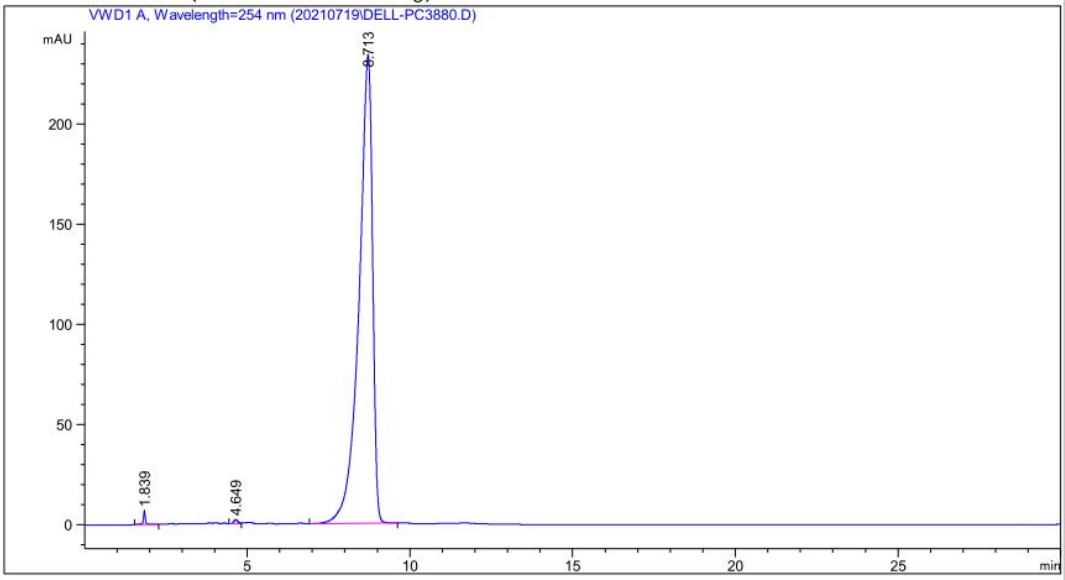

===
Area Percent Report
===

Sorted By : Signal
Multiplier : 1.0000
Dilution : 1.0000
Sample Amount: : 5.00000 [ng/ul] (not used in calc.)
Use Multiplier & Dilution Factor with ISTDs

Signal 1: VWD1 A, Wavelength=254 nm

Peak #	RetTime [min]	Type	Width [min]	Area [mAU*s]	Height [mAU]	Area %
1	1.839	VB R	0.0686	32.79169	6.88557	0.4808
2	4.649	BV	0.1485	19.77110	2.01385	0.2899
3	8.713	BB	0.4225	6767.38574	233.93098	99.2293

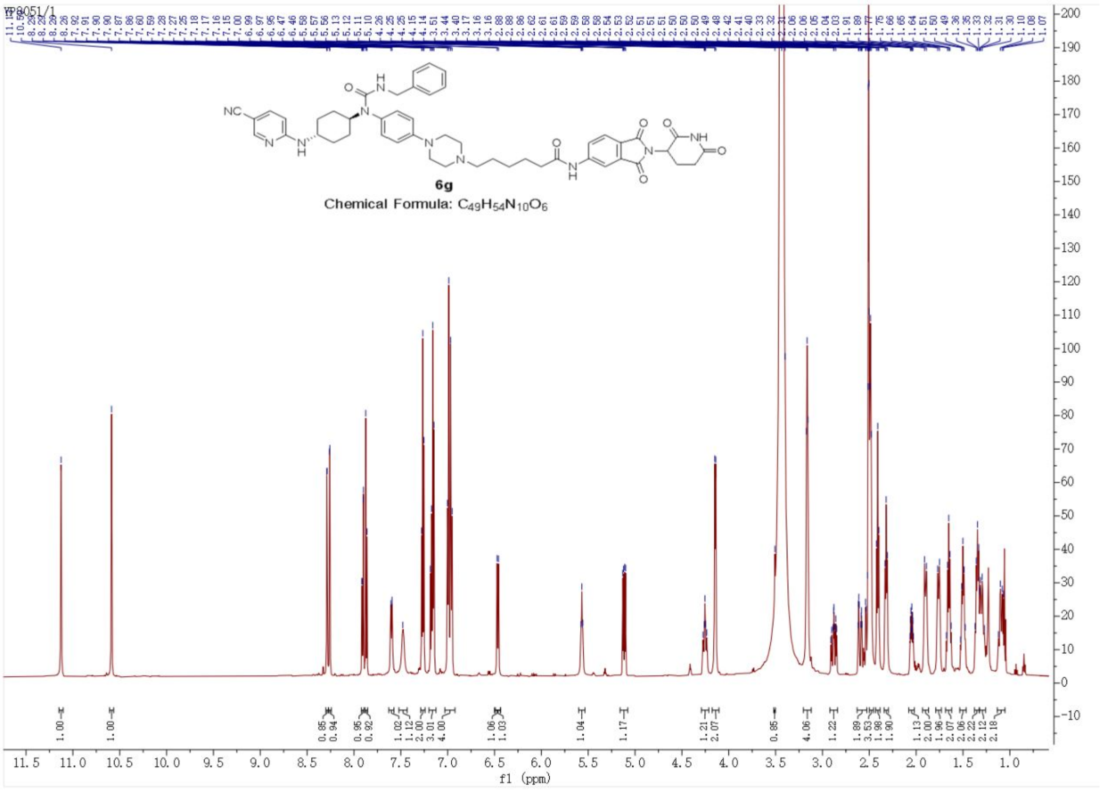
YP8051/1
6g
Chemical Formula: $C_{49}H_{54}N_{10}O_6$
f1 (ppm)

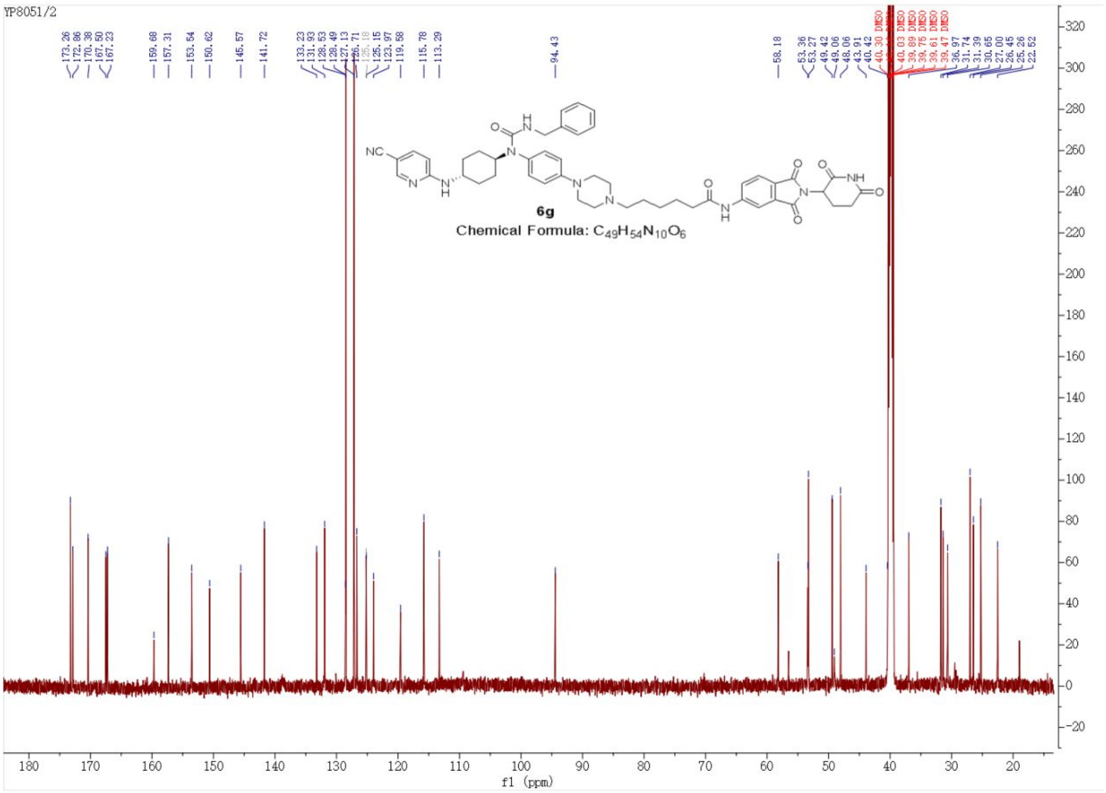
YP8051/2
6g
Chemical Formula: $C_{49}H_{54}N_{10}O_6$
f1 (ppm)

Hit	Formula	m/z	RDB	ppm	MS Rank	MSMS ppm	MSMS Rank	Found
1	C49H54N10O6	879.4301	28.0	-1.5	1			NA/NA

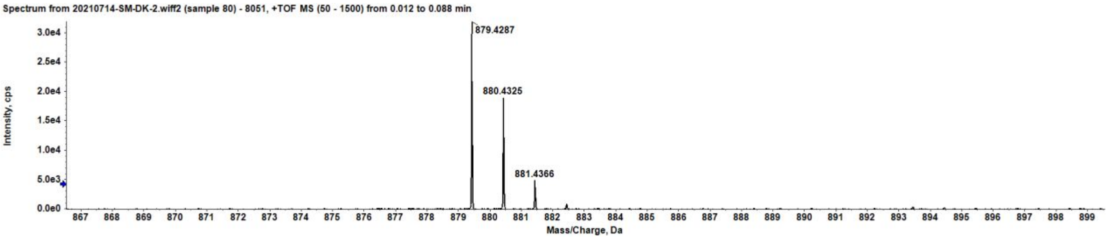


```
Data File E:\DK\ZZ\20210624-ZZ-YJZ-ZFT\YJZ1901DEF_LC 2021-06-24 20-23-22\8051.D
Sample Name: 8051

    =====================================================================
    Acq. Operator   : 系统                          Seq. Line :  58
    Acq. Instrument : 1260LC                         Location :   26
    Injection Date  : 26/06/2021 03:40:35                 Inj :   1
                                                   Inj Volume : 10.000 µl
    Method          : E:\DK\ZZ\20210624-ZZ-YJZ-ZFT\YJZ1901DEF_LC 2021-06-24 20-23-22\75C-25A-
                      30min-1u.M (Sequence Method)
    Last changed    : 24/06/2021 20:23:22 by 系统
```

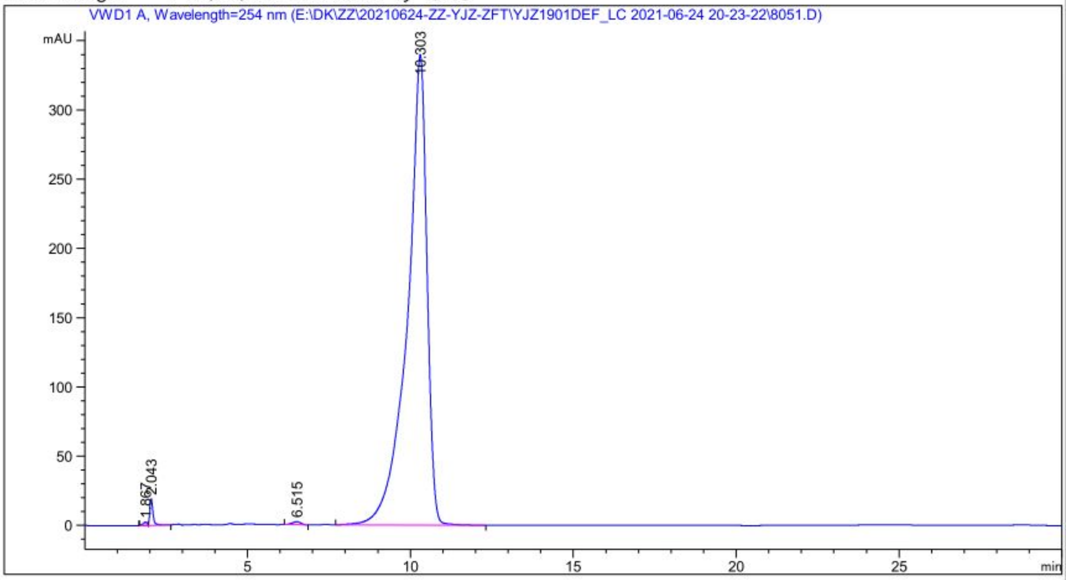


```
    =====================================================================
                             Area Percent Report
    =====================================================================

    Sorted By             :      Signal
    Multiplier            :      1.0000
    Dilution              :      1.0000
    Use Multiplier & Dilution Factor with ISTDs


    Signal 1: VWD1 A, Wavelength=254 nm

    Peak RetTime Type  Width     Area      Height     Area
      #   [min]        [min]   [mAU*s]     [mAU]        %
    ----|-------|----|-------|----------|----------|--------|
       1   1.867 BV E  0.1160   19.76631    2.45275   0.1378
       2   2.043 VB R  0.1079  134.77461   18.96876   0.9396
       3   6.515 BB    0.2519   38.51670    2.29363   0.2685
       4  10.303 BB    0.5839 1.41509e4  339.74838  98.6541
```

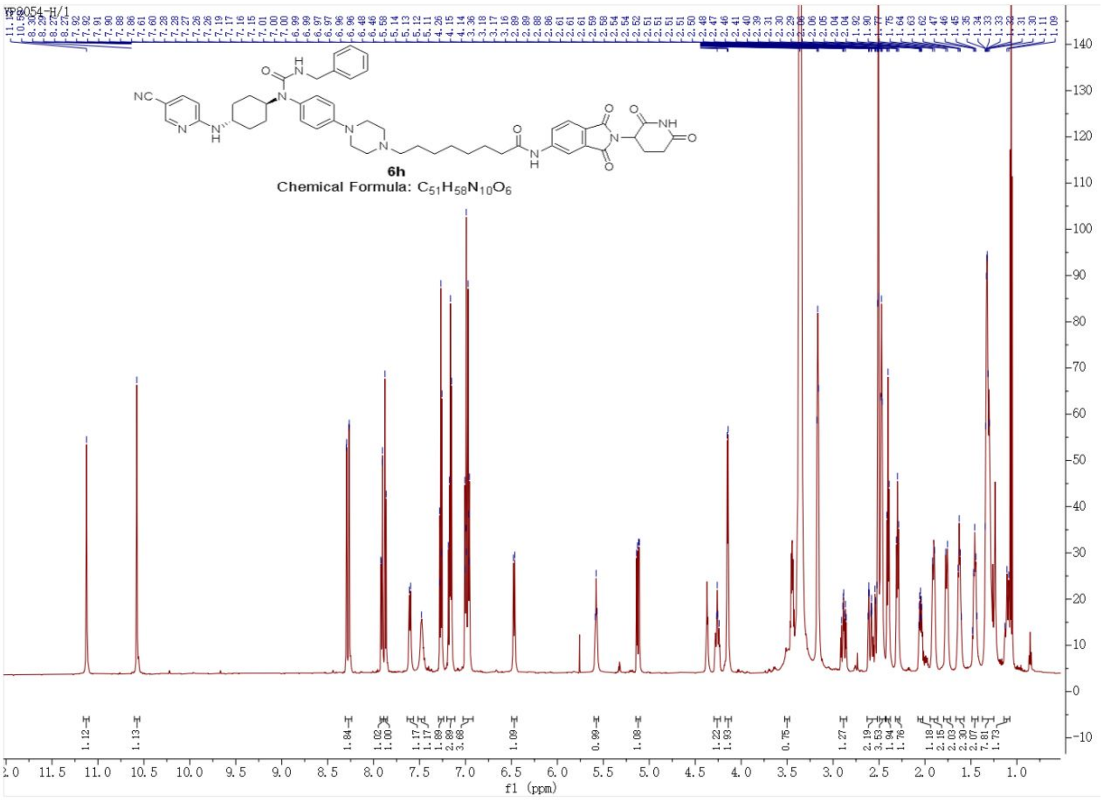

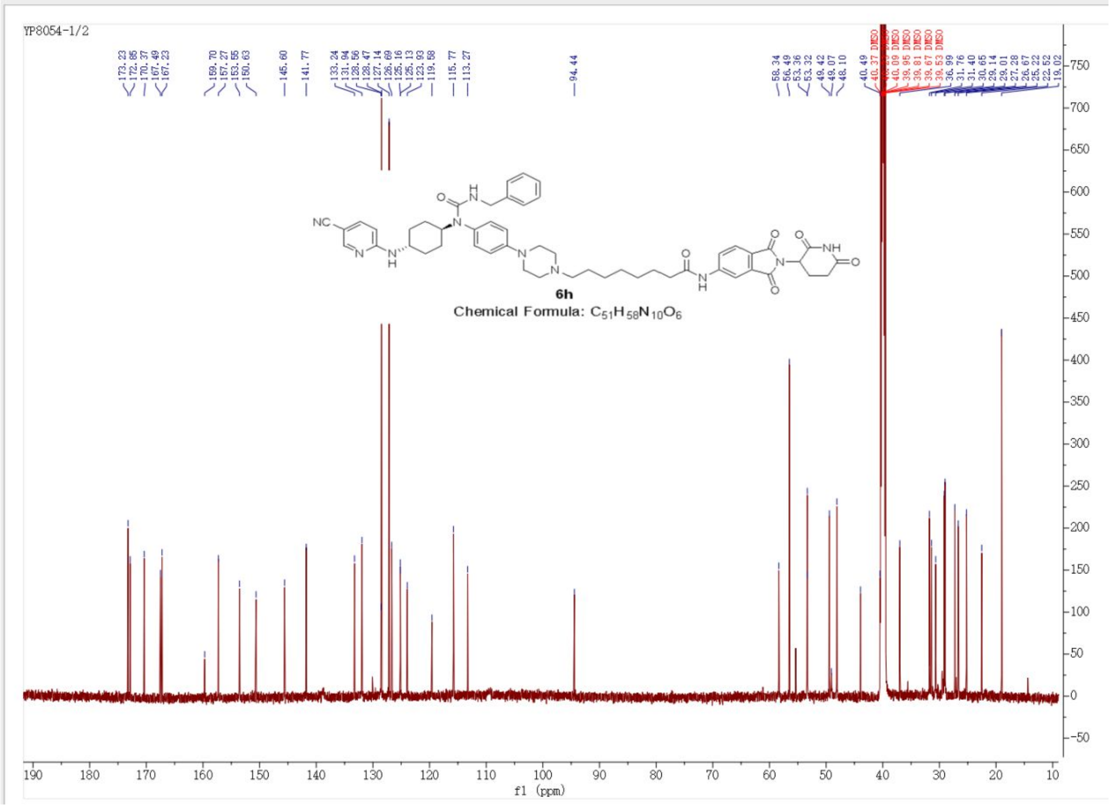

Hit	Formula	m/z	RDB	ppm	MS Rank	MSMS ppm	MSMS Rank	Found

	C51H58N1006	907.4614	28.0	-1.3	1			NA/NA

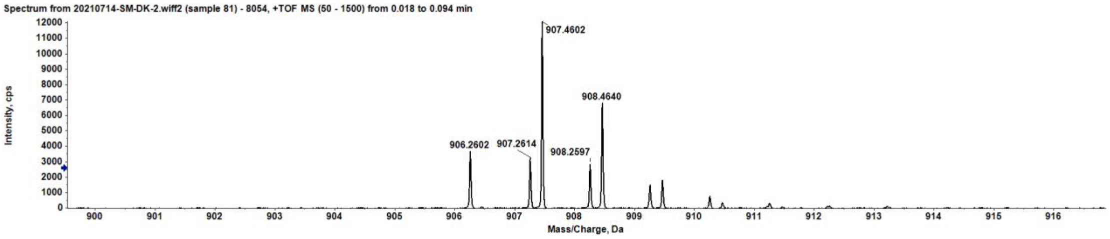


```
Data File E:\DK\YJZ\data\20210624\80543025.D
Sample Name: 8054

=====================================================================
Acq. Operator   : 系统
Sample Operator : 系统
Acq. Instrument : 1260LC                        Location :   98
Injection Date  : 26/06/2021 14:23:29
                                              Inj Volume : 5.000 µl
Acq. Method     : E:\DK\TL\方法\80C-20A-30min-1u.M
Last changed    : 26/06/2021 14:17:22 by 系统
                  (modified after loading)
Analysis Method : E:\DK\TL\方法\80C-20A-30min-1u.M
Last changed    : 06/04/2021 10:23:35 by 系统
Additional Info : Peak(s) manually integrated
```

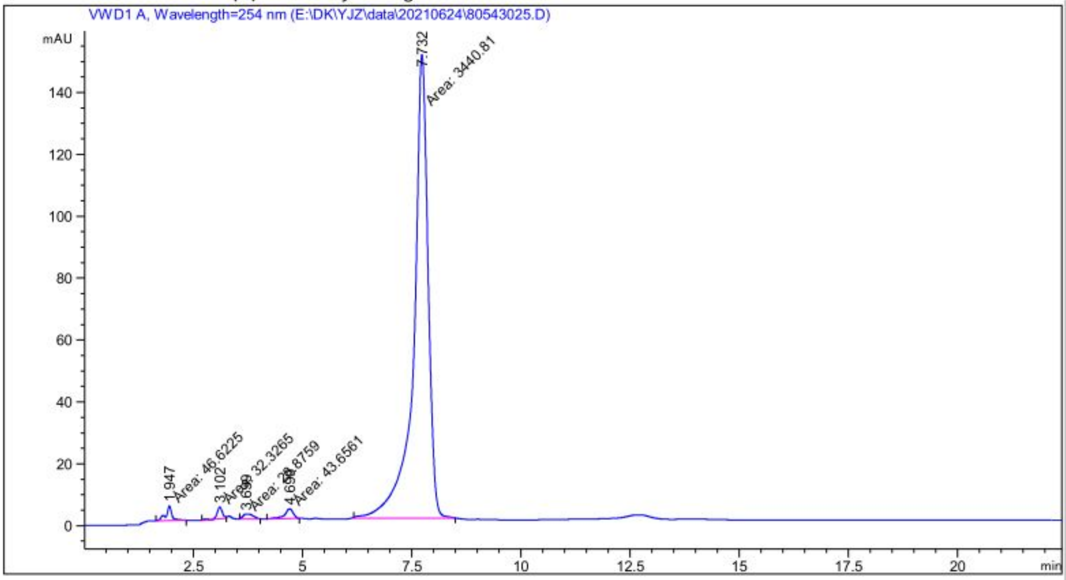


```
=====================================================================
                         Area Percent Report
=====================================================================

Sorted By             :      Signal
Multiplier            :      1.0000
Dilution              :      1.0000
Sample Amount:               :      10.00000  [ng/ul]   (not used in calc.)
Use Multiplier & Dilution Factor with ISTDs


Signal 1: VWD1 A, Wavelength=254 nm
```

Peak #	RetTime [min]	Type	Width [min]	Area [mAU*s]	Height [mAU]	Area %
1	1.947	MM	0.1623	46.62246	4.78820	1.2978
2	3.102	MM	0.1426	32.32653	3.77775	0.8999
3	3.699	MM	0.2803	28.87589	1.71698	0.8038
4	4.699	MM	0.2288	43.65606	3.18018	1.2153
5	7.732	MM	0.3824	3440.81177	149.96432	95.7832

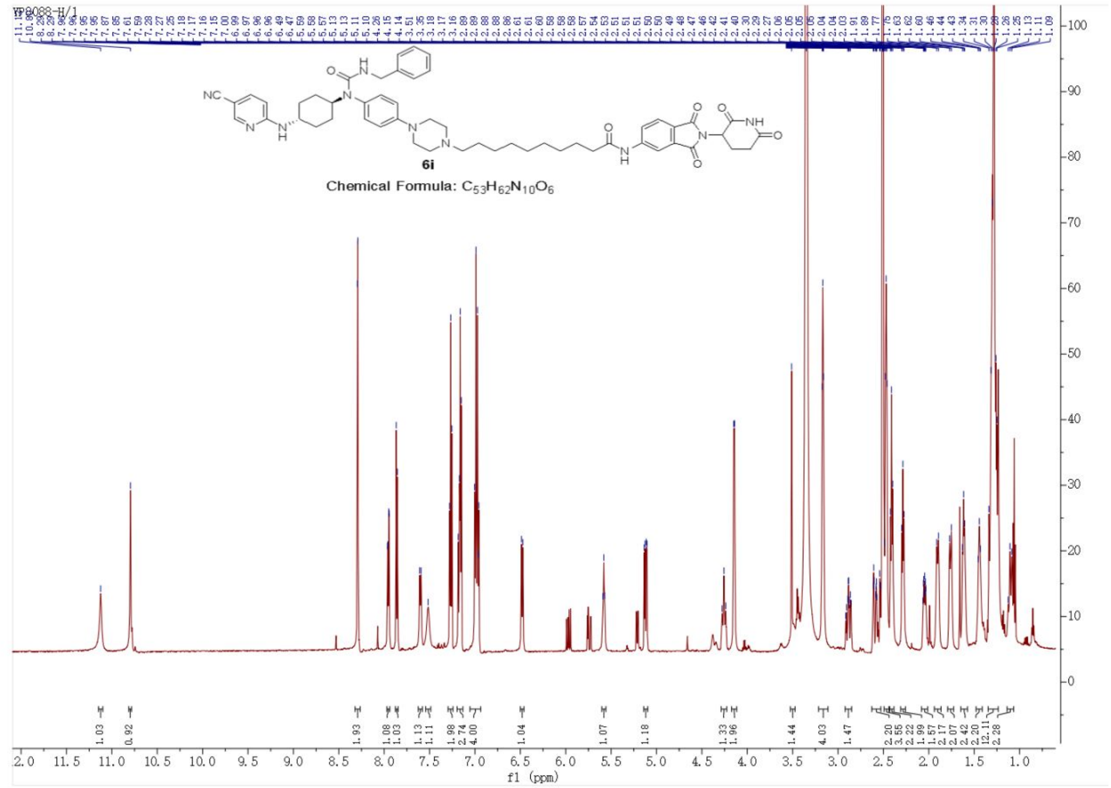

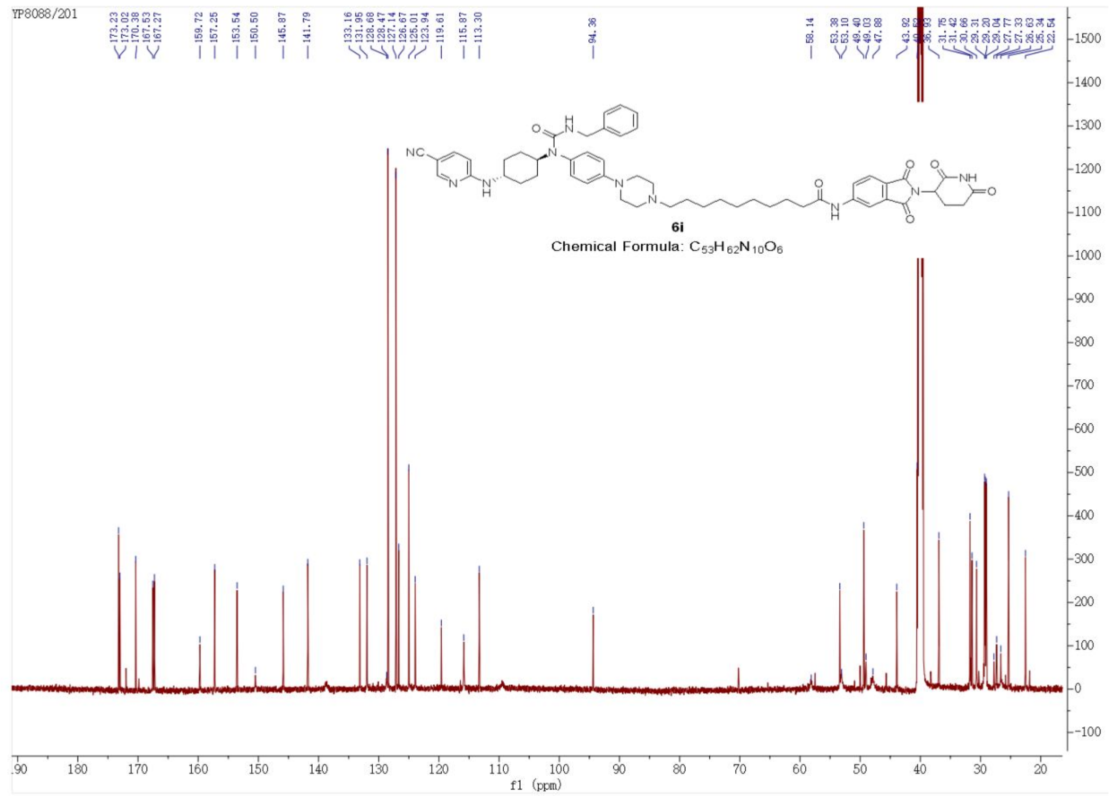

Hit	Formula	m/z	RDB	ppm	MS Rank	MSMS ppm	MSMS Rank	Found
1	C53H62N10O6	935.4927	28.0	-1.1	1			NA/NA

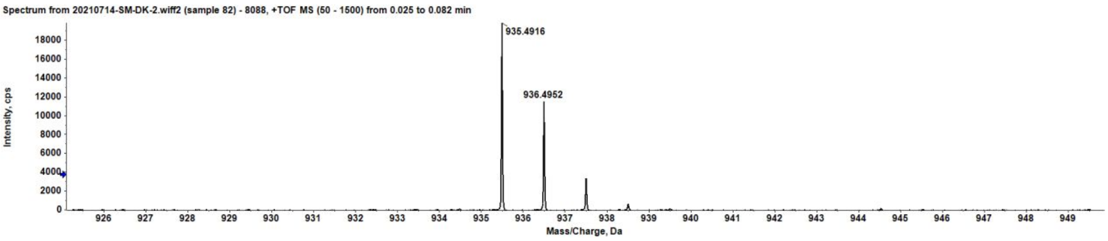


```
Data File E:\DK\YJZ\data\20210721\DELL-PC4105.D
Sample Name: 8088

=====================================================================
Acq. Operator   : 系统
Sample Operator : 系统
Acq. Instrument : 1260LC                        Location :   5
Injection Date  : 21/07/2021 17:58:47
                                              Inj Volume : 10.000 µl
Method          : E:\DK\TL\方法\80B-20A-30min-1u.M
Last changed    : 21/07/2021 17:46:06 by 系统
                  (modified after loading)
```

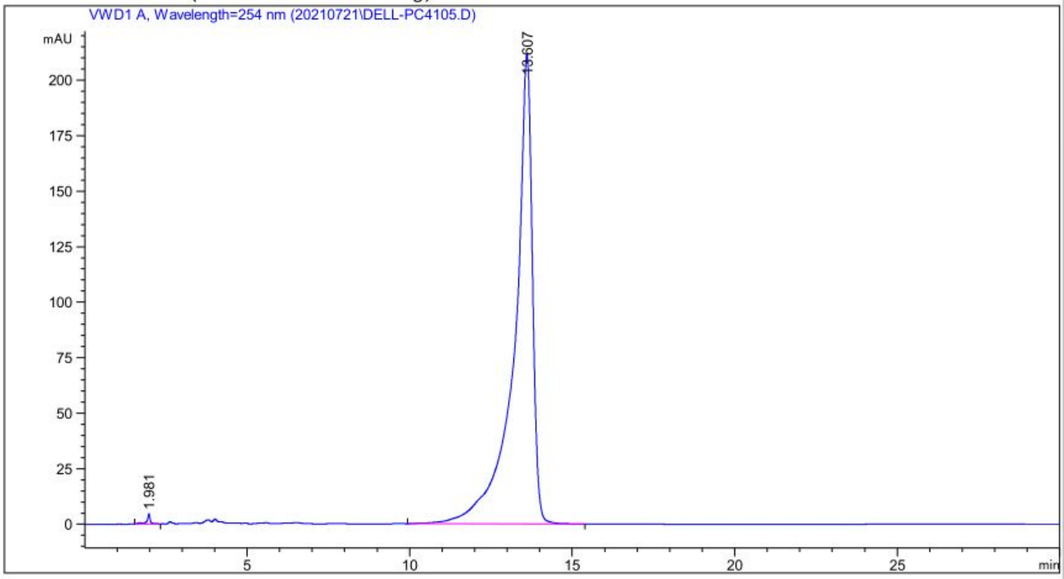


```
=====================================================================
                         Area Percent Report
=====================================================================

Sorted By             :      Signal
Multiplier            :      1.0000
Dilution              :      1.0000
Sample Amount:               :       5.00000  [ng/ul]   (not used in calc.)
Use Multiplier & Dilution Factor with ISTDs


Signal 1: VWD1 A, Wavelength=254 nm
```

Peak #	RetTime [min]	Type	Width [min]	Area [mAU*s]	Height [mAU]	Area %
1	1.981	VB R	0.1056	34.41807	4.53229	0.4186
2	13.607	BB	0.5367	8188.67773	211.48193	99.5814

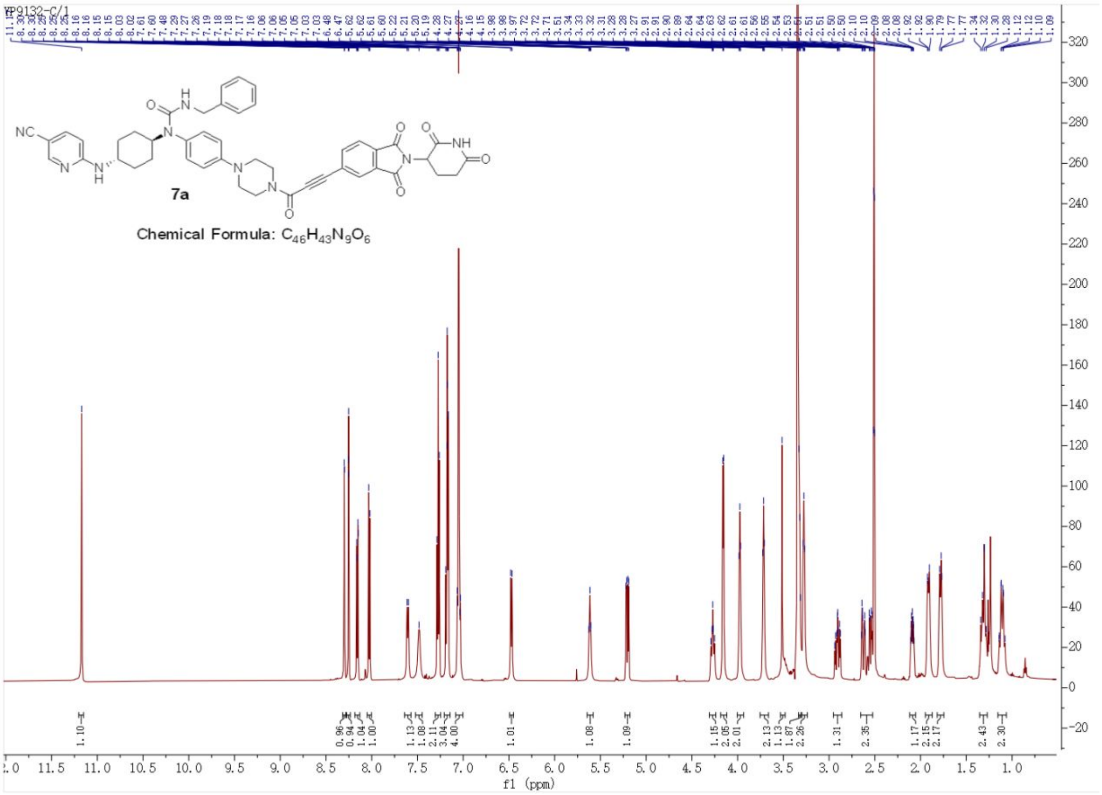

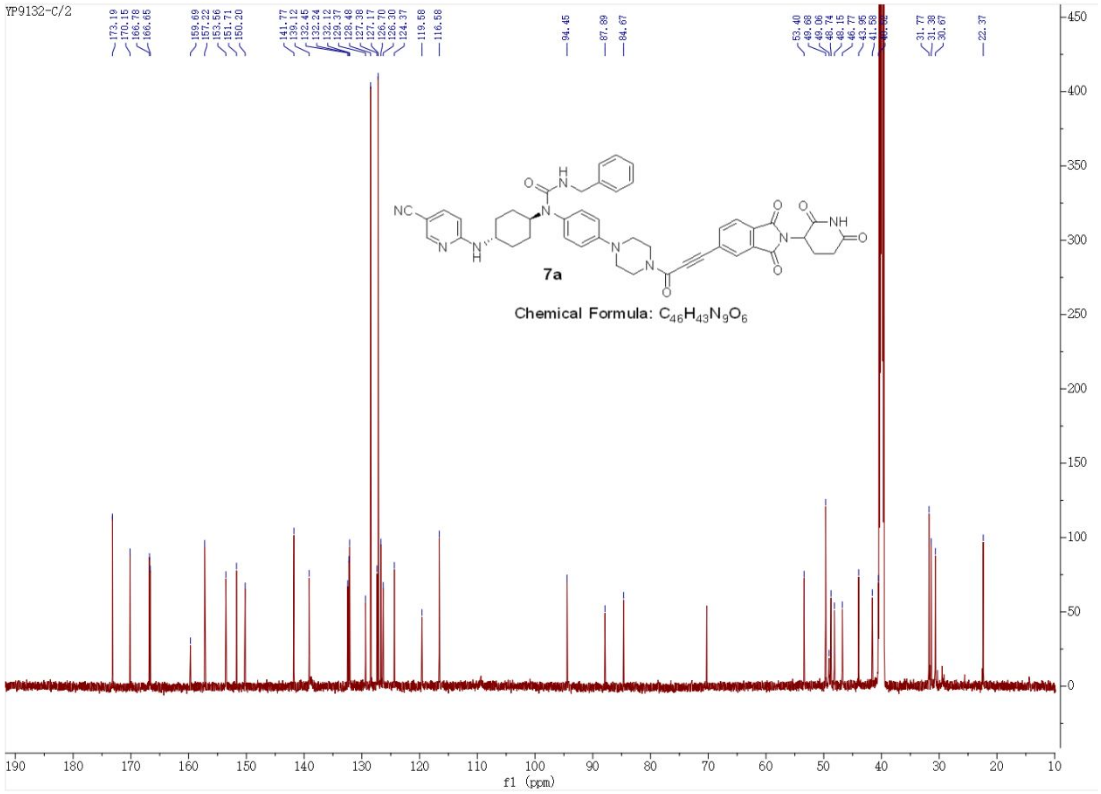

Hit	Formula	m/z	RDB	ppm	MS Rank	MSMS ppm	MSMS Rank	Found

1	C46H43N906	818.3409	30.0	-4.2	1			NA/NA

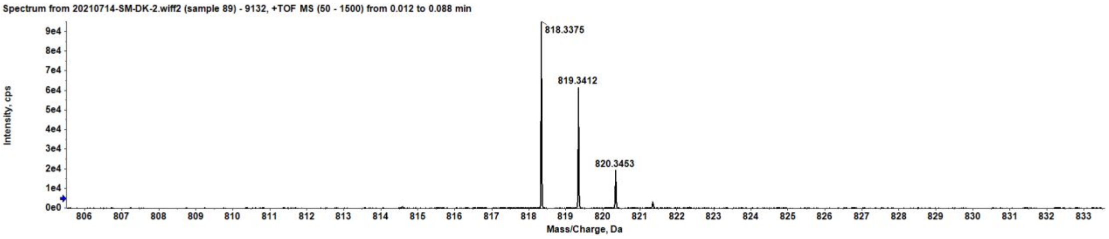

Data File E:\DK\ZZ\20210624-ZZ-YJZ-ZFT\YJZ1901DEF_LC 2021-06-24 20-23-22\9132.D
Sample Name: 9132

```
=====================================================================
Acq. Operator   : 系统                          Seq. Line :  18
Acq. Instrument : 1260LC                         Location :   17
Injection Date  : 25/06/2021 06:30:38                  Inj :   1
                                               Inj Volume : 10.000 µl
Different Inj Volume from Sample Entry!  Actual Inj Volume : 8.000 µl
Acq. Method     : E:\DK\ZZ\20210624-ZZ-YJZ-ZFT\YJZ1901DEF_LC 2021-06-24 20-23-22\75C-25A-
                  30min-1u.M
Last changed    : 24/06/2021 20:23:22 by 系统
Analysis Method : E:\DK\ZZ\20210624-ZZ-YJZ-ZFT\YJZ1901DEF_LC 2021-06-24 20-23-22\75C-25A-
                  30min-1u.M (Sequence Method)
Last changed    : 26/06/2021 10:55:14 by 系统
```

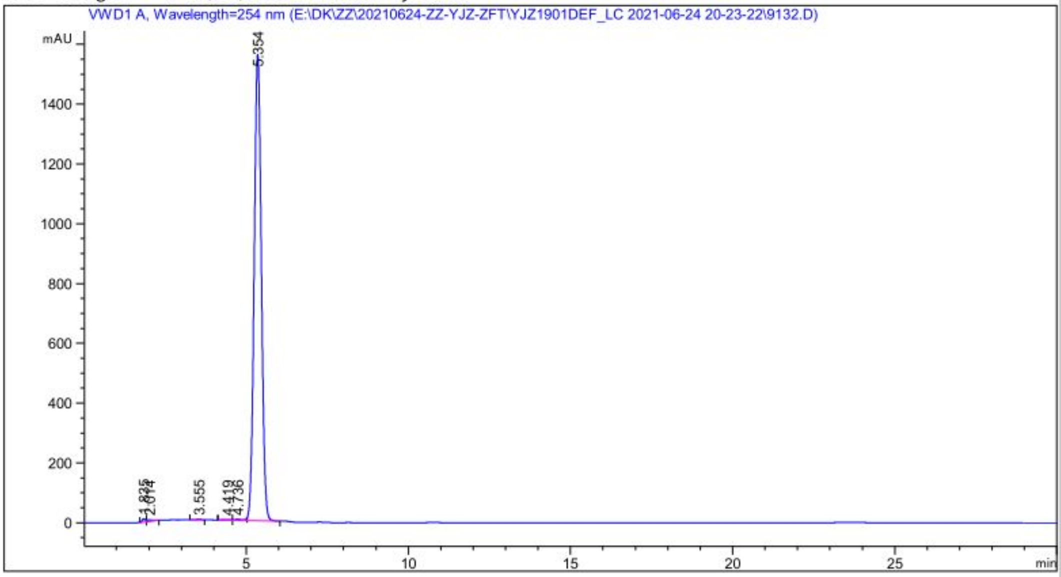


```
=====================================================================
                         Area Percent Report
=====================================================================

Sorted By             :      Signal
Multiplier            :      1.0000
Dilution              :      1.0000
Use Multiplier & Dilution Factor with ISTDs
```

Signal 1: VWD1 A, Wavelength=254 nm

Peak #	RetTime [min]	Type	Width [min]	Area [mAU*s]	Height [mAU]	Area %
1	1.835	BV	0.0986	70.01733	10.52607	0.2899
2	2.014	VB	0.1899	68.60731	4.91310	0.2840
3	3.555	BV	0.1678	25.11827	2.24088	0.1040
4	4.419	BV E	0.2793	36.67261	2.08396	0.1518
5	4.736	VV E	0.2467	61.37310	3.51439	0.2541
6	5.354	VB R	0.2386	2.38941e4	1561.19421	98.9163

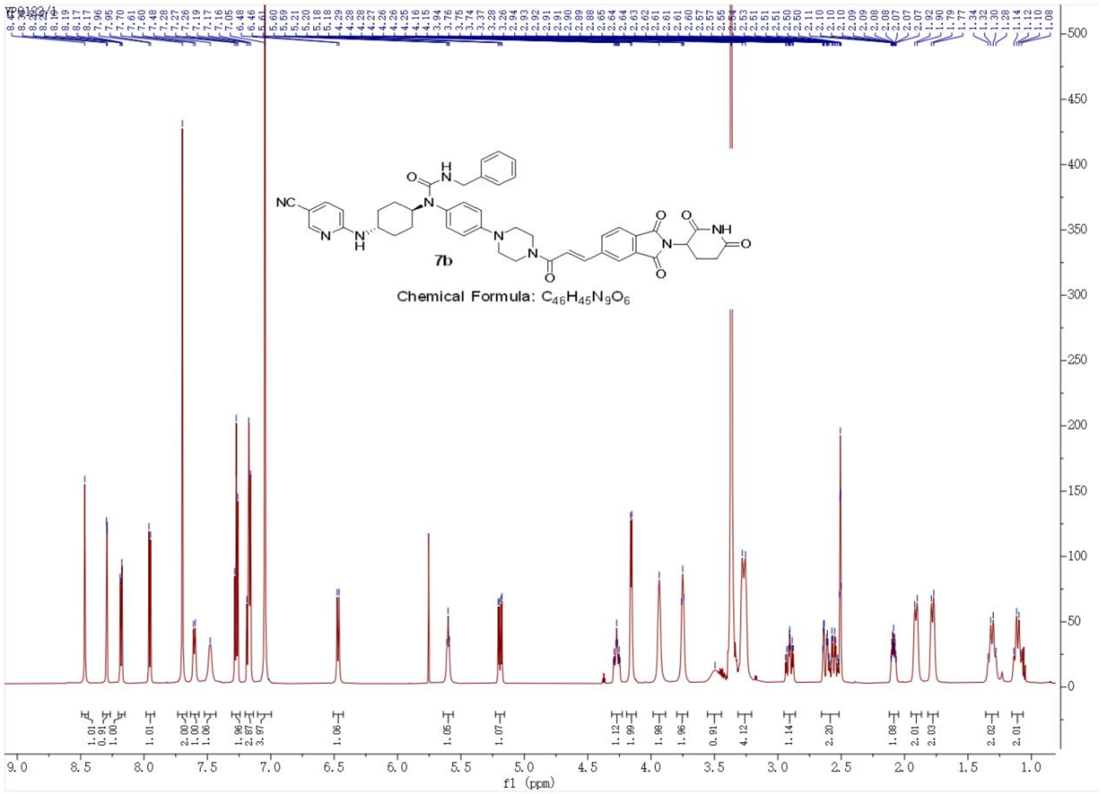

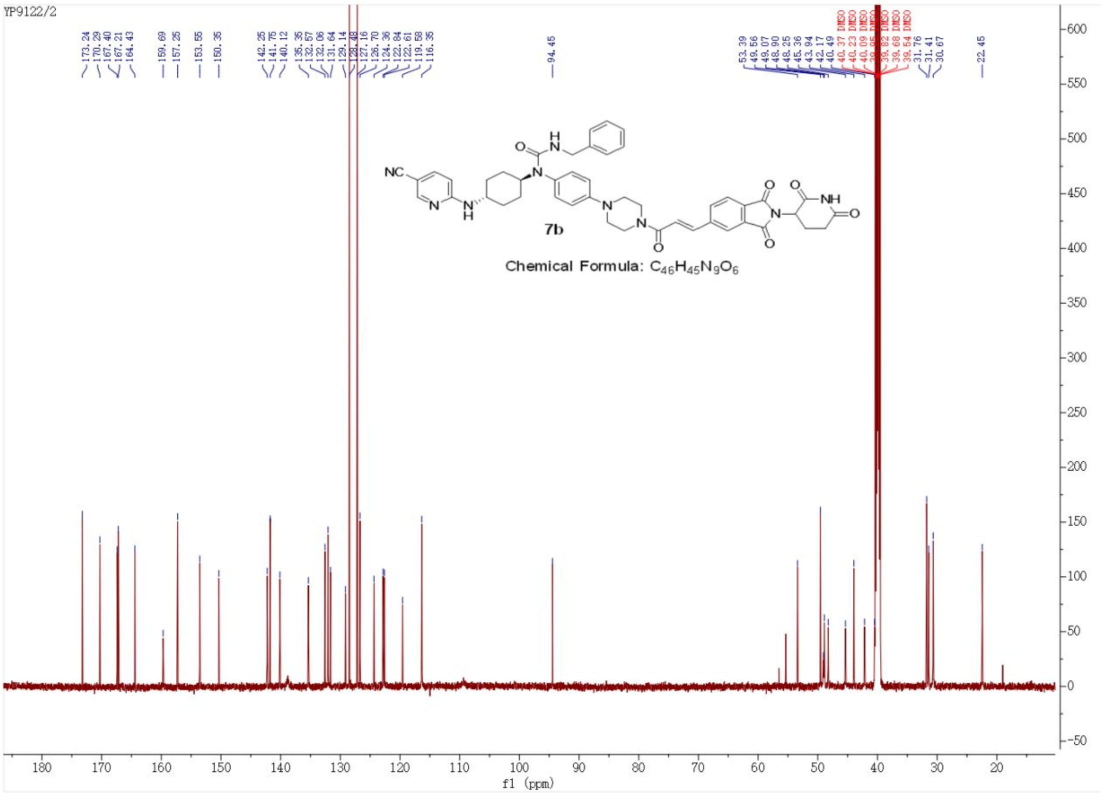

Hit	Formula	m/z	RDB	ppm	MS Rank	MSMS ppm	MSMS Rank	Found
1	C46H45N9O6	820.3566	29.0	-1.8	1			NA/NA

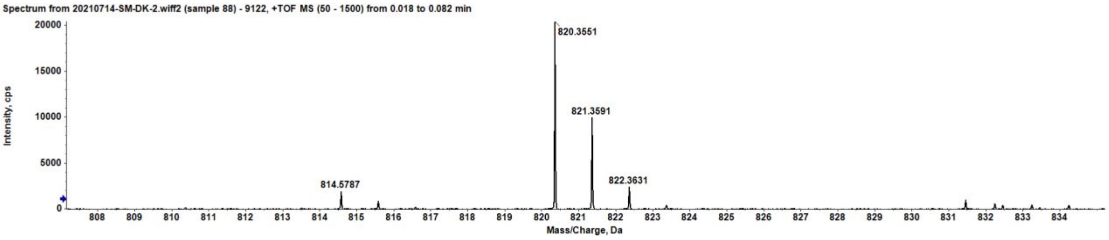


```
Data File E:\DK\ZZ\20210624-ZZ-YJZ-ZFT\YJZ1901DEF_LC 2021-06-24 20-23-22\9122-.D
Sample Name: 9122-

    =====================================================================
    Acq. Operator    : 系统                              Seq. Line :  15
    Acq. Instrument : 1260LC                             Location :   14
    Injection Date  : 25/06/2021 04:58:07                     Inj :   1
                                                   Inj Volume : 10.000 µl
    Different Inj Volume from Sample Entry!  Actual Inj Volume : 8.000 µl
    Acq. Method     : E:\DK\ZZ\20210624-ZZ-YJZ-ZFT\YJZ1901DEF_LC 2021-06-24 20-23-22\75C-25A-
                      30min-1u.M
    Last changed    : 24/06/2021 20:23:22 by 系统
    Analysis Method : E:\DK\ZZ\20210624-ZZ-YJZ-ZFT\YJZ1901DEF_LC 2021-06-24 20-23-22\75C-25A-
                      30min-1u.M (Sequence Method)
    Last changed    : 26/06/2021 10:55:14 by 系统
```

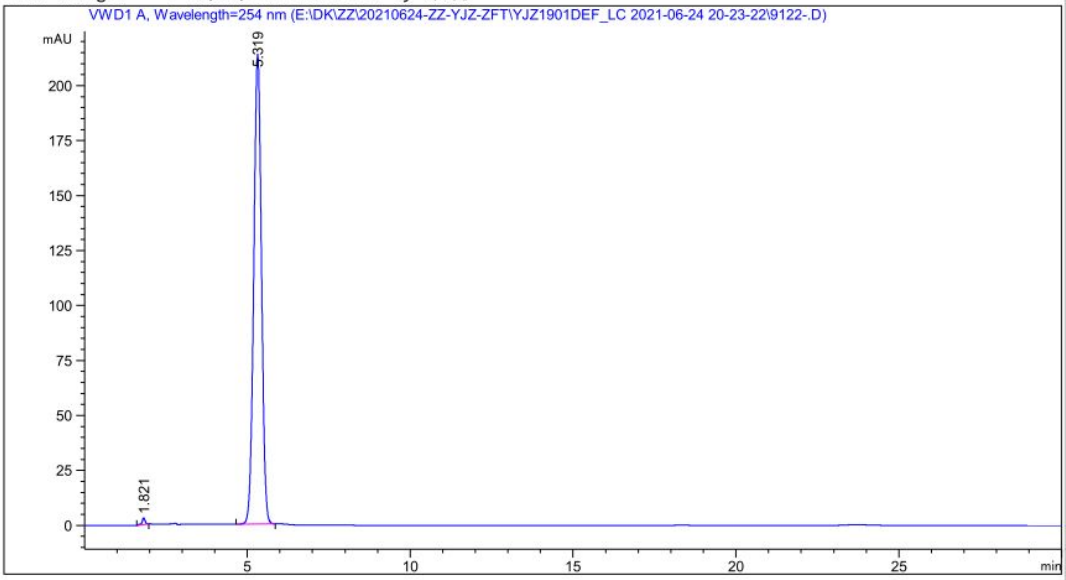


```
    =====================================================================
                         Area Percent Report
    =====================================================================

    Sorted By             :      Signal
    Multiplier            :      1.0000
    Dilution              :      1.0000
    Use Multiplier & Dilution Factor with ISTDs


    Signal 1: VWD1 A, Wavelength=254 nm

    Peak RetTime Type  Width     Area      Height     Area
      #   [min]        [min]   [mAU*s]     [mAU]        %
    ----|-------|----|-------|----------|----------|--------|
       1   1.821 VB R  0.0948   19.25407    3.04563   0.5458
       2   5.319 BB    0.2557 3508.68384  213.56126  99.4542
```

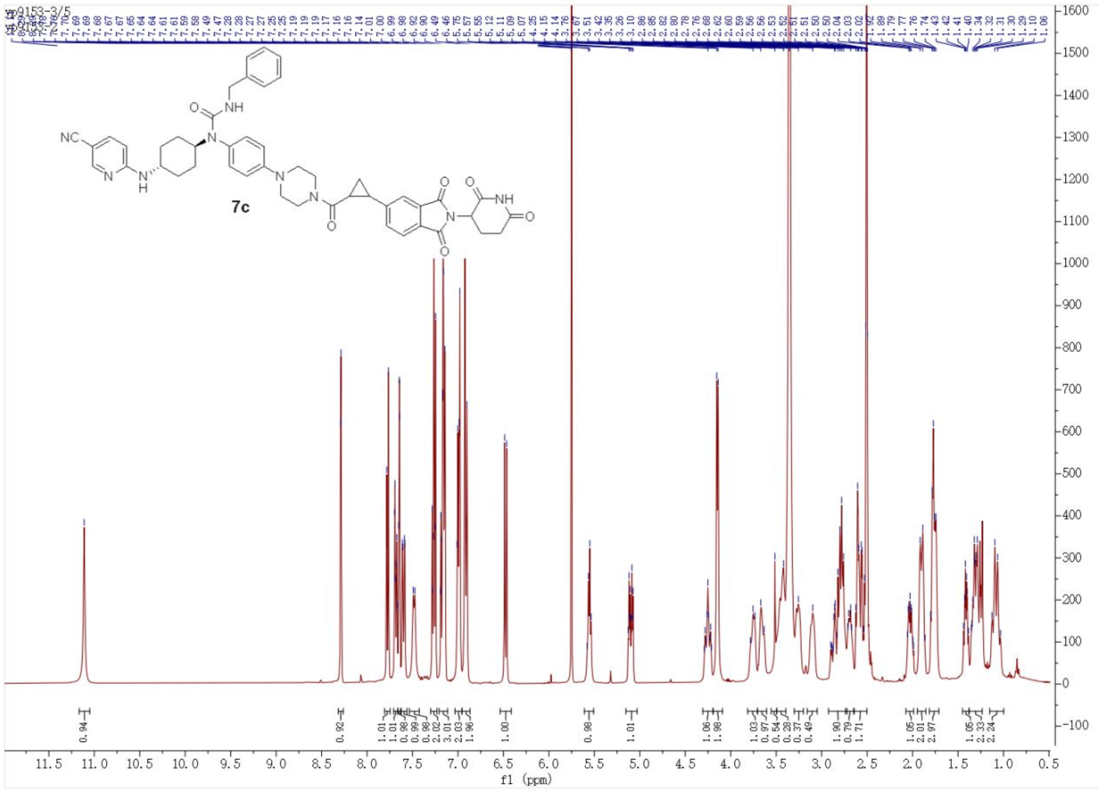

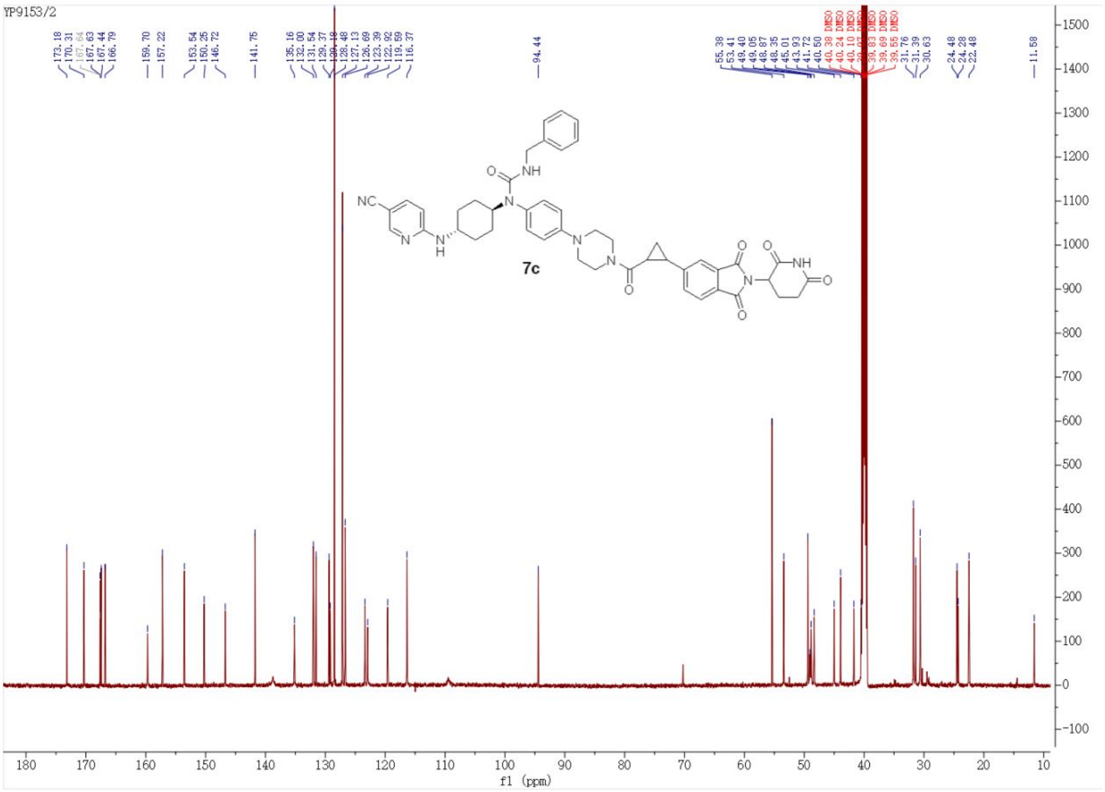

Hit	Formula	m/z	RDB	ppm	MS Rank	MSMS ppm	MSMS Rank	Found
1	C47H47N9O6	834.3722	29.0	-4.1	1			NA/NA

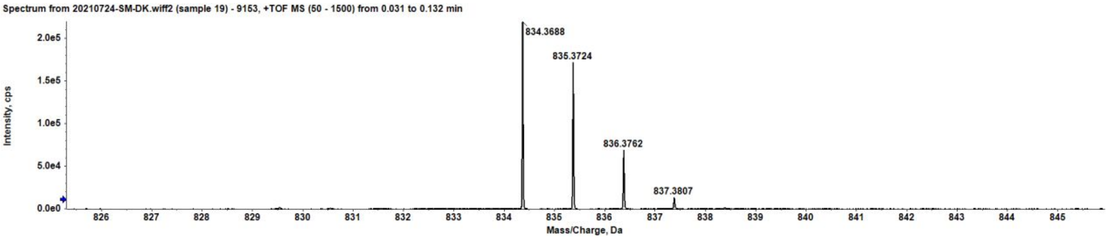

Data File E:\DK\YJZ\data\20210715\YJZ1901DEF_LC 2021-07-16 22-36-17\9153.D
Sample Name: 9153

===
Acq. Operator : 系统 Seq. Line : 2
Acq. Instrument : 1260LC Location : 3
Injection Date : 16/07/2021 23:07:50 Inj : 1
Inj Volume : 5.000 µl
Sequence File : E:\DK\YJZ\data\20210715\YJZ1901DEF_LC 2021-07-16 22-36-17\YJZ1901DEF_LC.S
Method : E:\DK\YJZ\data\20210715\YJZ1901DEF_LC 2021-07-16 22-36-17\70b-30a-30MIN.M (Sequence Method)
Last changed : 16/07/2021 22:36:17 by 系统

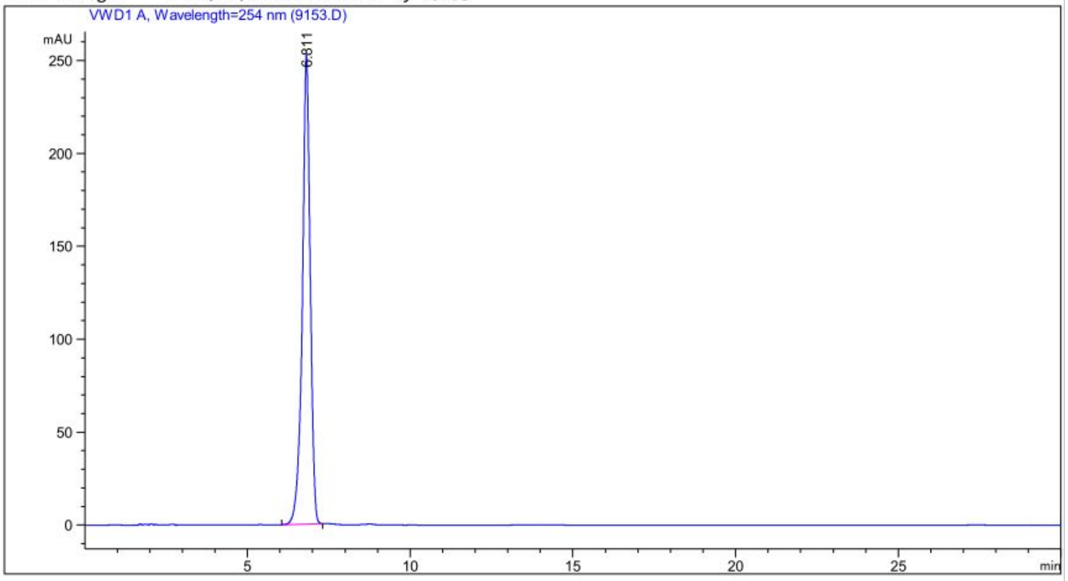

===
Area Percent Report
===

Sorted By : Signal
Multiplier : 1.0000
Dilution : 1.0000
Use Multiplier & Dilution Factor with ISTDs

Signal 1: VWD1 A, Wavelength=254 nm

Peak #	RetTime [min]	Type	Width [min]	Area [mAU*s]	Height [mAU]	Area %
1	6.811	BB	0.2453	4249.19727	252.49466	100.0000

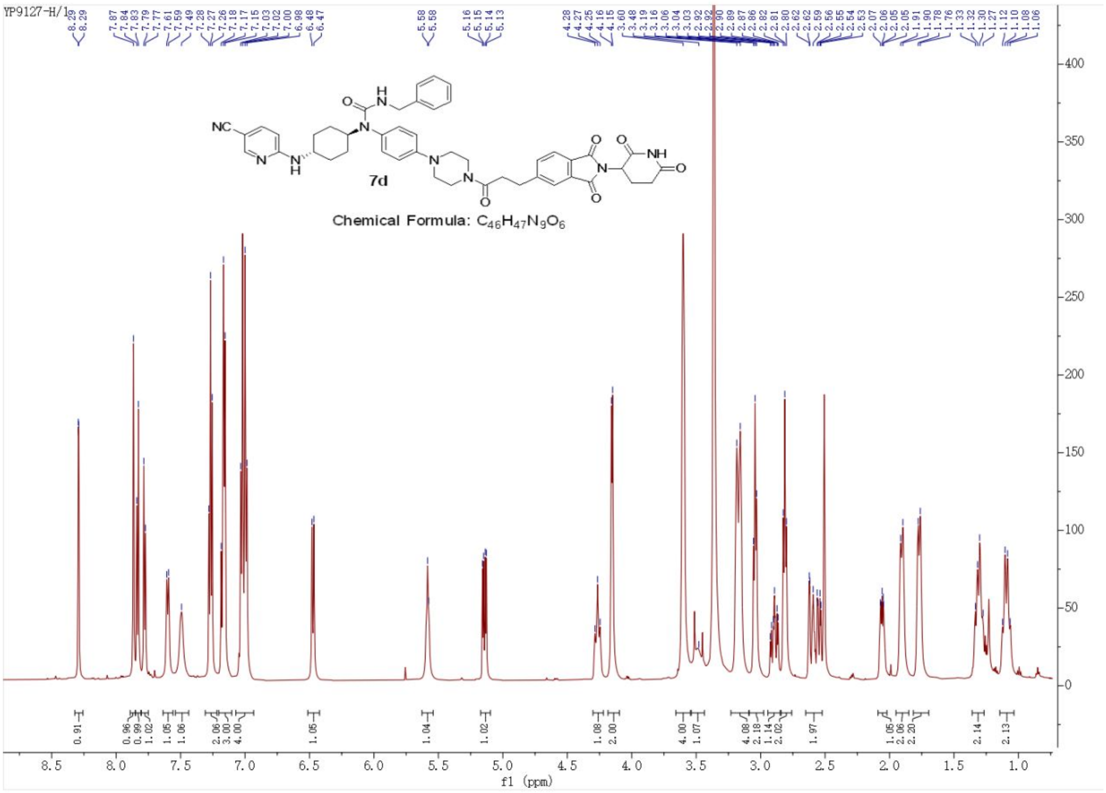
YP9127-H/1
Chemical Formula: $C_{46}H_{47}N_9O_6$
7d
f1 (ppm)

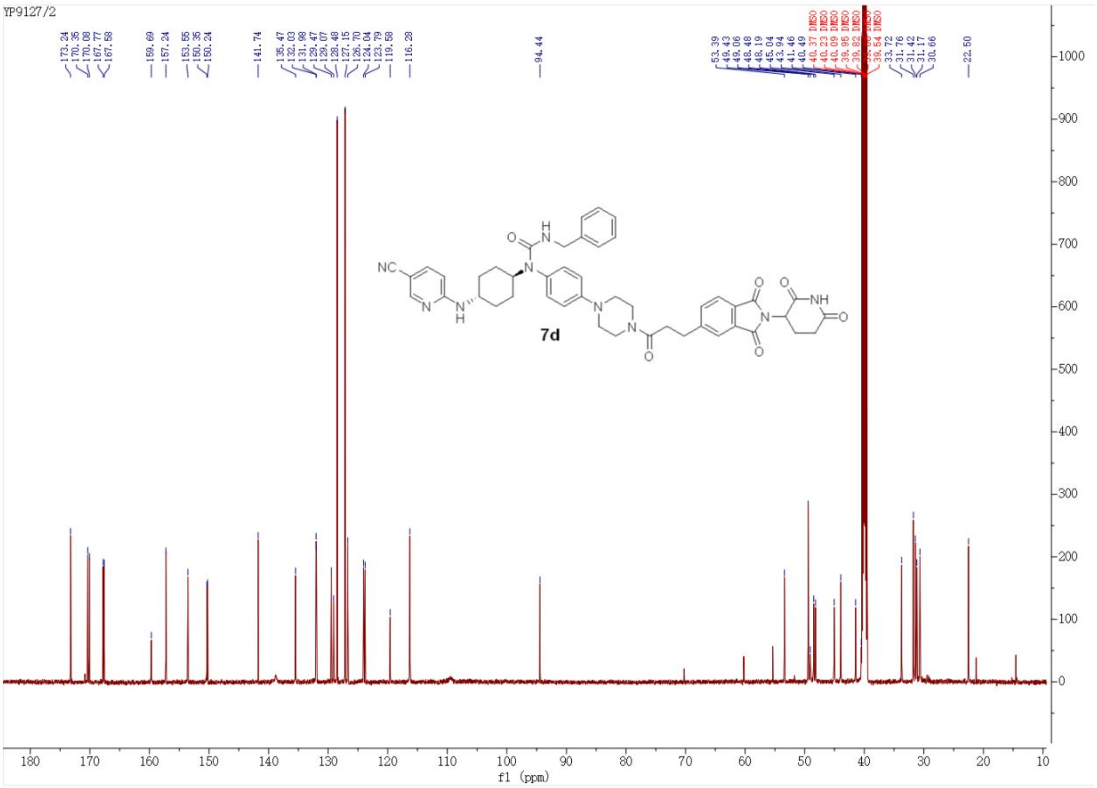
YP9127/2
7d
f1 (ppm)

Hit	Formula	m/z	RDB	ppm	MS Rank	MSMS ppm	MSMS Rank	Found
1	C46H47N9O6	822.3722	28.0	-3.7	1			NA/NA

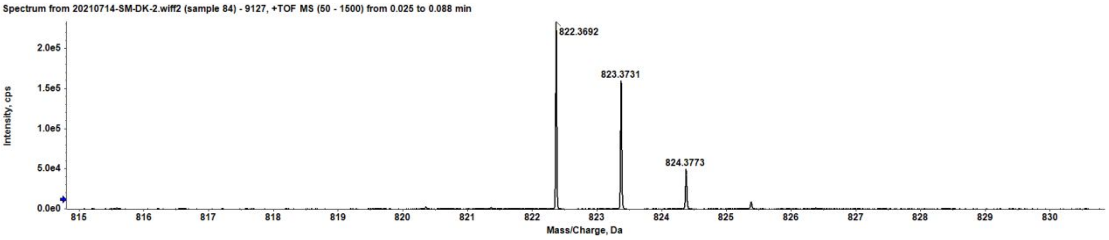


```
Data File E:\DK\ZZ\20210624-ZZ-YJZ-ZFT\YJZ1901DEF_LC 2021-06-24 20-23-22\9127.D
Sample Name: 9127

    =====================================================================
    Acq. Operator   : 系统                          Seq. Line :  16
    Acq. Instrument : 1260LC                         Location :   15
    Injection Date  : 25/06/2021 05:28:56                 Inj :   1
                                                   Inj Volume : 10.000 µl
    Different Inj Volume from Sample Entry!  Actual Inj Volume : 8.000 µl
    Acq. Method     : E:\DK\ZZ\20210624-ZZ-YJZ-ZFT\YJZ1901DEF_LC 2021-06-24 20-23-22\75C-25A-
                      30min-1u.M
    Last changed    : 24/06/2021 20:23:22 by 系统
    Analysis Method : E:\DK\ZZ\20210624-ZZ-YJZ-ZFT\YJZ1901DEF_LC 2021-06-24 20-23-22\75C-25A-
                      30min-1u.M (Sequence Method)
    Last changed    : 26/06/2021 10:55:14 by 系统
```

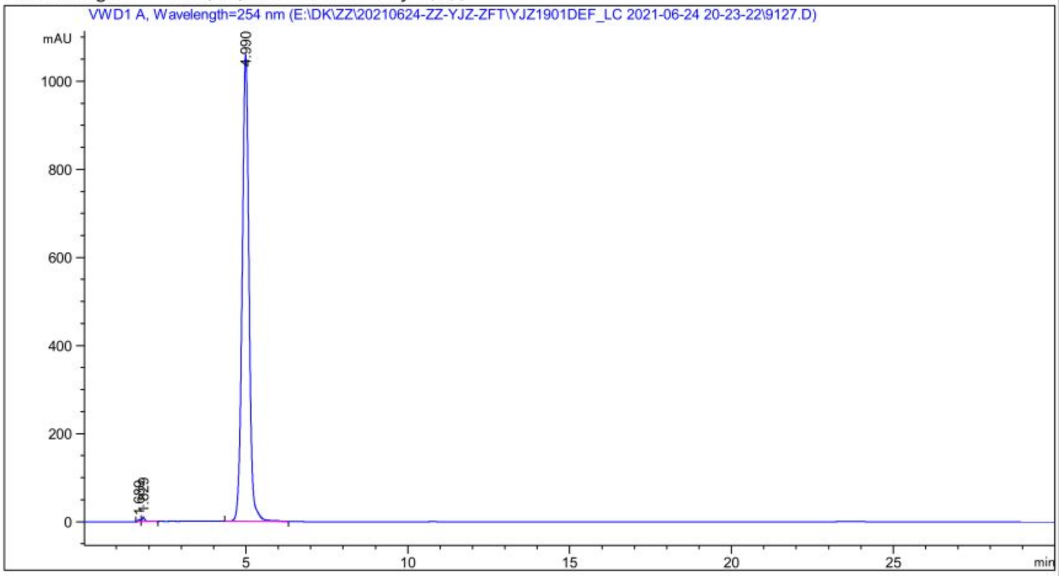


```
    =====================================================================
                             Area Percent Report
    =====================================================================

    Sorted By             :      Signal
    Multiplier            :      1.0000
    Dilution              :      1.0000
    Use Multiplier & Dilution Factor with ISTDs


    Signal 1: VWD1 A, Wavelength=254 nm

    Peak RetTime Type  Width     Area      Height     Area
      #   [min]        [min]   [mAU*s]     [mAU]        %
    ----|-------|----|-------|----------|----------|--------|
       1   1.689 BV    0.0825   24.66569    4.60498   0.1573
       2   1.829 VB    0.0901   64.63920   10.31032   0.4122
       3   4.990 BV R  0.2235 1.55929e4  1060.62671  99.4305
```

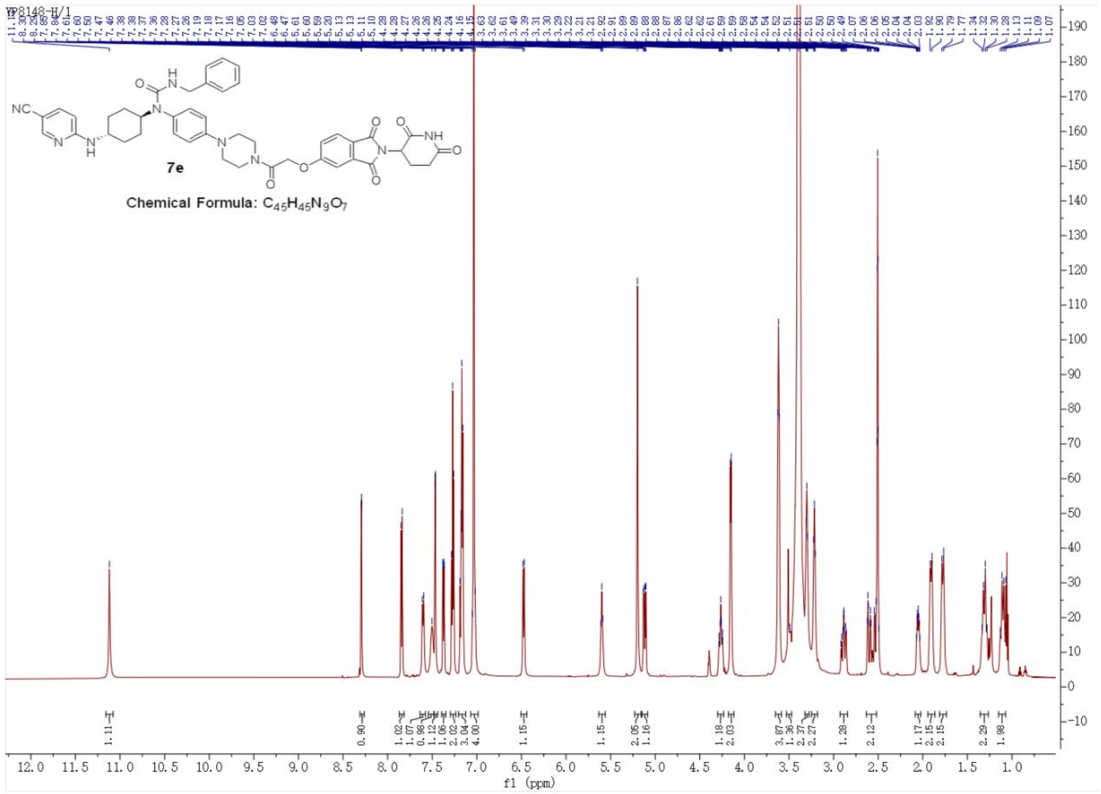
YP8148-H/1
7e
Chemical Formula: $C_{45}H_{45}N_9O_7$
f1 (ppm)

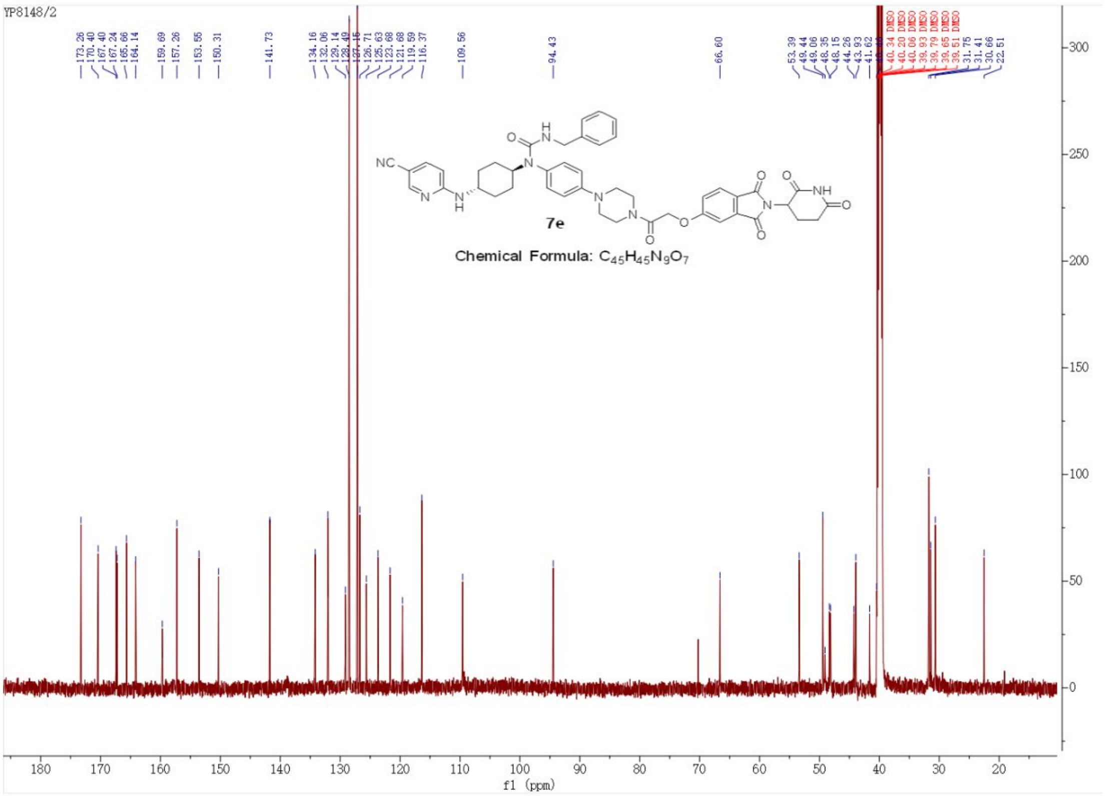
YP8148/2
7e
Chemical Formula: $C_{45}H_{45}N_9O_7$
f1 (ppm)

Hit	Formula	m/z	RDB	ppm	MS Rank	MSMS ppm	MSMS Rank	Found
1	C45H45N9O7	824.3515	28.0	-4.8	1			NA/NA

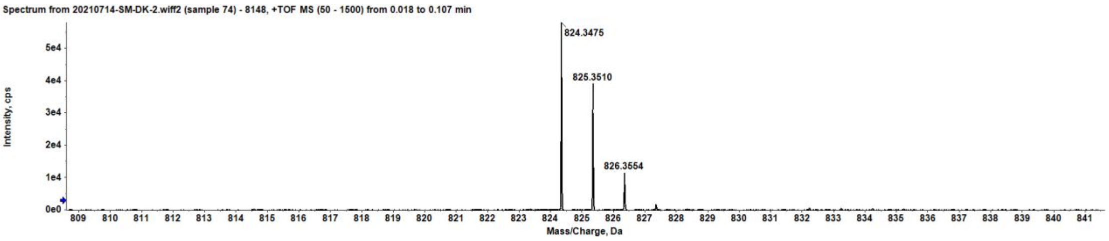

Data File E:\DK\YJZ\data\20210525\20210518 2021-05-25 19-42-00\8148.D
Sample Name: 8148

```
=====================================================================
Acq. Operator   : 系统                          Seq. Line :  12
Acq. Instrument : 1260LC                         Location :   92
Injection Date  : 26/05/2021 01:22:34                 Inj :   1
                                               Inj Volume : 5.000 µl
Different Inj Volume from Sample Entry!  Actual Inj Volume : 10.000 µl
Method          : E:\DK\YJZ\data\20210525\20210518 2021-05-25 19-42-00\78C-22A-30MIN-254NM.M
                  (Sequence Method)
Last changed    : 25/05/2021 19:42:00 by 系统
```

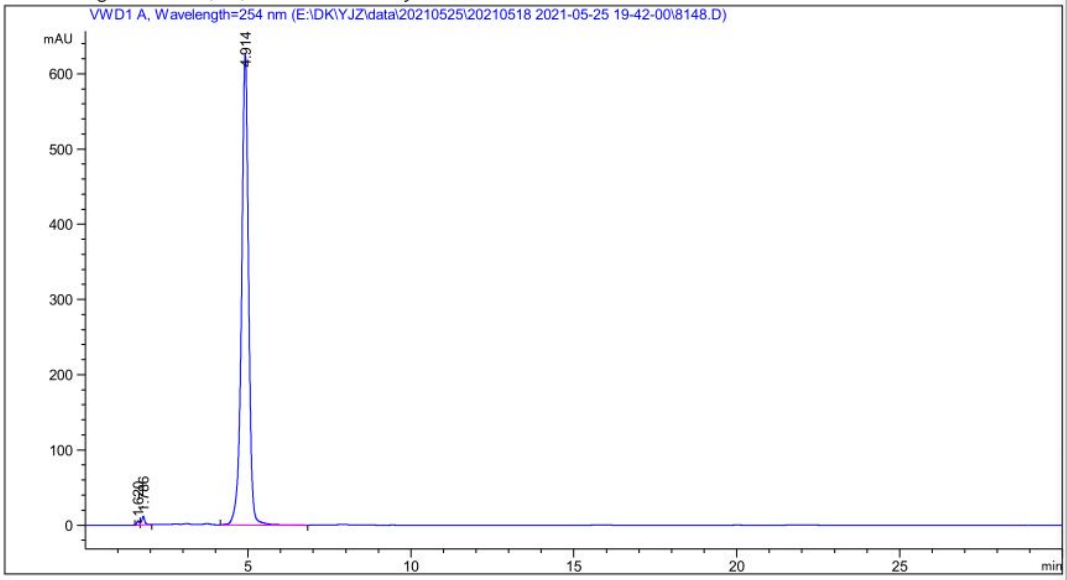


```
=====================================================================
                         Area Percent Report
=====================================================================

Sorted By             :      Signal
Multiplier            :      1.0000
Dilution              :      1.0000
Use Multiplier & Dilution Factor with ISTDs


Signal 1: VWD1 A, Wavelength=254 nm

Peak RetTime Type  Width     Area      Height     Area
  #   [min]        [min]   [mAU*s]     [mAU]        %
----|-------|----|-------|----------|----------|--------|
   1   1.620 BV    0.0815   29.53158    5.51427   0.3088
   2   1.786 VB    0.0904   74.81409   11.73323   0.7823
   3   4.914 BB    0.2274 9458.56445  625.40399  98.9089
```

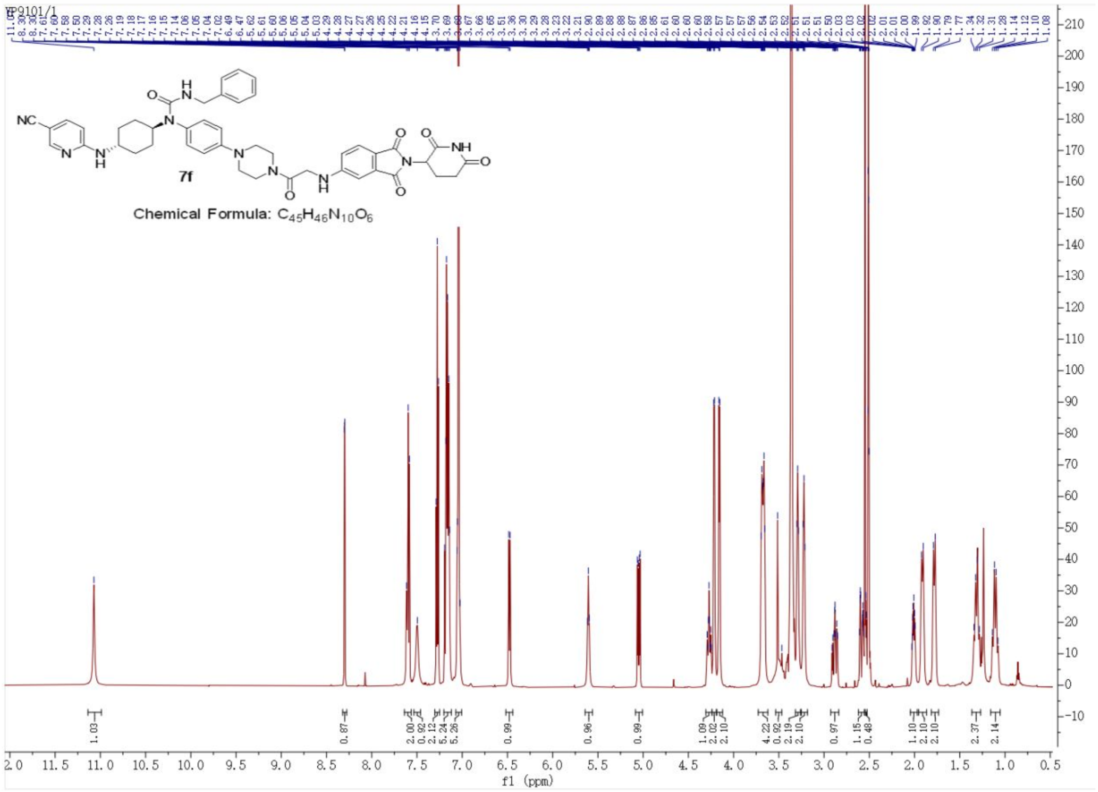

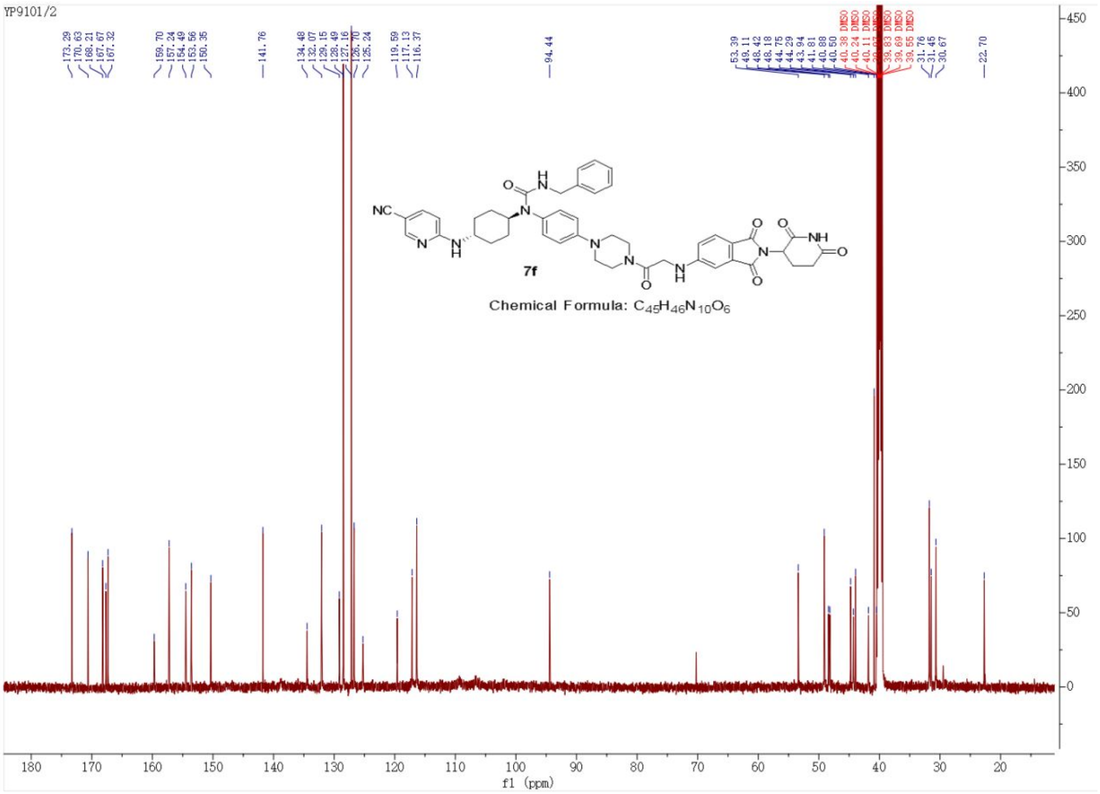

Hit	Formula	m/z	RDB	ppm	MS Rank	MSMS ppm	MSMS Rank	Found
1	C45H46N10O6	823.3675	28.0	-0.4	1			NA/NA

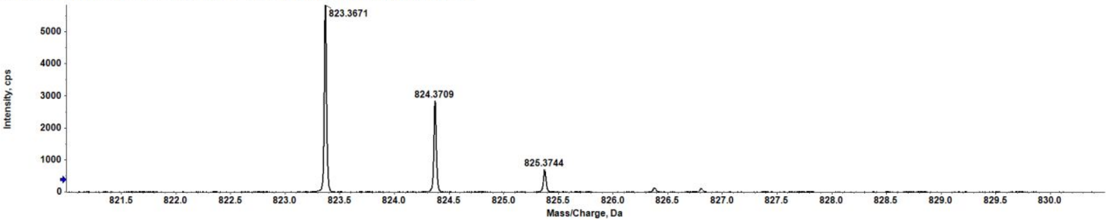

Data File E:\DK\YJZ\data\20210605\91011270.D
Sample Name: 9101

```
=====================================================================
Acq. Operator   : 系统
Sample Operator : 系统
Acq. Instrument : 1260LC                          Location :   87
Injection Date  : 05/06/2021 23:07:51
                                               Inj Volume : 5.000 µl
Acq. Method     : E:\DK\TL\方法\75C-25A-30min-1u.M
Last changed    : 05/06/2021 23:01:50 by 系统
                  (modified after loading)
Analysis Method : E:\DK\TL\方法\80C-20A-30min-1u.M
Last changed    : 06/04/2021 10:23:35 by 系统
```

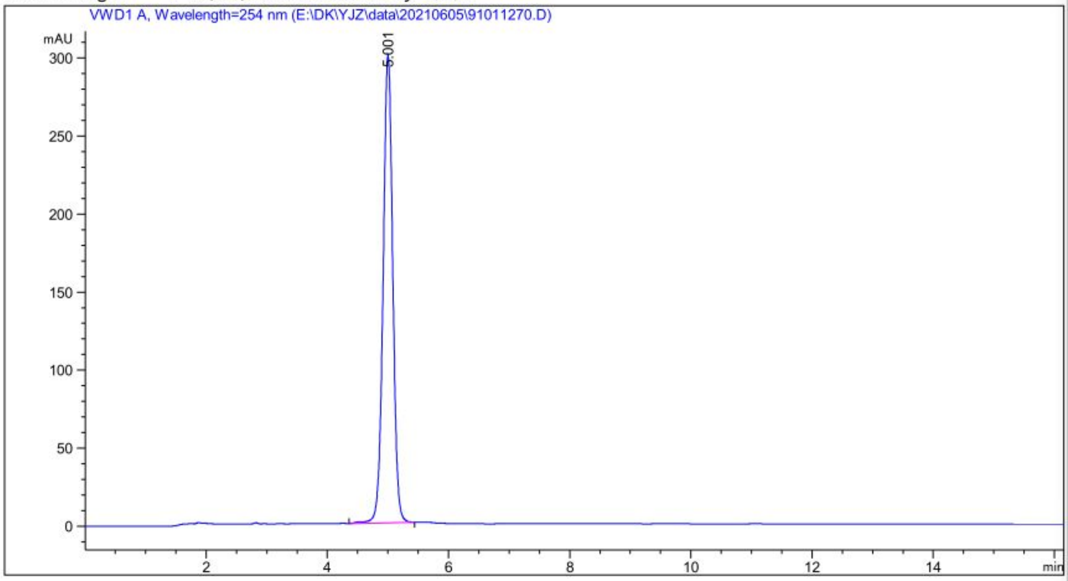


```
=====================================================================
                         Area Percent Report
=====================================================================

Sorted By             :      Signal
Multiplier            :      1.0000
Dilution              :      1.0000
Sample Amount:               :      5.00000  [ng/ul]   (not used in calc.)
Use Multiplier & Dilution Factor with ISTDs


Signal 1: VWD1 A, Wavelength=254 nm

Peak RetTime Type  Width     Area      Height     Area
  #   [min]        [min]   [mAU*s]     [mAU]        %
----|-------|----|-------|----------|----------|--------|
   1   5.001 BB    0.1699 3316.15039  300.15930 100.0000
```

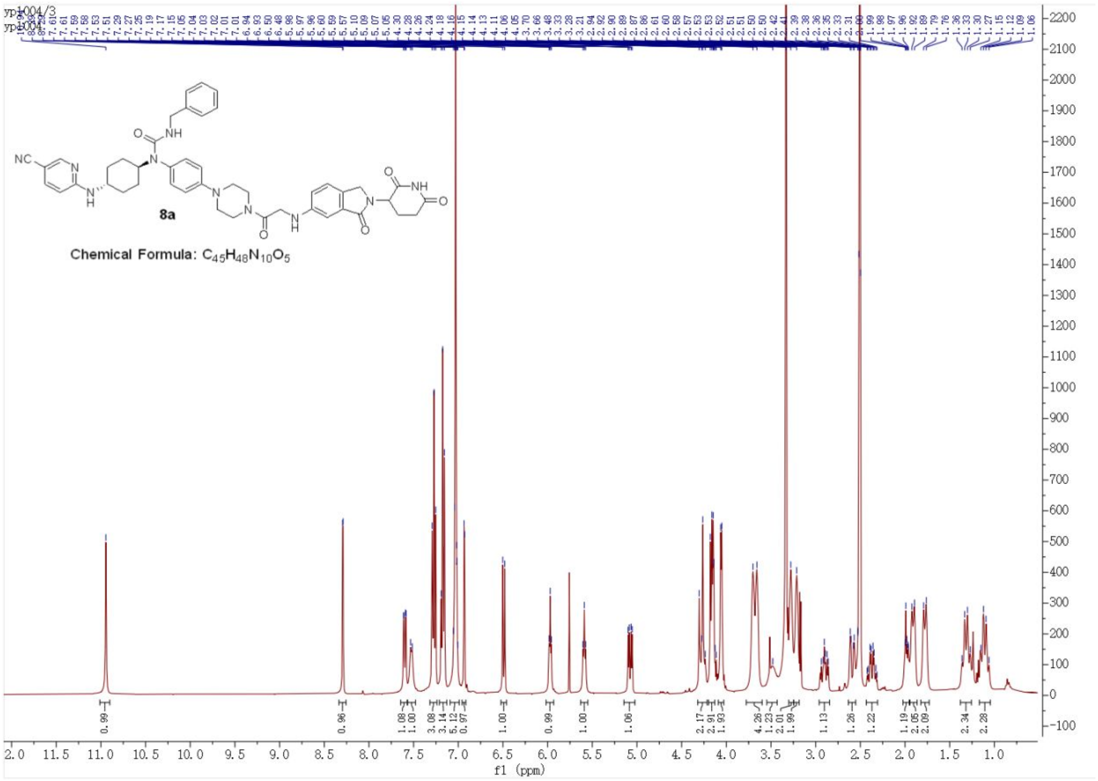

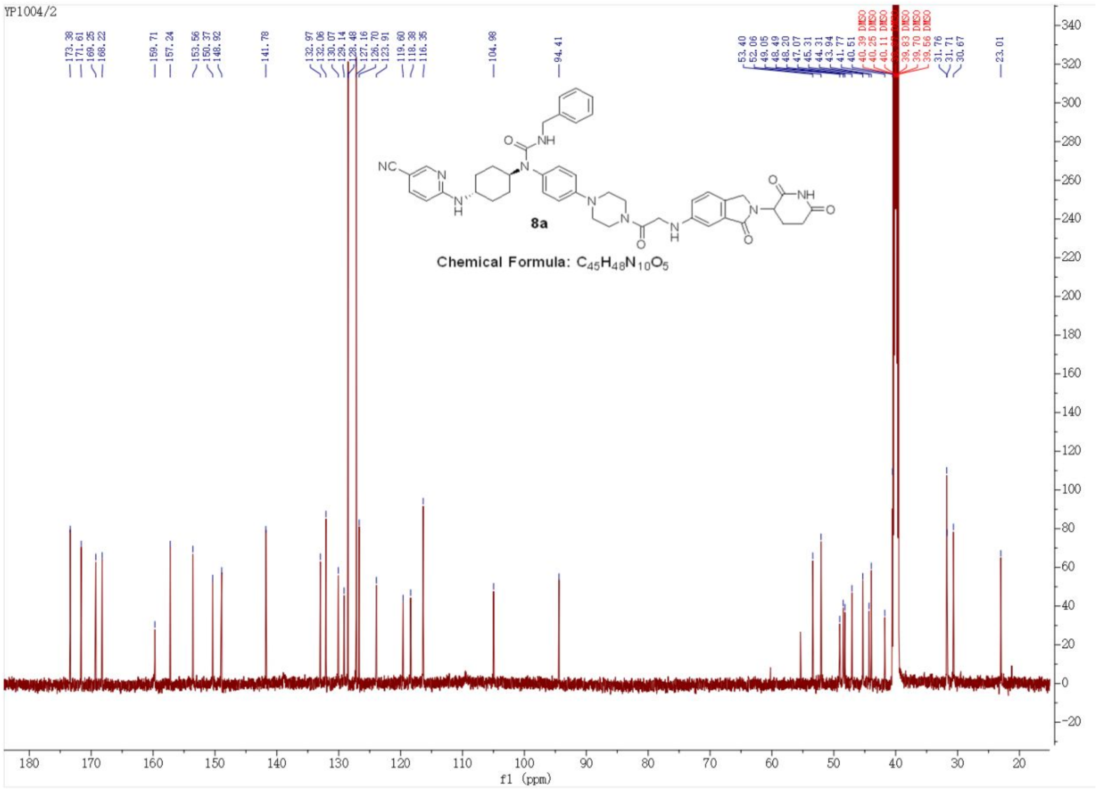

Hit	Formula	m/z	RDB	ppm	MS Rank	MSMS ppm	MSMS Rank	Found
1	C45H48N10O5	809.3882	27.0	-2.1	1			NA/NA

Spectrum from MASS20210907.wiff2 (sample 1) - 1004, +TOF MS (50 - 1000) from 0....S20210907.wiff2 (sample 1) - 1004, +TOF MS (50 - 1000) from 0.290 to 0.387 min]

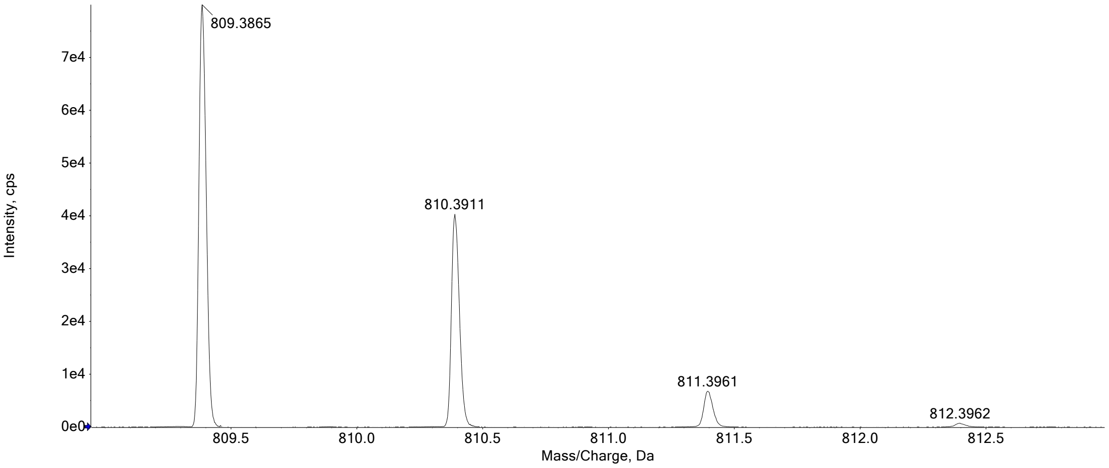


```
Data File E:\DK\YJZ\data\20210831\1004.D
Sample Name: 1004

   =====================================================================
   Acq. Operator   : 系统
   Sample Operator : 系统
   Acq. Instrument : 1260LC                          Location :   1
   Injection Date  : 31/08/2021 19:54:06
                                                   Inj Volume : 5.000 µl
   Method          : E:\DK\TL\方法\70C-30D-30min-1u.M
   Last changed    : 20/11/2019 13:02:51 by 系统
```

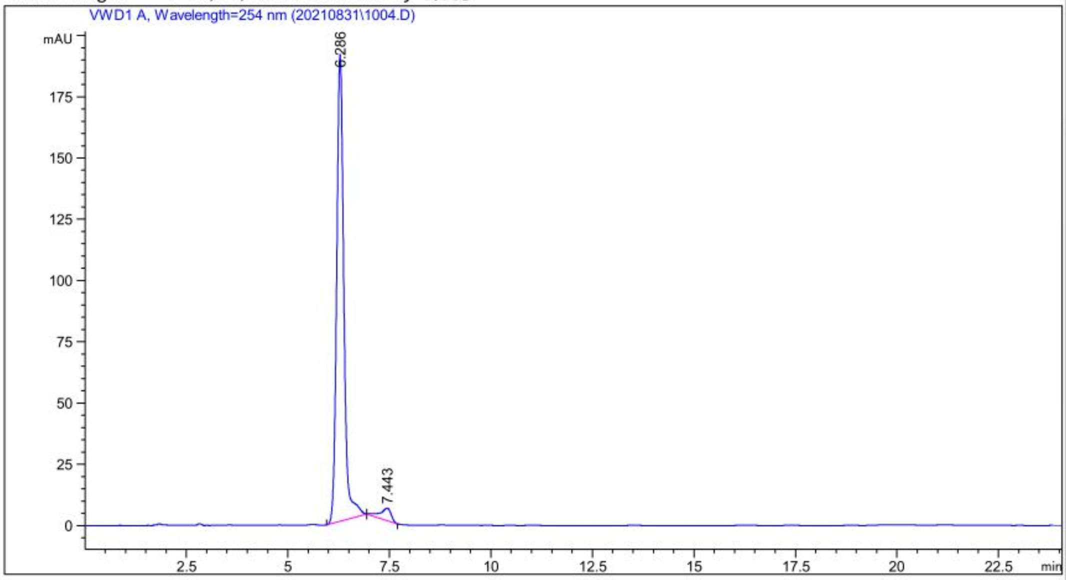


```
   =====================================================================
                          Area Percent Report
   =====================================================================

   Sorted By             :      Signal
   Multiplier            :      1.0000
   Dilution              :      1.0000
   Sample Amount:               :      10.00000  [ng/ul]   (not used in calc.)
   Use Multiplier & Dilution Factor with ISTDs


   Signal 1: VWD1 A, Wavelength=254 nm
```

Peak #	RetTime [min]	Type	Width [min]	Area [mAU*s]	Height [mAU]	Area %
1	6.286	BB	0.1862	2320.28662	190.35840	96.2324
2	7.443	BB	0.2599	90.84286	5.04946	3.7676

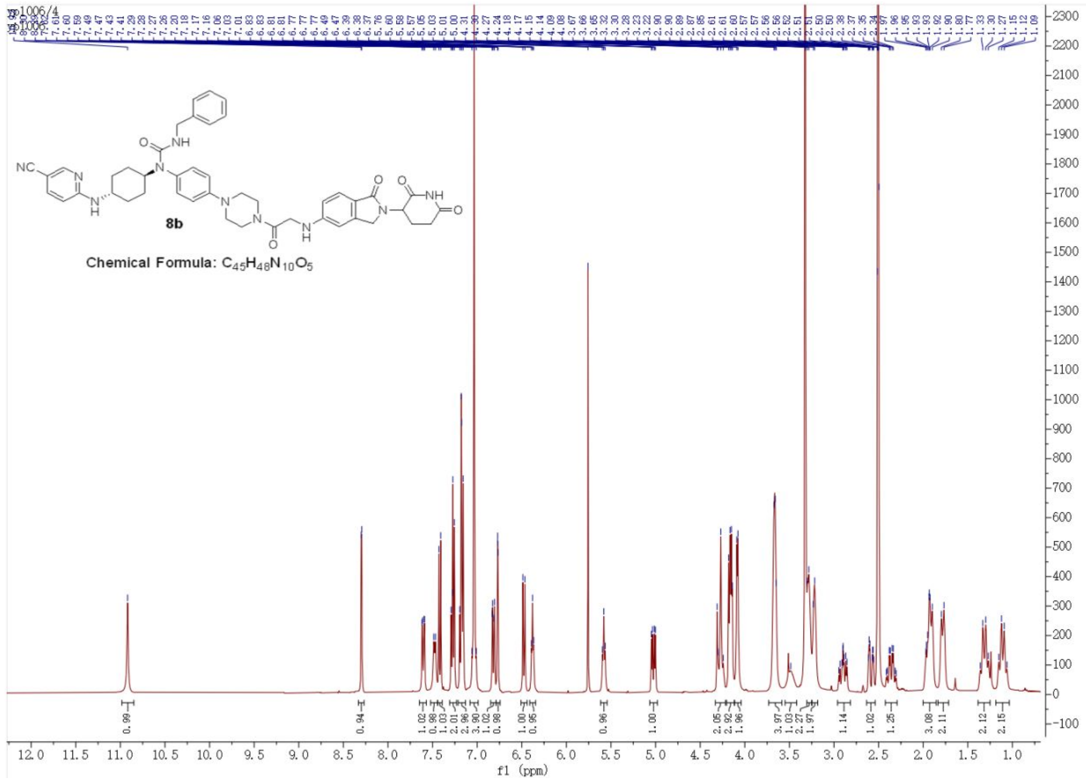

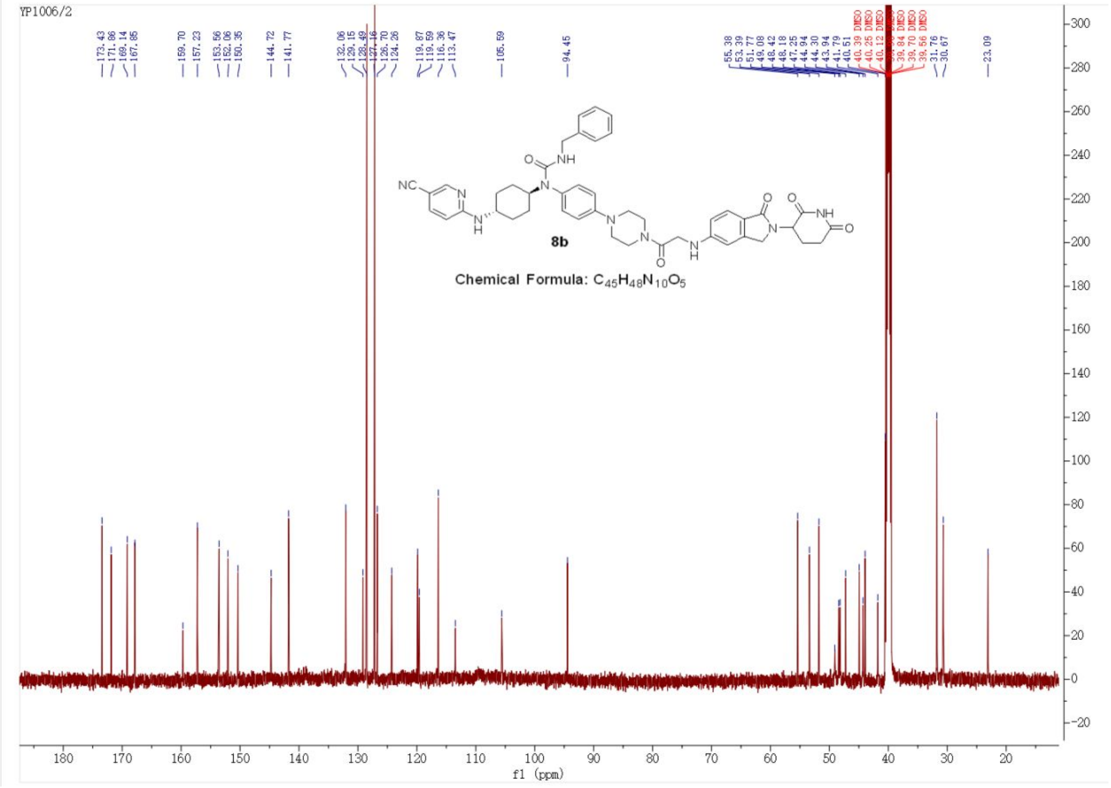

Hit	Formula	m/z	RDB	ppm	MS Rank	MSMS ppm	MSMS Rank	Found
1	C45H48N10O5	809.3882	27.0	1.9	1			NA/NA

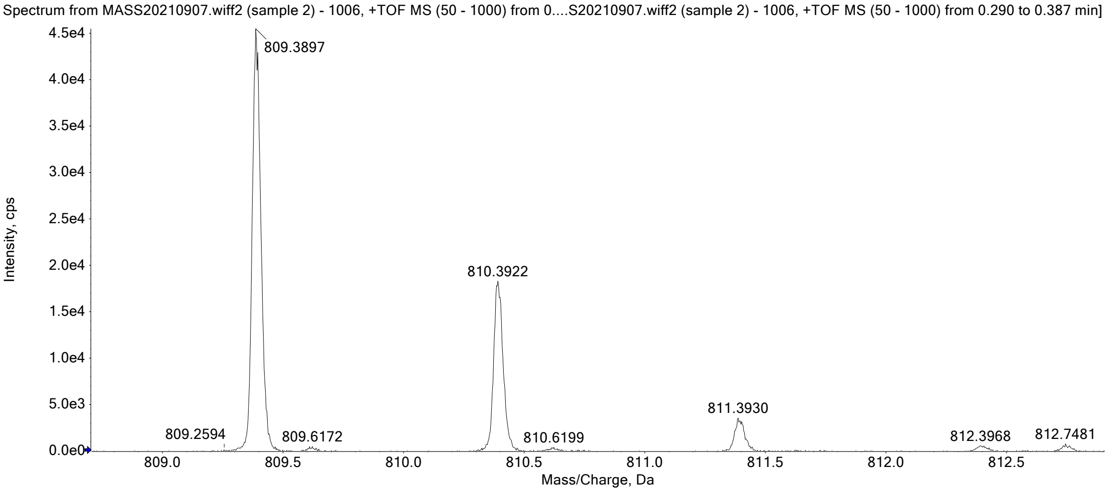

Data File E:\DK\YJZ\data\20210830\9001.D
Sample Name: 9001

===

Acq. Operator : 系统
Sample Operator : 系统
Acq. Instrument : 1260LC Location : 1
Injection Date : 30/08/2021 12:30:48
Inj Volume : 5.000 µl
Method : C:\CHEM32\1\METHODS\70C-30D-254NM.M
Last changed : 21/06/2021 10:31:49 by 系统

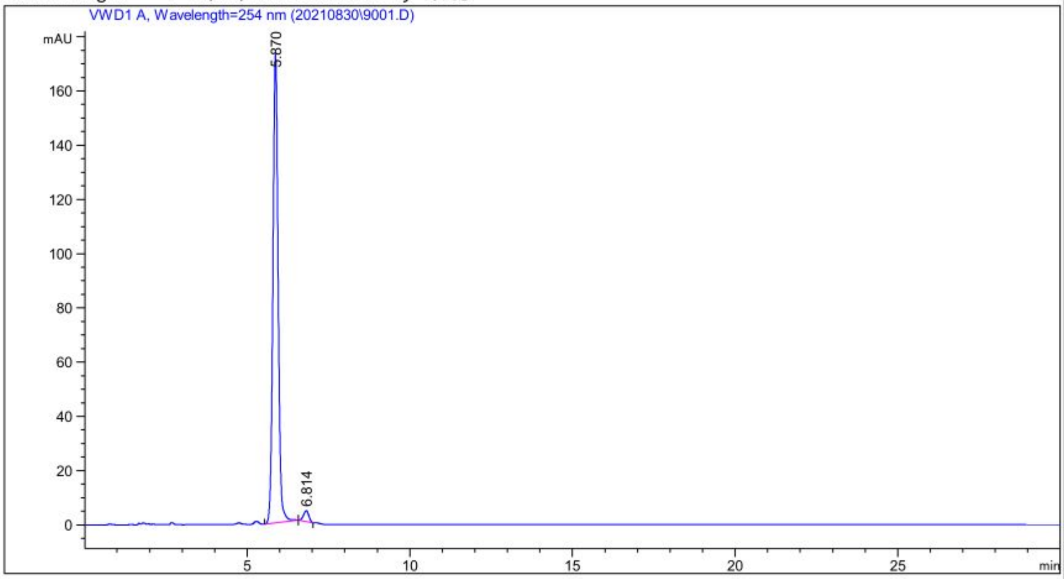

===

Area Percent Report

===

Sorted By : Signal
Multiplier : 1.0000
Dilution : 1.0000
Sample Amount: : 10.00000 [ng/ul] (not used in calc.)
Use Multiplier & Dilution Factor with ISTDs

Signal 1: VWD1 A, Wavelength=254 nm

Peak #	RetTime [min]	Type	Width [min]	Area [mAU*s]	Height [mAU]	Area %
1	5.870	BB	0.1705	1915.91248	172.65315	97.6814
2	6.814	BB	0.1798	45.47676	3.99442	2.3186

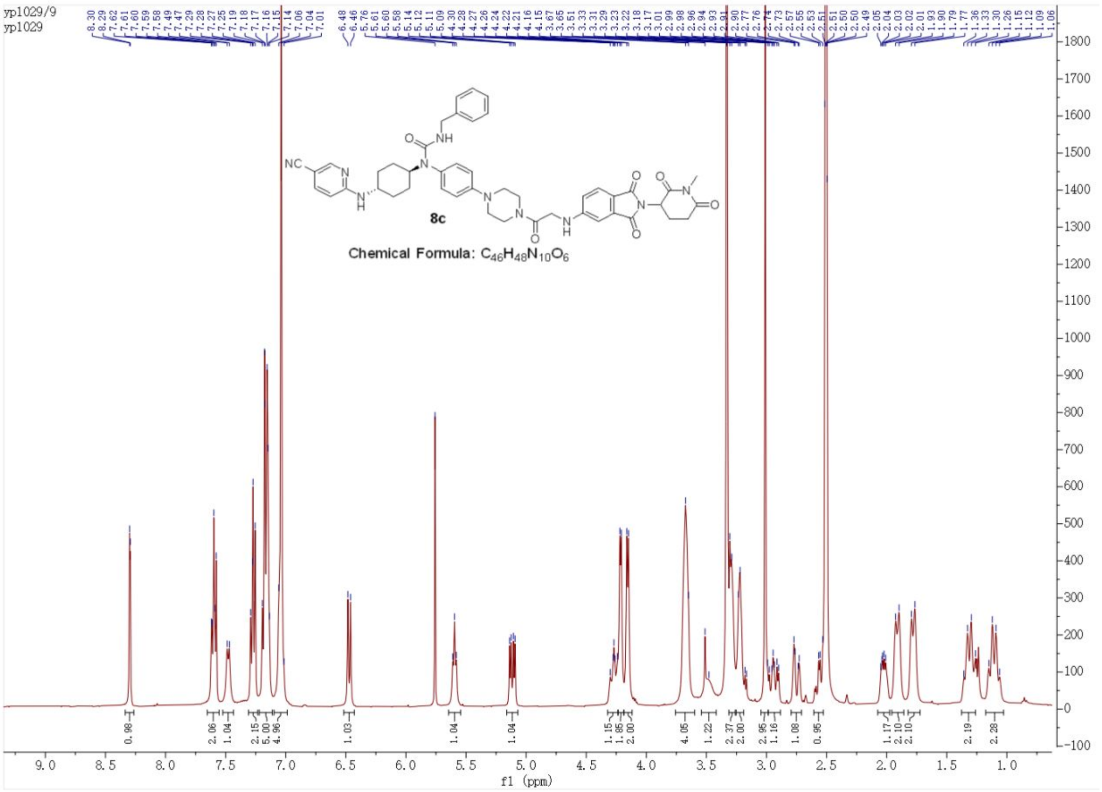
yp1029/9
yp1029
8c
Chemical Formula: $C_{46}H_{48}N_{10}O_6$
f1 (ppm)

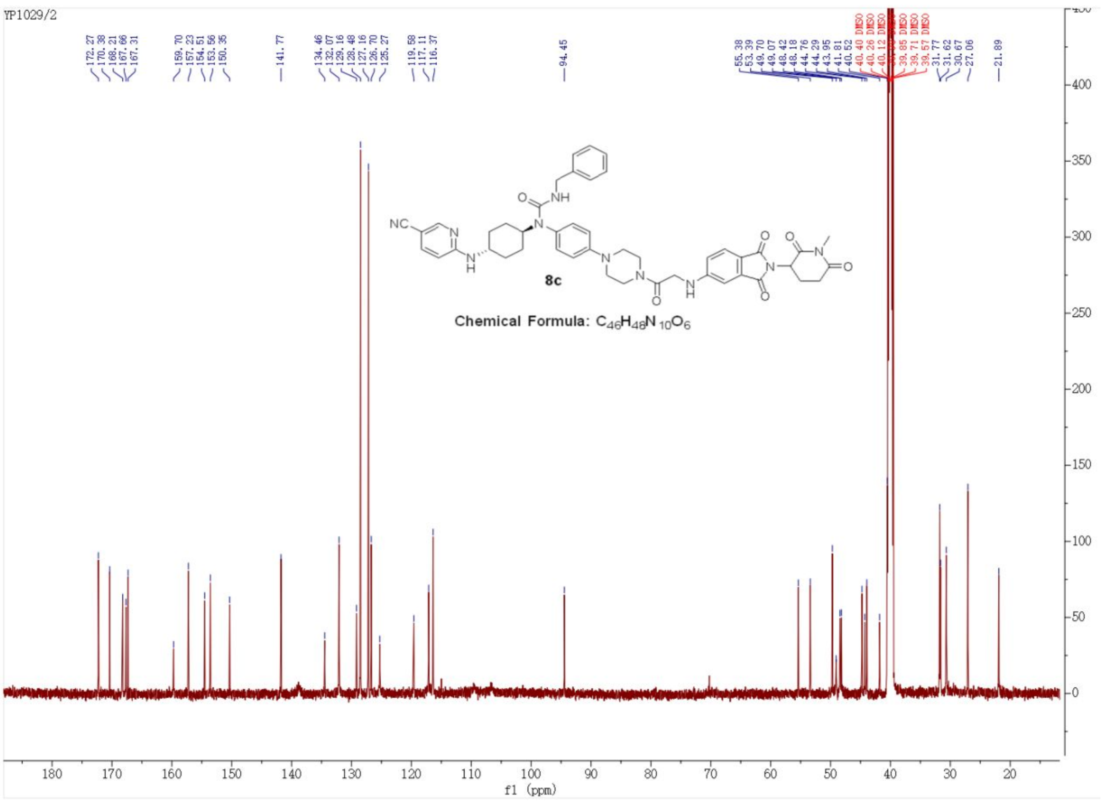
YP1029/2
8c
Chemical Formula: $C_{46}H_{48}N_{10}O_6$
f1 (ppm)

Hit	Formula	m/z	RDB	ppm	MS Rank	MSMS ppm	MSMS Rank	Found
1	C46H48N10O6	837.3831	28.0	-3.2	1			NA/NA

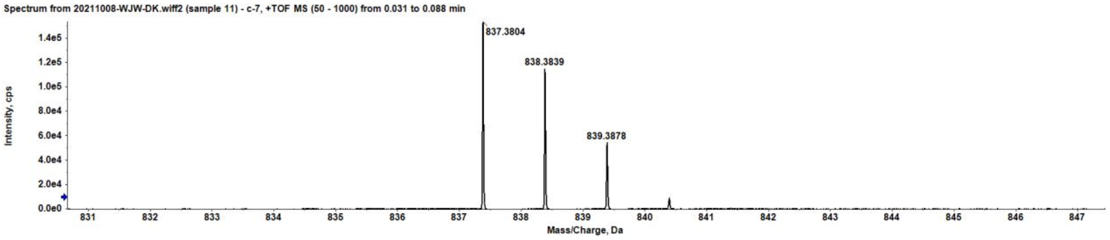

Data File E:\DK\YJZ\data\20211102\7219系统.D
Sample Name: 1029

===
Acq. Operator : 系统
Sample Operator : 系统
Acq. Instrument : 1260LC Location : 41
Injection Date : 02/11/2021 16:06:29
Inj Volume : 10.000 µl
Acq. Method : E:\DK\TL\方法\70C-30D-30min-1u.M
Last changed : 02/11/2021 16:05:10 by 系统
(modified after loading)
Analysis Method : C:\Chem32\1\Methods\DEF_LC.M
Last changed : 23/06/2014 04:13:01 by SYSTEM

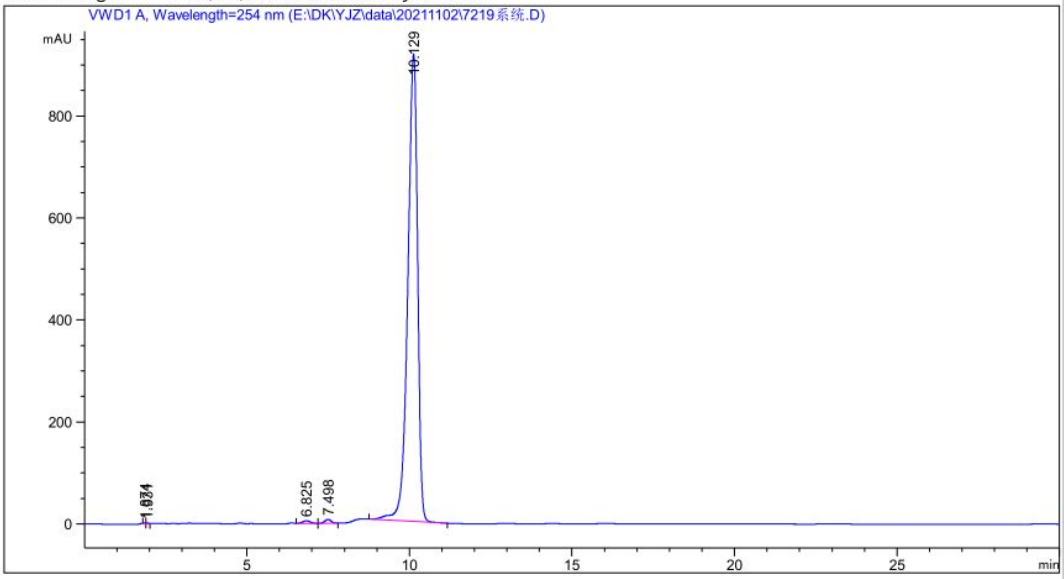

===
Area Percent Report
===

Sorted By : Signal
Multiplier : 1.0000
Dilution : 1.0000
Sample Amount: : 10.00000 [ng/ul] (not used in calc.)
Use Multiplier & Dilution Factor with ISTDs

Signal 1: VWD1 A, Wavelength=254 nm

Peak #	RetTime [min]	Type	Width [min]	Area [mAU*s]	Height [mAU]	Area %
1	1.874	VV	0.0696	9.65067	2.02643	0.0496
2	1.931	VV	0.0727	9.47954	1.91564	0.0487
3	6.825	VV	0.2611	93.33497	5.41608	0.4796
4	7.498	VB	0.2086	102.54706	7.68736	0.5269
5	10.129	BB	0.3232	1.92461e4	915.73523	98.8952
